# Genetic Architecture of Abdominal Pigmentation in *Drosophila melanogaster*

**DOI:** 10.1371/journal.pgen.1005163

**Published:** 2015-05-01

**Authors:** Lauren M. Dembeck, Wen Huang, Michael M. Magwire, Faye Lawrence, Richard F. Lyman, Trudy F. C. Mackay

**Affiliations:** 1 Department of Biological Sciences, North Carolina State University, Raleigh, North Carolina, United States of America; 2 Program in Genetics, North Carolina State University, Raleigh, North Carolina, United States of America; 3 W. M. Keck Center for Behavioral Biology, North Carolina State University, Raleigh, North Carolina, United States of America; 4 Syngenta Biotechnology, Durham, North Carolina, United States of America; The University of North Carolina at Chapel Hill, UNITED STATES

## Abstract

Pigmentation varies within and between species and is often adaptive. The amount of pigmentation on the abdomen of *Drosophila melanogaster* is a relatively simple morphological trait, which serves as a model for mapping the genetic basis of variation in complex phenotypes. Here, we assessed natural variation in female abdominal pigmentation in 175 sequenced inbred lines of the *Drosophila melanogaster* Genetic Reference Panel, derived from the Raleigh, NC population. We quantified the proportion of melanization on the two most posterior abdominal segments, tergites 5 and 6 (T5, T6). We found significant genetic variation in the proportion of melanization and high broad-sense heritabilities for each tergite. Genome-wide association studies identified over 150 DNA variants associated with the proportion of melanization on T5 (84), T6 (34), and the difference between T5 and T6 (35). Several of the top variants associated with variation in pigmentation are in *tan*, *ebony*, and *bric-a-brac1*, genes known to affect *D*. *melanogaster* abdominal pigmentation. Mutational analyses and targeted RNAi-knockdown showed that 17 out of 28 (61%) novel candidate genes implicated by the genome-wide association study affected abdominal pigmentation. Several of these genes are involved in developmental and regulatory pathways, chitin production, cuticle structure, and vesicle formation and transport. These findings show that genetic variation may affect multiple steps in pathways involved in tergite development and melanization. Variation in these novel candidates may serve as targets for adaptive evolution and sexual selection in *D*. *melanogaster*.

## Author Summary

Body pigmentation contributes to the spectacular biodiversity present in nature and mediates mate choice, mimicry, and physiological functions such as thermoregulation and UV resistance. Thus, pigmentation is a significant contributor to fitness. In order to understand how complex traits such as pigmentation evolve, we must first identify the genetic variants underlying phenotypic variation. We used the *Drosophila melanogaster* Genetic Reference Panel, a wild derived population of fully sequenced inbred fly lines, to identify the contributions of both known and novel genetic variants to natural variation in abdominal pigmentation in female flies. Our results show that genetic variation within many biological pathways contributes to variation in *D*. *melanogaster* pigmentation.

## Introduction

Body pigmentation is a conspicuous trait that is variable within species, giving rise to natural variation, polyphenism and sexual dimorphism [1–4]. It also varies between species, contributing to species recognition, mate choice, thermoregulation, protection (warning signals), mimicry, and crypsis [5–7]. Changes in pigmentation are often adaptive and vital to the fitness of the organism [5,6].

Not only is body pigmentation ecologically relevant, in *Drosophila* it is a relatively simple and easily measured phenotype to study the genetic architecture of natural variation in complex traits [2,7–10]. Each tergite of female *D*. *melanogaster* generally has a stripe of dark coloration (melanin) on a lighter tan background (sclerotin). During pre- and post-ecdysis, the epidermal cells underlying the cuticle secrete tyrosine-derived catecholamines into the cuticle for sclerotinization and melanization [11,12]. The melanin/sclerotin biosynthetic pathway and its underlying genetic basis have been well studied. However, many of the genes known to affect *D*. *melanogaster* pigmentation do not form part of this pathway or any parallel pathway [5,13]. Furthermore, the genes that lead to natural variation in body pigmentation are not necessarily the same genes that are directly involved in the biosynthesis of melanin and sclerotin. By mapping the genetic basis of natural variation in body pigmentation, we may discover new genes affecting pigment biosynthesis as well as regulatory regions that determine when and where pigmentation will develop [3,13].

We used the *D*. *melanogaster* Genetic Reference Panel (DGRP) to perform a genome-wide association (GWA) study of natural variation in the proportion of melanization on female abdominal tergites 5 and 6. The DGRP consists of 205 sequenced inbred lines derived from a single North American population, facilitating GWA analyses for quantitative traits when all genetic variants are known. Local linkage disequilibrium (LD) in the DGRP is low and thus favorable for identifying candidate genes and even causal polymorphisms [14,15]. We identified single nucleotide polymorphisms (SNPs) affecting three genes previously known to contribute to variation in abdominal pigmentation, *bric-à-brac 1* (*bab1*), *tan* (*t*), and *ebony* (*e*). However, we also identified novel candidate genes and showed that these contribute to abdominal pigmentation using mutations and RNAi knock-down constructs. Many of these novel genes affect other well-studied pathways and phenotypes, such as wing and bristle development, providing evidence for widespread pleiotropy. Four of the novel genes affecting pigmentation are computationally predicted genes with previously unknown functions. Based on their mutant or RNAi knockdown phenotypes, we have named them *pinstripe* (*pns*, *CG7852*), *triforce* (*tfc*, *CG9134*), *plush* (*ph*, *CG1887*), and *farmer* (*frm*, *CG10625*).

## Results

### Quantitative genetics of pigmentation

**Fig 1:**
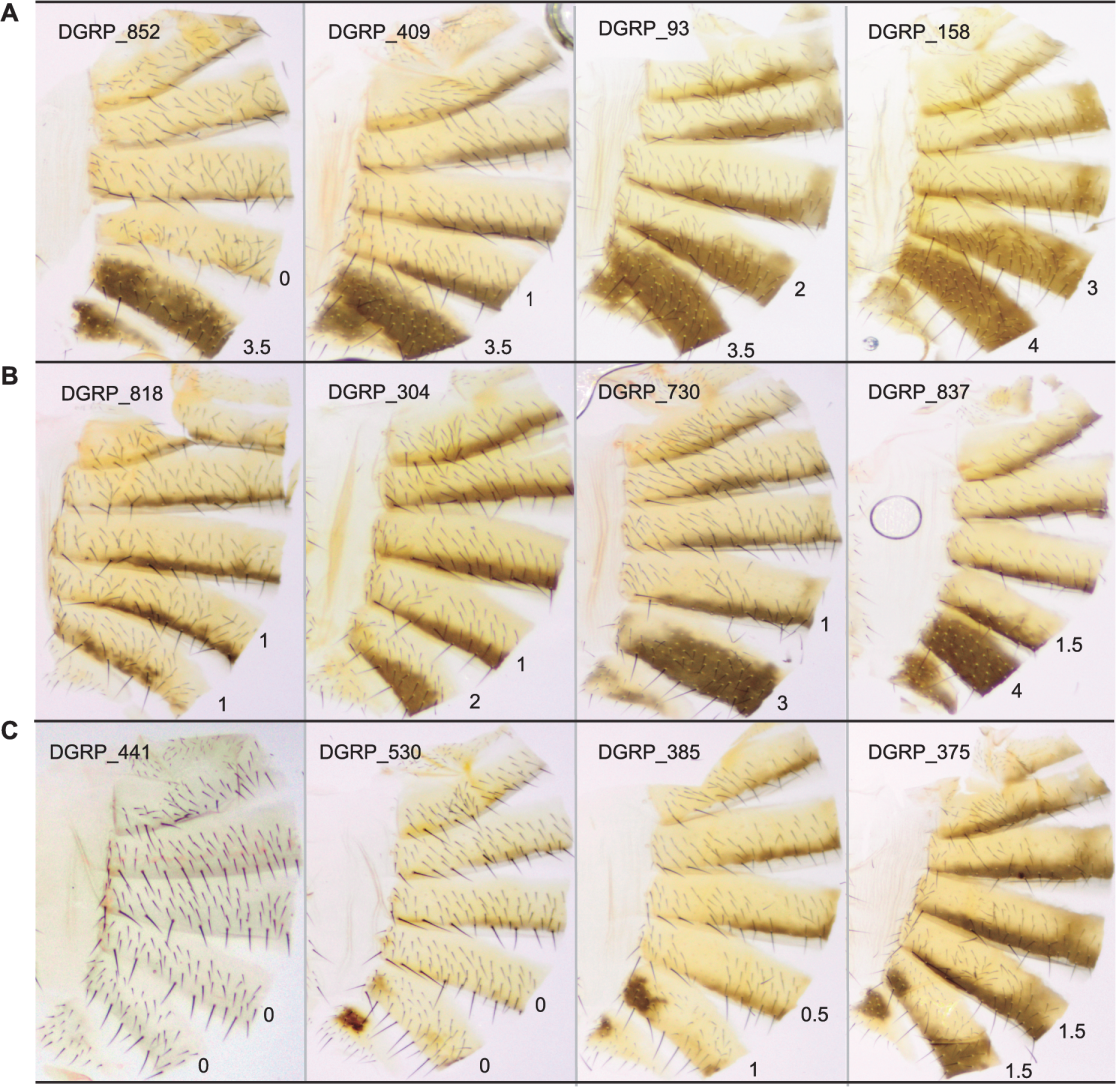
Natural variation in T5 and T6 pigmentation in the DGRP. Images display one half of the fly abdominal cuticle, split along the dorsal midline. Pigmentation scores are given for T5 (upper) and T6 (lower). DGRP lines are denoted by DGRP_XXX in the upper left corner of each image. (**A**) Variation in T5. (**B**) Variation in T6. (**C**) Variation in spatial patterning of pigmentation.

**Fig 2:**
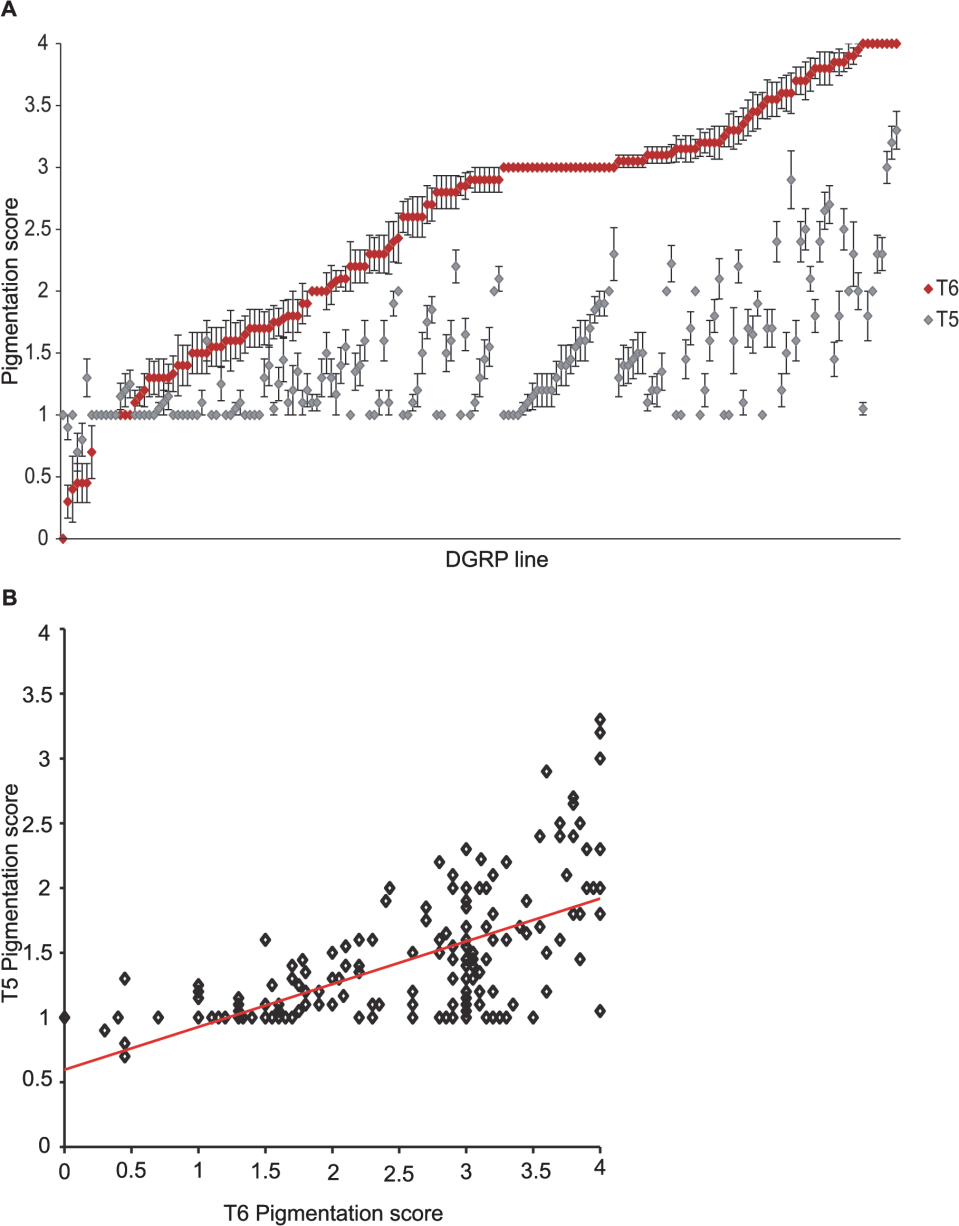
Natural variation in female abdominal pigmentation. (**A**) T5 (gray) and T6 (red). DGRP lines are in order from least to most pigmentation on T6. (**B**) Scatter plot of T5 and T6 line means.

We characterized natural variation in the proportion of melanization of tergites 5 (T5) and 6 (T6) in females for 175 DGRP lines (Figs 1 and 2 and S1 Table). Averaged across all lines, the mean pigmentation scores are 1.44 for T5 and 2.55 for T6 (Fig 2A). There is significant genetic variation in pigmentation among lines for both tergites (*P*_*T5*_ = 4.68 x 10^–48^ and *P*_*T6*_ = 6.65 x 10^–96^; S2 Table), with broad sense heritabilities (*H* ^2^) of *H* ^2^_T5_ = 0.66 and *H* ^2^_T6_ = 0.88. The phenotypic (*r*_*P*(T5,T6)_ = 0.63 ± 0.059) and genetic (*r*_*G*(T5,T6)_ = 0.72 ± 0.053) correlations (± standard error) between the tergites for proportion of pigmentation are high but significantly different from unity, suggesting they have different genetic bases (Fig 2B). The high broad sense heritabilities for abdominal pigmentation traits provide a favorable scenario for GWA studies.

### Genome-wide association analyses

**Fig 3:**
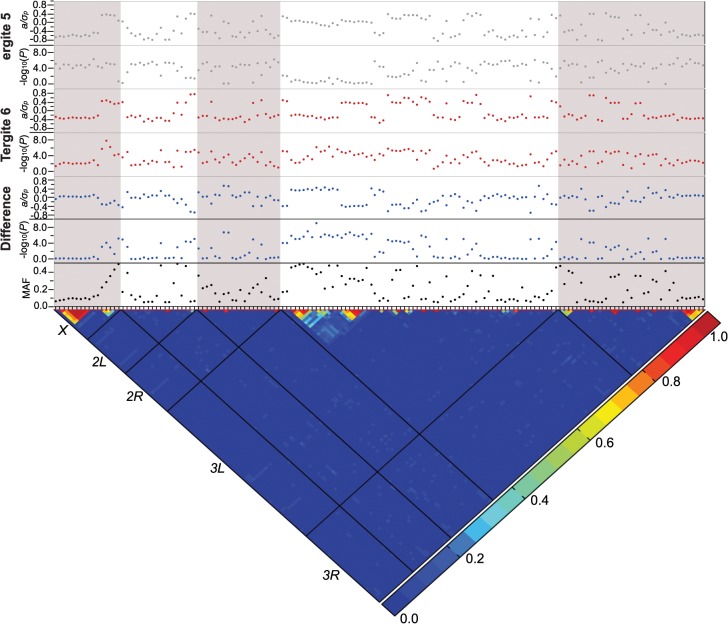
Genome-wide association analyses. Results are depicted for T5, T6, and the T5-T6 difference. A nominal *P* ≤ 10^–5^ is indicated with a red line for each trait. The triangular heat map depicts the degree of LD, *r* ^2^, between variants. The five major chromosome arms are delineated by the black lines. Red corresponds to complete LD and blue to absence of LD. The upper panels show the mixed model significance threshold (-log10(*P*)) and the effect size in phenotypic standard deviation units (*a*/*σp*) for each trait. The minor allele frequency (MAF) is shown on the bottom panel.

We performed genome-wide association analyses on the proportion of T5 and T6 melanization to identify genomic regions harboring variants contributing to natural variation in female abdominal pigmentation. The DGRP lines vary in *Wolbachia* infection status and karyotype for several common polymorphic inversions. We did not find significant associations of *Wolbachia* infection (*P*_*T5*_ = 0.58 and *P*_*T6*_ = 0.92) nor inversion karyotype on T5 or T6 pigmentation; however, the difference in pigmentation between T5 and T6 was significantly affected by *ln*(*2L*)*t* (*P* = 0.04) and *In*(*2R*)*NS* (*P* = 0.01) (S3 Table). For each GWA analysis, we used both a mixed model that accounted for any effects of *Wolbachia*, inversions, and cryptic relatedness and a regression model that corrected for all of the aforementioned effects except for cryptic relatedness [15]. Combining all of these models, we identified a total of 155 variants associated with pigmentation for any trait at a nominal reporting threshold of *P* < 10^–5^ (S4 Table). Of these, 84 were associated with T5 pigmentation, 34 with T6 pigmentation, 28 with the average of T5 and T6, and 35 with the difference in pigmentation between T5 and T6. A total of 84 candidate genes were implicated by these associated variants. Since variants associated with the average of the two posterior tergites were largely the same as those associated with either T5 or T6 alone, we focus our subsequent analyses on T5, T6 and the difference between them (Fig 3 and S4 and S5 Tables). Among the genes harboring SNPs associated with variation in abdominal pigmentation, we find genes with well documented effects on pigmentation (*t*, *e*, *bab1*); *osa*, a transcription factor recently shown to affect pigmentation; and a large group of novel candidate genes [2,9,16]. The identification of *t*, *e*, and *bab1* as prominent contributors to variation in abdominal pigmentation instills confidence in the efficacy of our GWA analyses, as described below.

Only a few variants exceeded a strict Bonferroni correction for multiple tests (*P* = 2.64 × 10^–8^): a SNP 41 bp upstream of *Gr8a* and 528 bp downstream of *CG15370*—the *cis*-regulatory region of *t*—in the T6 and average of T5 and T6 analyses (*X*_9121129_SNP); and two SNPs in the first intron of *bab1* in the analysis of the difference between T5 and T6 (*3L*_1084990_SNP and *3L*_1084199_SNP; S4 Table). The three SNPs that achieved Bonferroni significance levels were all at intermediate frequency and had moderately large effects. The minor allele of the polymorphism in the *t cis*-regulatory element (CRE) was associated with reduced pigmentation, while the minor alleles of the *bab1* intronic polymorphisms were both associated with increased pigmentation in T6.

Although the other variants do not reach individual Bonferroni-corrected significance levels, quantile-quantile plots (S1 Fig) indicate a systematic departure from random expectation below *P* < 10^–5^, justifying our choice of this reporting threshold and suggesting that the top associations are enriched for true positives. Indeed, the SNP in the *t* CRE that reached Bonferroni significance in the T6 analysis was also significant in the T5 analysis at the more lenient reporting threshold, and two additional polymorphisms in the *t* CRE were significant at *P* < 10^–5^: *X*_9121177_SNP in the T5 and T6 analyses, and *X*_9121094_SNP in the T6 analysis.

**Fig 4:**
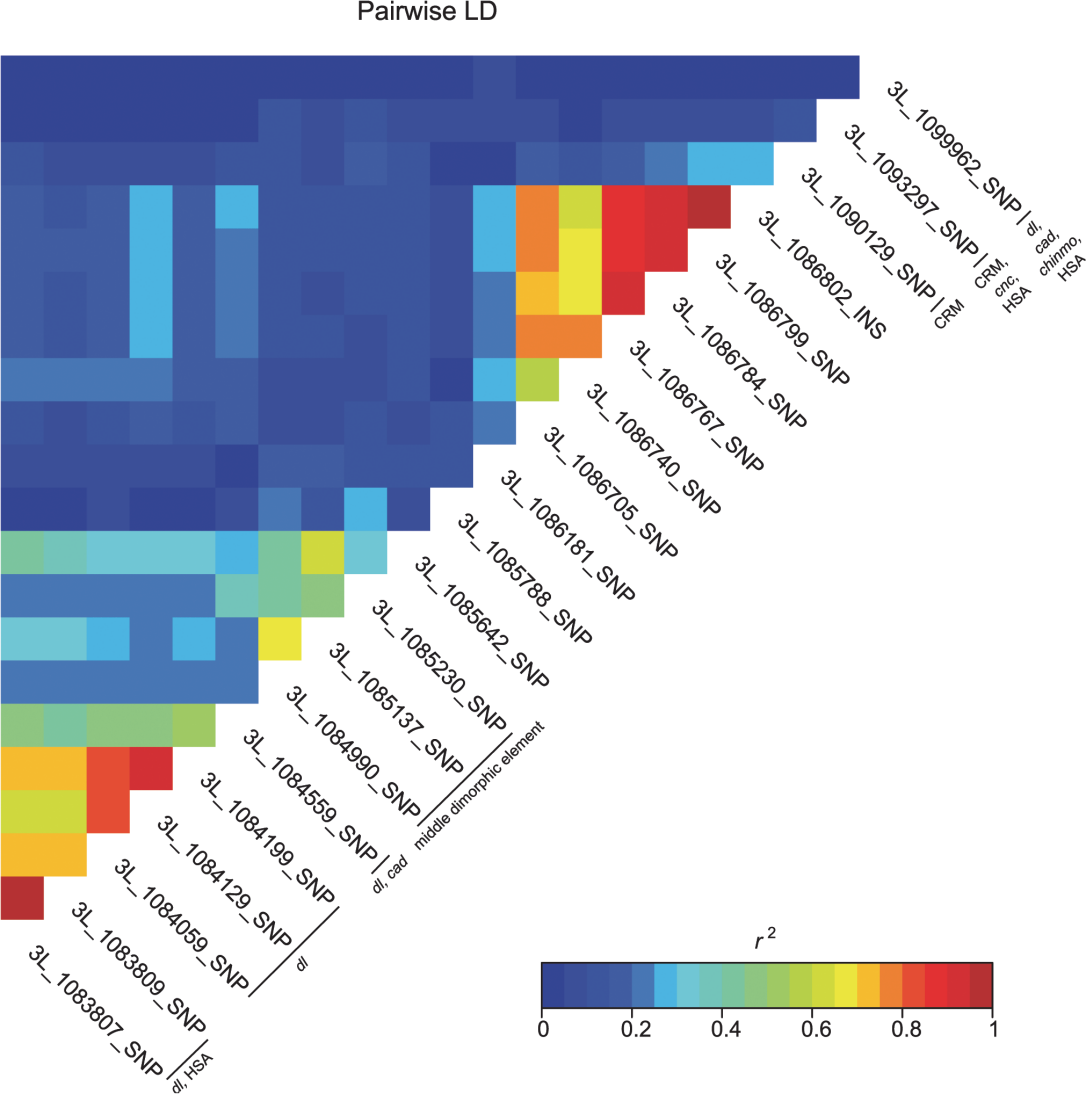
Pairwise linkage disequilibrium among the associated *bab1* variants. Heat map of LD (*r* ^2^) of the 21 *bab1* variants. Individual variants, *cis*-regulatory regions and transcription factor binding sites are labeled on the diagonal.

These data also highlight the importance of *bab1* with respect to female abdominal pigmentation: we found a total of 21 polymorphisms (20 SNPs, one indel) in the first intron of this gene that are associated with natural variation in pigmentation in one or more analyses (Fig 4 and S4 Table). One *bab1* SNP is unique to the T5 analysis, six *bab1* SNPs are common between the T6 and T5—T6 difference analysis, and the remaining *bab1* variants are unique to the difference in pigmentation between T5 and T6. Twelve of the *bab1* variants are located within the minimal functional *cis*-regulatory regions as reported by REDfly or within other transcription factor binding sites (S4 and S6 Tables) [17]. Three SNPs (*3L*_1084990_SNP, *3L*_1085137_SNP, and *3L*_1085230_SNP) are located in the *bab1* middle dimorphic element which contain binding sites for the transcription factors *caudal* (*cad*) and *dl* (*dorsal*) [3]. All of the polymorphisms segregating in *bab1* associated with pigmentation have minor allele frequencies ranging from 0.22 to 0.49 and moderate effects. Interestingly, the direction of the effects is both positive (the minor allele is associated with reduced pigmentation) and negative (the minor allele is associated with increased pigmentation), such that variants in the *dl* and *cad cis*-regulatory modules have positive effects while those in the latter regions of the intron have largely negative effects (the exceptions are *3L*_1093297_SNP in the latter region of the intron and *3L*_1099962_SNP in the T5 GWAS, which have positive effects). The functionality of these *bab1* CREs has been thoroughly investigated [3]. However, similar to the results of Bickel *et al*. [18], nine of the *bab1* variants from this study are in regions outside of the known *cis*-regulatory regions. These variants may indicate the presence of a not-yet-described regulatory element, or the structure of the regulatory elements in this region may be more complex than previously thought (Fig 4).

A majority of the variants associated with variation in pigmentation are located within intronic or intergenic regions, suggesting they could affect gene regulation. In support of this hypothesis, we found many of these variants are located in annotated regulatory sites (S4 and S6 Tables). In total, variants associated with pigmentation were located in 24 different transcription factor binding sites (TFBS, each of which contain numerous variants), 17 *cis*-regulatory modules (CRM), 1 *polycomb* response element (PRE), and 31 hot spot analysis sites (HSA; where one or more 41 tested TFs bind to a given site) (S6 Table). TFBS for *dl* and *cad* are the most frequent of all TFBS, containing 28 and 22 associated variants, respectively. Two intergenic TFBS for *bab1* (FBsf0000214860 and FBsf0000214320) were tagged by *3R*_25139342_SNP, *3R*_25139132_SNP, and *2R*_16793853_SNP. A few variants are located in more than one regulatory site (S4 Table).

### Variance in pigmentation explained by top variants

We asked what fraction of the total broad sense heritability was explained by variants in *bab1*, *t* and *e* using stepwise regression to select the top associations for pigmentation genes. The *R* ^2^ from these models for each trait gives the heritability explained by the known genes. These loci explain 25.62%, 37.55%, 31.17% and 36.58% of the heritability for T5, T6, and the average and difference of T5 and T6, respectively; consistent with the intermediate allele frequencies and large effects of their top associated variants. Next, we used genomic best linear unbiased prediction (GBLUP) to estimate the total variance explained by all top variants. All variants explain 59.77%, 34.32%, 47.44% and 51.61% of the heritability for T5, T6, and the average and difference of T5 and T6, respectively. With the exception of T6, for which most of the variance is explained by the known pigmentation genes, substantial additional variance in proportion of pigmented cuticle is contributed by variants in novel genes. Finally, we estimated the faction of heritability explained for each variant as well as the fraction of heritability explained after accounting for the variance explained by the pigmentation genes. On average, the variants in novel candidate genes explained an additional 7.3% (T5), 4.5% (T6), 5.8% (average of T5 and T6) and 2.8% (difference between T5 and T6) of the heritability (S2 Fig and S4 Table)

### Comparison with previous studies

*e*, *t*, and *bab1* have been associated with variation in *D*. *melanogaster* female pigmentation in other populations [2,9,16]. We compared the top variants in these genes in our analyses with those from prior studies [9,16,19]. Three of the four SNPs identified by Bastide *et al*. [9] are identical to the three *t* CRE SNPs associated with our T5 and T6 analyses (*X*_9121094_SNP, *X*_9121129_SNP, and *X*_9121177_SNP). The *bab1* SNP identified in the Bastide *et al*. [9] study did not overlap with our results nor those of Bickel *et al*. [18]. None of the top *bab1* variants in this study were significant in the study of Bickel *et al*. [18], although three of our significant variants were also polymorphic in the Bickel data set (*3L*_1085788_SNP, *3L*_1086799_SNP, and *3L*_1086802_INS). Both the *e* CRE SNPs associated with pigmentation in the DGRP T6 analysis (*3R*_17063120_SNP) and the Bastide *et al*. study [9] (*3R*_17064232_SNP) are located within the CRE regulating *e* expression in the haltere (*e*_C [19]; S4 Table). The SNP at *3R*_17064232 was also reported in the Pool and Aquadro study of light and dark African *D*. *melanogaster* [16]. The concordance among these datasets indicates that the haltere regulatory element may also control expression in the abdomen and warrants further investigation.

### Validation of candidate genes

We selected 30 novel candidate genes based on the GWA results for functional validation using mutant alleles and RNAi knockdown (S7 Table). We phenotyped Exelixis insertion lines [20] and RNAi knockdown lines [21] with their appropriate controls for the proportion of melanization on T5 and T6 as done for the DGRP (S8 and S9 Tables). Wherever possible we tested both mutant and RNAi lines for the same gene as independent forms of validation. We used three *GAL4* drivers for the *UAS-RNAi* lines. *tubulin-GAL4/TM3*, *Sb (tub-GAL4)* and *ubiquitin-GAL4/CyO (ubi-GAL4)* are ubiquitously expressed, while the pannier driver, *y* ^*1*^ *w* ^*1118*^ *; P{w[+mW*.*hs] = GawB}pnr* ^*MD237*^ */TM3*, *P{w[+mC] = UAS-y*.*C}MC2*, *Ser* ^*1*^ *(pnr-GAL4)*, has restricted expression in the midline [22]. The use of the *pnr-GAL4* driver adds a spatial component to the validation experiments and allows for more precise testing of the candidate genes (S3 Fig). As positive controls, we also tested RNAi constructs for e and t (S3 Fig and S8 and S9 Tables).

**Fig 5:**
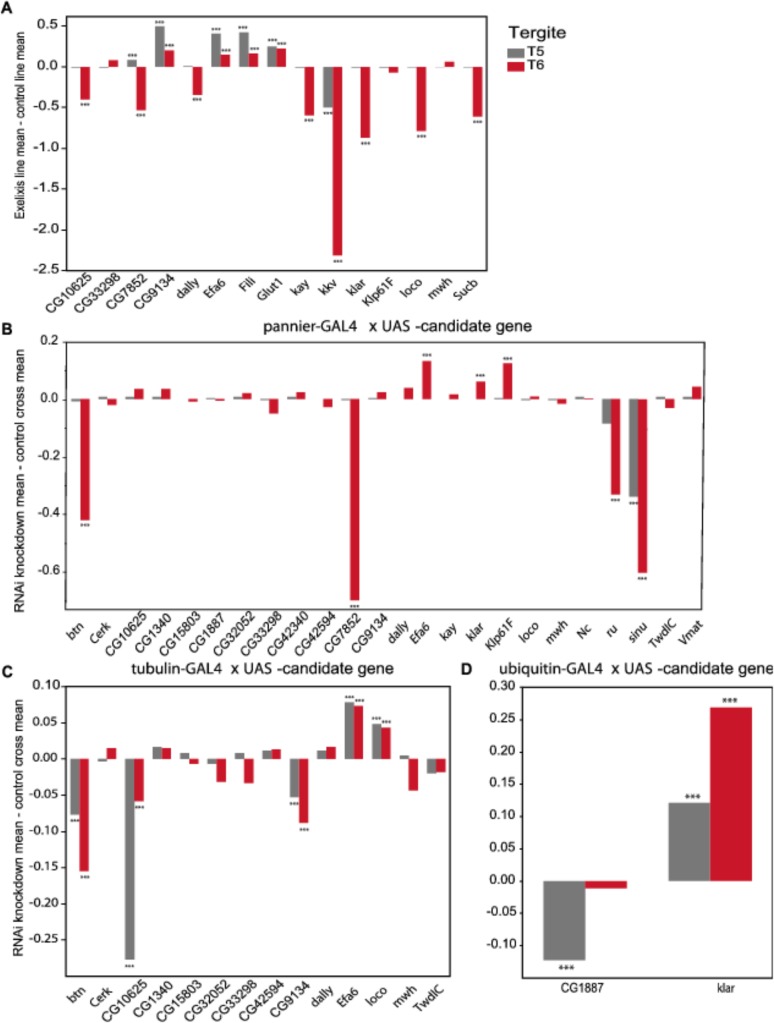
Validation of pigmentation candidate genes using Exelixis insertion mutants and RNAi knockdown. The *y*-axis in all panels is the deviation of the appropriate control line mean from the experimental line mean. Increases and decreases in the proportion of melanization are given above and below the *x*-axis, respectively. (**A**) Exelixis insertion mutants. (**B**) *pnr-GAL4* x RNAi-*UAS* lines. (**C**) *tub*-*GAL4* x RNAi-*UAS* lines. (**D**) *ubi*-*GAL4* x RNAi-*UAS*-lines. ***: *P*<0.0001.

**Fig 6:**
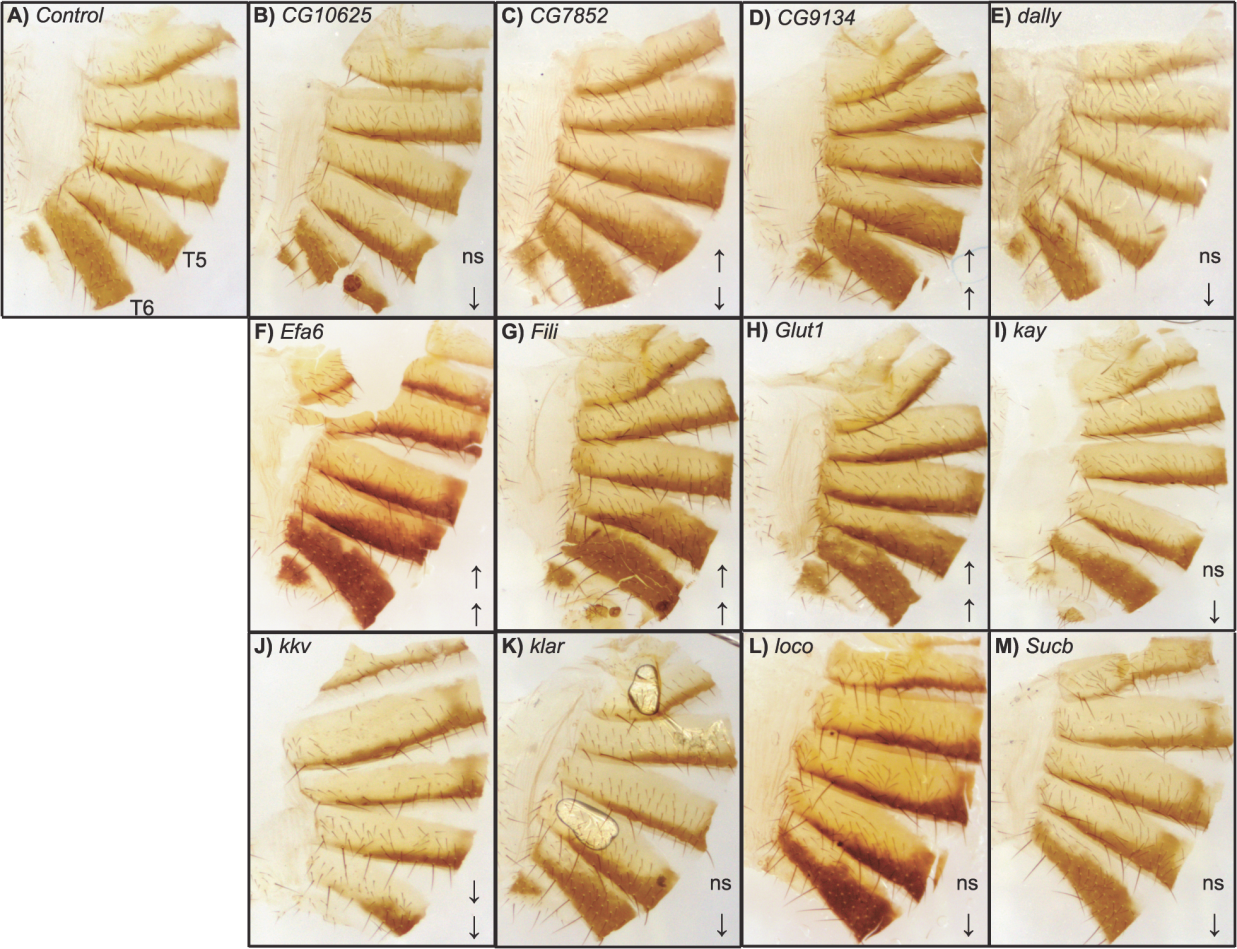
Female abdominal cuticles of Exelixis transposon insertion lines with significant effects on pigmentation. (**A**) Exelixis *w^1118^* control. (**B**) *CG10625^e01211^*. (**C**) *CG7852^c04511^*. (**D**) *CG9134^e00088^*. (**E**) *dally^f01097^*. (**F**) *Efa6^f03476^*. (**G**) *Fili^f04573^*. (**H**) *Glut1^d05758^*. (**I**) *kay^f02002^*. (**J**) *kkv^c06225^*. (**K**) *klar^d05910^*. (**L**) *loco^d09879^*. (**M**) *Sucb^e01940^*. ns = not significant. ↑ and ↓ indicate significantly increased and decreased proportions of dark melanin, respectively. T5 = tergite 5 and T6 = tergite 6.

We evaluated 15 Exelixis transposon insertions in candidate genes for effects on pigmentation (See Methods; Fig 5A and S7 Table). Six of these mutations affected the proportion of melanization on T5 (*P* < 0.0001 for all significant mutations): *CG9134* ^*e00088*^, *CG7852* ^*c04511*^, *Exchange factor for arf6* (*Efa6* ^f03476^), *Fish-lips* (*Fili* ^*f04573*^), and *Glucose transporter 1* (*Glut1* ^*d05758*^) showed increased melanization; and *krotzkopf verkehrt* (*kkv* ^*c06225*^) showed decreased melanization (Figs 5A and 6). Twelve of the mutations affected the proportion of melanization on T6 (*P* < 0.0001 for all significant mutations). *CG9134* ^*e0008*^, *Efa6* ^*f03476*^, *Fili* ^*f04573*^, and *Glut1* ^*d05758*^ showed increased melanization; and *CG10625* ^*e01211*^, *CG7852* ^*c04511*^, *division abnormally delayed* (*dally* ^*f01097*^), *kayak* (*kay* ^*f02002*^), *kkv* ^*c06225*^, *klarsicht* (*klar* ^*d05910*^), *locomotion defects* (*loco* ^*d09879*^), and *Sucb* ^*e01940*^ showed decreased melanization (Figs 5A and 6). *CG7852* ^*c04511*^ had increased pigmentation on T5 and decreased pigmentation on T6 (Fig 6C). *CG33298* ^*d10678a*^, *multiple wing hairs* (*mwh* ^*d01620*^), and *Kinesin-like protein at 61F* (*Klp61F* ^*f02870*^) were not significantly different from the control. *Efa6* ^*f03476*^ also has a light and somewhat elongated thoracic trident; this thoracic pigmentation is completely absent in the control flies (S4 Fig).

**Fig 7:**
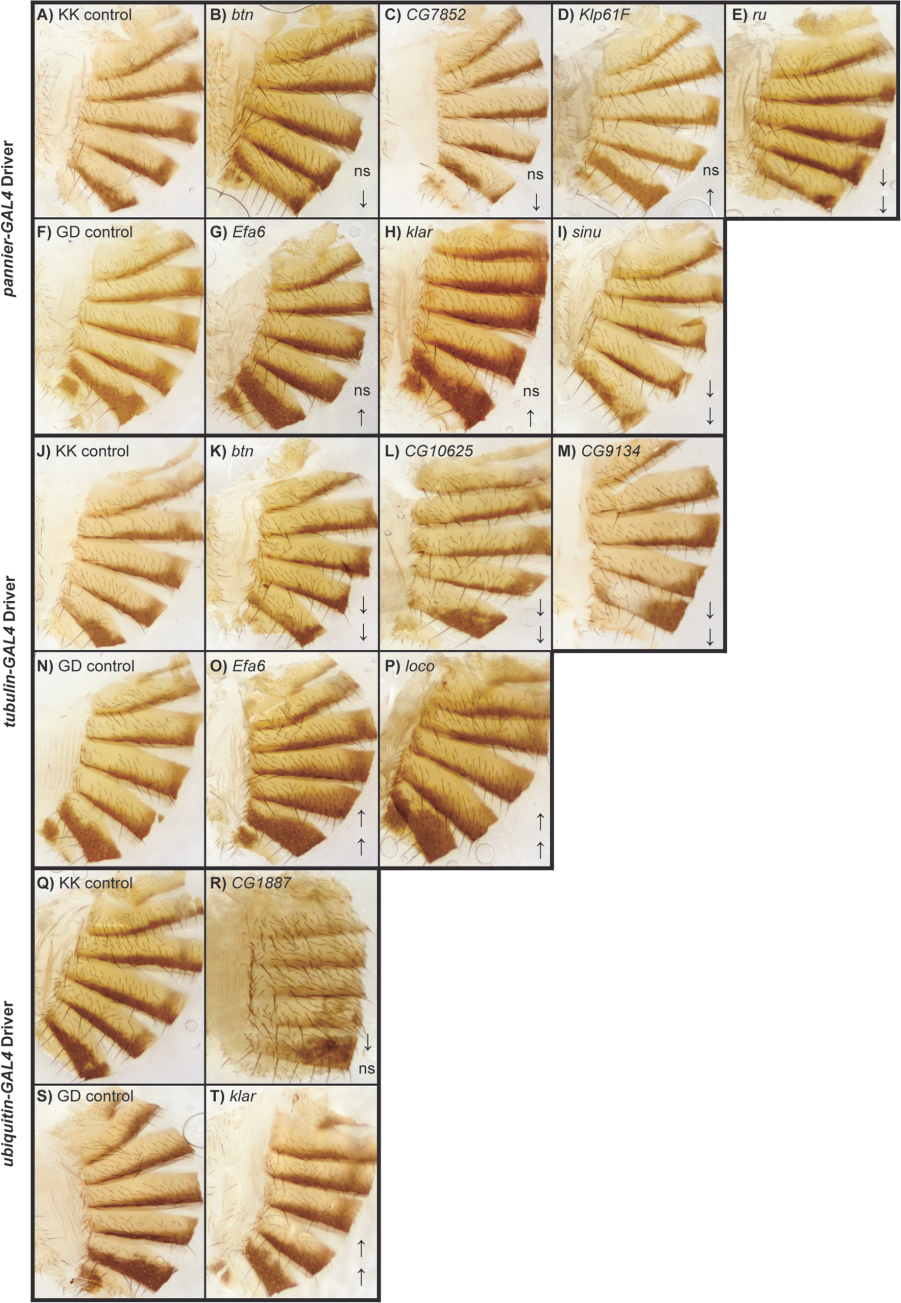
Female abdominal cuticles of with significant effects on pigmentation in lines with targeted RNAi knockdown constructs. **A**-**J**: *pnr-GAL4* x *UAS-RNAi* genotypes. (**A**) VDRC KK library control. (**B**) *btn*. (**C**) *CG7852*. (**D**) *Klp61F*. (**E**) *ru*. (**F**) VDRC GD library control. (**G**) *Efa6*. (**H**) *klar*. (**I**) *sinu*. **J**-**Q**: *tub*-*GAL4* x *UAS-RNAi* genotypes. (**J**) VDRC KK library control. (**K**) *btn*, (**L**) *CG10625*. (**M**) *CG9134*. (**N**) VDRC GD library control. (**O**) *Efa6*. (**P**) *loco*. **Q**-**T**: *ubi*-*GAL4* x RNAi-*UAS* genotypes. (**Q**) VDRC KK library control. (**R**) *CG1887*. (**S**) VDRC GD control. (**T**) *klar*. ns = not significant. ↑ and ↓ indicate significantly increased and decreased proportions of dark melanin, respectively.

Of the 28 candidate genes, 26 were available as RNAi knockdown constructs (S7 and S8 Tables). We crossed all of these constructs to the *pnr*-*GAL4* driver, and obtained viable female progeny from all crosses except for *kkv* and *Fili*. We found that seven of these knockdown mutations affected the proportion of melanization of T5 and/or T6 (Figs 5B and 7A–7I, *P* < 0.0001 in all cases). *Efa6*, *klar*, and *Klp61F* knockdowns increased the proportion of dark melanin on T6; *buttonless* (*btn*) and *CG7852* decreased it. Knockdown of *roughoid* (*ru*) and *sinuous* (*sinu*) showed decreases in pigmentation for both T5 and T6.

We also crossed the 26 *UAS*-RNAi constructs to an ubiquitously expressed *tub*-*GAL4* driver, and found that 11 (42%) were lethal in both sexes, suggesting pleiotropic effects on vital functions: *Vesicular monoamine transporter* (*Vmat*), *Klp61F*, *CG7852*, *CG1887*, *klar*, *ru*, *sinu*, *Nedd2-like caspase* (*Nc*), *kkv*, *kay*, and *CG42340* (Table 1). Of the 15 *UAS*-RNAi/*tub*-*GAL4* knockdown alleles available for testing, six had significant (*P* < 0.0001) effects on pigmentation. Knockdowns of *btn*, *CG10625*, and *CG9134* had decreased proportions of dark melanin on T5 and T6; *Efa6* and *loco* knockdowns showed increases in pigmentation on both tergites (Figs 5C and 7).

**Table 1:** Summary of candidate gene validation experiments.

			RNAi Driver		
Candidate Gene	GWAS Association	Exelixis (T5/T6)	*tub*-*GAL4* (T5/T6)	*pnr*-*GAL4* (T5/T6)	*ubi*-*GAL4* (T5/T6)
*CG33298*	T6	NS/NS	NS/NS	NS/NS	-
*Fish-lips* (*Fili*)	T6	↑/↑	lethal (♀ only)	lethal (♀ only)	-
*Vesicular monoamine transporter* (*Vmat*)	T6	-	lethal	NS/NS	lethal
*multiple wing hairs* (*mwh*)	T6	NS/NS	NS/NS	NS/NS	-
*Kinesin-like protein at 61F* (*Klp61F*)	T6	NS/NS	lethal	NS/↑	lethal
*CG9134*	T6	↑/↑	↓/↓	NS/NS	-
*CG7852*	T6, T5-T6	↑/↓	lethal	NS/↓	lethal
*CG1887*	T6	-	lethal	NS/NS	↓/NS
*klarsicht* (*klar*)	T6	NS/↓	lethal	NS/↑	↑/↑
*Glucose transporter 1* (*Glut1*)	T6	↑/↑	-	-	-
*Exchange factor for Arf 6* (*Efa6*)	T6	↑/↑	↑/↑	NS/↑	-
*buttonless* (*btn*)	T6	-	↓/↓	NS/↓	-
*roughoid* (*ru*)	T5	-	lethal	↓/↓	lethal
*CG10625*	T5	NS/↓	↓/↓	NS/NS	-
*sinuous* (*sinu*)	T5	-	lethal	↓/↓	lethal
*Sucb* (*Sucb*)	T5	NS/↓	-	-	-
*division abnormally delayed* (*dally*)	T5	NS/↓	NS/NS	NS/NS	-
*CG32052*	T5	-	NS/NS	NS/NS	-
*Nedd2-like caspase* (*Nc*)	T5	-	lethal	NS/NS	lethal
*Ceramide kinase* (*Cerk*)	T5	-	NS/NS	NS/NS	-
*krotzkopf verkehrt* (*kkv*)	T5	↓/↓	lethal	lethal	lethal
*CG15803*	T5	-	NS/NS	NS/NS	-
*locomotion defects* (*loco*)	T5	NS/↓	↑/↑	NS/NS	-
*TweedleC* (*TwdlC*)	T5	-	NS/NS	NS/NS	
*kayak* (*kay*)	T5	NS/↓	lethal	NS/NS	lethal
*CG1340*	T5	-	NS/NS	NS/NS	-
*CG42594*	T5	-	NS/NS	NS/NS	-
*CG42340*	T5	-	lethal	NS/NS	lethal

NS = not significant,"–" = line not available or RNAi cross not tested. ↑ = increased pigmentation. ↓ = decreased pigmentation.

Next, we crossed the 11 *UAS*-RNAi constructs that were lethal when crossed to the *tub*-*GAL4* driver to another ubiquitously expressed *GAL4* driver, *ubi*-*GAL4*. Only two RNAi constructs were viable when driven by *ubi*-*GAL4*, *CG1887* and *klar*, and both had significant (*P* < 0.0001) effects on abdominal pigmentation (Figs 5D and 7 and Table 1). The *CG1887* knockdown showed a decrease in T5 melanization. Although T6 did not show a significant difference in the proportion of melanin in the *CG1887* knockdown (*P* = 0.71), the dark melanin that is present is a light brown melanin that is only slightly darker than the adjacent sclerotin (S5 Fig). The *CG1887* knockdown flies have obvious qualitative differences in overall body coloration from the control. The cuticle as a whole is semi-transparent and its strength is compromised as it ruptures easily when probed with forceps. The third thoracic leg of these progeny is also malformed and bristle number and patterning is highly disrupted (S5 Fig). All progeny of the cross die within 24 hours of eclosion; thus, pigmentation scoring was performed 8 hours after eclosion. The *klar* knockdown shows an increase of melanization on both tergites. Similar to the *Efa6* mutant, this cross leads to a relative darkening of the thoracic trident compared to the surrounding cuticle (S4 Fig). In summary, we found that 17 of the 28 candidate genes tested affected female abdominal pigmentation and that for 12 of these genes, both tergites are affected (Table 1 and S8 and S9 Tables).

## Discussion

The DGRP lines show substantial natural variation in female abdominal pigmentation, ranging from lines with no dark melanin to complete melanization on tergites 5 and 6. Despite being sampled from a single population, the lines span the range of pigmentation difference between the well-studied sister species *D*. *yakuba* and *D*. *santomea*. *D*. *santomea* is the lightest member of the *D*. *melanogaster* species subgroup; however, several of the DGRP lines are lighter than *D*. *santomea* (e.g., DGRP_441, Fig 1C). Utilizing the substantial genetic variation and a mapping population that is powerful to detect common variants associated with the variation, especially those with moderate to large effects (S6 Fig), we identified a total of 155 genetic variants associated with variation in female abdominal pigmentation using GWA analyses.

We identified variants in four genes previously shown to affect adult *D*. *melanogaster* pigmentation: *bab1*, *t*, *e*, and *osa* [2,16,23,24]. Most of the *bab1* SNPs were associated only with the difference between T5 and T6 pigmentation, suggesting that variation in *bab1* may underlie the genetic and phenotypic correlations between the traits. Most of the *bab1* and *t* minor alleles are at moderate frequencies in the DGRP. These SNPs could be neutral or could be maintained segregating by a balance of unknown selective forces. We identified three SNPs in the CRE of *t* that were also found in the European populations studied by Bastide *et al*. [9], indicating that these SNPs are maintained in distant populations. Of note, our study is the first to associate natural variation in pigmentation with genetic variation in the transcription factor *osa*.

A majority of the variants identified in this study are in intronic or intergenic regions. Among the total regulatory elements identified, *dl* and *cad* binding sites were the most highly represented, suggesting a role for these two TFs on pigmentation patterning. Over half of the genetic variants located within *bab1* were in known regulatory regions including some for *dl* and *cad* binding. We also identified a SNP upstream of *e* that is located within a *cis*-regulatory region consistent with the studies of African and European *D*. *melanogaster* [9,16,19]. These results implicate *cis*-regulatory evolution, which likely limits negative pleiotropic effects, as a major contributor to phenotypic variation within the DGRP population.

In addition to genes previously known to affect *Drosophila* pigmentation, we identified many novel candidate genes. We showed that 61% of the candidate genes affect the proportion of dark pigmentation on tergites 5 and 6 using mutant and RNAi knockdown experiments. These genes are known to be involved in processes such as sugar binding and transport, vesicle formation and transport, and cuticle formation. We summarize what is known from the literature about these candidate genes below and speculate about their roles in pigmentation and phenotypic evolution.

Prior to molting and eclosion, insects accumulate tyrosine-derivatives conjugated with hydrophilic molecules such as glucose, phosophate, and sulfate in the hemolymph. This keeps the reactive pigment precursors in an inert state until the organism is ready to molt or eclose [13,25–29]. We identified two genes, *triforce* (*tfc*, *CG9134*) and *Glut1*, which may facilitate the transport of glucose or glucose conjugates from the hemolymph to the epidermal cells. *tfc* is a C-type lectin with a carbohydrate binding domain and *Glut1* is a membrane bound glucose transporter [30].

Several of our new pigmentation genes have roles in relatively well-described developmental pathways. These include *kay*, *dally*, *Fili*, and *ru*. *kay* is a transcription factor in the JNK signal transduction pathway [31]. It is required for *decapentaplegic* (*dpp*) expression, wing and leg development, and the elongation of the cells in the epidermis [32]. *dpp* expression in the tergite corresponds to the midline pigment stripe, and ectopic expression of *dpp* in pupae expands this stripe [32]. Furthermore, *dpp* signal transduction is potentiated by *dally* [33]. Additionally, *dpp* and *Epidermal growth factor receptor* (*Egfr*) signaling work synergistically to specify the lateral tergite cell fate [32. *ru*, also known as *Rhomboid-3* (*Rho*-*3*), activates *Egfr* signaling and thus may help determine epidermal cell fate in the developing tergites; however, there are several Rho proteins capable of this activation [34]. *Fili* is a transmembrane protein that is involved with apoptosis in the wing imaginal disc and we speculate may facilitate proper tergite differentiation during metamorphosis [35].

Four validated candidate genes are involved with vesicle formation and transport: *pinstripe* (*pns*, *CG7852*, which describes the vertical stripe of pigment remaining on T6 in the RNAi knockdown), *Efa6*, *Klp61F*, and *klar*. *pns* is predicted to have Rab guanyl-nucleotide exchange factor activity, which facilitates vesicle transport by activating Rab proteins [36,37]. Rab5 works in conjunction with Megalin to remove the Yellow protein from the tanning *D*. *melanogaster* wing [38]. *Efa6* is also involved with Rab signaling and vesicle mediated transport [36]. *Klp61F* is a kinesin motor, and *klarsicht* (*klar*) regulates microtubule-dependent vesicle transport along microtubules. Both could be involved in transporting vesicles of enzymes and/or dopamine-derivatives to and from the cuticle. Together these genes may represent components of the little-known transport mechanism for cuticle tanning in *D*. *melanogaster*.

*farmer* (*frm*, *CG10625*—the not quite fully developed stripes of pigment on tergites 5 and 6 are similar to the tan lines on the arms of a farmer after much time spent in the sun), *kkv*, and *sinu*, and *loco* may affect cuticle development and structure. *frm* is a cuticle structural protein [36]. *kkv* is one of two chitin synthase genes in *D*. *melanogaster* [39]. *sinu* is a claudin required for septate junction organization, cell-cell adhesion, and proper localization of proteins involved in chitin filament assembly in *D*. *melanogaster* [40]. *loco* regulates G protein signaling, and Gγ1 signaling is required for proper septate junction protein localization including *sinu* [41,42]. These proteins may help maintain the structural integrity of the adult cuticle and our study shows that when perturbed, they affect pigmentation.

*Sucb* and *ph* may affect the organism more broadly. *Sucb* is a succinate-CoA synthetase in the Krebs cycle [36]. It is plausible that variation in energy production due to genetic variation in key enzymes could indirectly affect variation in adult pigmentation by altering resources available for cuticle development. *ph* is a CD36 homologue, and dipteran CD36 family members may have roles in transport of cholesterol and steroids during ecdysone synthesis [43]. Since ecdysone is required for proper insect development and molting, disrupted transport of ecdysone precursors may explain the severe RNAi phenotype for this gene.

In summary, we provide evidence that genetic variation at a number of steps in regulatory, developmental, and transport may pathways contribute to natural variation in abdominal pigmentation. These findings exemplify the pleiotropic nature of these genes which may limit their potential as adaptive targets [44–46]. Several of the mutant and RNAi knockdown lines were lethal or had strong debilitating effects providing some support of this. It is known that the large-effect genes, *t* and *e*, are pleiotropic as well [23]. The difference may be that not all pigmentation genes have the necessary regulatory modules to alleviate pleiotropic effects. However, these candidate genes may contain tissue- or stage-specific gene regulatory architectures since most of the GWAS associated SNPs are in intronic and intergenic regions. Furthermore, a distinction should be made between pleiotropic genes and pleiotropic variants. A given gene may be pleiotropic, while particular variants within that gene may not be [47]. Additionally, in the DGRP lines allelic variants are homozygous. In nature, alleles with detrimental effects may be tempered in the heterozygous state or epistatic interactions may arise with differing combinations of polymorphic loci.

Consistent with other DGRP studies, we identified many more genetic variants associated with pigmentation than previous studies [48–51]. We suspect earlier studies may have only had the ability to identify the major effect loci and missed the more polygenic variation at these other loci. Most used only a small number of fly lines and thus interrogated a smaller sample of allelic variation, analyzed only known pigmentation genes, or the limited sample size of the mapping population gave reduced statistical power to detect variants with smaller effects. For example, the Winter's Lines, a panel of 144 recombinant inbred lines used to map the effect of *bab1* on pigmentation in female *D*. *melanogaster*, were generated from only two gravid wild caught females [2]. The study of Bastide *et al*. [9] pooled individuals with extreme phenotypes for sequencing. This approach may filter out variants that lead to intermediate phenotypes and select for large effect variants. Pool and Aquadro focused only on *e* sequences among the 25 African lines eliminating any possibility of identifying additional loci [16]. The DGRP is more representative of natural populations and harbors many more polymorphic loci that may contribute to phenotypic variation and evolution [52]. Given both the population sample and the genome-wide coverage of polymorphisms, this study is perhaps the most comprehensive analysis of variation in *Drosophila* pigmentation to date.

It is important to acknowledge that gene expression knockdown and mutant analyses are only an approximate confirmation of causative SNPs. Genes implicated by the GWA analyses that do not confirm in these functional tests may be true positives and contribute to variation in pigmentation, but they do not change pigmentation when gene expression is reduced. Future studies should test effects of individual SNPs and further characterize the mechanisms though which the candidate genes affect variation in pigmentation; their potential interactions with variants in the candidate genes with major effects; and their allele frequency distributions in different populations. These studies will help elucidate the contribution of these novel variants to adaptive phenotypic evolution or whether they are population-specific deleterious variants that are maintained segregating by mutation-selection balance.

Our results open the door for new hypotheses to be tested on the transport of dopamine derivatives and conjugates from the hemolymph to the cuticle, the formation and movement of vesicles within epidermal cells, the mechanisms of regulatory and developmental pathways during tergite differentiation, the interactions of chitin filaments with cell adhesion and cuticle proteins, and how metabolic and hormonal regulation could lead to variation in pigmentation. Genetic variants that affect these processes could potentially serve as targets of adaptive evolution or sexual selection in natural populations. This study is a start. However, much more work is needed to draw mechanistic inferences about these novel candidate genes and their contributions to the evolution of pigmentation.

## Materials and Methods

### *Drosophila* stocks and phenotyping

The DGRP consists of 205 inbred lines with complete genome sequences. We scored female flies of 175 DGRP lines—aged 5 to 8 days—for the proportion of melanization on abdominal tergites 5 and 6. Two independent replicates for each DGRP line were reared and five individuals were scored from each replicate vial (*N* = 10 flies per line). The flies were reared in vials at a controlled adult density (CAD) of 10 males and 10 females on cornmeal-molasses-agar medium at 25°C, 75% relative humidity, and a 12-h light-dark cycle. The parental generation was allowed to lay eggs for 3 days. Each fly was visually assessed by a single observer for the percentage of brown/black melanin covering each tergite; the scores ranged from 0 for no dark pigmentation to 4 for 100% dark pigmentation in increments of 0.5.

### Statistical and quantitative genetic analyses

We partitioned variation in pigmentation into genetic and environmental components using an ANOVA model of form *Y* = *μ* + *L* + *T* + *L*×*T* + *R*(*L*×*T*) + *ε*, where *Y* is phenotype, *μ* is the overall mean, *L* is the random effect of line, *T* is the fixed effect of tergite, *R* is the random effect of replicate vial, and *ε* is the residual. We also performed reduced ANOVAs separately for each tergite of form *Y* = *μ* + *L* + *R* (*L*) + *ε*. We estimated variance components for the random effects using REML. We computed the broad-sense heritability (*H* ^2^) of pigmentation for each tergite separately as *H* ^2^ = *σ* ^2^_L_/ (*σ* ^2^_L_ + *σ* ^2^_ε_), where *σ* ^2^_L_ is the among-line variance component and *σ* ^2^_ε_ is the error variance. We computed the genetic correlation between the tergites (r_T5,T6_) as *Cov*_T5,T6_/ *σ*_LT5_ *σ*_LT6_, where *Cov*_T5,T6_ is the covariance in pigmentation score between tergites 5 and 6. All analyses were performed with version 9.3 of the SAS System for Windows (2013 SAS Institute Inc.).

### Genome-wide association analysis

To identify genomic regions harboring variants contributing to natural variation in the proportion of tergite melanization, we conducted a GWA study for each tergite. The DGRP lines are also segregating for *Wolbachia* infection and for the following common inversions: *In(2L)t*, *In(2R)NS*, *In(3R)P*, *In(3R)K*, and *In(3R)Mo*. We performed GWA studies in two stages. In the first stage, we adjusted the line means for the effects of *Wolbachia* infection and major inversions. We then used the adjusted line means to fit a linear mixed model in the form of *Y =* **X** *b +* **Z** *u + e*, where *Y* is the adjusted phenotypic values, **X** is the design matrix for the fixed SNP effect *b*, **Z** is the incidence matrix for the random polygenic effect *u*, and *e* is the residual. The vector of polygenic effects *u* has a covariance matrix in the form of **A** *σ* ^2^, where *σ* ^2^ is the polygenic variance component. We fitted this linear mixed model using the FastLMM program (version 1.09) [53]. We performed these single marker analyses for the 1,897,337 biallelic variants (SNPs and indels) with minor allele frequencies ≥ 0.05 whose Phred scale quality scores were at least 500 and genotypes whose sequencing depths were at least one and genotype quality scores at least 20 [15]. All segregating sites within lines were treated as missing data. Additionally, we performed single marker tests for association on line means that were adjusted for the effects of *Wolbachia* infection and major inversions but not corrected for the relationship matrix. Significant variants were annotated using the 5.49 Release of the Flybase annotations.

### Variance in pigmentation explained by top variants

For each variant, we calculated two variance components. First, to calculate the variance explained by a variant without adjusting for variants in known pigmentation genes, we fitted a linear model for the adjusted line means for only the focal variant and used the *R* ^*2*^ of the model to represent the variance explained by it. Second, to calculate additional variance explained by a focal variant after accounting for variants in known pigmentation genes, we first used stepwise selection to select the top associations for each pigmentation gene (*tan*, *ebony*, or *bab1*), requiring *P*-values to be smaller than 10^–5^ if more than one variant entered the model, and no *P*-value requirement if there was only one variant. The *R* ^*2*^ of this baseline model (different for each of the four traits) is the variance explained by the pigmentation genes. We added each focal variant to the baseline model and calculated the difference between the *R* ^*2*^ of the new model and the *R* ^*2*^ of the baseline model, which represented the additional variance explained by the variant after accounting for the pigmentation genes. To calculate total variance explained by all significant variants, we used a mixed model approach because of the large number of variants. We computed the variance/covariance matrix based on the genotype matrix and estimated the variance components using the rrBLUP R package.

### Validation of candidate genes

We tested 12 of the 13 genes implicated by the T6 pigmentation GWA analysis, none of which were previously known to affect variation in pigmentation in *D*. *melanogaster*: *CG33298*, *Fili*, *Vmat*, *mwh*, *Klp61F*, *CG9134*, *CG7852*, *CG1887*, *klar*, *Glut1*, *Efa6*, and *btn*. From the T5 pigmentation GWA analysis, we selected candidate genes that (1) had an Exelixis mutant [20] or VDRC RNAi [21] line available at the time of the study; (2) are involved in development, especially of the cuticle or epidermis, or pigmentation according to FlyBase and the available literature; and (3) show mRNA expression patterns similar to the regulatory genes, *bab1* and *Dsx*, and genes in the pigmentation biosynthesis pathway (such as *t* and *e*), a peak of expression at 24 hr after puparium formation and 2–4 days after puparium formation, respectively, according to the modENCODE tissue and temporal expression data [27,52]. This resulted in 16 additional candidate genes: *ru*, *CG10625*, *sinu*, *Sucb*, *dally*, *CG32052*, *Nc*, *Cerk*, *kkv*, *CG15803*, *loco*, *TwdlC*, *kay*, *CG1340*, *CG42594*, and *CG42340*. For each candidate gene, we tested either an Exelixis transposon insertion line [20], a VDRC RNAi line [21], or when possible, both a mutation and RNAi construct. We assessed the proportion of melanization for both T5 and T6 for all candidate genes.

We evaluated 15 Exelixis transposon insertion lines: *CG33298* ^*d10678a*^, *Fili* ^*f04573*^, *mwh* ^*d01620*^, *Klp61F* ^*f02870*^, *CG9134* ^*e00088*^, *CG7852* ^*c04511*^, *klar* ^*d05910*^, *Glut1* ^*d05758*^, *Efa6* ^*f03476*^, *CG10625* ^*e01211*^, *Sucb* ^*e01940*^, *dally* ^*f01097*^, *kkv* ^*c06225*^, *loco* ^*d09879*^, and *kay* ^*f02002*^. The Exelixis progenitor *w* ^*1118*^ line was used as a control. The KK and GD library progenitor lines were used to make control crosses for the RNAi knockdown experiments. Males from the *GAL4* driver line were crossed to virgin females of the VDRC *UAS* line for all crosses. Three *GAL4* driver lines were used for the RNAi crosses. All VDRC *UAS* lines were crossed with the full-body *tubulin*-*GAL4*/*Sb* driver and a *pannier*-*GAL4* driver (*y* ^1^ *w* ^1118^; *P*{*w* ^+mW.hs^ = *GawB*}*pnr* ^MD237^/*TM3*, *P*{*w* ^+mC^ = *UAS*-*y*.C}MC2, *Ser* ^1^). In instances of lethality with the tubulin-*GAL4/Sb* driver, the lines were crossed to another full-body *ubiquitin*-*GAL4*/*Cy* driver. All *GAL4*-driver lines were obtained from the Bloomington, Indiana *Drosophila* Stock Center. We tested in total 26 RNAi knockdown constructs: *CG33298*, *Fili*, *Vmat*, *mwh*, *Klp61F*, *CG9134*, *CG7852*, *CG1887*, *klar*, *Efa6*, *btn*, *ru*, *CG10625*, *sinu*, *dally*, *CG32052*, *Nc*, *Cerk*, *kkv*, *CG15803*, *loco*, *TwdlC*, *kay*, *CG1340*, *CG42594*, and *CG42340*.

We reared three independent replicates for each Exelixis transposon insertion line, for each RNAi cross and for the appropriate controls under the same conditions as the DGRP lines, but in 8 oz. bottles with a controlled adult density of 20 males and 20 virgin females. We scored the proportion of melanization on T5 and T6 for 50 5–8 day old female progeny per replicate (*N* = 150 flies per genotype) from each Exelixis line or RNAi cross. In a few instances where viability was low fewer than 50 individuals per replicate were scored: *pnr-GAL4* x *sinu* (*N* = 23), *ubi-GAL4* x *CG1887* (*N* = 90), and *ubi-GAL4* x *klar* (*N* = 95). Means of test lines were compared to those of the appropriate controls with a Dunnett's test, which corrects for multiple testing, using JMP Pro 10.0.0 (2012 SAS Institute Inc.)

### Dissection and photography

After mutant lines and RNAi knockdown progeny were scored for pigmentation, they were preserved in a 3:1 ethanol/glycerol solution and stored at 4°C until dissection for imaging. The fly cuticles were dissected from the abdomen and mounted to a glass slide using Permount and a glass cover slip. All photographs were taken with an Olympus DP25 microscope camera on an Olympus SZ61 stereo microscope.

## Supporting Information

S1 Fig Quantile-quantile plots of *P*-values for genetic variants in GWA analyses. (**A**) T5. (**B**) T6. (**C**) T5—T6.
(EPS)

S2 Fig Additional variance explained by significant variants after accounting for known pigmentation genes. For each GWAS conducted (T5, T6, Average, and Difference), the additional variance explained by significant variants after accounting for variants in known pigmentation genes (*y*-axis) is plotted against variance explained by the same variants without accounting for variants in known pigmentation genes. Each colored point represents an individual variant identified in the GWAS and classified according to the genes to which are mapped. Note that not all GWAS identified variants were in the known pigmentation genes.
(PDF)

S3 Fig *pannier* expression pattern. (**A**) schematic of *pnr* expression (yellow) in the *D*. *melanogaster* abdomen. Positive control crosses of (**B**) *pnr-GAL4* x UAS-*ebony* and (**C**) *pnr*-*GAL4* x UAS-*tan* as examples.
(EPS)

S4 Fig Candidate genes affecting differences in thoracic pigmentation. (**A**) Exelixis *w* ^*1118*^ control and (**B**) *Efa6* ^*f03476*^. (**C**) *ubi*-*GAL4* x VDRC GD library control and (**D**) *ubi*-*GAL4* x *klar*-*UAS*.
(EPS)

S5 Fig Representative female of the *ubi*-*GAL4* x *CG1887* RNAi knockdown. The cuticle is semi-transparent and weak. The meconium (M) remains lodged at the end of the abdomen in this cross; all individuals die within 24 hours after eclosion. The third thoracic leg (T3) is malformed.
(EPS)

S6 Fig Power to detect QTLs associated with quantitative trait variation in the DGRP. Assuming a population size of 175 (the same as the present study), we estimated power to detect a QTL by simulating phenotypes based on the genetic parameters for 1,000 times and performing single marker regression. Power was calculated as the number of iterations when the *P*-values were below 1 x 10^–5^ divided by 1,000 (the number of total iterations). We simulated phenotypes with underlying QTLs of effects ranging from 0 to 1.0 phenotypic standard deviation and minor allele frequency of the QTL ranging from 0.05 to 0.50. The range of the observed effect sizes in the present study is indicated by grey shading.
(EPS)

S1 Table Pigmentation data for DGRP lines. Sheet (**A**) DGRP Tergite 5 and 6 line means and standard errors. (**B**) DGRP raw data.
(XLSX)

S2 Table Analyses of variance of female abdominal pigmentation. *σ* ^2^: Variance component; *H* ^2^: Broad sense heritability.
(DOC)

S3 Table Analyses of variance of the effects of *Wolbachia* infection and common polymorphic inversions on pigmentation of female abdominal tergites. (DOC)

S4 Table Genome wide association analyses for female abdominal pigmentation. (XLSX)

S5 Table S4 Table in vcf format. (RTF)

S6 Table Number of GWAS variants affecting each type of identified regulatory element. (DOC)

S7 Table List of candidate genes, VDRC RNAi lines, and Exelixis transposon insertion lines. (DOC)

S8 Table Mutant and RNAi knockdown summary statistics, Dunnett's test results, and deviations. Sheet (**A**) Exelixis mutants and control data. (**B**) *tub-GAL4* x UAS lines from the VDRC KK library. (**C**) *tub-GAL4* x UAS lines from the VDRC GD library. (**D**) *pnr-GAL4* x UAS lines from the VDRC KK library. (**E**) *pnr-GAL4* x UAS lines from the VDRC GD library. (**F**) *ubi-GAL4* x UAS lines from the VDRC KK library. (**G**) *ubi-GAL4* x UAS lines from the VDRC GD library.
(XLSX)

S9 Table Mutant and RNAi knockdown raw data. Sheet (**A**) Exelixis mutants and control data. (**B**) *tub-GAL4* x UAS lines from the VDRC KK library. (**C**) *tub-GAL4* x UAS lines from the VDRC GD library. (**D**) *pnr-GAL4* x UAS lines from the VDRC KK library. (**E**) *pnr-GAL4* x UAS lines from the VDRC GD library. (**F**) *ubi-GAL4* x UAS lines from the VDRC KK library. (**G**) *ubi-GAL4* x UAS lines from the VDRC GD library.
(XLSX)
