# Supplementary figures and images for: Genetic Architecture of Abdominal Pigmentation in Drosophila melanogaster
Source: PLoS Genet. 2015 May 1;11(5):e1005163. doi: 10.1371/journal.pgen.1005163 (PMC4416719; doi:10.1371/journal.pgen.1005163)

**T5**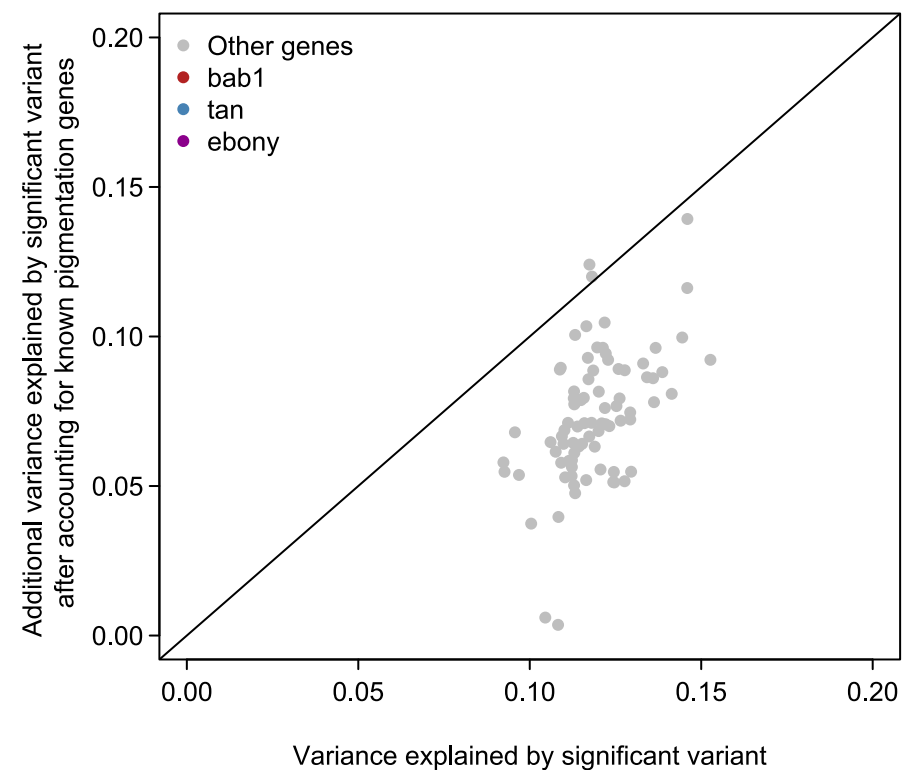**T6**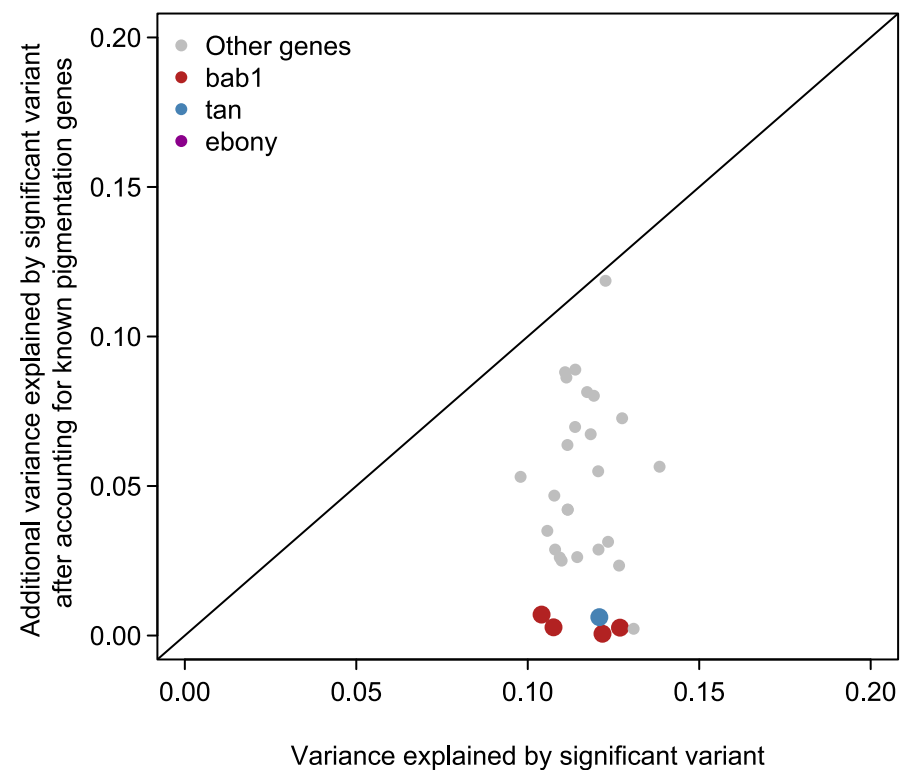**(T5 + T6)/2**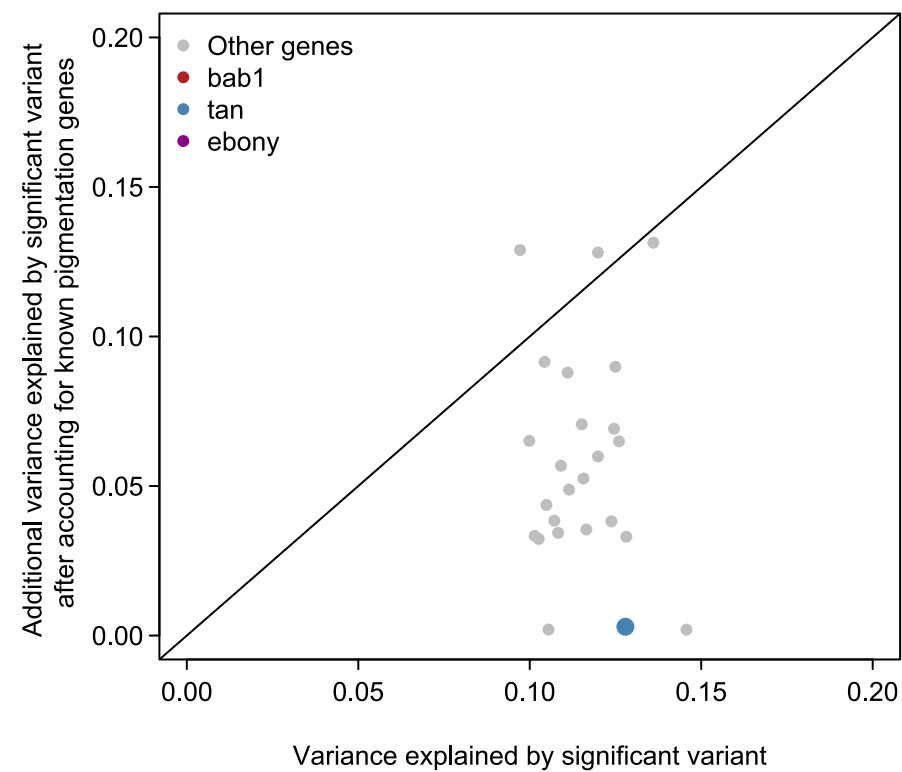**T5 - T6**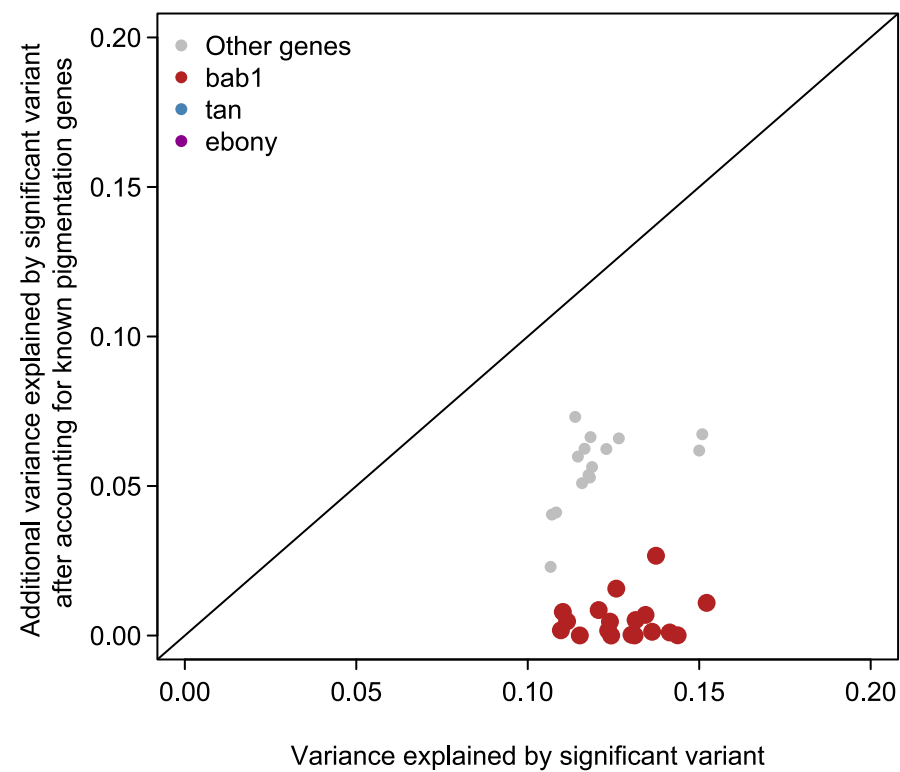

Supplement: S2 Fig — For each GWAS conducted (T5, T6, Average, and Difference), the additional variance explained by significant variants after accounting for variants in known pigmentation genes (y-axis) is plotted against variance explained by the same variants without accounting for variants in known pigmentation genes. Each colored point represents an individual variant identified in the GWAS and classified according to the genes to which are mapped. Note that not all GWAS identified variants were in the known pigmentation genes. (PDF) [file pgen.1005163.s002.pdf]
