# Supplementary material for: Genetic Architecture of Abdominal Pigmentation in Drosophila melanogaster
Source: PLoS Genet. 2015 May 1;11(5):e1005163. doi: 10.1371/journal.pgen.1005163 (PMC4416719; doi:10.1371/journal.pgen.1005163)
Supplement: S2 Table — σ 2: Variance component; H 2: Broad sense heritability. (DOC) [file pgen.1005163.s008.doc]

| **Analysis** | **Source** | **Degrees of Freedom** | **Type III Sums of Squares** | **Mean Squares** | **F** | ***P*-value** | ***σ*^2^** | ***H*^2^** |
| --- | --- | --- | --- | --- | --- | --- | --- | --- |
| **T5** | Line | 174 | 436.96 | 2.51 | 11.55 | 4.68x10^-48^ | 0.2322 | 0.6585 |
|  | Vial (Line) | 172 | 37.41 | 0.22 | 1.68 | 5.07x10^-7^ | 0.0175 |  |
|  | Error | 1,378 | 178.20 | 0.13 |  |  | 0.1295 |  |
| **T6** | Line | 174 | 1578.22 | 9.07 | 46.96 | 6.65x10^-96^ | 0.8978 | 0.8811 |
|  | Vial (line) | 172 | 33.22 | 0.19 | 1.57 | 1.24x10^-5^ | 0.0141 |  |
|  | Error | 1,378 | 169.38 | 0.12 |  |  | 0.1230 |  |
| **T5 and T6 Pooled** | Line | 174 | 1529.45 | 8.79 | 3.15 | 9.09x10^-14^ | 0.3041 | 0.8215 |
|  | Tergite | 1 | 1034.76 | 1034.76 | 374.37 | 2.97x10^-45^ | fixed |  |
|  | Line×Tergite | 174 | 485.73 | 2.79 | 13.60 | 6.91x10^-90^ | 0.2609 |  |
|  | Vial (Line×Tergite) | 344 | 70.63 | 0.21 | 1.63 | 6.09x10^-11^ | 0.0158 |  |
|  | Error | 2,756 | 347.58 | 0.13 |  |  | 0.1262 |  |

**Table S2.** Analyses of variance of female abdominal pigmentation. *σ*^2^: Variance component; *H*^2^: Broad sense heritability.
