# Supplementary material for: Genetic Architecture of Abdominal Pigmentation in Drosophila melanogaster
Source: PLoS Genet. 2015 May 1;11(5):e1005163. doi: 10.1371/journal.pgen.1005163 (PMC4416719; doi:10.1371/journal.pgen.1005163)
Supplement: S3 Table — (DOC) [file pgen.1005163.s009.doc]

**Table S3.** Analyses of variance of the effects of *Wolbachia* infection and common polymorphic inversions on pigmentation of female abdominal tergites. T5: tergite 5; T6: tergite 6; T5 – T6: Difference between tergites 5 and 6.

| **Analysis** | **Covariates** | **Degrees of Freedom** | **Sums of Squares**  **(Type III)** | **Residual Sums of Squares** | **AIC** | **F** | ***P*-value** |
| --- | --- | --- | --- | --- | --- | --- | --- |
| **T5** | none |  |  | 41.97 | -225.88 |  |  |
|  | *Wolbachia* | 1 | 0.08 | 42.05 | -227.54 | 0.31 | 0.58 |
|  | *In(2L)t* | 2 | 0.34 | 42.31 | -228.45 | 0.67 | 0.51 |
|  | *In(2R)NS* | 2 | 0.19 | 42.16 | -229.08 | 0.37 | 0.69 |
|  | *In(3R)P* | 2 | 0.89 | 42.86 | -226.21 | 1.73 | 0.18 |
|  | *In(3R)K* | 2 | 0.20 | 42.16 | -229.07 | 0.38 | 0.68 |
|  | *In(3R)Mo* | 2 | 0.23 | 42.20 | -228.94 | 0.44 | 0.64 |
| **T6** | none |  |  | 147.71 | -5.67 |  |  |
|  | *Wolbachia* | 1 | 0.01 | 147.72 | -7.66 | 0.01 | 0.92 |
|  | *In(2L)t* | 2 | 4.62 | 152.33 | -4.28 | 2.55 | 0.08 |
|  | *In(2R)NS* | 2 | 4.08 | 151.79 | -4.90 | 2.25 | 0.11 |
|  | *In(3R)P* | 2 | 0.94 | 148.64 | -8.57 | 0.52 | 0.60 |
|  | *In(3R)K* | 2 | 0.80 | 148.50 | -8.73 | 0.44 | 0.64 |
|  | *In(3R)Mo* | 2 | 0.38 | 148.08 | -9.23 | 0.21 | 0.81 |
| **T5 – T6** | none |  |  | 87.52 | -97.27 |  |  |
|  | *Wolbachia* | 1 | 0.14 | 87.66 | -98.99 | 0.26 | 0.61 |
|  | *In(2L)t* | 2 | 3.58 | 91.09 | -94.26 | 3.33 | 0.04 |
|  | *In(2R)NS* | 2 | 5.37 | 92.89 | -90.84 | 5.01 | 0.01 |
|  | *In(3R)P* | 2 | 0.00 | 87.52 | -101.26 | 0.00 | 1.00 |
|  | *In(3R)K* | 2 | 1.48 | 89.00 | -98.34 | 1.38 | 0.26 |
|  | *In(3R)Mo* | 2 | 0.16 | 87.67 | -100.96 | 0.14 | 0.87 |
