# Supplementary material for: Genetic Architecture of Abdominal Pigmentation in Drosophila melanogaster
Source: PLoS Genet. 2015 May 1;11(5):e1005163. doi: 10.1371/journal.pgen.1005163 (PMC4416719; doi:10.1371/journal.pgen.1005163)
Supplement: S5 Table — (RTF) [file pgen.1005163.s011.rtf]

##VCF accompanying Dembeck et al. (re-format of Table S4)
##INFO=<ID=T5EFF,Number=1,Type=Float,Description="Effect size for T5">
##INFO=<ID=T5VAR,Number=1,Type=Float,Description="Variance explained for T5">
##INFO=<ID=T5VARACC,Number=1,Type=Float,Description="Variance explained for T5 after accounting for known pigmentation genes">
##INFO=<ID=T5PVAL,Number=1,Type=Float,Description="P value for T5 (regression)">
##INFO=<ID=T5PMIXED,Number=1,Type=Float,Description="P value for T5 (mixed model)">
##INFO=<ID=T6EFF,Number=1,Type=Float,Description="Effect size for T6">
##INFO=<ID=T6VAR,Number=1,Type=Float,Description="Variance explained for T6">
##INFO=<ID=T6VARACC,Number=1,Type=Float,Description="Variance explained for T6 after accounting for known pigmentation genes">
##INFO=<ID=T6PVAL,Number=1,Type=Float,Description="P value for T6 (regression)">
##INFO=<ID=T6PMIXED,Number=1,Type=Float,Description="P value for T6 (mixed model)">
##INFO=<ID=AVGEFF,Number=1,Type=Float,Description="Effect size for average of T5 and T6">
##INFO=<ID=AVGVAR,Number=1,Type=Float,Description="Variance explained for average of T5 and T6">
##INFO=<ID=AVGVARACC,Number=1,Type=Float,Description="Variance explained for average of T5 and T6 after accounting for known pigmentation genes">
##INFO=<ID=AVGPVAL,Number=1,Type=Float,Description="P value for average of T5 and T6 (regression)">
##INFO=<ID=AVGPMIXED,Number=1,Type=Float,Description="P value for average of T5 and T6 (mixed model)">
##INFO=<ID=DIFFEFF,Number=1,Type=Float,Description="Effect size for T5-T6">
##INFO=<ID=DIFFVAR,Number=1,Type=Float,Description="Variance explained for T5-T6">
##INFO=<ID=DIFFVARACC,Number=1,Type=Float,Description="Variance explained for T5-T6 after accounting for known pigmentation genes">
##INFO=<ID=DIFFPVAL,Number=1,Type=Float,Description="P value for average of T5-T6 (regression)">
##INFO=<ID=DIFFPMIXED,Number=1,Type=Float,Description="P value for average of T5-T6 (mixed model)">
##INFO=<ID=ANNOT,Number=.,Type=STRING,Description="Annotation for the variant">
##FORMAT=<ID=GT,Number=1,Type=String,Description="Genotype">
#CHROM	POS	ID	REF	ALT	QUAL	FILTER	INFO	FORMAT	line_101	line_105	line_109	line_129	line_136	line_138	line_142	line_149	line_153	line_158	line_161	line_176	line_177	line_181	line_189	line_195	line_208	line_21	line_217	line_223	line_227	line_228	line_229	line_233	line_235	line_237	line_239	line_256	line_26	line_28	line_280	line_301	line_303	line_304	line_306	line_307	line_309	line_310	line_313	line_315	line_317	line_318	line_319	line_324	line_325	line_332	line_335	line_336	line_338	line_340	line_350	line_352	line_356	line_357	line_358	line_359	line_360	line_361	line_362	line_365	line_367	line_370	line_371	line_373	line_374	line_375	line_377	line_379	line_38	line_380	line_381	line_382	line_383	line_385	line_386	line_391	line_392	line_399	line_409	line_41	line_42	line_426	line_427	line_437	line_440	line_441	line_443	line_45	line_461	line_486	line_49	line_491	line_492	line_502	line_508	line_509	line_517	line_531	line_535	line_555	line_563	line_57	line_584	line_589	line_59	line_595	line_639	line_642	line_646	line_703	line_705	line_707	line_712	line_714	line_716	line_721	line_727	line_73	line_730	line_732	line_737	line_738	line_748	line_75	line_757	line_761	line_765	line_774	line_776	line_783	line_786	line_787	line_790	line_796	line_799	line_801	line_802	line_804	line_805	line_808	line_810	line_812	line_818	line_819	line_820	line_821	line_822	line_83	line_832	line_837	line_843	line_849	line_85	line_850	line_852	line_853	line_855	line_857	line_859	line_861	line_879	line_88	line_882	line_887	line_890	line_892	line_894	line_897	line_900	line_907	line_908	line_91	line_911	line_913	line_93
2L	1479243	2L_1479243_SNP	C	A	.	.	T5EFF=0.02592;T5VAR=0.00150244840951694;T5VARACC=0.000530905363145817;T5PVAL=0.6233;T5MIXED=0.555765750612173;T6EFF=0.3404;T6VAR=0.0786893216269917;T6VARACC=0.0335449188633913;T6PVAL=0.0002868;T6MIXED=0.000239320931652168;AVGEFF=0.1832;AVGVAR=0.0449528874503895;AVGVARACC=0.0146386399155704;AVGPVAL=0.006587;AVGMIXED=0.00472973654174126;DIFFEFF=-0.3145;DIFFVAR=0.115832616211106;DIFFVARACC=0.0509524895607639;DIFFPVAL=8.781e-06;DIFFMIXED=1.02308724039962e-05;ANNOT=(FBgn0263321|CG43402|35927 bp DOWNSTREAM),(FBgn0051928|CG31928|13052 bp DOWNSTREAM)	GT	0/0	0/0	0/0	0/0	0/0	0/0	1/1	0/0	.	0/0	0/0	0/0	0/0	0/0	0/0	0/0	0/0	0/0	1/1	1/1	0/0	0/0	0/0	0/0	1/1	0/0	0/0	0/0	0/0	1/1	0/0	.	0/0	0/0	0/0	0/0	0/0	0/0	0/0	.	1/1	0/0	0/0	1/1	1/1	1/1	.	0/0	0/0	1/1	0/0	0/0	0/0	0/0	0/0	0/0	0/0	0/0	0/0	0/0	0/0	1/1	1/1	0/0	0/0	0/0	.	1/1	0/0	.	0/0	0/0	0/0	1/1	0/0	0/0	1/1	1/1	0/0	0/0	0/0	0/0	1/1	0/0	0/0	0/0	.	0/0	0/0	.	0/0	0/0	0/0	0/0	0/0	0/0	0/0	0/0	0/0	0/0	0/0	0/0	0/0	0/0	0/0	0/0	0/0	0/0	0/0	0/0	1/1	0/0	0/0	0/0	0/0	0/0	0/0	0/0	0/0	1/1	0/0	.	0/0	0/0	0/0	1/1	0/0	.	0/0	0/0	0/0	0/0	0/0	0/0	0/0	1/1	.	0/0	0/0	0/0	1/1	0/0	0/0	1/1	0/0	0/0	0/0	1/1	0/0	0/0	1/1	0/0	.	0/0	0/0	0/0	0/0	0/0	0/0	0/0	1/1	0/0	0/0	0/0	1/1	0/0	0/0	0/0	0/0	0/0	0/0	0/0	1/1	0/0	0/0
2L	15101358	2L_15101358_SNP	A	G	.	.	T5EFF=-0.3889;T5VAR=0.124348747935557;T5VARACC=0.0513183719130082;T5PVAL=2.403e-06;T5MIXED=3.61234498766835e-06;T6EFF=-0.4518;T6VAR=0.0479591890169862;T6VARACC=0.0115679055586037;T6PVAL=0.004115;T6MIXED=0.00400013602396955;AVGEFF=-0.4204;AVGVAR=0.084097581852876;AVGVARACC=0.0327655893317558;AVGPVAL=0.0001251;AVGMIXED=0.000155700631999144;DIFFEFF=0.06287;DIFFVAR=0.00155556772173035;DIFFVARACC=0.0044906817864625;DIFFPVAL=0.6096;DIFFMIXED=0.611295748156609;ANNOT=(FBgn0028879|CG15270|4443 bp DOWNSTREAM),(FBgn0028878|CG15269|8047 bp DOWNSTREAM)	GT	0/0	0/0	0/0	0/0	0/0	0/0	0/0	0/0	0/0	1/1	1/1	0/0	0/0	0/0	0/0	1/1	0/0	0/0	0/0	0/0	0/0	0/0	0/0	0/0	0/0	0/0	0/0	0/0	0/0	0/0	0/0	.	0/0	0/0	0/0	0/0	0/0	0/0	0/0	0/0	0/0	0/0	0/0	0/0	0/0	0/0	0/0	0/0	0/0	0/0	0/0	0/0	0/0	0/0	0/0	1/1	0/0	0/0	0/0	0/0	0/0	0/0	0/0	0/0	0/0	0/0	0/0	0/0	0/0	0/0	0/0	0/0	0/0	0/0	0/0	0/0	0/0	0/0	.	0/0	0/0	.	0/0	0/0	0/0	0/0	0/0	0/0	0/0	0/0	0/0	0/0	0/0	.	0/0	0/0	0/0	0/0	0/0	0/0	0/0	0/0	0/0	0/0	0/0	1/1	0/0	0/0	0/0	0/0	0/0	0/0	0/0	0/0	0/0	0/0	0/0	0/0	0/0	0/0	0/0	0/0	0/0	0/0	0/0	0/0	0/0	0/0	0/0	0/0	0/0	0/0	0/0	0/0	0/0	0/0	0/0	0/0	0/0	0/0	0/0	0/0	0/0	0/0	0/0	0/0	0/0	0/0	0/0	1/1	0/0	0/0	0/0	0/0	0/0	.	0/0	0/0	0/0	0/0	0/0	0/0	0/0	0/0	0/0	0/0	0/0	1/1	0/0	0/0	0/0	1/1	0/0	0/0	1/1
2L	15101580	2L_15101580_SNP	T	A	.	.	T5EFF=-0.3863;T5VAR=0.124646759464592;T5VARACC=0.0512000790195024;T5PVAL=2.872e-06;T5MIXED=3.98862810621797e-06;T6EFF=-0.4579;T6VAR=0.0496335388254645;T6VARACC=0.0127198739519951;T6PVAL=0.003805;T6MIXED=0.00350720183240796;AVGEFF=-0.4221;AVGVAR=0.0851877390847983;AVGVARACC=0.0334674975212988;AVGPVAL=0.0001296;AVGMIXED=0.000142019470326705;DIFFEFF=0.07166;DIFFVAR=0.00207230180210849;DIFFVARACC=0.00408373118740019;DIFFPVAL=0.5591;DIFFMIXED=0.561268541783411;ANNOT=(FBgn0028879|CG15270|4665 bp DOWNSTREAM),(FBgn0028878|CG15269|7825 bp DOWNSTREAM)	GT	0/0	0/0	0/0	0/0	0/0	0/0	0/0	0/0	0/0	1/1	1/1	0/0	0/0	0/0	0/0	1/1	0/0	0/0	0/0	0/0	0/0	0/0	0/0	0/0	0/0	0/0	0/0	0/0	.	0/0	0/0	.	0/0	0/0	0/0	0/0	0/0	0/0	0/0	0/0	0/0	0/0	0/0	0/0	0/0	0/0	0/0	0/0	0/0	0/0	0/0	0/0	0/0	0/0	0/0	1/1	0/0	0/0	0/0	0/0	0/0	0/0	0/0	0/0	0/0	0/0	0/0	0/0	0/0	0/0	0/0	0/0	0/0	0/0	0/0	0/0	0/0	0/0	0/0	0/0	0/0	.	0/0	0/0	0/0	0/0	0/0	.	0/0	0/0	0/0	0/0	0/0	.	0/0	0/0	0/0	0/0	0/0	0/0	0/0	0/0	0/0	0/0	0/0	1/1	0/0	0/0	0/0	0/0	0/0	0/0	0/0	0/0	0/0	0/0	0/0	.	0/0	0/0	0/0	0/0	0/0	0/0	0/0	.	0/0	0/0	0/0	0/0	0/0	0/0	0/0	0/0	0/0	0/0	0/0	0/0	0/0	0/0	0/0	0/0	0/0	0/0	0/0	0/0	0/0	0/0	0/0	1/1	0/0	0/0	0/0	0/0	0/0	.	0/0	0/0	0/0	0/0	0/0	0/0	0/0	0/0	0/0	0/0	0/0	1/1	0/0	0/0	0/0	1/1	0/0	0/0	1/1
2L	15240081	2L_15240081_SNP	A	T	.	.	T5EFF=-0.185;T5VAR=0.109367929745111;T5VARACC=0.0666411112732974;T5PVAL=1.83e-05;T5MIXED=5.5239484602324e-06;T6EFF=-0.1339;T6VAR=0.0164293406777931;T6VARACC=0.00618709222287112;T6PVAL=0.1051;T6MIXED=0.0773020957087656;AVGEFF=-0.1595;AVGVAR=0.0467948342680903;AVGVARACC=0.0321297915294252;AVGPVAL=0.005849;AVGMIXED=0.00254768163023096;DIFFEFF=-0.05105;DIFFVAR=0.0040972542056112;DIFFVARACC=0.0090117933282724;DIFFPVAL=0.4198;DIFFMIXED=0.43796460918023;ANNOT=(FBgn0028516|ZnT35C|INTRON)	GT	0/0	0/0	.	1/1	0/0	0/0	0/0	1/1	0/0	1/1	.	0/0	0/0	1/1	0/0	1/1	0/0	1/1	0/0	0/0	0/0	0/0	0/0	.	0/0	.	0/0	1/1	0/0	1/1	0/0	.	1/1	1/1	.	0/0	0/0	0/0	0/0	0/0	0/0	0/0	1/1	0/0	0/0	0/0	0/0	0/0	0/0	1/1	1/1	0/0	0/0	0/0	1/1	0/0	0/0	0/0	0/0	1/1	0/0	0/0	0/0	1/1	1/1	0/0	0/0	0/0	0/0	0/0	0/0	1/1	0/0	1/1	1/1	0/0	1/1	1/1	0/0	1/1	0/0	.	0/0	1/1	0/0	1/1	.	.	0/0	0/0	0/0	0/0	0/0	.	0/0	0/0	0/0	0/0	0/0	0/0	0/0	0/0	0/0	1/1	0/0	1/1	1/1	0/0	0/0	0/0	0/0	0/0	0/0	1/1	1/1	0/0	1/1	0/0	0/0	0/0	0/0	0/0	0/0	0/0	1/1	0/0	0/0	0/0	.	1/1	0/0	0/0	1/1	0/0	0/0	0/0	0/0	0/0	0/0	0/0	0/0	1/1	0/0	0/0	0/0	0/0	1/1	1/1	0/0	1/1	0/0	.	0/0	1/1	0/0	.	1/1	1/1	0/0	0/0	0/0	.	0/0	0/0	0/0	1/1	0/0	0/0	0/0	0/0	0/0	0/0	0/0	1/1	1/1
2L	16369024	2L_16369024_SNP	A	T	.	.	T5EFF=0.139;T5VAR=0.0837247935822687;T5VARACC=0.0320884155272144;T5PVAL=0.0001636;T5MIXED=0.000444118785213751;T6EFF=0.3167;T6VAR=0.118355221207657;T6VARACC=0.0673176272264527;T6PVAL=6.057e-06;T6MIXED=4.74960195490631e-06;AVGEFF=0.2279;AVGVAR=0.126034706297207;AVGVARACC=0.0649487641258622;AVGPVAL=2.884e-06;AVGMIXED=4.54311892806744e-06;DIFFEFF=-0.1777;DIFFVAR=0.0620608567652103;DIFFVARACC=0.0273963022847185;DIFFPVAL=0.001252;DIFFMIXED=0.000973936195019776;ANNOT=(FBgn0028841|jhamt|2047 bp DOWNSTREAM),(FBgn0028523|CG5888|31145 bp UPSTREAM)	GT	1/1	0/0	.	.	1/1	0/0	1/1	0/0	1/1	1/1	1/1	1/1	1/1	1/1	1/1	0/0	1/1	1/1	1/1	1/1	1/1	0/0	1/1	0/0	0/0	0/0	1/1	0/0	0/0	1/1	1/1	.	0/0	1/1	0/0	0/0	0/0	0/0	1/1	0/0	1/1	1/1	0/0	1/1	0/0	1/1	0/0	0/0	0/0	1/1	0/0	0/0	.	1/1	0/0	0/0	1/1	0/0	1/1	1/1	1/1	1/1	0/0	0/0	0/0	0/0	0/0	1/1	1/1	1/1	0/0	0/0	1/1	0/0	0/0	0/0	0/0	0/0	.	1/1	1/1	1/1	1/1	1/1	1/1	0/0	0/0	0/0	1/1	1/1	0/0	1/1	1/1	.	1/1	0/0	0/0	1/1	0/0	0/0	0/0	0/0	0/0	1/1	0/0	0/0	0/0	1/1	1/1	1/1	1/1	1/1	0/0	.	1/1	1/1	1/1	1/1	1/1	0/0	0/0	0/0	1/1	0/0	0/0	0/0	1/1	1/1	0/0	1/1	1/1	1/1	1/1	1/1	0/0	0/0	.	0/0	1/1	1/1	0/0	0/0	1/1	0/0	1/1	0/0	1/1	0/0	1/1	0/0	1/1	1/1	0/0	0/0	1/1	.	0/0	1/1	0/0	0/0	1/1	.	0/0	0/0	0/0	0/0	0/0	0/0	1/1	1/1	0/0	0/0	1/1	1/1	0/0
2L	1762040	2L_1762040_SNP	T	A	.	.	T5EFF=-0.1201;T5VAR=0.0552232690025274;T5VARACC=0.0282103966000087;T5PVAL=0.002307;T5MIXED=0.000980579955009372;T6EFF=-0.3037;T6VAR=0.101921337589955;T6VARACC=0.0564666904206685;T6PVAL=2.76e-05;T6MIXED=1.05770458894897e-05;AVGEFF=-0.2119;AVGVAR=0.099883453532445;AVGVARACC=0.0651056731603861;AVGPVAL=3.354e-05;AVGMIXED=8.22951818308423e-06;DIFFEFF=0.1836;DIFFVAR=0.0630990394394557;DIFFVARACC=0.0193127158519111;DIFFPVAL=0.001097;DIFFMIXED=0.000782783143679833;ANNOT=(FBgn0031365|CG17650|2947 bp UPSTREAM),(FBgn0045497|Gr22e|22022 bp DOWNSTREAM)	GT	.	0/0	0/0	0/0	0/0	0/0	1/1	0/0	0/0	1/1	0/0	1/1	0/0	0/0	0/0	1/1	1/1	0/0	0/0	0/0	0/0	1/1	1/1	0/0	0/0	0/0	1/1	1/1	0/0	1/1	0/0	.	0/0	0/0	0/0	0/0	0/0	1/1	0/0	0/0	0/0	0/0	1/1	1/1	1/1	0/0	1/1	0/0	.	0/0	0/0	0/0	0/0	0/0	0/0	0/0	0/0	0/0	1/1	1/1	0/0	0/0	1/1	0/0	0/0	0/0	.	0/0	1/1	1/1	0/0	0/0	0/0	0/0	0/0	0/0	1/1	0/0	0/0	1/1	0/0	0/0	0/0	0/0	1/1	0/0	0/0	1/1	1/1	0/0	1/1	1/1	0/0	.	1/1	0/0	0/0	1/1	0/0	0/0	0/0	0/0	0/0	0/0	1/1	1/1	1/1	0/0	0/0	0/0	0/0	1/1	1/1	1/1	1/1	0/0	0/0	1/1	1/1	0/0	1/1	1/1	0/0	1/1	1/1	0/0	1/1	0/0	0/0	1/1	0/0	0/0	1/1	.	1/1	0/0	0/0	0/0	0/0	0/0	1/1	.	1/1	1/1	0/0	1/1	0/0	0/0	0/0	1/1	1/1	.	.	1/1	0/0	1/1	0/0	0/0	0/0	1/1	1/1	1/1	1/1	0/0	1/1	1/1	0/0	0/0	0/0	1/1	0/0	1/1	1/1	1/1	1/1
2L	1890502	2L_1890502_SNP	T	A	.	.	T5EFF=-0.2429;T5VAR=0.115262297722404;T5VARACC=0.0641056837529561;T5PVAL=8.695e-06;T5MIXED=8.44752070460239e-06;T6EFF=-0.2355;T6VAR=0.0308646785687288;T6VARACC=0.0281537686173512;T6PVAL=0.02444;T6MIXED=0.0264190979133992;AVGEFF=-0.2392;AVGVAR=0.0646704846441913;AVGVARACC=0.0547880319789463;AVGPVAL=0.001016;AVGMIXED=0.00135701403279117;DIFFEFF=-0.007334;DIFFVAR=4.98297132164375e-05;DIFFVARACC=4.5235212353556e-05;DIFFPVAL=0.9285;DIFFMIXED=0.924397014032153;ANNOT=(FBgn0031374|CG7337|INTRON)	GT	.	0/0	0/0	.	0/0	0/0	0/0	0/0	0/0	1/1	0/0	0/0	0/0	0/0	0/0	1/1	1/1	0/0	1/1	1/1	0/0	0/0	0/0	0/0	0/0	0/0	0/0	1/1	0/0	1/1	0/0	.	0/0	0/0	0/0	0/0	0/0	1/1	0/0	0/0	0/0	.	0/0	0/0	0/0	0/0	0/0	0/0	0/0	0/0	0/0	.	1/1	0/0	0/0	0/0	0/0	1/1	0/0	1/1	0/0	0/0	0/0	0/0	0/0	.	0/0	0/0	0/0	0/0	0/0	.	0/0	.	0/0	0/0	1/1	0/0	0/0	0/0	0/0	0/0	0/0	0/0	0/0	0/0	.	0/0	0/0	0/0	1/1	0/0	0/0	.	0/0	0/0	0/0	0/0	1/1	0/0	0/0	0/0	0/0	0/0	1/1	0/0	0/0	0/0	1/1	0/0	0/0	0/0	0/0	0/0	0/0	0/0	0/0	0/0	0/0	0/0	0/0	0/0	0/0	0/0	0/0	0/0	0/0	0/0	0/0	0/0	0/0	0/0	0/0	0/0	0/0	0/0	0/0	0/0	1/1	0/0	0/0	0/0	0/0	0/0	0/0	0/0	0/0	1/1	0/0	0/0	0/0	0/0	0/0	1/1	1/1	.	0/0	0/0	0/0	0/0	0/0	0/0	0/0	0/0	1/1	0/0	0/0	0/0	0/0	0/0	0/0	0/0	0/0	1/1	0/0
2L	1981812	2L_1981812_SNP	C	T	.	.	T5EFF=-0.3017;T5VAR=0.113132301512585;T5VARACC=0.0776905539811517;T5PVAL=8.824e-06;T5MIXED=1.13215768805303e-05;T6EFF=-0.3043;T6VAR=0.03396041968465;T6VARACC=0.0344710968138596;T6PVAL=0.01712;T6MIXED=0.0189168464701476;AVGEFF=-0.303;AVGVAR=0.0673681121772466;AVGVARACC=0.045984974274086;AVGPVAL=0.0007061;AVGMIXED=0.00101367630313805;DIFFEFF=0.002683;DIFFVAR=4.46487630319126e-06;DIFFVARACC=0.00239365004962899;DIFFPVAL=0.9784;DIFFMIXED=0.987284554796506;ANNOT=(FBgn0031378|CG15362|NON_SYNONYMOUS_CODING),(FBgn0031379|CG7289|405 bp UPSTREAM)	GT	.	0/0	0/0	0/0	0/0	0/0	0/0	0/0	0/0	1/1	0/0	0/0	0/0	0/0	0/0	0/0	0/0	0/0	0/0	1/1	0/0	0/0	0/0	0/0	0/0	0/0	0/0	0/0	0/0	0/0	0/0	0/0	0/0	0/0	0/0	0/0	0/0	0/0	0/0	0/0	0/0	0/0	1/1	1/1	0/0	0/0	0/0	.	.	0/0	0/0	0/0	0/0	0/0	0/0	0/0	0/0	0/0	0/0	0/0	0/0	0/0	0/0	0/0	0/0	0/0	0/0	0/0	0/0	0/0	0/0	0/0	0/0	0/0	0/0	0/0	0/0	0/0	0/0	0/0	0/0	0/0	0/0	0/0	0/0	0/0	0/0	0/0	0/0	0/0	1/1	0/0	0/0	.	0/0	1/1	0/0	0/0	0/0	0/0	0/0	0/0	0/0	0/0	0/0	0/0	1/1	0/0	0/0	0/0	0/0	0/0	.	0/0	0/0	0/0	0/0	0/0	0/0	0/0	0/0	0/0	0/0	0/0	0/0	0/0	0/0	1/1	1/1	1/1	0/0	0/0	0/0	0/0	0/0	0/0	0/0	1/1	0/0	0/0	0/0	0/0	0/0	0/0	0/0	1/1	0/0	1/1	0/0	0/0	0/0	.	0/0	0/0	0/0	.	0/0	0/0	.	0/0	0/0	0/0	0/0	0/0	0/0	1/1	0/0	0/0	0/0	0/0	0/0	0/0	0/0	0/0	0/0
2L	20467147	2L_20467147_SNP	C	T	.	.	T5EFF=-0.2462;T5VAR=0.112306562525946;T5VARACC=0.0586209162424674;T5PVAL=7.93e-06;T5MIXED=4.60388800240557e-05;T6EFF=-0.1988;T6VAR=0.0209187697802671;T6VARACC=0.0012946670710704;T6PVAL=0.05987;T6MIXED=0.0551825783422448;AVGEFF=-0.2225;AVGVAR=0.0529091319231751;AVGVARACC=0.0161633217335662;AVGPVAL=0.002548;AVGMIXED=0.00392695012066836;DIFFEFF=-0.04733;DIFFVAR=0.00200637881675831;DIFFVARACC=0.0107385049895311;DIFFPVAL=0.5619;DIFFMIXED=0.575879486167137;ANNOT=(FBgn0032864|CG2493|3703 bp UPSTREAM),(FBgn0262878|CG43233|5646 bp UPSTREAM)	GT	0/0	0/0	0/0	0/0	0/0	0/0	0/0	0/0	0/0	1/1	0/0	0/0	0/0	1/1	0/0	1/1	0/0	0/0	0/0	0/0	0/0	0/0	0/0	0/0	0/0	0/0	0/0	0/0	0/0	0/0	1/1	0/0	0/0	0/0	0/0	0/0	1/1	0/0	0/0	1/1	1/1	0/0	0/0	0/0	0/0	0/0	0/0	0/0	0/0	0/0	0/0	0/0	.	0/0	0/0	0/0	0/0	0/0	0/0	1/1	0/0	0/0	0/0	0/0	0/0	0/0	0/0	0/0	0/0	0/0	0/0	0/0	0/0	1/1	0/0	0/0	1/1	1/1	0/0	0/0	0/0	0/0	1/1	0/0	0/0	0/0	.	1/1	0/0	1/1	1/1	0/0	0/0	0/0	1/1	0/0	0/0	0/0	0/0	0/0	0/0	0/0	0/0	0/0	0/0	1/1	0/0	0/0	0/0	0/0	0/0	0/0	0/0	1/1	0/0	0/0	0/0	0/0	0/0	0/0	0/0	0/0	0/0	0/0	0/0	0/0	0/0	0/0	0/0	0/0	0/0	0/0	0/0	.	0/0	0/0	0/0	0/0	0/0	0/0	0/0	0/0	0/0	0/0	0/0	0/0	0/0	1/1	0/0	1/1	0/0	.	0/0	0/0	0/0	0/0	0/0	0/0	0/0	0/0	0/0	0/0	0/0	0/0	0/0	1/1	0/0	0/0	0/0	0/0	0/0	0/0	0/0	.	1/1
2L	20628607	2L_20628607_SNP	C	A	.	.	T5EFF=0.0897;T5VAR=0.0318178288905025;T5VARACC=0.0222395262939439;T5PVAL=0.02738;T5MIXED=0.0285110730538667;T6EFF=0.3218;T6VAR=0.119274496515801;T6VARACC=0.080173858803587;T6PVAL=1.23e-05;T6MIXED=9.9500105731188e-06;AVGEFF=0.2058;AVGVAR=0.0977498351762396;AVGVARACC=0.06300453098581;AVGPVAL=8.332e-05;AVGMIXED=8.97516438957918e-05;DIFFEFF=-0.2321;DIFFVAR=0.10565024173517;DIFFVARACC=0.0690571088778411;DIFFPVAL=4.142e-05;DIFFMIXED=3.37519816066485e-05;ANNOT=(FBgn0262425|mir-288|10145 bp UPSTREAM),(FBgn0032868|CG17472|5950 bp DOWNSTREAM)	GT	.	1/1	0/0	1/1	1/1	1/1	1/1	1/1	.	1/1	0/0	1/1	1/1	0/0	0/0	0/0	1/1	1/1	1/1	1/1	1/1	0/0	1/1	1/1	0/0	.	1/1	0/0	0/0	0/0	1/1	1/1	0/0	1/1	0/0	0/0	1/1	1/1	1/1	.	0/0	0/0	.	1/1	.	0/0	1/1	.	.	0/0	0/0	.	.	0/0	0/0	0/0	0/0	1/1	0/0	0/0	0/0	0/0	1/1	1/1	0/0	1/1	0/0	1/1	0/0	1/1	.	1/1	0/0	0/0	1/1	0/0	0/0	0/0	1/1	1/1	0/0	0/0	1/1	1/1	0/0	0/0	.	1/1	1/1	0/0	0/0	1/1	0/0	0/0	1/1	0/0	0/0	1/1	1/1	0/0	0/0	1/1	1/1	1/1	1/1	0/0	1/1	0/0	0/0	1/1	0/0	1/1	.	1/1	1/1	0/0	0/0	1/1	0/0	1/1	1/1	1/1	1/1	1/1	1/1	1/1	1/1	.	.	1/1	0/0	0/0	1/1	.	1/1	1/1	0/0	.	1/1	0/0	0/0	0/0	0/0	0/0	1/1	1/1	1/1	0/0	0/0	0/0	0/0	.	.	1/1	1/1	.	1/1	.	0/0	1/1	1/1	.	0/0	1/1	0/0	1/1	1/1	1/1	0/0	0/0	1/1	1/1	0/0	0/0	1/1
2L	21914814	2L_21914814_SNP	C	A	.	.	T5EFF=0.2044;T5VAR=0.0400815651826753;T5VARACC=0.0123482724623041;T5PVAL=0.009704;T5MIXED=0.0250686832436696;T6EFF=0.667;T6VAR=0.120535928932063;T6VARACC=0.0549309484726371;T6PVAL=4.59e-06;T6MIXED=9.26715771085584e-06;AVGEFF=0.4357;AVGVAR=0.1043052444897;AVGVARACC=0.0525031693692079;AVGPVAL=2.196e-05;AVGMIXED=0.000102879935395861;DIFFEFF=-0.4626;DIFFVAR=0.0978845012382971;DIFFVARACC=0.0291743575924719;DIFFPVAL=4.06e-05;DIFFMIXED=4.40144207282109e-05;ANNOT=(FBgn0051600|CG31600|SYNONYMOUS_CODING)	GT	0/0	0/0	0/0	0/0	0/0	0/0	0/0	.	.	0/0	0/0	0/0	0/0	0/0	0/0	0/0	0/0	0/0	0/0	0/0	0/0	0/0	0/0	0/0	0/0	.	0/0	0/0	0/0	0/0	0/0	0/0	0/0	0/0	0/0	0/0	0/0	0/0	0/0	0/0	0/0	1/1	0/0	0/0	0/0	0/0	0/0	0/0	0/0	0/0	0/0	0/0	0/0	0/0	0/0	0/0	.	0/0	1/1	0/0	0/0	1/1	0/0	0/0	0/0	0/0	0/0	0/0	0/0	0/0	0/0	0/0	0/0	0/0	0/0	0/0	0/0	0/0	0/0	0/0	0/0	.	0/0	0/0	0/0	0/0	0/0	0/0	0/0	1/1	0/0	0/0	0/0	0/0	0/0	0/0	0/0	0/0	0/0	0/0	0/0	0/0	0/0	0/0	0/0	0/0	0/0	0/0	0/0	0/0	1/1	0/0	0/0	.	0/0	0/0	0/0	0/0	0/0	0/0	0/0	0/0	0/0	0/0	0/0	0/0	0/0	0/0	0/0	0/0	1/1	1/1	0/0	0/0	0/0	0/0	.	0/0	0/0	1/1	0/0	0/0	0/0	0/0	0/0	0/0	0/0	0/0	.	0/0	0/0	0/0	0/0	0/0	0/0	0/0	0/0	.	0/0	0/0	0/0	0/0	0/0	0/0	0/0	0/0	0/0	0/0	1/1	0/0	0/0	0/0	1/1	0/0	0/0
2L	22075281	2L_22075281_SNP	A	T	.	.	T5EFF=0.1939;T5VAR=0.039120929194213;T5VARACC=0.00909379815560027;T5PVAL=0.01064;T5MIXED=0.0387692666326226;T6EFF=0.6799;T6VAR=0.138438497298707;T6VARACC=0.0564503451069632;T6PVAL=7.975e-07;T6MIXED=3.02433096379051e-06;AVGEFF=0.4369;AVGVAR=0.115665289357725;AVGVARACC=0.0525153471696542;AVGPVAL=7.357e-06;AVGMIXED=9.75868488880164e-05;DIFFEFF=-0.486;DIFFVAR=0.118122020789404;DIFFVARACC=0.0528387590682843;DIFFPVAL=5.8e-06;DIFFMIXED=7.43565409976771e-06;ANNOT=(FBgn0051702|CG31702|2379 bp DOWNSTREAM),(FBgn0032971|ttm3|4391 bp DOWNSTREAM)	GT	0/0	0/0	0/0	0/0	0/0	0/0	0/0	0/0	0/0	0/0	0/0	0/0	0/0	1/1	0/0	0/0	0/0	0/0	0/0	0/0	0/0	0/0	0/0	0/0	1/1	.	0/0	0/0	0/0	0/0	0/0	0/0	.	0/0	0/0	0/0	0/0	0/0	0/0	0/0	0/0	0/0	0/0	0/0	0/0	0/0	0/0	0/0	0/0	1/1	0/0	0/0	0/0	0/0	0/0	0/0	0/0	0/0	1/1	0/0	0/0	1/1	0/0	0/0	0/0	0/0	0/0	0/0	0/0	0/0	0/0	0/0	0/0	0/0	0/0	0/0	0/0	0/0	0/0	0/0	0/0	0/0	0/0	0/0	0/0	0/0	0/0	0/0	0/0	1/1	0/0	0/0	0/0	0/0	0/0	0/0	0/0	0/0	0/0	0/0	0/0	0/0	0/0	0/0	0/0	0/0	0/0	0/0	0/0	0/0	1/1	0/0	1/1	.	0/0	0/0	0/0	0/0	0/0	0/0	0/0	0/0	0/0	0/0	0/0	0/0	0/0	0/0	0/0	0/0	.	1/1	0/0	0/0	0/0	0/0	.	0/0	0/0	1/1	0/0	0/0	0/0	1/1	0/0	0/0	0/0	0/0	0/0	0/0	0/0	.	0/0	0/0	0/0	0/0	0/0	.	0/0	0/0	0/0	0/0	0/0	0/0	0/0	0/0	0/0	0/0	0/0	0/0	0/0	0/0	.	.	0/0
2L	3457244	2L_3457244_SNP	C	T	.	.	T5EFF=-0.2652;T5VAR=0.120128860195941;T5VARACC=0.0815546672370495;T5PVAL=3.656e-06;T5MIXED=3.78380560359413e-06;T6EFF=-0.2429;T6VAR=0.0286373128259844;T6VARACC=0.0105357134265212;T6PVAL=0.02738;T6MIXED=0.0173516349843961;AVGEFF=-0.2541;AVGVAR=0.0632346010727843;AVGVARACC=0.042734558473211;AVGPVAL=0.0009402;AVGMIXED=0.000395675576042875;DIFFEFF=-0.02232;DIFFVAR=0.000412113902744662;DIFFVARACC=0.00440257222565665;DIFFPVAL=0.7927;DIFFMIXED=0.832112023614318;ANNOT=(FBgn0031537|sec5|85 bp UPSTREAM),(FBgn0031536|Cog3|186 bp UPSTREAM)	GT	0/0	0/0	0/0	1/1	0/0	0/0	1/1	1/1	0/0	1/1	0/0	0/0	0/0	0/0	0/0	0/0	0/0	0/0	0/0	0/0	0/0	0/0	0/0	0/0	0/0	0/0	0/0	0/0	0/0	1/1	0/0	0/0	1/1	1/1	1/1	0/0	0/0	1/1	0/0	1/1	0/0	0/0	1/1	0/0	0/0	0/0	0/0	0/0	0/0	0/0	0/0	0/0	0/0	0/0	0/0	0/0	0/0	0/0	0/0	0/0	0/0	0/0	0/0	0/0	0/0	0/0	0/0	0/0	0/0	0/0	0/0	0/0	0/0	0/0	0/0	0/0	1/1	1/1	0/0	0/0	0/0	0/0	0/0	0/0	0/0	0/0	0/0	0/0	0/0	0/0	.	0/0	0/0	0/0	0/0	0/0	0/0	0/0	0/0	.	0/0	1/1	0/0	0/0	1/1	0/0	0/0	0/0	0/0	0/0	0/0	1/1	0/0	0/0	0/0	0/0	0/0	0/0	0/0	0/0	0/0	0/0	0/0	1/1	0/0	0/0	0/0	0/0	0/0	0/0	0/0	0/0	0/0	0/0	0/0	0/0	0/0	0/0	0/0	0/0	0/0	0/0	0/0	0/0	0/0	0/0	0/0	1/1	0/0	0/0	0/0	0/0	0/0	0/0	0/0	.	0/0	0/0	0/0	1/1	.	.	0/0	0/0	0/0	1/1	0/0	0/0	0/0	0/0	0/0	0/0	0/0	0/0	0/0
2L	5303348	2L_5303348_SNP	A	G	.	.	T5EFF=-0.3837;T5VAR=0.120063187191524;T5VARACC=0.0683773029419955;T5PVAL=3.442e-06;T5MIXED=1.37809600270553e-05;T6EFF=-0.4889;T6VAR=0.056029339594597;T6VARACC=0.0191902097139563;T6PVAL=0.001827;T6MIXED=0.00198794375110765;AVGEFF=-0.4363;AVGVAR=0.089985667235534;AVGVARACC=0.0481685861728668;AVGPVAL=6.716e-05;AVGMIXED=0.000135125994639461;DIFFEFF=0.1052;DIFFVAR=0.00438530588631203;DIFFVARACC=0.000543656022875039;DIFFPVAL=0.3895;DIFFMIXED=0.388293120453458;ANNOT=(FBgn0016076|vri|INTRON)	GT	.	0/0	0/0	1/1	0/0	0/0	0/0	0/0	0/0	0/0	0/0	0/0	0/0	0/0	0/0	0/0	0/0	0/0	0/0	0/0	0/0	0/0	0/0	0/0	0/0	0/0	0/0	0/0	0/0	0/0	0/0	0/0	0/0	0/0	0/0	0/0	0/0	0/0	0/0	0/0	0/0	0/0	0/0	0/0	0/0	0/0	0/0	0/0	0/0	0/0	0/0	0/0	0/0	0/0	0/0	0/0	0/0	0/0	0/0	0/0	0/0	0/0	0/0	0/0	0/0	0/0	0/0	0/0	0/0	1/1	0/0	0/0	0/0	0/0	0/0	0/0	0/0	0/0	.	0/0	0/0	0/0	0/0	0/0	0/0	0/0	.	0/0	0/0	0/0	0/0	0/0	0/0	0/0	1/1	0/0	0/0	0/0	0/0	0/0	0/0	0/0	1/1	0/0	0/0	0/0	0/0	0/0	0/0	0/0	0/0	0/0	0/0	0/0	0/0	0/0	0/0	0/0	0/0	0/0	0/0	0/0	0/0	0/0	0/0	0/0	0/0	0/0	0/0	0/0	0/0	0/0	0/0	0/0	0/0	0/0	0/0	0/0	0/0	0/0	0/0	0/0	0/0	0/0	0/0	0/0	0/0	1/1	0/0	0/0	0/0	0/0	.	0/0	0/0	1/1	0/0	0/0	0/0	0/0	0/0	0/0	0/0	0/0	0/0	0/0	0/0	1/1	0/0	0/0	0/0	1/1	0/0	0/0	1/1
2L	5421039	2L_5421039_SNP	T	G	.	.	T5EFF=-0.3534;T5VAR=0.11228044575241;T5VARACC=0.0563485100681196;T5PVAL=6.595e-06;T5MIXED=6.04863126648796e-06;T6EFF=-0.3723;T6VAR=0.0361020549973617;T6VARACC=0.0110993826690275;T6PVAL=0.01228;T6MIXED=0.0105546332864325;AVGEFF=-0.3629;AVGVAR=0.0688572694026186;AVGVARACC=0.0425607113379945;AVGPVAL=0.0004872;AVGMIXED=0.000356964934811792;DIFFEFF=0.01894;DIFFVAR=0.000158962179084792;DIFFVARACC=0.00453518991892404;DIFFPVAL=0.8692;DIFFMIXED=0.853585289577761;ANNOT=(FBgn0016660|H15|5111 bp DOWNSTREAM),(FBgn0263866|CR43713|10803 bp DOWNSTREAM)	GT	0/0	0/0	0/0	0/0	0/0	0/0	0/0	0/0	.	1/1	0/0	0/0	0/0	0/0	0/0	0/0	0/0	0/0	0/0	0/0	0/0	0/0	0/0	0/0	0/0	0/0	0/0	0/0	0/0	0/0	1/1	0/0	0/0	0/0	0/0	0/0	0/0	0/0	0/0	.	0/0	0/0	0/0	0/0	0/0	0/0	0/0	0/0	0/0	0/0	0/0	0/0	0/0	1/1	0/0	0/0	0/0	1/1	0/0	1/1	0/0	0/0	0/0	0/0	1/1	0/0	0/0	0/0	0/0	0/0	0/0	0/0	0/0	0/0	0/0	0/0	0/0	0/0	0/0	0/0	0/0	0/0	0/0	0/0	0/0	0/0	0/0	0/0	0/0	0/0	0/0	0/0	0/0	0/0	0/0	0/0	0/0	0/0	0/0	0/0	0/0	0/0	0/0	0/0	0/0	0/0	0/0	0/0	0/0	0/0	0/0	0/0	0/0	0/0	0/0	0/0	0/0	0/0	0/0	0/0	0/0	0/0	0/0	0/0	0/0	0/0	0/0	0/0	0/0	0/0	0/0	0/0	0/0	0/0	0/0	0/0	0/0	0/0	0/0	0/0	0/0	0/0	0/0	0/0	0/0	0/0	0/0	1/1	0/0	0/0	0/0	0/0	0/0	0/0	0/0	1/1	0/0	0/0	0/0	0/0	0/0	0/0	0/0	0/0	0/0	0/0	0/0	1/1	0/0	0/0	0/0	0/0	0/0	1/1	0/0
2L	5880924	2L_5880924_SNP	G	A	.	.	T5EFF=-0.3917;T5VAR=0.138648864740328;T5VARACC=0.0880803672946016;T5PVAL=4.543e-07;T5MIXED=2.19783621320863e-06;T6EFF=-0.4653;T6VAR=0.0561952816343207;T6VARACC=0.0402779222479163;T6PVAL=0.001688;T6MIXED=0.0018562373528874;AVGEFF=-0.4285;AVGVAR=0.0952674891138426;AVGVARACC=0.0676430710196284;AVGPVAL=3.602e-05;AVGMIXED=6.29754776519935e-05;DIFFEFF=0.07354;DIFFVAR=0.00244864828220761;DIFFVARACC=4.85441509212592e-09;DIFFPVAL=0.5179;DIFFMIXED=0.524297868318411;ANNOT=(FBgn0031745|CG8965|1443 bp UPSTREAM),(FBgn0259959|Sfp26Ac|3426 bp UPSTREAM)	GT	0/0	0/0	0/0	1/1	0/0	0/0	0/0	0/0	0/0	0/0	0/0	0/0	0/0	0/0	0/0	0/0	0/0	0/0	0/0	0/0	0/0	0/0	0/0	0/0	0/0	0/0	0/0	0/0	0/0	0/0	0/0	0/0	0/0	0/0	0/0	0/0	0/0	0/0	0/0	0/0	0/0	0/0	0/0	0/0	0/0	0/0	0/0	0/0	0/0	0/0	0/0	0/0	0/0	0/0	0/0	0/0	0/0	0/0	0/0	0/0	0/0	0/0	0/0	0/0	0/0	0/0	0/0	0/0	0/0	0/0	0/0	0/0	0/0	0/0	0/0	0/0	0/0	0/0	0/0	0/0	0/0	0/0	0/0	0/0	0/0	0/0	0/0	0/0	0/0	0/0	0/0	0/0	0/0	0/0	0/0	0/0	0/0	0/0	0/0	0/0	0/0	0/0	0/0	0/0	0/0	0/0	0/0	0/0	0/0	1/1	0/0	0/0	0/0	0/0	0/0	0/0	0/0	0/0	0/0	0/0	0/0	1/1	0/0	1/1	0/0	0/0	0/0	0/0	1/1	0/0	0/0	0/0	0/0	0/0	0/0	0/0	0/0	1/1	0/0	0/0	0/0	0/0	0/0	0/0	0/0	0/0	0/0	1/1	0/0	0/0	0/0	0/0	0/0	0/0	0/0	1/1	0/0	0/0	0/0	0/0	0/0	0/0	.	.	0/0	1/1	0/0	1/1	0/0	0/0	0/0	0/0	0/0	0/0	0/0
2L	9465938	2L_9465938_SNP	C	T	.	.	T5EFF=-0.2573;T5VAR=0.120630719344102;T5VARACC=0.0555235754694589;T5PVAL=4.548e-06;T5MIXED=1.19124392985e-05;T6EFF=-0.3256;T6VAR=0.0549951790170074;T6VARACC=0.0142850953885997;T6PVAL=0.002357;T6MIXED=0.00359101975814391;AVGEFF=-0.2914;AVGVAR=0.089493127556985;AVGVARACC=0.0473036324120182;AVGPVAL=9.031e-05;AVGMIXED=0.000314956878217117;DIFFEFF=0.06835;DIFFVAR=0.00403821766620396;DIFFVARACC=0.00047294992848762;DIFFPVAL=0.416;DIFFMIXED=0.430536767605175;ANNOT=(FBgn0002973|numb|1754 bp DOWNSTREAM),(FBgn0264269|CR43769|4388 bp DOWNSTREAM)	GT	0/0	0/0	0/0	0/0	0/0	0/0	1/1	0/0	0/0	1/1	0/0	1/1	0/0	0/0	0/0	0/0	0/0	0/0	0/0	0/0	0/0	0/0	0/0	1/1	0/0	0/0	0/0	0/0	0/0	0/0	0/0	0/0	0/0	0/0	0/0	0/0	0/0	0/0	0/0	1/1	0/0	0/0	0/0	1/1	0/0	0/0	0/0	0/0	0/0	0/0	0/0	0/0	0/0	1/1	0/0	0/0	0/0	1/1	0/0	0/0	0/0	0/0	0/0	0/0	0/0	0/0	1/1	0/0	0/0	0/0	.	0/0	0/0	0/0	0/0	0/0	1/1	0/0	.	0/0	0/0	.	.	0/0	0/0	0/0	.	0/0	0/0	.	1/1	0/0	0/0	0/0	1/1	0/0	0/0	0/0	0/0	0/0	.	0/0	0/0	0/0	0/0	0/0	1/1	0/0	1/1	0/0	0/0	0/0	0/0	.	0/0	1/1	0/0	0/0	0/0	0/0	0/0	1/1	0/0	1/1	0/0	0/0	0/0	0/0	0/0	0/0	0/0	.	0/0	0/0	0/0	0/0	0/0	1/1	0/0	0/0	0/0	0/0	0/0	0/0	0/0	0/0	0/0	1/1	0/0	0/0	0/0	0/0	0/0	0/0	0/0	0/0	0/0	0/0	0/0	0/0	0/0	0/0	1/1	0/0	1/1	0/0	0/0	0/0	0/0	0/0	0/0	0/0	0/0	0/0	0/0
2L	9496975	2L_9496975_SNP	T	C	.	.	T5EFF=-0.1162;T5VAR=0.0541504024527803;T5VARACC=0.0195958974899724;T5PVAL=0.003571;T5MIXED=0.00752863865509707;T6EFF=-0.3337;T6VAR=0.126632283131057;T6VARACC=0.023390345741134;T6PVAL=5.516e-06;T6MIXED=3.76369815230992e-06;AVGEFF=-0.2249;AVGVAR=0.116311340278733;AVGVARACC=0.0316083252150107;AVGPVAL=1.409e-05;AVGMIXED=2.37092326390502e-05;DIFFEFF=0.2175;DIFFVAR=0.0914810912184986;DIFFVARACC=0.022965993917659;DIFFPVAL=0.0001307;DIFFMIXED=8.87595266633233e-05;ANNOT=(FBgn0032120|CG33298|INTRON)	GT	1/1	0/0	.	0/0	1/1	1/1	1/1	1/1	1/1	0/0	1/1	1/1	0/0	0/0	.	1/1	0/0	0/0	0/0	0/0	1/1	0/0	0/0	1/1	0/0	1/1	1/1	0/0	1/1	1/1	0/0	1/1	.	0/0	0/0	1/1	0/0	1/1	1/1	1/1	0/0	0/0	1/1	1/1	1/1	.	1/1	.	1/1	0/0	1/1	.	0/0	1/1	1/1	1/1	0/0	1/1	0/0	0/0	0/0	0/0	1/1	0/0	1/1	1/1	.	0/0	1/1	.	.	0/0	0/0	0/0	1/1	0/0	1/1	0/0	1/1	1/1	0/0	.	1/1	0/0	.	1/1	0/0	.	1/1	1/1	.	1/1	1/1	.	.	0/0	1/1	0/0	1/1	1/1	0/0	0/0	1/1	0/0	0/0	1/1	1/1	0/0	1/1	0/0	0/0	0/0	0/0	.	1/1	1/1	0/0	.	0/0	0/0	0/0	1/1	1/1	1/1	1/1	1/1	0/0	0/0	1/1	0/0	0/0	0/0	0/0	0/0	1/1	.	1/1	0/0	0/0	0/0	0/0	1/1	0/0	0/0	0/0	1/1	0/0	1/1	0/0	1/1	1/1	1/1	1/1	0/0	1/1	.	0/0	1/1	0/0	0/0	0/0	.	0/0	1/1	0/0	1/1	1/1	0/0	0/0	0/0	0/0	0/0	0/0	0/0	1/1
2R	10514639	2R_10514639_SNP	T	G	.	.	T5EFF=-0.3569;T5VAR=0.114934487121637;T5VARACC=0.0787302937442689;T5PVAL=6.117e-06;T5MIXED=3.22948890413598e-05;T6EFF=-0.4339;T6VAR=0.0506768692667484;T6VARACC=0.0163218259930313;T6PVAL=0.003163;T6MIXED=0.00468115729935621;AVGEFF=-0.3954;AVGVAR=0.0836313660754928;AVGVARACC=0.0434874653417001;AVGPVAL=0.0001309;AVGMIXED=0.000424905258408081;DIFFEFF=0.07702;DIFFVAR=0.00273508271031146;DIFFVARACC=0.00192591841518397;DIFFPVAL=0.4982;DIFFMIXED=0.505655973016306;ANNOT=(FBgn0083123|Uhg5|2626 bp DOWNSTREAM),(FBgn0033949|CG10131|2779 bp DOWNSTREAM)	GT	0/0	0/0	0/0	1/1	0/0	.	.	1/1	0/0	0/0	0/0	1/1	0/0	0/0	0/0	1/1	0/0	0/0	0/0	1/1	0/0	0/0	0/0	0/0	0/0	0/0	0/0	0/0	0/0	0/0	0/0	0/0	0/0	0/0	0/0	0/0	0/0	0/0	0/0	0/0	0/0	0/0	0/0	0/0	0/0	0/0	0/0	0/0	0/0	0/0	0/0	0/0	0/0	0/0	0/0	0/0	0/0	0/0	0/0	0/0	0/0	0/0	0/0	0/0	1/1	0/0	0/0	0/0	0/0	0/0	0/0	0/0	0/0	0/0	0/0	0/0	0/0	0/0	0/0	0/0	0/0	0/0	0/0	1/1	0/0	0/0	0/0	0/0	0/0	0/0	0/0	0/0	0/0	0/0	1/1	0/0	0/0	0/0	0/0	0/0	.	0/0	0/0	0/0	0/0	0/0	0/0	0/0	0/0	0/0	0/0	.	0/0	0/0	0/0	0/0	0/0	0/0	0/0	0/0	0/0	0/0	0/0	0/0	0/0	0/0	0/0	0/0	0/0	0/0	0/0	0/0	0/0	0/0	0/0	0/0	.	0/0	0/0	0/0	0/0	0/0	0/0	0/0	0/0	0/0	0/0	0/0	0/0	0/0	0/0	0/0	0/0	0/0	0/0	0/0	0/0	0/0	0/0	0/0	0/0	0/0	0/0	0/0	1/1	1/1	0/0	0/0	0/0	0/0	0/0	0/0	0/0	0/0	0/0
2R	12076924	2R_12076924_SNP	G	A	.	.	T5EFF=0.02435;T5VAR=0.00121680687140631;T5VARACC=0.00415432059088139;T5PVAL=0.6516;T5MIXED=0.756744067573577;T6EFF=-0.3643;T6VAR=0.0784093872080174;T6VARACC=0.0413266661647083;T6PVAL=0.000217;T6MIXED=0.000237357015534881;AVGEFF=-0.17;AVGVAR=0.0344051671065848;AVGVARACC=0.0218625486164362;AVGPVAL=0.01545;AVGMIXED=0.015435347164674;DIFFEFF=0.3886;DIFFVAR=0.150892854495208;DIFFVARACC=0.0673043069443952;DIFFPVAL=1.655e-07;DIFFMIXED=1.56826487439071e-07;ANNOT=(FBgn0034087|clu|SYNONYMOUS_CODING)	GT	0/0	1/1	0/0	1/1	0/0	0/0	0/0	0/0	1/1	0/0	0/0	1/1	0/0	0/0	0/0	0/0	1/1	0/0	0/0	0/0	0/0	1/1	0/0	1/1	0/0	0/0	0/0	0/0	1/1	0/0	0/0	0/0	0/0	0/0	0/0	1/1	0/0	1/1	0/0	0/0	1/1	0/0	0/0	0/0	.	0/0	0/0	0/0	0/0	0/0	0/0	0/0	0/0	0/0	0/0	0/0	0/0	0/0	0/0	0/0	0/0	0/0	0/0	0/0	0/0	0/0	.	0/0	1/1	.	0/0	0/0	0/0	0/0	1/1	0/0	0/0	0/0	0/0	0/0	0/0	0/0	1/1	0/0	0/0	0/0	0/0	1/1	0/0	0/0	0/0	0/0	0/0	0/0	0/0	0/0	1/1	0/0	0/0	0/0	.	0/0	0/0	0/0	0/0	0/0	0/0	0/0	0/0	0/0	0/0	0/0	0/0	0/0	0/0	0/0	0/0	1/1	0/0	0/0	1/1	0/0	0/0	0/0	0/0	1/1	0/0	0/0	0/0	0/0	0/0	.	0/0	0/0	0/0	0/0	0/0	0/0	1/1	0/0	0/0	0/0	0/0	0/0	0/0	0/0	0/0	0/0	0/0	0/0	0/0	0/0	0/0	1/1	0/0	0/0	0/0	0/0	0/0	0/0	0/0	1/1	1/1	1/1	1/1	0/0	0/0	0/0	0/0	0/0	0/0	0/0	0/0	0/0	0/0
2R	12076960	2R_12076960_SNP	A	C	.	.	T5EFF=-0.01379;T5VAR=0.000426887988883986;T5VARACC=0.00135437148607498;T5PVAL=0.7898;T5MIXED=0.717533337073225;T6EFF=-0.3885;T6VAR=0.0966447420280534;T6VARACC=0.0446812343252084;T6PVAL=3.892e-05;T6MIXED=4.77510952365191e-05;AVGEFF=-0.2012;AVGVAR=0.0525673809497001;AVGVARACC=0.0238018523069408;AVGPVAL=0.002713;AVGMIXED=0.00312896245443543;DIFFEFF=0.3747;DIFFVAR=0.149999710883442;DIFFVARACC=0.0618570351360819;DIFFPVAL=1.972e-07;DIFFMIXED=1.81523753394132e-07;ANNOT=(FBgn0034087|clu|SYNONYMOUS_CODING)	GT	0/0	1/1	0/0	1/1	0/0	0/0	0/0	0/0	1/1	1/1	0/0	1/1	0/0	0/0	0/0	0/0	1/1	0/0	0/0	0/0	0/0	1/1	0/0	1/1	0/0	0/0	0/0	0/0	1/1	0/0	0/0	0/0	0/0	0/0	0/0	1/1	0/0	1/1	0/0	0/0	1/1	0/0	0/0	0/0	.	0/0	0/0	0/0	0/0	0/0	0/0	0/0	0/0	0/0	0/0	0/0	0/0	0/0	0/0	0/0	0/0	0/0	0/0	0/0	0/0	0/0	.	0/0	1/1	1/1	0/0	0/0	0/0	0/0	1/1	0/0	0/0	0/0	0/0	0/0	0/0	0/0	1/1	0/0	0/0	0/0	0/0	1/1	0/0	0/0	0/0	0/0	0/0	0/0	0/0	0/0	1/1	0/0	0/0	0/0	.	0/0	0/0	0/0	0/0	0/0	0/0	0/0	0/0	0/0	0/0	0/0	0/0	1/1	0/0	0/0	0/0	1/1	0/0	0/0	1/1	0/0	0/0	0/0	0/0	1/1	0/0	0/0	0/0	0/0	0/0	.	0/0	0/0	0/0	0/0	.	0/0	1/1	0/0	0/0	0/0	0/0	0/0	0/0	0/0	0/0	0/0	0/0	0/0	0/0	0/0	0/0	1/1	0/0	0/0	0/0	0/0	0/0	0/0	0/0	1/1	1/1	1/1	.	0/0	0/0	0/0	0/0	0/0	0/0	0/0	0/0	0/0	0/0
2R	12305000	2R_12305000_SNP	G	A	.	.	T5EFF=-0.3111;T5VAR=0.112207914000067;T5VARACC=0.0533473100517114;T5PVAL=8.007e-06;T5MIXED=2.80592314500714e-05;T6EFF=-0.3837;T6VAR=0.0488820999755623;T6VARACC=0.016241901847627;T6PVAL=0.003763;T6MIXED=0.00488714375904161;AVGEFF=-0.3474;AVGVAR=0.0810270161945039;AVGVARACC=0.0432253428864511;AVGPVAL=0.0001685;AVGMIXED=0.000416718740706491;DIFFEFF=0.07264;DIFFVAR=0.00294580959465377;DIFFVARACC=4.53797365529973e-05;DIFFPVAL=0.4821;DIFFMIXED=0.490034195572212;ANNOT=(FBgn0029154|Menl-1|SYNONYMOUS_CODING),(FBgn0029153|Menl-2|START_GAINED),(FBgn0264273|Sema-2b|INTRON)	GT	0/0	0/0	0/0	0/0	0/0	0/0	0/0	0/0	0/0	1/1	0/0	0/0	0/0	0/0	0/0	0/0	0/0	0/0	0/0	0/0	1/1	0/0	0/0	1/1	0/0	0/0	1/1	0/0	0/0	0/0	0/0	0/0	0/0	0/0	0/0	0/0	0/0	0/0	0/0	0/0	0/0	1/1	0/0	0/0	0/0	0/0	0/0	0/0	0/0	0/0	0/0	0/0	0/0	0/0	0/0	0/0	0/0	0/0	0/0	0/0	0/0	0/0	0/0	0/0	0/0	.	0/0	0/0	0/0	0/0	0/0	0/0	0/0	0/0	0/0	.	0/0	0/0	0/0	0/0	0/0	0/0	1/1	0/0	0/0	0/0	0/0	0/0	0/0	0/0	0/0	0/0	.	0/0	0/0	0/0	0/0	0/0	0/0	0/0	.	0/0	0/0	0/0	0/0	0/0	0/0	0/0	1/1	0/0	0/0	0/0	0/0	1/1	0/0	0/0	0/0	0/0	0/0	0/0	0/0	0/0	0/0	0/0	1/1	0/0	0/0	0/0	0/0	0/0	0/0	0/0	0/0	0/0	0/0	.	0/0	0/0	0/0	0/0	0/0	0/0	0/0	0/0	0/0	0/0	0/0	1/1	0/0	0/0	0/0	0/0	0/0	0/0	0/0	0/0	0/0	0/0	0/0	0/0	0/0	0/0	0/0	1/1	0/0	0/0	0/0	0/0	0/0	0/0	0/0	1/1	0/0	0/0	1/1
2R	14221079	2R_14221079_SNP	G	C	.	.	T5EFF=-0.3224;T5VAR=0.112989734393852;T5VARACC=0.0610645084285019;T5PVAL=8.947e-06;T5MIXED=5.30469834861916e-05;T6EFF=-0.3359;T6VAR=0.0348795094729804;T6VARACC=0.0285778676297551;T6PVAL=0.01566;T6MIXED=0.0204315018033278;AVGEFF=-0.3292;AVGVAR=0.0678317774865767;AVGVARACC=0.0510739606659091;AVGPVAL=0.0006756;AVGMIXED=0.00205343985201735;DIFFEFF=0.01345;DIFFVAR=9.42428756224088e-05;DIFFVARACC=0.000445387084600124;DIFFPVAL=0.9009;DIFFMIXED=0.909559956977182;ANNOT=(FBgn0010575|sbb|INTRON)	GT	0/0	0/0	0/0	0/0	0/0	0/0	0/0	0/0	0/0	1/1	0/0	0/0	0/0	0/0	0/0	1/1	0/0	0/0	0/0	0/0	0/0	1/1	0/0	0/0	0/0	.	1/1	0/0	0/0	1/1	0/0	0/0	0/0	0/0	0/0	0/0	0/0	0/0	0/0	0/0	0/0	0/0	0/0	0/0	.	1/1	0/0	.	.	0/0	0/0	0/0	0/0	0/0	0/0	0/0	0/0	0/0	0/0	0/0	0/0	0/0	0/0	0/0	0/0	0/0	.	0/0	0/0	0/0	0/0	0/0	1/1	0/0	0/0	0/0	0/0	0/0	0/0	1/1	0/0	0/0	0/0	0/0	0/0	0/0	0/0	0/0	0/0	0/0	0/0	0/0	0/0	0/0	0/0	0/0	0/0	0/0	0/0	0/0	.	0/0	0/0	0/0	0/0	0/0	0/0	0/0	0/0	0/0	0/0	0/0	0/0	0/0	0/0	0/0	0/0	0/0	0/0	0/0	0/0	0/0	0/0	0/0	0/0	1/1	0/0	0/0	0/0	0/0	0/0	0/0	0/0	0/0	0/0	0/0	0/0	0/0	0/0	0/0	0/0	0/0	0/0	0/0	0/0	.	0/0	1/1	0/0	0/0	0/0	0/0	0/0	0/0	0/0	0/0	0/0	.	0/0	0/0	0/0	0/0	0/0	0/0	0/0	1/1	0/0	0/0	0/0	0/0	0/0	1/1	0/0	0/0	0/0
2R	14492459	2R_14492459_SNP	T	A	.	.	T5EFF=-0.22;T5VAR=0.124521232488655;T5VARACC=0.0547020375916364;T5PVAL=3.34e-06;T5MIXED=1.02380028338637e-05;T6EFF=-0.2761;T6VAR=0.0582830279712014;T6VARACC=0.0155098603368076;T6PVAL=0.001785;T6MIXED=0.0023046722416772;AVGEFF=-0.248;AVGVAR=0.094099897884065;AVGVARACC=0.0433469508887743;AVGPVAL=6.137e-05;AVGMIXED=0.000156150945579597;DIFFEFF=0.05611;DIFFVAR=0.00404693823047403;DIFFVARACC=1.93236298691724e-05;DIFFPVAL=0.4169;DIFFMIXED=0.423301077547949;ANNOT=(FBgn0050115|GEFmeso|INTRON)	GT	0/0	0/0	0/0	1/1	0/0	0/0	.	0/0	1/1	1/1	0/0	0/0	0/0	0/0	0/0	0/0	0/0	0/0	0/0	0/0	0/0	0/0	0/0	0/0	0/0	.	0/0	0/0	1/1	0/0	0/0	0/0	0/0	1/1	0/0	0/0	1/1	1/1	0/0	0/0	0/0	0/0	.	0/0	0/0	0/0	0/0	0/0	0/0	0/0	0/0	0/0	0/0	1/1	0/0	1/1	0/0	1/1	1/1	0/0	0/0	0/0	0/0	0/0	1/1	0/0	.	0/0	0/0	1/1	1/1	0/0	0/0	.	0/0	0/0	1/1	.	0/0	0/0	0/0	.	1/1	0/0	0/0	1/1	0/0	0/0	0/0	0/0	1/1	0/0	0/0	0/0	1/1	0/0	0/0	0/0	1/1	0/0	.	.	0/0	0/0	0/0	0/0	0/0	0/0	1/1	1/1	0/0	0/0	0/0	0/0	0/0	0/0	0/0	0/0	0/0	0/0	0/0	0/0	0/0	0/0	1/1	0/0	0/0	0/0	0/0	0/0	0/0	0/0	0/0	0/0	1/1	0/0	1/1	1/1	0/0	0/0	0/0	1/1	0/0	0/0	0/0	0/0	0/0	1/1	0/0	0/0	0/0	0/0	0/0	0/0	0/0	0/0	0/0	.	0/0	0/0	0/0	1/1	0/0	0/0	0/0	1/1	0/0	0/0	1/1	0/0	0/0	1/1	0/0	0/0	0/0
2R	14558686	2R_14558686_SNP	T	C	.	.	T5EFF=-0.3364;T5VAR=0.112917834891958;T5VARACC=0.0816310160656971;T5PVAL=6.586e-06;T5MIXED=1.37290429586994e-05;T6EFF=-0.5076;T6VAR=0.0721209343178146;T6VARACC=0.0374678193429621;T6PVAL=0.000368;T6MIXED=0.00040457301389064;AVGEFF=-0.422;AVGVAR=0.101373397842672;AVGVARACC=0.0681868644253755;AVGPVAL=2.076e-05;AVGMIXED=4.06620348048617e-05;DIFFEFF=0.1712;DIFFVAR=0.0138065823053977;DIFFVARACC=0.00131700705746046;DIFFPVAL=0.1248;DIFFMIXED=0.122016057299679;ANNOT=(FBgn0023214|edl|INTRON)	GT	0/0	0/0	0/0	0/0	0/0	1/1	0/0	0/0	1/1	1/1	0/0	0/0	0/0	0/0	0/0	0/0	0/0	0/0	0/0	0/0	0/0	0/0	0/0	0/0	0/0	0/0	0/0	0/0	0/0	0/0	0/0	1/1	0/0	0/0	0/0	0/0	0/0	0/0	0/0	0/0	0/0	0/0	0/0	0/0	0/0	0/0	0/0	0/0	0/0	0/0	0/0	0/0	0/0	0/0	0/0	0/0	0/0	0/0	0/0	0/0	0/0	0/0	0/0	0/0	1/1	0/0	0/0	0/0	0/0	0/0	0/0	0/0	0/0	0/0	0/0	0/0	0/0	0/0	0/0	0/0	0/0	0/0	0/0	0/0	0/0	0/0	0/0	0/0	0/0	0/0	.	0/0	0/0	0/0	0/0	0/0	0/0	0/0	.	0/0	0/0	0/0	0/0	0/0	0/0	0/0	0/0	0/0	0/0	0/0	0/0	0/0	0/0	1/1	0/0	0/0	0/0	0/0	0/0	0/0	0/0	0/0	0/0	0/0	0/0	0/0	0/0	0/0	0/0	0/0	0/0	0/0	0/0	0/0	0/0	0/0	0/0	0/0	1/1	0/0	0/0	0/0	0/0	0/0	0/0	0/0	1/1	1/1	1/1	0/0	0/0	0/0	0/0	0/0	0/0	0/0	0/0	.	0/0	0/0	0/0	0/0	0/0	0/0	0/0	1/1	0/0	0/0	0/0	0/0	0/0	0/0	0/0	0/0	0/0
2R	15490188	2R_15490188_SNP	G	A	.	.	T5EFF=-0.2298;T5VAR=0.113921409130417;T5VARACC=0.0698953862933744;T5PVAL=7.202e-06;T5MIXED=1.47578355129186e-05;T6EFF=-0.4001;T6VAR=0.100056964739688;T6VARACC=0.0500389547110152;T6PVAL=2.793e-05;T6MIXED=3.50376363529826e-05;AVGEFF=-0.315;AVGVAR=0.124973766644446;AVGVARACC=0.0898579820367688;AVGPVAL=2.42e-06;AVGMIXED=4.47702421230168e-06;DIFFEFF=0.1703;DIFFVAR=0.0304729134066963;DIFFVARACC=0.00692921065783919;DIFFPVAL=0.02321;DIFFMIXED=0.0235321591775828;ANNOT=(FBgn0003435|sm|INTRON)	GT	0/0	0/0	0/0	0/0	0/0	1/1	0/0	1/1	0/0	0/0	1/1	0/0	0/0	0/0	0/0	0/0	0/0	0/0	0/0	0/0	1/1	0/0	0/0	1/1	0/0	.	0/0	0/0	0/0	0/0	0/0	1/1	0/0	0/0	0/0	0/0	0/0	0/0	0/0	1/1	0/0	0/0	0/0	0/0	0/0	0/0	0/0	0/0	0/0	0/0	0/0	0/0	0/0	1/1	0/0	0/0	1/1	0/0	0/0	0/0	0/0	0/0	0/0	0/0	0/0	0/0	0/0	0/0	0/0	0/0	0/0	0/0	0/0	0/0	.	.	0/0	0/0	0/0	0/0	0/0	.	0/0	0/0	0/0	0/0	0/0	0/0	1/1	0/0	0/0	0/0	0/0	0/0	0/0	0/0	0/0	0/0	0/0	1/1	.	0/0	0/0	0/0	0/0	1/1	0/0	0/0	1/1	0/0	0/0	0/0	0/0	0/0	0/0	0/0	1/1	0/0	0/0	0/0	0/0	0/0	1/1	1/1	1/1	0/0	0/0	0/0	0/0	0/0	0/0	0/0	0/0	0/0	0/0	0/0	0/0	1/1	0/0	0/0	0/0	0/0	0/0	0/0	0/0	0/0	0/0	1/1	0/0	1/1	0/0	0/0	0/0	1/1	0/0	1/1	0/0	0/0	.	0/0	0/0	0/0	0/0	0/0	1/1	1/1	1/1	1/1	0/0	0/0	0/0	0/0	0/0	0/0	0/0
2R	16412807	2R_16412807_SNP	C	A	.	.	T5EFF=0.02747;T5VAR=0.00217636748176849;T5VARACC=0.000404830898600494;T5PVAL=0.5422;T5MIXED=0.442885117520274;T6EFF=0.3076;T6VAR=0.0774024092736151;T6VARACC=0.0289793618897731;T6PVAL=0.0002104;T6MIXED=0.000239812928980353;AVGEFF=0.1676;AVGVAR=0.0466790774162992;AVGVARACC=0.0232169550920584;AVGPVAL=0.004305;AVGMIXED=0.00450144258637202;DIFFEFF=-0.2802;DIFFVAR=0.107046275884169;DIFFVARACC=0.0404601750322564;DIFFPVAL=1.114e-05;DIFFMIXED=9.92939167865142e-06;ANNOT=(FBgn0034510|CG13426|12133 bp UPSTREAM),(FBgn0034511|CG13422|1025 bp DOWNSTREAM)	GT	0/0	0/0	0/0	0/0	0/0	0/0	0/0	0/0	0/0	1/1	0/0	0/0	0/0	1/1	0/0	0/0	0/0	0/0	1/1	1/1	1/1	0/0	0/0	0/0	0/0	0/0	0/0	0/0	0/0	0/0	0/0	0/0	0/0	0/0	0/0	0/0	.	0/0	0/0	0/0	0/0	1/1	.	0/0	0/0	0/0	0/0	0/0	0/0	1/1	0/0	0/0	1/1	0/0	0/0	0/0	0/0	0/0	1/1	1/1	0/0	1/1	0/0	1/1	0/0	0/0	0/0	0/0	1/1	0/0	1/1	1/1	1/1	1/1	0/0	0/0	1/1	0/0	0/0	0/0	0/0	0/0	0/0	1/1	0/0	0/0	0/0	0/0	0/0	1/1	0/0	0/0	0/0	0/0	1/1	1/1	0/0	0/0	0/0	0/0	0/0	0/0	0/0	0/0	1/1	0/0	0/0	0/0	0/0	1/1	0/0	0/0	0/0	0/0	0/0	1/1	0/0	0/0	0/0	0/0	0/0	0/0	0/0	0/0	0/0	0/0	1/1	1/1	1/1	1/1	0/0	1/1	0/0	0/0	0/0	0/0	0/0	0/0	1/1	0/0	0/0	0/0	0/0	0/0	0/0	0/0	0/0	0/0	0/0	1/1	1/1	0/0	0/0	0/0	0/0	0/0	1/1	0/0	0/0	0/0	1/1	0/0	0/0	0/0	0/0	1/1	0/0	1/1	0/0	0/0	0/0	0/0	1/1	0/0	0/0
2R	16793853	2R_16793853_SNP	C	G	.	.	T5EFF=-0.1942;T5VAR=0.119661997434252;T5VARACC=0.0963388733228823;T5PVAL=4.996e-06;T5MIXED=5.10344287319222e-06;T6EFF=-0.2261;T6VAR=0.046488013154808;T6VARACC=0.0214321166041734;T6PVAL=0.005273;T6MIXED=0.0034902745955898;AVGEFF=-0.2102;AVGVAR=0.0815875607088406;AVGVARACC=0.0673276225729872;AVGPVAL=0.0001912;AVGMIXED=9.74906079547696e-05;DIFFEFF=0.03186;DIFFVAR=0.00152764345791518;DIFFVARACC=7.54512001710506e-07;DIFFPVAL=0.6171;DIFFMIXED=0.577632013880463;ANNOT=(FBgn0015524|otp|2953 bp UPSTREAM),(FBgn0034560|CG9235|3356 bp DOWNSTREAM)	GT	0/0	0/0	1/1	1/1	0/0	0/0	0/0	0/0	0/0	0/0	0/0	0/0	0/0	0/0	0/0	1/1	0/0	0/0	0/0	0/0	0/0	1/1	1/1	1/1	0/0	1/1	0/0	0/0	0/0	0/0	1/1	1/1	0/0	0/0	0/0	0/0	0/0	0/0	0/0	0/0	1/1	.	.	0/0	.	1/1	0/0	0/0	0/0	0/0	0/0	.	0/0	0/0	0/0	0/0	1/1	0/0	0/0	0/0	0/0	1/1	1/1	0/0	0/0	0/0	0/0	0/0	0/0	0/0	0/0	0/0	0/0	0/0	0/0	1/1	1/1	.	0/0	0/0	0/0	0/0	0/0	1/1	0/0	0/0	1/1	0/0	0/0	0/0	1/1	0/0	0/0	0/0	0/0	0/0	1/1	1/1	.	1/1	.	0/0	.	0/0	1/1	0/0	1/1	0/0	1/1	0/0	1/1	0/0	1/1	1/1	1/1	0/0	0/0	0/0	0/0	0/0	0/0	0/0	0/0	0/0	1/1	0/0	0/0	0/0	0/0	0/0	0/0	0/0	1/1	0/0	0/0	0/0	0/0	0/0	0/0	0/0	0/0	0/0	0/0	1/1	0/0	0/0	0/0	1/1	0/0	1/1	.	1/1	0/0	1/1	0/0	1/1	0/0	0/0	1/1	1/1	0/0	0/0	0/0	0/0	1/1	0/0	0/0	1/1	0/0	0/0	0/0	1/1	0/0	0/0	1/1
2R	17790450	2R_17790450_SNP	A	G	.	.	T5EFF=0.1509;T5VAR=0.0513564637346813;T5VARACC=0.0235293383444439;T5PVAL=0.00323;T5MIXED=0.0052823934541865;T6EFF=0.4205;T6VAR=0.113804308231682;T6VARACC=0.0697420801681768;T6PVAL=8.267e-06;T6MIXED=1.15192525858178e-05;AVGEFF=0.2857;AVGVAR=0.105920692407837;AVGVARACC=0.0726760240413391;AVGPVAL=1.774e-05;AVGMIXED=5.67519092177628e-05;DIFFEFF=-0.2695;DIFFVAR=0.0796841745799053;DIFFVARACC=0.0696845908335993;DIFFPVAL=0.0002191;DIFFMIXED=0.000201429998898027;ANNOT=(FBgn0085397|Fili|INTRON)	GT	0/0	0/0	0/0	0/0	1/1	0/0	0/0	0/0	0/0	0/0	0/0	0/0	0/0	1/1	1/1	0/0	0/0	1/1	0/0	0/0	0/0	0/0	0/0	0/0	1/1	0/0	0/0	0/0	0/0	0/0	0/0	0/0	0/0	1/1	0/0	0/0	1/1	0/0	0/0	0/0	0/0	0/0	0/0	1/1	.	1/1	0/0	.	.	1/1	0/0	0/0	0/0	.	0/0	0/0	1/1	0/0	1/1	0/0	0/0	0/0	0/0	0/0	0/0	0/0	0/0	0/0	0/0	0/0	1/1	0/0	0/0	1/1	0/0	0/0	0/0	1/1	0/0	0/0	0/0	0/0	0/0	0/0	0/0	0/0	0/0	0/0	0/0	1/1	0/0	0/0	0/0	0/0	0/0	1/1	1/1	0/0	0/0	0/0	0/0	0/0	0/0	0/0	0/0	0/0	0/0	0/0	0/0	0/0	0/0	0/0	0/0	0/0	0/0	.	0/0	0/0	0/0	0/0	0/0	0/0	0/0	0/0	0/0	1/1	1/1	0/0	0/0	0/0	0/0	0/0	0/0	1/1	0/0	1/1	0/0	0/0	0/0	1/1	0/0	0/0	0/0	0/0	1/1	.	0/0	0/0	0/0	0/0	1/1	0/0	0/0	0/0	0/0	0/0	0/0	.	0/0	0/0	1/1	0/0	0/0	0/0	0/0	0/0	0/0	0/0	1/1	0/0	.	0/0	1/1	0/0	0/0
2R	17938981	2R_17938981_SNP	G	A	.	.	T5EFF=-0.1641;T5VAR=0.0956716866114743;T5VARACC=0.0679578867819445;T5PVAL=5.015e-05;T5MIXED=3.83927721849836e-06;T6EFF=-0.166;T6VAR=0.0281506161768171;T6VARACC=0.0128199750019575;T6PVAL=0.03072;T6MIXED=0.0260156476775253;AVGEFF=-0.165;AVGVAR=0.0556790668250864;AVGVARACC=0.0305973303521562;AVGPVAL=0.002209;AVGMIXED=0.0011281405016444;DIFFEFF=0.001893;DIFFVAR=6.36199176337272e-06;DIFFVARACC=0.00180390399144714;DIFFPVAL=0.9743;DIFFMIXED=0.986702977857205;ANNOT=(FBgn0005778|PpD5|8594 bp UPSTREAM),(FBgn0034683|CG13500|4991 bp UPSTREAM)	GT	0/0	0/0	0/0	1/1	1/1	0/0	1/1	0/0	0/0	1/1	0/0	0/0	1/1	0/0	1/1	1/1	1/1	0/0	0/0	0/0	0/0	.	0/0	1/1	0/0	0/0	1/1	1/1	0/0	0/0	0/0	1/1	0/0	0/0	0/0	0/0	.	1/1	0/0	0/0	0/0	0/0	1/1	0/0	.	0/0	0/0	.	0/0	1/1	0/0	0/0	1/1	0/0	1/1	1/1	1/1	0/0	1/1	0/0	1/1	0/0	1/1	0/0	1/1	0/0	0/0	0/0	0/0	.	1/1	1/1	0/0	0/0	1/1	0/0	1/1	0/0	0/0	0/0	0/0	.	0/0	0/0	0/0	0/0	1/1	1/1	0/0	0/0	0/0	0/0	0/0	0/0	1/1	0/0	0/0	0/0	0/0	0/0	0/0	1/1	1/1	1/1	0/0	1/1	1/1	0/0	1/1	1/1	0/0	0/0	0/0	0/0	0/0	0/0	0/0	0/0	1/1	1/1	.	1/1	0/0	0/0	1/1	0/0	0/0	1/1	1/1	1/1	1/1	1/1	0/0	0/0	1/1	0/0	0/0	1/1	0/0	0/0	0/0	1/1	0/0	0/0	0/0	.	1/1	0/0	0/0	0/0	0/0	0/0	.	0/0	0/0	1/1	0/0	0/0	0/0	0/0	0/0	0/0	0/0	0/0	1/1	1/1	1/1	0/0	0/0	0/0	0/0	0/0	0/0	1/1	0/0
2R	18700305	2R_18700305_SNP	G	C	.	.	T5EFF=-0.263;T5VAR=0.118944607259435;T5VARACC=0.0631653951421881;T5PVAL=4.112e-06;T5MIXED=5.15917944692214e-05;T6EFF=-0.324;T6VAR=0.0507671893642524;T6VARACC=0.0195609135452796;T6PVAL=0.003135;T6MIXED=0.00375037795258494;AVGEFF=-0.2935;AVGVAR=0.0843022539019924;AVGVARACC=0.0535849431933587;AVGPVAL=0.0001226;AVGMIXED=0.000386894926757992;DIFFEFF=0.06096;DIFFVAR=0.00306882168420329;DIFFVARACC=0.000860446158045425;DIFFPVAL=0.4731;DIFFMIXED=0.46729099046231;ANNOT=(FBgn0086408|stl|SYNONYMOUS_CODING)	GT	0/0	0/0	0/0	1/1	0/0	0/0	0/0	1/1	0/0	0/0	1/1	0/0	0/0	0/0	0/0	0/0	0/0	0/0	0/0	0/0	0/0	0/0	0/0	0/0	.	0/0	0/0	0/0	0/0	0/0	0/0	0/0	0/0	0/0	0/0	0/0	0/0	0/0	0/0	0/0	0/0	0/0	0/0	1/1	0/0	0/0	0/0	0/0	0/0	0/0	.	0/0	0/0	0/0	0/0	0/0	0/0	0/0	0/0	0/0	1/1	0/0	0/0	0/0	0/0	0/0	1/1	0/0	0/0	0/0	0/0	0/0	0/0	0/0	0/0	0/0	1/1	0/0	0/0	0/0	0/0	0/0	0/0	0/0	0/0	0/0	0/0	0/0	0/0	0/0	0/0	1/1	0/0	0/0	1/1	0/0	0/0	.	0/0	0/0	0/0	0/0	0/0	0/0	0/0	1/1	1/1	0/0	0/0	0/0	0/0	1/1	0/0	1/1	0/0	0/0	0/0	0/0	0/0	0/0	0/0	0/0	0/0	0/0	1/1	0/0	0/0	0/0	0/0	0/0	0/0	0/0	0/0	0/0	0/0	0/0	0/0	0/0	0/0	0/0	0/0	0/0	0/0	0/0	0/0	0/0	0/0	1/1	.	0/0	0/0	1/1	0/0	0/0	0/0	0/0	0/0	.	0/0	0/0	0/0	0/0	0/0	0/0	0/0	0/0	0/0	1/1	0/0	1/1	0/0	1/1	1/1	0/0	0/0
2R	19034644	2R_19034644_SNP	A	C	.	.	T5EFF=-0.3481;T5VAR=0.152679489510205;T5VARACC=0.0921957287660818;T5PVAL=4.581e-07;T5MIXED=5.32126401621379e-07;T6EFF=-0.2672;T6VAR=0.0249743396459397;T6VARACC=0.0140109781880454;T6PVAL=0.0488;T6MIXED=0.0406376742369489;AVGEFF=-0.3077;AVGVAR=0.0676112420069224;AVGVARACC=0.0472738506484043;AVGPVAL=0.001045;AVGMIXED=0.00121557131268811;DIFFEFF=-0.08084;DIFFVAR=0.00382976603327278;DIFFVARACC=0.0063437452930038;DIFFPVAL=0.4428;DIFFMIXED=0.44375252541071;ANNOT=(FBgn0034827|Klp59D|2622 bp DOWNSTREAM),(FBgn0262843|CG43207|5571 bp DOWNSTREAM)	GT	0/0	0/0	0/0	1/1	0/0	0/0	0/0	0/0	0/0	1/1	0/0	0/0	0/0	.	1/1	0/0	0/0	0/0	0/0	0/0	0/0	0/0	0/0	0/0	0/0	0/0	0/0	0/0	0/0	.	0/0	0/0	.	0/0	0/0	0/0	0/0	0/0	0/0	0/0	0/0	0/0	1/1	0/0	.	0/0	.	0/0	.	0/0	0/0	0/0	0/0	0/0	1/1	0/0	0/0	0/0	0/0	0/0	0/0	0/0	1/1	0/0	0/0	0/0	0/0	.	0/0	0/0	0/0	0/0	0/0	1/1	0/0	0/0	0/0	1/1	0/0	0/0	0/0	0/0	1/1	0/0	0/0	0/0	.	0/0	0/0	.	0/0	0/0	0/0	0/0	.	0/0	0/0	.	0/0	0/0	0/0	0/0	0/0	0/0	0/0	0/0	1/1	0/0	0/0	0/0	.	.	0/0	0/0	0/0	0/0	0/0	0/0	0/0	0/0	0/0	0/0	0/0	1/1	0/0	0/0	0/0	0/0	0/0	0/0	0/0	0/0	0/0	.	0/0	0/0	0/0	0/0	0/0	0/0	0/0	0/0	0/0	0/0	0/0	0/0	.	1/1	0/0	0/0	0/0	0/0	0/0	0/0	.	0/0	.	0/0	0/0	0/0	0/0	0/0	0/0	0/0	0/0	.	.	0/0	0/0	0/0	0/0	0/0	0/0	0/0	1/1
2R	19034657	2R_19034657_SNP	A	G	.	.	T5EFF=-0.2962;T5VAR=0.115766425873783;T5VARACC=0.0794783110175851;T5PVAL=7.77e-06;T5MIXED=3.00991396472143e-06;T6EFF=-0.2078;T6VAR=0.0164104096190786;T6VARACC=0.00980937293771911;T6PVAL=0.1011;T6MIXED=0.0934963729761191;AVGEFF=-0.252;AVGVAR=0.048598623925638;AVGVARACC=0.0326837450899408;AVGPVAL=0.004437;AVGMIXED=0.00507184034480824;DIFFEFF=-0.08835;DIFFVAR=0.00502807666676439;DIFFVARACC=0.00494547002116746;DIFFPVAL=0.3654;DIFFMIXED=0.360077511742004;ANNOT=(FBgn0034827|Klp59D|2635 bp DOWNSTREAM),(FBgn0262843|CG43207|5558 bp DOWNSTREAM)	GT	0/0	0/0	0/0	1/1	0/0	0/0	0/0	0/0	0/0	1/1	0/0	0/0	0/0	0/0	1/1	0/0	0/0	.	0/0	0/0	0/0	0/0	0/0	0/0	0/0	0/0	0/0	0/0	0/0	0/0	0/0	0/0	.	0/0	0/0	0/0	0/0	0/0	.	0/0	0/0	0/0	1/1	0/0	.	0/0	1/1	0/0	.	0/0	0/0	0/0	0/0	0/0	1/1	0/0	0/0	0/0	0/0	0/0	0/0	0/0	1/1	0/0	0/0	0/0	0/0	.	0/0	0/0	0/0	0/0	0/0	1/1	0/0	0/0	0/0	1/1	0/0	0/0	0/0	0/0	.	0/0	0/0	0/0	0/0	0/0	0/0	1/1	0/0	0/0	0/0	0/0	0/0	0/0	0/0	0/0	0/0	0/0	0/0	0/0	0/0	0/0	0/0	0/0	1/1	0/0	0/0	0/0	0/0	.	0/0	0/0	0/0	0/0	0/0	0/0	0/0	0/0	0/0	0/0	0/0	1/1	0/0	0/0	0/0	0/0	0/0	0/0	0/0	0/0	0/0	0/0	0/0	0/0	0/0	0/0	0/0	0/0	0/0	0/0	0/0	0/0	0/0	0/0	1/1	1/1	0/0	0/0	0/0	0/0	0/0	0/0	0/0	0/0	.	0/0	0/0	0/0	0/0	0/0	0/0	0/0	0/0	0/0	.	0/0	0/0	0/0	0/0	0/0	0/0	0/0	1/1
2R	3601548	2R_3601548_SNP	T	G	.	.	T5EFF=-0.1395;T5VAR=0.0715678283119054;T5VARACC=0.0287284759636639;T5PVAL=0.0006019;T5MIXED=0.000295422910066237;T6EFF=-0.3141;T6VAR=0.106378431611316;T6VARACC=0.0312038451842627;T6PVAL=2.419e-05;T6MIXED=1.36711182242004e-05;AVGEFF=-0.2268;AVGVAR=0.11146305053259;AVGVARACC=0.048785945370071;AVGPVAL=1.504e-05;AVGMIXED=5.58988246900359e-06;DIFFEFF=0.1746;DIFFVAR=0.0551334882777065;DIFFVARACC=0.0153015966234217;DIFFPVAL=0.002716;DIFFMIXED=0.00203039317752177;ANNOT=(FBgn0033215|CG1942|NON_SYNONYMOUS_CODING),(FBgn0033216|CG1946|880 bp UPSTREAM)	GT	1/1	1/1	0/0	0/0	.	0/0	1/1	1/1	0/0	1/1	1/1	0/0	0/0	1/1	1/1	1/1	0/0	1/1	1/1	0/0	0/0	1/1	1/1	0/0	1/1	0/0	1/1	0/0	0/0	0/0	1/1	.	.	1/1	1/1	1/1	0/0	0/0	1/1	0/0	1/1	0/0	.	0/0	.	1/1	.	1/1	1/1	1/1	1/1	.	1/1	0/0	1/1	1/1	1/1	0/0	.	0/0	1/1	1/1	0/0	1/1	1/1	1/1	0/0	1/1	1/1	1/1	.	1/1	1/1	.	.	0/0	0/0	.	1/1	0/0	1/1	1/1	0/0	1/1	1/1	1/1	.	1/1	1/1	0/0	1/1	1/1	1/1	0/0	1/1	1/1	1/1	1/1	1/1	0/0	1/1	1/1	1/1	1/1	0/0	1/1	1/1	0/0	0/0	0/0	1/1	1/1	0/0	0/0	0/0	0/0	1/1	1/1	1/1	1/1	0/0	1/1	1/1	1/1	0/0	0/0	1/1	1/1	0/0	1/1	1/1	1/1	1/1	1/1	1/1	1/1	1/1	0/0	0/0	0/0	1/1	1/1	1/1	0/0	0/0	1/1	1/1	0/0	1/1	1/1	1/1	.	1/1	0/0	1/1	1/1	0/0	1/1	1/1	0/0	1/1	1/1	1/1	0/0	1/1	0/0	0/0	1/1	1/1	1/1	1/1	0/0	1/1	0/0	0/0
2R	8619847	2R_8619847_DEL	CG	C	.	.	T5EFF=-0.2149;T5VAR=0.127650234470759;T5VARACC=0.0515956487284462;T5PVAL=2.843e-06;T5MIXED=6.53329027053636e-06;T6EFF=-0.2443;T6VAR=0.0468182517713513;T6VARACC=0.0215473436263677;T6PVAL=0.005534;T6MIXED=0.00676459692688426;AVGEFF=-0.2296;AVGVAR=0.0838658753152373;AVGVARACC=0.045766148453898;AVGPVAL=0.0001772;AVGMIXED=0.000385973917472123;DIFFEFF=0.02935;DIFFVAR=0.00113580043079653;DIFFVARACC=0.00108746082826472;DIFFPVAL=0.6693;DIFFMIXED=0.685046778434217;ANNOT=(FBgn0261545|CG42663|INTRON)	GT	0/0	0/0	0/0	1/1	0/0	0/0	0/0	1/1	0/0	1/1	0/0	0/0	0/0	1/1	0/0	0/0	1/1	0/0	0/0	1/1	0/0	0/0	0/0	1/1	0/0	.	0/0	0/0	0/0	0/0	1/1	0/0	.	1/1	0/0	0/0	0/0	0/0	1/1	0/0	0/0	0/0	.	0/0	0/0	0/0	0/0	.	.	0/0	0/0	0/0	0/0	0/0	0/0	1/1	.	0/0	0/0	0/0	1/1	0/0	1/1	0/0	0/0	0/0	0/0	0/0	0/0	0/0	0/0	0/0	0/0	0/0	0/0	1/1	0/0	0/0	0/0	0/0	0/0	.	0/0	0/0	0/0	0/0	0/0	0/0	0/0	0/0	0/0	0/0	0/0	0/0	0/0	1/1	0/0	0/0	1/1	0/0	.	1/1	0/0	0/0	1/1	1/1	1/1	0/0	1/1	1/1	0/0	0/0	0/0	0/0	0/0	0/0	0/0	0/0	0/0	0/0	0/0	1/1	0/0	0/0	1/1	0/0	0/0	0/0	0/0	0/0	0/0	0/0	1/1	0/0	0/0	0/0	0/0	1/1	0/0	1/1	0/0	0/0	0/0	0/0	0/0	.	1/1	1/1	1/1	0/0	0/0	.	0/0	.	1/1	0/0	0/0	.	0/0	1/1	0/0	0/0	0/0	0/0	0/0	1/1	1/1	0/0	0/0	1/1	0/0	0/0	0/0	0/0	0/0
2R	8619858	2R_8619858_SNP	A	G	.	.	T5EFF=-0.2108;T5VAR=0.129554493942309;T5VARACC=0.0547789363444865;T5PVAL=2.368e-06;T5MIXED=2.96764698631574e-06;T6EFF=-0.2486;T6VAR=0.0521966515795864;T6VARACC=0.0251516916626883;T6PVAL=0.003354;T6MIXED=0.00360655938577116;AVGEFF=-0.2297;AVGVAR=0.090084630551311;AVGVARACC=0.0506625447332901;AVGPVAL=9.918e-05;AVGMIXED=0.000142017262894185;DIFFEFF=0.03787;DIFFVAR=0.00201508866770418;DIFFVARACC=0.00100295212537721;DIFFPVAL=0.5694;DIFFMIXED=0.572649239607184;ANNOT=(FBgn0261545|CG42663|INTRON)	GT	0/0	0/0	0/0	1/1	0/0	0/0	0/0	1/1	0/0	1/1	0/0	0/0	0/0	1/1	0/0	0/0	1/1	0/0	0/0	1/1	0/0	0/0	.	1/1	0/0	.	0/0	0/0	0/0	0/0	1/1	0/0	.	1/1	0/0	0/0	0/0	0/0	1/1	0/0	0/0	0/0	.	0/0	0/0	0/0	0/0	.	.	0/0	0/0	0/0	0/0	0/0	0/0	1/1	1/1	0/0	0/0	0/0	1/1	0/0	1/1	0/0	0/0	0/0	0/0	0/0	0/0	0/0	0/0	0/0	0/0	.	0/0	1/1	0/0	0/0	0/0	0/0	0/0	.	0/0	0/0	0/0	0/0	0/0	0/0	0/0	0/0	0/0	0/0	0/0	0/0	0/0	1/1	0/0	0/0	1/1	0/0	.	1/1	0/0	0/0	1/1	1/1	1/1	0/0	1/1	1/1	0/0	0/0	0/0	0/0	0/0	0/0	0/0	0/0	0/0	0/0	0/0	1/1	0/0	0/0	1/1	0/0	0/0	0/0	0/0	0/0	0/0	0/0	1/1	0/0	0/0	0/0	0/0	1/1	0/0	1/1	0/0	0/0	0/0	0/0	0/0	.	1/1	1/1	1/1	0/0	0/0	1/1	0/0	1/1	1/1	0/0	0/0	.	0/0	1/1	0/0	0/0	0/0	0/0	0/0	1/1	1/1	0/0	.	1/1	0/0	0/0	0/0	0/0	0/0
2R	9404826	2R_9404826_SNP	A	G	.	.	T5EFF=0.1593;T5VAR=0.0775391026092783;T5VARACC=0.021435201983959;T5PVAL=0.0002687;T5MIXED=0.000489784295843754;T6EFF=0.3559;T6VAR=0.109877735519295;T6VARACC=0.0250475112533883;T6PVAL=1.21e-05;T6MIXED=7.76764402188522e-06;AVGEFF=0.2576;AVGVAR=0.116480073187641;AVGVARACC=0.0354571248992741;AVGPVAL=6.373e-06;AVGMIXED=6.16966799172805e-06;DIFFEFF=-0.1966;DIFFVAR=0.0567413008010523;DIFFVARACC=0.00822650760070776;DIFFPVAL=0.001936;DIFFMIXED=0.00152629238132209;ANNOT=(FBgn0033848|CG13330|UTR_3_PRIME),(FBgn0260964|Vmat|666 bp DOWNSTREAM)	GT	0/0	0/0	0/0	0/0	0/0	1/1	1/1	0/0	0/0	0/0	0/0	0/0	0/0	1/1	0/0	0/0	0/0	0/0	1/1	1/1	0/0	0/0	1/1	0/0	0/0	.	0/0	1/1	0/0	0/0	0/0	0/0	0/0	0/0	0/0	0/0	0/0	0/0	0/0	0/0	1/1	.	0/0	0/0	.	1/1	0/0	.	0/0	0/0	0/0	1/1	.	0/0	0/0	1/1	0/0	0/0	0/0	0/0	0/0	1/1	0/0	1/1	0/0	0/0	0/0	1/1	0/0	0/0	0/0	0/0	1/1	0/0	0/0	0/0	0/0	0/0	0/0	0/0	1/1	0/0	0/0	1/1	0/0	0/0	1/1	0/0	0/0	1/1	0/0	0/0	1/1	0/0	0/0	1/1	1/1	0/0	0/0	0/0	0/0	1/1	0/0	1/1	0/0	0/0	0/0	1/1	0/0	0/0	0/0	1/1	1/1	1/1	0/0	1/1	0/0	0/0	0/0	0/0	1/1	0/0	0/0	0/0	0/0	0/0	0/0	.	0/0	1/1	0/0	0/0	0/0	0/0	0/0	1/1	1/1	0/0	1/1	1/1	0/0	0/0	1/1	0/0	1/1	.	0/0	0/0	0/0	0/0	1/1	1/1	0/0	0/0	1/1	0/0	1/1	.	0/0	0/0	0/0	0/0	0/0	0/0	0/0	0/0	0/0	0/0	0/0	0/0	1/1	0/0	0/0	0/0	0/0
2R	9738814	2R_9738814_SNP	T	G	.	.	T5EFF=-0.232;T5VAR=0.114303863999084;T5VARACC=0.0632197038211032;T5PVAL=7.391e-06;T5MIXED=3.64771695431367e-05;T6EFF=-0.3289;T6VAR=0.0661024403193212;T6VARACC=0.0214106016175049;T6PVAL=0.0007674;T6MIXED=0.000829636504652462;AVGEFF=-0.2804;AVGVAR=0.096846060592978;AVGVARACC=0.0453726565398286;AVGPVAL=4.027e-05;AVGMIXED=8.92222340297251e-05;DIFFEFF=0.09686;DIFFVAR=0.00971416927700085;DIFFVARACC=0.000550230490834414;DIFFPVAL=0.2037;DIFFMIXED=0.19629787902491;ANNOT=(FBgn0050485|CG30485|SYNONYMOUS_CODING)	GT	0/0	0/0	0/0	1/1	0/0	0/0	0/0	0/0	0/0	1/1	0/0	0/0	0/0	0/0	0/0	0/0	1/1	0/0	0/0	0/0	0/0	0/0	0/0	0/0	0/0	0/0	1/1	1/1	0/0	0/0	0/0	0/0	0/0	0/0	0/0	0/0	0/0	1/1	0/0	0/0	0/0	0/0	0/0	1/1	.	0/0	0/0	1/1	.	0/0	0/0	0/0	0/0	0/0	0/0	0/0	1/1	0/0	0/0	0/0	0/0	0/0	1/1	0/0	0/0	0/0	.	0/0	1/1	0/0	0/0	0/0	0/0	0/0	0/0	0/0	0/0	0/0	0/0	0/0	0/0	0/0	0/0	1/1	0/0	0/0	1/1	0/0	0/0	0/0	0/0	0/0	0/0	0/0	0/0	0/0	0/0	1/1	0/0	0/0	0/0	0/0	0/0	0/0	0/0	1/1	0/0	0/0	0/0	1/1	0/0	0/0	0/0	1/1	0/0	0/0	0/0	1/1	1/1	0/0	0/0	0/0	0/0	0/0	0/0	0/0	0/0	0/0	0/0	0/0	0/0	0/0	0/0	0/0	0/0	0/0	0/0	0/0	0/0	0/0	0/0	1/1	0/0	0/0	.	.	0/0	1/1	0/0	0/0	0/0	1/1	0/0	0/0	1/1	0/0	0/0	.	0/0	1/1	1/1	0/0	0/0	0/0	0/0	0/0	0/0	0/0	0/0	.	0/0	0/0	0/0	0/0	1/1
3L	10017877	3L_10017877_SNP	G	C	.	.	T5EFF=-0.3117;T5VAR=0.113239993968178;T5VARACC=0.047594586402623;T5PVAL=6.791e-06;T5MIXED=9.42180398411614e-06;T6EFF=-0.4245;T6VAR=0.0595338890034815;T6VARACC=0.0198759109173733;T6PVAL=0.0013;T6MIXED=0.00132484729090768;AVGEFF=-0.3681;AVGVAR=0.0908353244445062;AVGVARACC=0.0355700396300553;AVGPVAL=6.18e-05;AVGMIXED=9.02903974090397e-05;DIFFEFF=0.1128;DIFFVAR=0.00705616118225525;DIFFVARACC=0.00441358815580295;DIFFPVAL=0.2747;DIFFMIXED=0.267068306762854;ANNOT=(FBgn0040823|dpr6|INTRON)	GT	0/0	0/0	0/0	1/1	0/0	0/0	0/0	0/0	1/1	0/0	0/0	0/0	0/0	0/0	0/0	1/1	0/0	.	0/0	0/0	0/0	0/0	0/0	0/0	0/0	1/1	0/0	0/0	0/0	0/0	0/0	0/0	0/0	0/0	0/0	0/0	0/0	0/0	0/0	0/0	0/0	0/0	0/0	0/0	0/0	0/0	0/0	0/0	0/0	0/0	0/0	0/0	0/0	0/0	0/0	0/0	0/0	1/1	0/0	0/0	0/0	0/0	0/0	0/0	0/0	0/0	0/0	0/0	0/0	0/0	0/0	0/0	0/0	0/0	0/0	0/0	0/0	.	0/0	0/0	0/0	0/0	1/1	1/1	0/0	0/0	0/0	0/0	0/0	0/0	0/0	0/0	0/0	0/0	0/0	0/0	0/0	.	1/1	0/0	0/0	0/0	0/0	0/0	0/0	0/0	1/1	0/0	0/0	0/0	0/0	0/0	0/0	0/0	0/0	0/0	0/0	0/0	0/0	0/0	0/0	0/0	0/0	0/0	0/0	0/0	0/0	0/0	0/0	0/0	0/0	0/0	0/0	0/0	0/0	0/0	0/0	1/1	0/0	0/0	0/0	0/0	0/0	0/0	0/0	0/0	0/0	0/0	0/0	0/0	0/0	0/0	0/0	0/0	1/1	.	1/1	0/0	0/0	1/1	0/0	0/0	0/0	0/0	0/0	0/0	0/0	0/0	0/0	0/0	0/0	0/0	0/0	0/0	0/0
3L	10151119	3L_10151119_SNP	G	A	.	.	T5EFF=-0.3382;T5VAR=0.141399123801982;T5VARACC=0.0808246561487894;T5PVAL=3.425e-07;T5MIXED=1.51637997756291e-06;T6EFF=-0.3773;T6VAR=0.050222621521361;T6VARACC=0.0260995977950993;T6PVAL=0.003036;T6MIXED=0.00348494036227679;AVGEFF=-0.3578;AVGVAR=0.091065977215874;AVGVARACC=0.0630050272722815;AVGPVAL=5.463e-05;AVGMIXED=0.00011123918095089;DIFFEFF=0.03907;DIFFVAR=0.000917020354745759;DIFFVARACC=1.41654226404331e-07;DIFFPVAL=0.6925;DIFFMIXED=0.689476198112234;ANNOT=(FBgn0052057|dpr10|INTRON)	GT	0/0	0/0	0/0	0/0	0/0	0/0	0/0	1/1	0/0	0/0	0/0	0/0	0/0	0/0	0/0	0/0	0/0	0/0	0/0	0/0	0/0	0/0	0/0	0/0	0/0	0/0	0/0	0/0	0/0	0/0	0/0	0/0	0/0	0/0	0/0	0/0	0/0	0/0	0/0	0/0	0/0	0/0	1/1	0/0	1/1	0/0	0/0	0/0	0/0	0/0	0/0	0/0	0/0	0/0	0/0	0/0	0/0	0/0	0/0	0/0	0/0	0/0	0/0	0/0	0/0	0/0	0/0	.	1/1	0/0	0/0	0/0	0/0	0/0	0/0	0/0	0/0	0/0	0/0	0/0	0/0	0/0	0/0	0/0	0/0	0/0	0/0	0/0	0/0	0/0	0/0	0/0	0/0	0/0	0/0	0/0	0/0	0/0	0/0	0/0	0/0	0/0	0/0	0/0	0/0	0/0	1/1	0/0	0/0	1/1	0/0	1/1	0/0	1/1	0/0	0/0	0/0	0/0	.	0/0	0/0	0/0	0/0	0/0	0/0	0/0	0/0	0/0	0/0	0/0	0/0	0/0	0/0	0/0	0/0	0/0	0/0	0/0	0/0	0/0	0/0	0/0	0/0	0/0	0/0	1/1	0/0	1/1	0/0	0/0	0/0	0/0	0/0	0/0	0/0	0/0	1/1	0/0	0/0	0/0	0/0	0/0	0/0	0/0	0/0	1/1	0/0	1/1	0/0	0/0	0/0	0/0	0/0	0/0	1/1
3L	10157408	3L_10157408_SNP	T	C	.	.	T5EFF=-0.3856;T5VAR=0.134187426336316;T5VARACC=0.0863695313789986;T5PVAL=6.657e-07;T5MIXED=1.41488105876373e-06;T6EFF=-0.4703;T6VAR=0.0566111335732834;T6VARACC=0.0172210117092042;T6PVAL=0.00157;T6MIXED=0.00155165619096214;AVGEFF=-0.4279;AVGVAR=0.0946529558286622;AVGVARACC=0.0543310145873345;AVGPVAL=3.633e-05;AVGMIXED=4.75322546463212e-05;DIFFEFF=0.08464;DIFFVAR=0.00312826755669387;DIFFVARACC=0.000281703121148502;DIFFPVAL=0.4635;DIFFMIXED=0.458850273976077;ANNOT=(FBgn0052057|dpr10|INTRON)	GT	0/0	0/0	0/0	0/0	0/0	0/0	0/0	1/1	0/0	0/0	0/0	0/0	0/0	0/0	0/0	0/0	0/0	0/0	0/0	0/0	0/0	0/0	0/0	0/0	0/0	0/0	0/0	0/0	0/0	0/0	0/0	0/0	0/0	0/0	0/0	0/0	0/0	0/0	0/0	0/0	0/0	0/0	1/1	0/0	1/1	0/0	0/0	0/0	0/0	0/0	0/0	0/0	0/0	0/0	0/0	0/0	0/0	0/0	0/0	0/0	0/0	0/0	0/0	0/0	0/0	0/0	0/0	0/0	1/1	0/0	0/0	0/0	0/0	0/0	0/0	0/0	0/0	0/0	0/0	0/0	0/0	0/0	1/1	0/0	0/0	0/0	0/0	0/0	0/0	0/0	0/0	0/0	0/0	0/0	0/0	0/0	0/0	0/0	0/0	0/0	0/0	0/0	0/0	0/0	0/0	0/0	0/0	0/0	0/0	1/1	0/0	0/0	0/0	1/1	0/0	0/0	0/0	0/0	.	0/0	0/0	0/0	0/0	0/0	0/0	0/0	0/0	0/0	0/0	0/0	0/0	0/0	0/0	0/0	0/0	0/0	0/0	0/0	0/0	0/0	0/0	0/0	0/0	0/0	0/0	0/0	0/0	1/1	0/0	0/0	0/0	0/0	0/0	0/0	0/0	0/0	0/0	0/0	0/0	0/0	0/0	0/0	0/0	0/0	0/0	1/1	0/0	0/0	0/0	0/0	0/0	0/0	0/0	0/0	1/1
3L	10160379	3L_10160379_SNP	C	G	.	.	T5EFF=-0.3225;T5VAR=0.136231348470594;T5VARACC=0.0780410902992583;T5PVAL=5.395e-07;T5MIXED=1.08839966101809e-06;T6EFF=-0.3116;T6VAR=0.0360584950694541;T6VARACC=0.0165903774953088;T6PVAL=0.01208;T6MIXED=0.0130413877492939;AVGEFF=-0.317;AVGVAR=0.0756244376913823;AVGVARACC=0.0457037023312238;AVGPVAL=0.0002403;AVGMIXED=0.000382803079421984;DIFFEFF=-0.01085;DIFFVAR=7.36977817733426e-05;DIFFVARACC=0.000309688400051911;DIFFPVAL=0.9105;DIFFMIXED=0.902364201614052;ANNOT=(FBgn0052057|dpr10|INTRON),(FBgn0036072|CG6628|256 bp UPSTREAM)	GT	0/0	1/1	0/0	0/0	.	0/0	0/0	0/0	0/0	0/0	0/0	0/0	0/0	0/0	0/0	0/0	0/0	0/0	0/0	1/1	0/0	0/0	0/0	0/0	0/0	0/0	0/0	0/0	0/0	0/0	0/0	0/0	0/0	0/0	0/0	0/0	0/0	0/0	0/0	0/0	0/0	0/0	1/1	0/0	0/0	0/0	0/0	0/0	0/0	0/0	0/0	0/0	0/0	0/0	0/0	0/0	0/0	0/0	0/0	0/0	0/0	0/0	0/0	0/0	0/0	0/0	0/0	0/0	0/0	0/0	0/0	0/0	1/1	0/0	0/0	0/0	0/0	0/0	0/0	0/0	0/0	0/0	1/1	0/0	0/0	0/0	0/0	0/0	0/0	0/0	0/0	0/0	0/0	0/0	0/0	0/0	0/0	0/0	1/1	0/0	0/0	0/0	0/0	0/0	0/0	0/0	1/1	0/0	0/0	1/1	0/0	0/0	0/0	1/1	0/0	0/0	0/0	0/0	0/0	0/0	0/0	0/0	0/0	0/0	0/0	0/0	0/0	0/0	0/0	0/0	0/0	0/0	0/0	0/0	0/0	0/0	1/1	1/1	0/0	0/0	0/0	0/0	0/0	0/0	0/0	0/0	0/0	1/1	0/0	1/1	0/0	0/0	0/0	0/0	0/0	0/0	1/1	0/0	0/0	0/0	0/0	0/0	0/0	0/0	0/0	1/1	0/0	0/0	0/0	0/0	0/0	0/0	0/0	0/0	0/0
3L	10169370	3L_10169370_INS	A	TT	.	.	T5EFF=-0.2944;T5VAR=0.113232136225934;T5VARACC=0.10052258173813;T5PVAL=5.994e-06;T5MIXED=2.82811466156604e-05;T6EFF=-0.3329;T6VAR=0.0418137500117056;T6VARACC=0.0224414707008601;T6PVAL=0.006962;T6MIXED=0.00948378752526026;AVGEFF=-0.3137;AVGVAR=0.0745951867733014;AVGVARACC=0.0523884588678038;AVGPVAL=0.0002773;AVGMIXED=0.000810958694124785;DIFFEFF=0.0385;DIFFVAR=0.000952281276842795;DIFFVARACC=0.00179204649549242;DIFFPVAL=0.6869;DIFFMIXED=0.701414921383783;ANNOT=(FBgn0052057|dpr10|INTRON)	GT	1/1	1/1	0/0	1/1	0/0	0/0	0/0	0/0	.	0/0	0/0	0/0	0/0	0/0	0/0	0/0	0/0	0/0	0/0	0/0	0/0	0/0	0/0	0/0	0/0	0/0	0/0	0/0	0/0	0/0	0/0	0/0	0/0	0/0	0/0	0/0	0/0	0/0	0/0	0/0	0/0	0/0	0/0	0/0	0/0	0/0	0/0	0/0	0/0	0/0	0/0	0/0	0/0	0/0	0/0	0/0	0/0	0/0	0/0	0/0	0/0	0/0	0/0	0/0	1/1	0/0	0/0	0/0	1/1	0/0	0/0	0/0	0/0	0/0	0/0	0/0	0/0	0/0	0/0	0/0	0/0	0/0	0/0	0/0	0/0	1/1	0/0	0/0	0/0	0/0	0/0	0/0	0/0	0/0	1/1	0/0	0/0	0/0	0/0	0/0	0/0	0/0	0/0	0/0	0/0	0/0	1/1	0/0	0/0	1/1	0/0	0/0	0/0	0/0	0/0	0/0	0/0	0/0	1/1	0/0	0/0	0/0	0/0	0/0	0/0	0/0	0/0	0/0	1/1	0/0	0/0	0/0	0/0	0/0	0/0	0/0	0/0	1/1	0/0	0/0	0/0	0/0	0/0	0/0	0/0	0/0	0/0	1/1	0/0	0/0	0/0	0/0	.	0/0	0/0	0/0	1/1	0/0	0/0	0/0	0/0	0/0	0/0	0/0	0/0	1/1	0/0	0/0	0/0	0/0	0/0	0/0	0/0	0/0	0/0
3L	10170887	3L_10170887_SNP	A	G	.	.	T5EFF=-0.3313;T5VAR=0.116937265182145;T5VARACC=0.0928565815634814;T5PVAL=3.627e-06;T5MIXED=2.28596282110229e-05;T6EFF=-0.2891;T6VAR=0.0252977354706074;T6VARACC=0.0137985690020987;T6PVAL=0.03552;T6MIXED=0.0443098643154115;AVGEFF=-0.3102;AVGVAR=0.058968017225394;AVGVARACC=0.0433508925098308;AVGPVAL=0.001204;AVGMIXED=0.00309254073113449;DIFFEFF=-0.04222;DIFFVAR=0.000910418627940729;DIFFVARACC=4.6234157159708e-05;DIFFPVAL=0.6918;DIFFMIXED=0.681552366413258;ANNOT=(FBgn0052057|dpr10|INTRON)	GT	1/1	1/1	0/0	1/1	0/0	0/0	0/0	0/0	0/0	0/0	0/0	0/0	0/0	0/0	0/0	0/0	0/0	0/0	0/0	0/0	0/0	0/0	0/0	0/0	0/0	0/0	0/0	0/0	0/0	0/0	0/0	0/0	0/0	0/0	0/0	0/0	0/0	0/0	0/0	0/0	0/0	0/0	0/0	0/0	0/0	0/0	0/0	0/0	0/0	0/0	0/0	0/0	0/0	0/0	0/0	0/0	0/0	0/0	0/0	0/0	0/0	0/0	0/0	0/0	1/1	0/0	0/0	0/0	0/0	0/0	0/0	0/0	0/0	0/0	0/0	0/0	0/0	0/0	0/0	0/0	0/0	0/0	0/0	0/0	0/0	1/1	0/0	0/0	0/0	0/0	0/0	0/0	0/0	0/0	1/1	0/0	0/0	0/0	0/0	0/0	0/0	0/0	0/0	0/0	0/0	0/0	1/1	0/0	0/0	1/1	0/0	0/0	0/0	0/0	0/0	0/0	0/0	0/0	0/0	0/0	0/0	0/0	0/0	0/0	0/0	0/0	0/0	0/0	1/1	0/0	0/0	0/0	0/0	0/0	0/0	0/0	0/0	0/0	0/0	0/0	0/0	0/0	0/0	0/0	0/0	0/0	0/0	1/1	0/0	0/0	0/0	0/0	0/0	0/0	0/0	0/0	1/1	0/0	0/0	0/0	0/0	0/0	0/0	0/0	0/0	1/1	0/0	0/0	0/0	0/0	0/0	0/0	0/0	0/0	0/0
3L	10399602	3L_10399602_SNP	C	T	.	.	T5EFF=-0.1778;T5VAR=0.0923209066358557;T5VARACC=0.0578923532130368;T5PVAL=5.624e-05;T5MIXED=7.06283390883305e-06;T6EFF=-0.1844;T6VAR=0.0283683740456491;T6VARACC=0.0335468803753102;T6PVAL=0.02812;T6MIXED=0.0264228608123324;AVGEFF=-0.1811;AVGVAR=0.0554943536198539;AVGVARACC=0.0426344044926563;AVGPVAL=0.001985;AVGMIXED=0.0010572581531827;DIFFEFF=0.006598;DIFFVAR=6.06274840693064e-05;DIFFVARACC=0.00010547980704706;DIFFPVAL=0.9197;DIFFMIXED=0.921777317583031;ANNOT=(FBgn0265415|CR44327|39046 bp DOWNSTREAM),(FBgn0264722|CR43990|5405 bp UPSTREAM)	GT	0/0	0/0	0/0	1/1	.	1/1	0/0	1/1	0/0	1/1	0/0	0/0	0/0	1/1	0/0	1/1	0/0	0/0	0/0	0/0	0/0	1/1	0/0	0/0	0/0	1/1	1/1	0/0	0/0	0/0	0/0	0/0	0/0	0/0	0/0	0/0	0/0	0/0	0/0	0/0	0/0	0/0	1/1	0/0	0/0	1/1	0/0	0/0	1/1	0/0	1/1	0/0	0/0	0/0	1/1	0/0	1/1	1/1	0/0	0/0	0/0	0/0	0/0	0/0	0/0	0/0	0/0	0/0	0/0	0/0	0/0	0/0	0/0	0/0	0/0	0/0	0/0	0/0	0/0	0/0	0/0	0/0	0/0	0/0	1/1	1/1	0/0	1/1	1/1	0/0	0/0	0/0	0/0	0/0	1/1	1/1	0/0	0/0	0/0	0/0	0/0	.	0/0	0/0	0/0	1/1	0/0	0/0	1/1	1/1	0/0	0/0	0/0	.	0/0	1/1	0/0	0/0	0/0	1/1	0/0	0/0	0/0	0/0	1/1	0/0	0/0	0/0	0/0	0/0	0/0	0/0	0/0	0/0	0/0	0/0	0/0	0/0	1/1	0/0	1/1	1/1	0/0	0/0	1/1	0/0	0/0	1/1	0/0	0/0	0/0	0/0	0/0	1/1	0/0	1/1	0/0	0/0	1/1	1/1	0/0	0/0	0/0	0/0	0/0	0/0	0/0	0/0	0/0	.	0/0	0/0	.	1/1	0/0
3L	10722020	3L_10722020_SNP	G	A	.	.	T5EFF=-0.319;T5VAR=0.111155055817624;T5VARACC=0.0711182946322143;T5PVAL=8.884e-06;T5MIXED=1.34266349306231e-05;T6EFF=-0.3341;T6VAR=0.0351483153788675;T6VARACC=0.0312553825274478;T6PVAL=0.01436;T6MIXED=0.0183074623950638;AVGEFF=-0.3265;AVGVAR=0.0674174915136854;AVGVARACC=0.0479421641355458;AVGPVAL=0.0006279;AVGMIXED=0.00156982990137566;DIFFEFF=0.01512;DIFFVAR=0.000123120821035113;DIFFVARACC=4.06168030592235e-06;DIFFPVAL=0.8858;DIFFMIXED=0.891416366725536;ANNOT=(FBgn0036101|NijA|32043 bp UPSTREAM),(FBgn0262890|CG43245|43005 bp DOWNSTREAM)	GT	0/0	0/0	0/0	0/0	0/0	0/0	0/0	0/0	0/0	1/1	0/0	0/0	0/0	0/0	0/0	1/1	0/0	0/0	0/0	1/1	0/0	0/0	0/0	0/0	.	0/0	1/1	0/0	0/0	0/0	0/0	0/0	0/0	0/0	0/0	0/0	0/0	0/0	0/0	0/0	0/0	0/0	0/0	0/0	0/0	0/0	0/0	0/0	0/0	0/0	.	0/0	0/0	0/0	1/1	0/0	0/0	0/0	0/0	0/0	0/0	0/0	0/0	0/0	1/1	0/0	0/0	0/0	0/0	0/0	0/0	0/0	1/1	0/0	0/0	0/0	0/0	0/0	0/0	0/0	0/0	0/0	0/0	0/0	0/0	0/0	0/0	0/0	0/0	0/0	0/0	0/0	0/0	0/0	0/0	0/0	0/0	0/0	0/0	0/0	0/0	0/0	0/0	0/0	0/0	1/1	0/0	0/0	0/0	0/0	0/0	0/0	0/0	1/1	0/0	0/0	0/0	0/0	0/0	0/0	.	0/0	0/0	0/0	0/0	0/0	0/0	0/0	0/0	0/0	0/0	0/0	0/0	0/0	0/0	0/0	0/0	0/0	1/1	0/0	0/0	0/0	0/0	0/0	0/0	0/0	0/0	1/1	0/0	0/0	0/0	0/0	0/0	0/0	0/0	0/0	0/0	0/0	0/0	0/0	0/0	1/1	0/0	0/0	0/0	0/0	0/0	0/0	0/0	0/0	0/0	0/0	.	.	0/0
3L	1083807	3L_1083807_SNP	C	T	.	.	T5EFF=0.0284;T5VAR=0.00324783802044734;T5VARACC=0.0110907908689701;T5PVAL=0.4672;T5MIXED=0.244819263460148;T6EFF=-0.2308;T6VAR=0.0629447489094217;T6VARACC=3.24133235779511e-06;T6PVAL=0.001153;T6MIXED=0.00116289968229743;AVGEFF=-0.1012;AVGVAR=0.0244181127597066;AVGVARACC=0.00487952066230651;AVGPVAL=0.04504;AVGMIXED=0.0541070822866761;DIFFEFF=0.2592;DIFFVAR=0.131443648502479;DIFFVARACC=0.00518256707926468;DIFFPVAL=1.706e-06;DIFFMIXED=1.17802954600227e-06;ANNOT=(FBgn0004870|bab1|INTRON)	GT	0/0	1/1	0/0	0/0	1/1	1/1	0/0	1/1	1/1	0/0	0/0	0/0	1/1	1/1	0/0	0/0	1/1	1/1	0/0	0/0	1/1	0/0	0/0	1/1	0/0	0/0	0/0	0/0	0/0	0/0	1/1	1/1	0/0	0/0	0/0	0/0	0/0	1/1	1/1	1/1	1/1	0/0	0/0	1/1	0/0	1/1	1/1	.	0/0	0/0	1/1	0/0	1/1	.	0/0	.	0/0	1/1	.	.	0/0	0/0	0/0	1/1	0/0	0/0	0/0	0/0	1/1	1/1	0/0	0/0	0/0	0/0	0/0	0/0	1/1	0/0	1/1	1/1	.	1/1	1/1	1/1	1/1	0/0	1/1	1/1	1/1	1/1	0/0	0/0	0/0	.	1/1	0/0	0/0	1/1	0/0	1/1	1/1	0/0	1/1	0/0	1/1	1/1	0/0	1/1	0/0	0/0	0/0	.	0/0	0/0	1/1	1/1	0/0	1/1	1/1	0/0	0/0	1/1	0/0	1/1	1/1	1/1	0/0	0/0	1/1	1/1	0/0	0/0	0/0	1/1	1/1	0/0	0/0	0/0	1/1	0/0	0/0	0/0	.	1/1	0/0	0/0	0/0	0/0	1/1	1/1	1/1	1/1	1/1	0/0	1/1	0/0	0/0	0/0	0/0	1/1	1/1	1/1	0/0	1/1	0/0	1/1	0/0	1/1	0/0	.	1/1	1/1	1/1	1/1	0/0
3L	1083809	3L_1083809_SNP	G	A	.	.	T5EFF=0.04264;T5VAR=0.00733959188413019;T5VARACC=0.0136171549736944;T5PVAL=0.2724;T5MIXED=0.0939118914535044;T6EFF=-0.1958;T6VAR=0.0449398330136432;T6VARACC=4.14057537654888e-05;T6PVAL=0.006109;T6MIXED=0.00633274262634858;AVGEFF=-0.07656;AVGVAR=0.0138647720312704;AVGVARACC=0.00466938460015015;AVGPVAL=0.1308;AVGMIXED=0.17475867037077;DIFFEFF=0.2384;DIFFVAR=0.11145976108702;DIFFVARACC=0.0047510765458027;DIFFPVAL=1.104e-05;DIFFMIXED=7.85663621850212e-06;ANNOT=(FBgn0004870|bab1|INTRON)	GT	0/0	1/1	0/0	0/0	1/1	.	0/0	1/1	1/1	0/0	0/0	0/0	1/1	1/1	0/0	0/0	1/1	1/1	0/0	0/0	1/1	0/0	0/0	1/1	0/0	0/0	0/0	0/0	0/0	0/0	1/1	1/1	0/0	0/0	0/0	0/0	0/0	1/1	1/1	1/1	1/1	0/0	0/0	1/1	0/0	1/1	1/1	.	0/0	0/0	1/1	0/0	1/1	1/1	0/0	1/1	0/0	1/1	1/1	1/1	0/0	0/0	0/0	1/1	0/0	0/0	0/0	0/0	1/1	1/1	0/0	0/0	0/0	0/0	0/0	0/0	1/1	0/0	1/1	.	1/1	1/1	1/1	1/1	1/1	0/0	1/1	1/1	1/1	1/1	0/0	0/0	0/0	.	1/1	.	0/0	1/1	0/0	1/1	1/1	0/0	1/1	0/0	1/1	1/1	0/0	1/1	0/0	0/0	0/0	.	0/0	0/0	1/1	1/1	0/0	1/1	1/1	.	0/0	1/1	0/0	1/1	1/1	1/1	0/0	0/0	1/1	1/1	0/0	0/0	0/0	1/1	1/1	0/0	0/0	0/0	1/1	0/0	0/0	0/0	0/0	1/1	.	0/0	0/0	0/0	1/1	1/1	1/1	1/1	1/1	0/0	1/1	0/0	0/0	0/0	0/0	1/1	1/1	1/1	0/0	1/1	0/0	1/1	0/0	1/1	0/0	.	1/1	1/1	1/1	1/1	0/0
3L	1084059	3L_1084059_SNP	T	C	.	.	T5EFF=0.04124;T5VAR=0.00688047981430549;T5VARACC=0.0116548438446204;T5PVAL=0.2851;T5MIXED=0.119109884405944;T6EFF=-0.1954;T6VAR=0.0442015251816655;T6VARACC=0.00368164551543704;T6PVAL=0.006232;T6MIXED=0.00508479067448339;AVGEFF=-0.0771;AVGVAR=0.0138902101213461;AVGVARACC=0.00832996459571145;AVGPVAL=0.1281;AVGMIXED=0.137409331630395;DIFFEFF=0.2367;DIFFVAR=0.109661128631422;DIFFVARACC=0.00175526729238823;DIFFPVAL=1.162e-05;DIFFMIXED=7.38138500018222e-06;ANNOT=(FBgn0004870|bab1|INTRON)	GT	0/0	1/1	0/0	0/0	1/1	1/1	0/0	1/1	1/1	0/0	0/0	1/1	1/1	1/1	0/0	0/0	.	1/1	0/0	0/0	1/1	0/0	0/0	1/1	0/0	0/0	0/0	0/0	0/0	0/0	1/1	1/1	.	0/0	.	0/0	1/1	1/1	1/1	1/1	1/1	0/0	0/0	1/1	0/0	1/1	.	0/0	0/0	1/1	1/1	0/0	1/1	1/1	0/0	1/1	0/0	1/1	1/1	1/1	1/1	0/0	0/0	0/0	0/0	0/0	1/1	0/0	1/1	1/1	0/0	0/0	0/0	0/0	1/1	0/0	1/1	0/0	1/1	1/1	1/1	1/1	1/1	1/1	1/1	0/0	0/0	1/1	1/1	1/1	0/0	0/0	0/0	0/0	1/1	0/0	0/0	1/1	0/0	1/1	1/1	0/0	1/1	1/1	1/1	1/1	0/0	1/1	0/0	0/0	0/0	.	0/0	0/0	.	1/1	0/0	1/1	1/1	0/0	0/0	1/1	0/0	1/1	1/1	1/1	0/0	0/0	1/1	1/1	0/0	1/1	.	1/1	1/1	0/0	0/0	0/0	1/1	0/0	0/0	0/0	0/0	1/1	0/0	0/0	0/0	0/0	1/1	1/1	1/1	1/1	1/1	0/0	1/1	0/0	0/0	0/0	0/0	1/1	1/1	1/1	0/0	1/1	0/0	1/1	0/0	1/1	0/0	0/0	1/1	1/1	0/0	1/1	0/0
3L	1084129	3L_1084129_SNP	T	C	.	.	T5EFF=0.01636;T5VAR=0.00110442195694797;T5VARACC=0.0101676714845079;T5PVAL=0.668;T5MIXED=0.307668866697053;T6EFF=-0.2334;T6VAR=0.0638407509055388;T6VARACC=0.000468775412496014;T6PVAL=0.0009189;T6MIXED=0.000818253969682069;AVGEFF=-0.1085;AVGVAR=0.0279352841352549;AVGVARACC=0.00458857265424056;AVGPVAL=0.02986;AVGMIXED=0.0409101181478911;DIFFEFF=0.2497;DIFFVAR=0.123454306211257;DIFFVARACC=0.00164324993327136;DIFFPVAL=2.813e-06;DIFFMIXED=1.96073837152426e-06;ANNOT=(FBgn0004870|bab1|INTRON)	GT	0/0	1/1	0/0	0/0	1/1	1/1	0/0	1/1	1/1	0/0	1/1	1/1	1/1	1/1	0/0	1/1	1/1	1/1	0/0	0/0	1/1	0/0	0/0	1/1	0/0	0/0	0/0	0/0	0/0	0/0	1/1	1/1	.	0/0	1/1	0/0	1/1	1/1	1/1	1/1	1/1	0/0	0/0	1/1	0/0	1/1	1/1	.	0/0	0/0	1/1	0/0	1/1	1/1	0/0	1/1	.	1/1	1/1	1/1	1/1	0/0	0/0	0/0	0/0	0/0	1/1	0/0	1/1	1/1	0/0	0/0	0/0	.	1/1	0/0	1/1	0/0	1/1	1/1	1/1	1/1	1/1	1/1	1/1	0/0	0/0	1/1	1/1	0/0	0/0	0/0	0/0	0/0	0/0	0/0	0/0	1/1	0/0	1/1	1/1	0/0	1/1	1/1	1/1	1/1	0/0	1/1	0/0	0/0	0/0	.	0/0	0/0	1/1	1/1	0/0	1/1	1/1	0/0	0/0	1/1	0/0	0/0	1/1	1/1	0/0	0/0	1/1	1/1	0/0	1/1	0/0	1/1	1/1	0/0	0/0	0/0	1/1	0/0	0/0	0/0	0/0	1/1	0/0	0/0	0/0	0/0	1/1	1/1	1/1	1/1	1/1	0/0	1/1	0/0	0/0	0/0	0/0	.	1/1	1/1	0/0	1/1	0/0	1/1	0/0	1/1	0/0	0/0	1/1	1/1	0/0	1/1	1/1
3L	1084199	3L_1084199_SNP	A	G	.	.	T5EFF=0.0099;T5VAR=0.000407772504390585;T5VARACC=0.0047526767991169;T5PVAL=0.7938;T5MIXED=0.402243039213883;T6EFF=-0.2692;T6VAR=0.0837049726424313;T6VARACC=0.00174414719058297;T6PVAL=0.0001299;T6MIXED=0.000119429539188186;AVGEFF=-0.1297;AVGVAR=0.0395488019599931;AVGVARACC=0.000185251328045699;AVGPVAL=0.009327;AVGMIXED=0.0144708189448535;DIFFEFF=0.2791;DIFFVAR=0.152162208721052;DIFFVARACC=0.0109240456435049;DIFFPVAL=1.454e-07;DIFFMIXED=8.74966558632467e-08;ANNOT=(FBgn0004870|bab1|INTRON)	GT	0/0	1/1	0/0	0/0	1/1	1/1	0/0	1/1	1/1	0/0	1/1	1/1	1/1	1/1	0/0	0/0	1/1	1/1	0/0	0/0	1/1	0/0	0/0	1/1	0/0	0/0	0/0	0/0	0/0	0/0	1/1	1/1	0/0	0/0	0/0	0/0	1/1	1/1	1/1	1/1	1/1	0/0	0/0	1/1	0/0	1/1	1/1	0/0	0/0	0/0	1/1	0/0	1/1	1/1	0/0	1/1	0/0	1/1	1/1	1/1	1/1	0/0	0/0	0/0	0/0	0/0	1/1	0/0	1/1	1/1	0/0	0/0	0/0	0/0	1/1	0/0	1/1	0/0	1/1	1/1	1/1	1/1	1/1	1/1	1/1	0/0	0/0	1/1	1/1	0/0	0/0	0/0	0/0	0/0	0/0	0/0	0/0	.	0/0	.	1/1	0/0	1/1	0/0	1/1	1/1	0/0	1/1	0/0	0/0	0/0	.	0/0	0/0	1/1	1/1	0/0	1/1	1/1	0/0	0/0	1/1	0/0	0/0	1/1	1/1	0/0	0/0	1/1	1/1	0/0	0/0	0/0	1/1	1/1	0/0	0/0	0/0	1/1	0/0	0/0	0/0	0/0	1/1	0/0	0/0	0/0	0/0	1/1	1/1	1/1	1/1	1/1	0/0	1/1	0/0	.	0/0	0/0	1/1	1/1	1/1	0/0	1/1	0/0	1/1	0/0	1/1	0/0	0/0	1/1	1/1	0/0	1/1	.
3L	1084559	3L_1084559_SNP	T	A	.	.	T5EFF=0.004616;T5VAR=8.4682099225809e-05;T5VARACC=0.00413214227377573;T5PVAL=0.9063;T5MIXED=0.508200359478918;T6EFF=-0.2481;T6VAR=0.0697638667384851;T6VARACC=0.000166699692847772;T6PVAL=0.0005847;T6MIXED=0.000741268982990151;AVGEFF=-0.1217;AVGVAR=0.0338217640809422;AVGVARACC=0.000532334355061392;AVGPVAL=0.0177;AVGMIXED=0.0348199817439461;DIFFEFF=0.2527;DIFFVAR=0.123971366176333;DIFFVARACC=0.00464598749335171;DIFFPVAL=3.287e-06;DIFFMIXED=2.6942349582987e-06;ANNOT=(FBgn0004870|bab1|INTRON)	GT	0/0	1/1	1/1	0/0	.	1/1	0/0	1/1	0/0	0/0	1/1	0/0	1/1	0/0	0/0	0/0	1/1	1/1	1/1	0/0	1/1	0/0	0/0	1/1	0/0	0/0	0/0	0/0	0/0	0/0	1/1	1/1	0/0	0/0	0/0	1/1	1/1	1/1	.	1/1	1/1	0/0	0/0	1/1	0/0	0/0	1/1	.	0/0	1/1	1/1	0/0	0/0	0/0	0/0	1/1	0/0	1/1	1/1	1/1	1/1	0/0	1/1	0/0	0/0	0/0	0/0	0/0	1/1	.	0/0	0/0	0/0	0/0	.	1/1	0/0	0/0	.	1/1	1/1	0/0	1/1	1/1	1/1	0/0	1/1	1/1	0/0	0/0	0/0	0/0	0/0	0/0	1/1	0/0	0/0	1/1	0/0	1/1	1/1	0/0	1/1	0/0	1/1	1/1	0/0	1/1	0/0	0/0	0/0	.	0/0	0/0	1/1	0/0	0/0	1/1	1/1	0/0	0/0	1/1	0/0	0/0	1/1	1/1	0/0	0/0	1/1	1/1	0/0	0/0	0/0	1/1	.	0/0	0/0	0/0	1/1	0/0	0/0	0/0	.	1/1	0/0	0/0	1/1	0/0	1/1	1/1	1/1	1/1	1/1	0/0	1/1	0/0	0/0	0/0	0/0	1/1	1/1	0/0	1/1	1/1	0/0	1/1	0/0	1/1	0/0	0/0	1/1	1/1	0/0	1/1	1/1
3L	1084990	3L_1084990_SNP	C	G	.	.	T5EFF=-0.01619;T5VAR=0.00102677419531545;T5VARACC=0.00622419497711041;T5PVAL=0.6765;T5MIXED=0.591831877752074;T6EFF=-0.3395;T6VAR=0.126895671322622;T6VARACC=0.00263921354909591;T6PVAL=1.618e-06;T6MIXED=6.39107489626544e-07;AVGEFF=-0.1779;AVGVAR=0.0707454795767167;AVGVARACC=0.000576066973357825;AVGPVAL=0.0004209;AVGMIXED=0.000135448284975009;DIFFEFF=0.3233;DIFFVAR=0.194014680780864;DIFFVARACC=NA;DIFFPVAL=1.479e-09;DIFFMIXED=7.28573093791763e-10;ANNOT=(FBgn0004870|bab1|INTRON)	GT	1/1	0/0	1/1	1/1	0/0	0/0	1/1	0/0	0/0	0/0	1/1	1/1	0/0	0/0	1/1	1/1	0/0	0/0	1/1	1/1	1/1	0/0	0/0	0/0	0/0	1/1	.	1/1	1/1	1/1	1/1	0/0	1/1	1/1	1/1	0/0	0/0	0/0	1/1	0/0	0/0	1/1	1/1	0/0	1/1	1/1	1/1	.	1/1	1/1	0/0	1/1	1/1	1/1	0/0	0/0	1/1	0/0	0/0	1/1	1/1	1/1	0/0	1/1	1/1	0/0	1/1	1/1	0/0	1/1	1/1	1/1	1/1	1/1	0/0	1/1	1/1	1/1	0/0	1/1	1/1	0/0	0/0	1/1	0/0	1/1	0/0	1/1	1/1	1/1	1/1	1/1	1/1	1/1	1/1	1/1	1/1	0/0	1/1	0/0	1/1	1/1	0/0	1/1	1/1	0/0	1/1	0/0	1/1	1/1	1/1	.	1/1	1/1	1/1	0/0	1/1	0/0	0/0	1/1	0/0	0/0	1/1	1/1	0/0	1/1	1/1	1/1	1/1	1/1	1/1	1/1	1/1	1/1	0/0	1/1	1/1	1/1	1/1	1/1	1/1	1/1	1/1	1/1	1/1	1/1	1/1	1/1	0/0	0/0	0/0	0/0	0/0	0/0	0/0	0/0	1/1	1/1	1/1	1/1	0/0	0/0	0/0	0/0	1/1	0/0	0/0	0/0	1/1	1/1	0/0	0/0	1/1	0/0	1/1
3L	1085137	3L_1085137_SNP	G	T	.	.	T5EFF=-0.05672;T5VAR=0.0129155274364006;T5VARACC=0.000677565751831477;T5PVAL=0.1424;T5MIXED=0.172062213016837;T6EFF=-0.3105;T6VAR=0.107509407127465;T6VARACC=0.00274992701553883;T6PVAL=1.433e-05;T6MIXED=8.60504328471707e-06;AVGEFF=-0.1836;AVGVAR=0.0767073242406158;AVGVARACC=0.000656649622849892;AVGPVAL=0.0002786;AVGMIXED=0.000192358021888457;DIFFEFF=0.2538;DIFFVAR=0.120679273447497;DIFFVARACC=0.00852197289445739;DIFFPVAL=3.958e-06;DIFFMIXED=2.29872660901441e-06;ANNOT=(FBgn0004870|bab1|INTRON)	GT	1/1	0/0	1/1	1/1	0/0	0/0	1/1	0/0	0/0	0/0	1/1	1/1	0/0	0/0	1/1	1/1	0/0	0/0	1/1	1/1	1/1	0/0	0/0	0/0	0/0	1/1	1/1	1/1	1/1	0/0	1/1	0/0	1/1	1/1	1/1	0/0	0/0	0/0	1/1	0/0	0/0	1/1	1/1	0/0	1/1	0/0	.	1/1	1/1	1/1	0/0	1/1	1/1	.	0/0	0/0	.	0/0	0/0	1/1	1/1	1/1	0/0	1/1	1/1	0/0	1/1	1/1	0/0	1/1	1/1	1/1	1/1	1/1	0/0	1/1	1/1	1/1	0/0	1/1	1/1	0/0	0/0	1/1	0/0	1/1	0/0	1/1	1/1	0/0	1/1	1/1	1/1	1/1	0/0	1/1	.	0/0	1/1	0/0	1/1	1/1	0/0	1/1	1/1	0/0	0/0	0/0	1/1	1/1	1/1	.	1/1	1/1	0/0	0/0	1/1	0/0	0/0	1/1	1/1	0/0	1/1	0/0	0/0	1/1	1/1	1/1	1/1	1/1	1/1	1/1	.	1/1	0/0	1/1	1/1	1/1	1/1	1/1	1/1	1/1	1/1	1/1	1/1	1/1	1/1	1/1	1/1	0/0	0/0	0/0	0/0	1/1	0/0	1/1	1/1	1/1	1/1	1/1	0/0	0/0	1/1	0/0	.	0/0	1/1	0/0	1/1	1/1	0/0	0/0	1/1	0/0	0/0
3L	1085230	3L_1085230_SNP	T	G	.	.	T5EFF=-0.065;T5VAR=0.0119461142687448;T5VARACC=1.06845794434207e-08;T5PVAL=0.173;T5MIXED=0.242239921561719;T6EFF=-0.3999;T6VAR=0.12178986611192;T6VARACC=0.000599801463069871;T6PVAL=7.483e-06;T6MIXED=4.84024762542384e-06;AVGEFF=-0.2324;AVGVAR=0.0850775334506382;AVGVARACC=0.0045536751286005;AVGPVAL=0.0002102;AVGMIXED=0.000177143765400324;DIFFEFF=0.3349;DIFFVAR=0.141406017453279;DIFFVARACC=0.00102291992848635;DIFFPVAL=1.213e-06;DIFFMIXED=5.40889191042564e-07;ANNOT=(FBgn0004870|bab1|INTRON)	GT	0/0	1/1	0/0	0/0	.	1/1	0/0	1/1	0/0	0/0	0/0	0/0	1/1	0/0	0/0	0/0	1/1	1/1	0/0	0/0	0/0	0/0	0/0	.	0/0	0/0	0/0	0/0	0/0	0/0	0/0	1/1	0/0	0/0	0/0	0/0	1/1	.	0/0	1/1	.	0/0	0/0	1/1	0/0	0/0	0/0	0/0	0/0	0/0	1/1	0/0	0/0	0/0	0/0	.	0/0	1/1	1/1	0/0	1/1	0/0	1/1	0/0	0/0	0/0	0/0	0/0	.	0/0	0/0	0/0	0/0	0/0	.	0/0	0/0	0/0	.	0/0	0/0	0/0	.	0/0	1/1	0/0	1/1	0/0	0/0	0/0	0/0	0/0	0/0	0/0	1/1	0/0	0/0	.	0/0	1/1	0/0	0/0	1/1	0/0	0/0	.	1/1	1/1	0/0	0/0	0/0	.	0/0	0/0	.	0/0	0/0	1/1	0/0	.	0/0	1/1	0/0	0/0	1/1	0/0	0/0	0/0	0/0	0/0	0/0	0/0	0/0	0/0	1/1	0/0	0/0	0/0	0/0	0/0	0/0	0/0	0/0	0/0	0/0	0/0	0/0	0/0	0/0	1/1	.	1/1	1/1	0/0	1/1	0/0	0/0	0/0	0/0	0/0	.	0/0	0/0	1/1	0/0	.	0/0	0/0	0/0	0/0	.	1/1	0/0	1/1	0/0
3L	1085642	3L_1085642_SNP	T	A	.	.	T5EFF=-0.08569;T5VAR=0.0299109771990398;T5VARACC=0.00617087016656609;T5PVAL=0.02497;T5MIXED=0.0269107458869634;T6EFF=-0.3436;T6VAR=0.143221776810806;T6VARACC=NA;T6PVAL=4.237e-07;T6MIXED=4.59220844701259e-07;AVGEFF=-0.2147;AVGVAR=0.111593208365646;AVGVARACC=NA;AVGPVAL=9.629e-06;AVGMIXED=9.55745669645393e-06;DIFFEFF=0.2579;DIFFVAR=0.136264314652652;DIFFVARACC=0.00125719064208285;DIFFPVAL=8.483e-07;DIFFMIXED=9.40936156172188e-07;ANNOT=(FBgn0004870|bab1|INTRON)	GT	1/1	0/0	1/1	1/1	0/0	0/0	1/1	.	0/0	0/0	1/1	1/1	0/0	0/0	1/1	0/0	0/0	0/0	1/1	1/1	1/1	.	0/0	0/0	0/0	1/1	1/1	0/0	1/1	1/1	0/0	0/0	1/1	1/1	1/1	0/0	0/0	0/0	0/0	0/0	0/0	1/1	1/1	0/0	1/1	1/1	0/0	1/1	1/1	1/1	0/0	.	1/1	1/1	0/0	0/0	1/1	0/0	0/0	1/1	1/1	1/1	0/0	1/1	0/0	0/0	1/1	1/1	0/0	0/0	1/1	1/1	1/1	1/1	0/0	1/1	0/0	1/1	0/0	.	0/0	0/0	0/0	1/1	0/0	1/1	0/0	1/1	1/1	0/0	1/1	1/1	1/1	1/1	0/0	1/1	1/1	0/0	1/1	.	1/1	1/1	0/0	1/1	1/1	0/0	0/0	0/0	1/1	1/1	1/1	.	1/1	0/0	0/0	0/0	1/1	0/0	0/0	1/1	1/1	0/0	1/1	0/0	0/0	0/0	1/1	1/1	0/0	0/0	0/0	1/1	1/1	0/0	0/0	1/1	1/1	0/0	0/0	1/1	1/1	1/1	1/1	1/1	1/1	1/1	1/1	1/1	1/1	0/0	0/0	0/0	0/0	1/1	0/0	1/1	1/1	1/1	.	0/0	0/0	0/0	1/1	0/0	1/1	0/0	1/1	0/0	1/1	1/1	0/0	0/0	1/1	0/0	0/0
3L	1085788	3L_1085788_SNP	G	A	.	.	T5EFF=-0.01341;T5VAR=0.000554157330562235;T5VARACC=0.00316838088515536;T5PVAL=0.7634;T5MIXED=0.913080269905533;T6EFF=-0.3116;T6VAR=0.0821749070889741;T6VARACC=0.00338069178142719;T6PVAL=0.0001809;T6MIXED=0.00015150217825792;AVGEFF=-0.1625;AVGVAR=0.0458151497128793;AVGVARACC=0.00568299965701707;AVGPVAL=0.005621;AVGMIXED=0.0051217898966983;DIFFEFF=0.2982;DIFFVAR=0.125813147583573;DIFFVARACC=0.0156876385157709;DIFFPVAL=2.748e-06;DIFFMIXED=2.26057153963798e-06;ANNOT=(FBgn0004870|bab1|INTRON)	GT	0/0	0/0	0/0	0/0	.	0/0	0/0	1/1	.	0/0	0/0	1/1	1/1	0/0	0/0	0/0	1/1	1/1	0/0	0/0	0/0	0/0	0/0	0/0	0/0	0/0	0/0	0/0	0/0	0/0	0/0	1/1	0/0	0/0	0/0	0/0	1/1	1/1	0/0	0/0	1/1	0/0	0/0	1/1	0/0	0/0	0/0	0/0	0/0	0/0	0/0	0/0	0/0	0/0	0/0	0/0	0/0	1/1	1/1	0/0	0/0	0/0	1/1	1/1	0/0	0/0	1/1	0/0	0/0	0/0	0/0	0/0	0/0	0/0	1/1	0/0	0/0	0/0	0/0	0/0	0/0	0/0	1/1	0/0	1/1	0/0	1/1	0/0	0/0	0/0	0/0	0/0	0/0	0/0	1/1	0/0	0/0	1/1	0/0	1/1	0/0	0/0	0/0	0/0	0/0	1/1	0/0	1/1	0/0	.	0/0	0/0	0/0	0/0	0/0	0/0	1/1	.	0/0	.	1/1	1/1	0/0	0/0	0/0	0/0	1/1	1/1	0/0	0/0	0/0	0/0	0/0	0/0	1/1	0/0	0/0	1/1	0/0	0/0	0/0	0/0	0/0	0/0	0/0	0/0	0/0	0/0	1/1	.	0/0	1/1	.	1/1	0/0	1/1	0/0	1/1	0/0	0/0	0/0	0/0	0/0	1/1	0/0	.	1/1	0/0	0/0	0/0	0/0	.	0/0	0/0	0/0
3L	1086181	3L_1086181_SNP	G	A	.	.	T5EFF=-0.05573;T5VAR=0.0125042444156691;T5VARACC=4.67391374612824e-05;T5PVAL=0.1515;T5MIXED=0.0477699168753591;T6EFF=-0.3151;T6VAR=0.117917222883429;T6VARACC=NA;T6PVAL=5.917e-06;T6MIXED=2.30943533553601e-06;AVGEFF=-0.1854;AVGVAR=0.0818583140673515;AVGVARACC=0.0385278126539216;AVGPVAL=0.0001864;AVGMIXED=3.03512943258764e-05;DIFFEFF=0.2594;DIFFVAR=0.134341354369695;DIFFVARACC=0.00689933269680065;DIFFPVAL=1.193e-06;DIFFMIXED=8.89700140042661e-07;ANNOT=(FBgn0004870|bab1|INTRON)	GT	0/0	1/1	1/1	1/1	1/1	1/1	0/0	0/0	1/1	0/0	0/0	1/1	1/1	.	0/0	1/1	1/1	1/1	0/0	0/0	0/0	0/0	0/0	1/1	0/0	0/0	0/0	0/0	0/0	0/0	1/1	1/1	1/1	1/1	1/1	1/1	1/1	1/1	0/0	1/1	1/1	0/0	1/1	0/0	0/0	0/0	0/0	.	1/1	1/1	0/0	0/0	0/0	1/1	.	.	0/0	1/1	1/1	0/0	0/0	0/0	1/1	1/1	1/1	0/0	1/1	1/1	1/1	0/0	1/1	0/0	0/0	0/0	0/0	1/1	1/1	0/0	.	0/0	.	1/1	1/1	0/0	1/1	0/0	0/0	0/0	1/1	1/1	0/0	1/1	0/0	0/0	1/1	0/0	0/0	1/1	0/0	0/0	0/0	0/0	0/0	0/0	0/0	1/1	1/1	1/1	0/0	0/0	0/0	1/1	0/0	1/1	1/1	1/1	1/1	1/1	1/1	0/0	1/1	1/1	0/0	.	1/1	0/0	.	0/0	0/0	0/0	0/0	0/0	1/1	0/0	1/1	0/0	0/0	0/0	0/0	0/0	0/0	0/0	0/0	0/0	0/0	0/0	0/0	1/1	1/1	1/1	1/1	1/1	0/0	1/1	1/1	1/1	0/0	1/1	0/0	0/0	1/1	1/1	0/0	1/1	1/1	0/0	1/1	0/0	0/0	1/1	1/1	1/1	.	1/1	1/1
3L	1086705	3L_1086705_SNP	T	C	.	.	T5EFF=0.0562;T5VAR=0.012030879252316;T5VARACC=0.000452687071950009;T5PVAL=0.1557;T5MIXED=0.169215815955417;T6EFF=0.3088;T6VAR=0.10327052317259;T6VARACC=0.00993968432498232;T6PVAL=2.042e-05;T6MIXED=1.09739951441677e-05;AVGEFF=0.1825;AVGVAR=0.0732916876197483;AVGVARACC=0.0142608488293356;AVGPVAL=0.0003708;AVGMIXED=0.000172803434208776;DIFFEFF=-0.2526;DIFFVAR=0.115178397926282;DIFFVARACC=3.53848156416925e-05;DIFFPVAL=6.365e-06;DIFFMIXED=3.86834434038492e-06;ANNOT=(FBgn0004870|bab1|INTRON)	GT	0/0	1/1	1/1	1/1	1/1	1/1	0/0	1/1	1/1	0/0	1/1	1/1	1/1	1/1	0/0	.	1/1	1/1	0/0	0/0	0/0	0/0	.	1/1	0/0	0/0	0/0	1/1	1/1	1/1	1/1	1/1	1/1	1/1	1/1	1/1	1/1	1/1	.	1/1	1/1	0/0	0/0	1/1	0/0	1/1	1/1	1/1	1/1	1/1	1/1	0/0	0/0	0/0	1/1	0/0	1/1	1/1	1/1	.	1/1	0/0	1/1	1/1	1/1	0/0	1/1	1/1	0/0	1/1	1/1	0/0	0/0	0/0	0/0	1/1	0/0	0/0	1/1	1/1	1/1	1/1	1/1	0/0	1/1	1/1	1/1	1/1	0/0	0/0	.	1/1	0/0	1/1	1/1	0/0	0/0	1/1	0/0	1/1	1/1	0/0	1/1	1/1	1/1	1/1	1/1	1/1	0/0	1/1	0/0	.	0/0	1/1	1/1	1/1	1/1	1/1	1/1	0/0	1/1	1/1	0/0	0/0	1/1	1/1	1/1	0/0	1/1	1/1	0/0	1/1	1/1	1/1	1/1	0/0	0/0	0/0	0/0	1/1	0/0	0/0	1/1	1/1	0/0	0/0	1/1	1/1	1/1	1/1	0/0	1/1	1/1	1/1	1/1	1/1	0/0	1/1	0/0	1/1	0/0	1/1	0/0	1/1	1/1	1/1	1/1	1/1	0/0	0/0	0/0	1/1	0/0	1/1	1/1
3L	1086740	3L_1086740_SNP	G	A	.	.	T5EFF=0.03032;T5VAR=0.00289592971576085;T5VARACC=0.00329869403928945;T5PVAL=0.4858;T5MIXED=0.369216230111688;T6EFF=0.3307;T6VAR=0.0972352884944273;T6VARACC=0.00950386613908555;T6PVAL=3.482e-05;T6MIXED=1.77511892840855e-05;AVGEFF=0.1805;AVGVAR=0.058411977782611;AVGVARACC=0.0146369477592609;AVGPVAL=0.001498;AVGMIXED=0.000600792044198122;DIFFEFF=-0.3004;DIFFVAR=0.138163529350648;DIFFVARACC=NA;DIFFPVAL=6.018e-07;DIFFMIXED=4.69645447625156e-07;ANNOT=(FBgn0004870|bab1|INTRON)	GT	0/0	1/1	1/1	1/1	1/1	1/1	0/0	1/1	1/1	0/0	1/1	1/1	1/1	1/1	0/0	1/1	1/1	1/1	0/0	0/0	0/0	0/0	.	1/1	0/0	0/0	0/0	1/1	1/1	1/1	1/1	1/1	1/1	1/1	1/1	1/1	1/1	1/1	1/1	1/1	1/1	0/0	1/1	1/1	0/0	1/1	1/1	1/1	1/1	1/1	1/1	0/0	1/1	1/1	1/1	1/1	1/1	1/1	1/1	1/1	1/1	0/0	1/1	1/1	1/1	0/0	1/1	1/1	1/1	1/1	1/1	0/0	0/0	0/0	0/0	1/1	1/1	0/0	1/1	1/1	1/1	1/1	1/1	0/0	1/1	1/1	1/1	1/1	1/1	1/1	.	1/1	0/0	1/1	0/0	0/0	0/0	1/1	0/0	1/1	1/1	0/0	1/1	1/1	1/1	1/1	1/1	1/1	.	1/1	0/0	1/1	0/0	1/1	1/1	1/1	1/1	1/1	1/1	0/0	1/1	1/1	0/0	1/1	1/1	1/1	1/1	0/0	1/1	1/1	0/0	1/1	1/1	1/1	.	0/0	0/0	0/0	1/1	1/1	0/0	0/0	1/1	1/1	.	0/0	1/1	1/1	1/1	0/0	1/1	1/1	1/1	1/1	1/1	1/1	0/0	1/1	0/0	1/1	1/1	1/1	1/1	1/1	1/1	1/1	1/1	1/1	0/0	1/1	1/1	1/1	1/1	1/1	1/1
3L	1086767	3L_1086767_SNP	A	G	.	.	T5EFF=0.03051;T5VAR=0.00337490821711932;T5VARACC=0.00321232367607177;T5PVAL=0.4477;T5MIXED=0.427055680991652;T6EFF=0.3182;T6VAR=0.104028331108314;T6VARACC=0.00700660870957498;T6PVAL=1.506e-05;T6MIXED=7.44826045969954e-06;AVGEFF=0.1744;AVGVAR=0.0632579619153583;AVGVARACC=0.00998824928278463;AVGPVAL=0.000844;AVGMIXED=0.000370956275378705;DIFFEFF=-0.2877;DIFFVAR=0.143710891884226;DIFFVARACC=7.30510871032042e-05;DIFFPVAL=2.699e-07;DIFFMIXED=1.65962344574677e-07;ANNOT=(FBgn0004870|bab1|INTRON)	GT	0/0	1/1	1/1	1/1	1/1	1/1	0/0	1/1	1/1	0/0	1/1	1/1	1/1	1/1	0/0	1/1	1/1	1/1	0/0	0/0	0/0	0/0	.	1/1	0/0	0/0	0/0	1/1	1/1	1/1	1/1	1/1	1/1	1/1	1/1	1/1	1/1	1/1	1/1	1/1	1/1	0/0	1/1	1/1	0/0	1/1	1/1	1/1	1/1	1/1	1/1	0/0	0/0	0/0	1/1	1/1	1/1	1/1	1/1	1/1	1/1	0/0	1/1	1/1	1/1	0/0	1/1	1/1	1/1	1/1	1/1	0/0	0/0	0/0	0/0	1/1	0/0	0/0	1/1	1/1	1/1	1/1	1/1	0/0	1/1	1/1	1/1	1/1	0/0	0/0	.	1/1	0/0	1/1	0/0	0/0	0/0	1/1	0/0	1/1	1/1	0/0	1/1	1/1	1/1	1/1	1/1	1/1	0/0	0/0	0/0	1/1	0/0	1/1	1/1	1/1	1/1	1/1	1/1	0/0	1/1	1/1	0/0	0/0	1/1	1/1	1/1	0/0	1/1	1/1	0/0	1/1	1/1	1/1	1/1	0/0	0/0	0/0	1/1	1/1	0/0	0/0	1/1	1/1	0/0	0/0	1/1	1/1	1/1	0/0	1/1	1/1	1/1	1/1	1/1	1/1	0/0	1/1	0/0	1/1	1/1	1/1	0/0	1/1	1/1	1/1	1/1	1/1	0/0	0/0	1/1	1/1	0/0	1/1	1/1
3L	1086784	3L_1086784_SNP	G	A	.	.	T5EFF=0.01097;T5VAR=0.000440795994315803;T5VARACC=0.00600348062309664;T5PVAL=0.7858;T5MIXED=0.737098318456494;T6EFF=0.2848;T6VAR=0.0845164756989153;T6VARACC=0.00491044047836114;T6PVAL=0.0001201;T6MIXED=6.27012905748893e-05;AVGEFF=0.1479;AVGVAR=0.0462832420207464;AVGVARACC=0.00634837440598079;AVGPVAL=0.004842;AVGMIXED=0.00210865317306561;DIFFEFF=-0.2738;DIFFVAR=0.13025505591844;DIFFVARACC=0.000262646002518019;DIFFPVAL=1.332e-06;DIFFMIXED=8.16319407603701e-07;ANNOT=(FBgn0004870|bab1|INTRON)	GT	0/0	1/1	1/1	1/1	1/1	1/1	0/0	1/1	1/1	0/0	1/1	1/1	1/1	1/1	0/0	.	1/1	1/1	0/0	0/0	0/0	0/0	0/0	1/1	0/0	0/0	0/0	1/1	1/1	.	1/1	1/1	1/1	1/1	1/1	1/1	1/1	1/1	1/1	1/1	1/1	0/0	1/1	1/1	0/0	1/1	1/1	1/1	.	1/1	1/1	0/0	0/0	0/0	1/1	1/1	1/1	1/1	1/1	.	1/1	0/0	1/1	1/1	1/1	0/0	1/1	1/1	1/1	1/1	1/1	0/0	0/0	1/1	0/0	1/1	0/0	1/1	1/1	1/1	1/1	1/1	1/1	0/0	1/1	1/1	1/1	1/1	0/0	0/0	0/0	1/1	0/0	1/1	0/0	0/0	0/0	1/1	0/0	1/1	1/1	0/0	1/1	1/1	1/1	1/1	1/1	1/1	0/0	0/0	.	1/1	0/0	1/1	1/1	1/1	1/1	1/1	1/1	0/0	1/1	1/1	0/0	0/0	1/1	1/1	1/1	0/0	1/1	1/1	0/0	1/1	1/1	1/1	1/1	0/0	0/0	0/0	1/1	1/1	0/0	0/0	1/1	1/1	0/0	0/0	1/1	1/1	1/1	0/0	1/1	1/1	1/1	1/1	1/1	1/1	0/0	1/1	0/0	1/1	1/1	1/1	0/0	1/1	1/1	1/1	1/1	1/1	0/0	0/0	1/1	1/1	0/0	1/1	1/1
3L	1086799	3L_1086799_SNP	A	C	.	.	T5EFF=0.02796;T5VAR=0.00275741707792815;T5VARACC=0.00281399693957526;T5PVAL=0.4952;T5MIXED=0.535812125159314;T6EFF=0.3003;T6VAR=0.0901421355445498;T6VARACC=0.00508814613061809;T6PVAL=6.614e-05;T6MIXED=3.9547618382283e-05;AVGEFF=0.1642;AVGVAR=0.0546638474989479;AVGVARACC=0.00864658886983422;AVGPVAL=0.002086;AVGMIXED=0.00117746235531284;DIFFEFF=-0.2724;DIFFVAR=0.124313884933328;DIFFVARACC=7.63594844732651e-06;DIFFPVAL=2.25e-06;DIFFMIXED=1.58441573912258e-06;ANNOT=(FBgn0004870|bab1|INTRON)	GT	0/0	1/1	1/1	1/1	1/1	1/1	0/0	1/1	1/1	0/0	1/1	1/1	1/1	1/1	0/0	1/1	1/1	1/1	0/0	0/0	0/0	0/0	0/0	1/1	0/0	0/0	0/0	1/1	1/1	1/1	1/1	1/1	1/1	1/1	1/1	1/1	1/1	1/1	1/1	1/1	1/1	0/0	1/1	1/1	0/0	1/1	1/1	1/1	1/1	1/1	1/1	0/0	0/0	.	1/1	1/1	1/1	1/1	1/1	1/1	1/1	0/0	1/1	1/1	1/1	0/0	1/1	1/1	1/1	1/1	1/1	0/0	0/0	1/1	0/0	1/1	0/0	1/1	1/1	1/1	1/1	1/1	1/1	0/0	1/1	1/1	1/1	1/1	0/0	0/0	0/0	1/1	0/0	1/1	1/1	0/0	0/0	1/1	0/0	1/1	1/1	0/0	1/1	1/1	1/1	.	1/1	1/1	.	0/0	0/0	1/1	0/0	1/1	1/1	1/1	1/1	1/1	1/1	0/0	1/1	1/1	0/0	0/0	1/1	1/1	1/1	0/0	1/1	1/1	0/0	1/1	1/1	1/1	1/1	0/0	0/0	0/0	1/1	1/1	0/0	0/0	1/1	1/1	0/0	0/0	1/1	1/1	1/1	1/1	1/1	1/1	1/1	1/1	1/1	1/1	0/0	1/1	0/0	1/1	1/1	1/1	0/0	1/1	1/1	1/1	1/1	1/1	0/0	0/0	1/1	1/1	.	1/1	1/1
3L	1086802	3L_1086802_INS	TC	CTAT	.	.	T5EFF=0.02579;T5VAR=0.00243340336438966;T5VARACC=0.00305679171418594;T5PVAL=0.518;T5MIXED=0.560174914250972;T6EFF=0.3032;T6VAR=0.0940089262334136;T6VARACC=0.00390474139177399;T6PVAL=3.874e-05;T6MIXED=2.2813587286105e-05;AVGEFF=0.1645;AVGVAR=0.056506426410754;AVGVARACC=0.00789818244981311;AVGPVAL=0.001586;AVGMIXED=0.000925704295904029;DIFFEFF=-0.2775;DIFFVAR=0.131162895352292;DIFFVARACC=3.78178995846801e-05;DIFFPVAL=9.081e-07;DIFFMIXED=5.71330889461822e-07;ANNOT=(FBgn0004870|bab1|INTRON)	GT	0/0	1/1	1/1	1/1	1/1	1/1	0/0	1/1	1/1	0/0	1/1	1/1	1/1	1/1	0/0	1/1	1/1	1/1	0/0	0/0	0/0	0/0	0/0	1/1	0/0	0/0	0/0	1/1	1/1	1/1	1/1	1/1	1/1	1/1	1/1	1/1	1/1	1/1	1/1	1/1	1/1	0/0	1/1	1/1	0/0	1/1	1/1	1/1	1/1	1/1	1/1	0/0	0/0	0/0	1/1	1/1	1/1	1/1	1/1	1/1	1/1	0/0	1/1	1/1	1/1	0/0	1/1	1/1	1/1	1/1	1/1	0/0	0/0	1/1	0/0	1/1	0/0	1/1	1/1	1/1	1/1	1/1	1/1	0/0	1/1	1/1	1/1	1/1	0/0	0/0	0/0	1/1	0/0	1/1	1/1	0/0	0/0	1/1	0/0	1/1	1/1	0/0	1/1	1/1	1/1	.	1/1	1/1	0/0	0/0	0/0	1/1	0/0	1/1	1/1	1/1	1/1	1/1	1/1	0/0	1/1	1/1	0/0	0/0	1/1	1/1	1/1	0/0	1/1	1/1	0/0	1/1	1/1	1/1	1/1	0/0	0/0	0/0	1/1	1/1	0/0	0/0	1/1	1/1	0/0	0/0	1/1	1/1	1/1	1/1	1/1	1/1	1/1	1/1	1/1	1/1	0/0	1/1	0/0	1/1	1/1	1/1	0/0	1/1	1/1	1/1	1/1	1/1	0/0	0/0	1/1	1/1	0/0	1/1	1/1
3L	1090129	3L_1090129_SNP	A	G	.	.	T5EFF=0.01582;T5VAR=0.00100099760007721;T5VARACC=0.00273393785237158;T5PVAL=0.6848;T5MIXED=0.399031634729457;T6EFF=0.2536;T6VAR=0.0745814234905645;T6VARACC=0.00432778464761174;T6PVAL=0.0003559;T6MIXED=0.000170621964719798;AVGEFF=0.1347;AVGVAR=0.0424104222158707;AVGVARACC=0.00165787145306584;AVGPVAL=0.007585;AVGMIXED=0.00206935873402524;DIFFEFF=-0.2378;DIFFVAR=0.110225017826399;DIFFVARACC=0.00789391451779597;DIFFPVAL=1.17e-05;DIFFMIXED=8.16072254148465e-06;ANNOT=(FBgn0004870|bab1|INTRON)	GT	0/0	1/1	0/0	0/0	1/1	1/1	0/0	1/1	1/1	0/0	1/1	0/0	1/1	0/0	1/1	1/1	1/1	.	0/0	0/0	.	0/0	0/0	1/1	0/0	0/0	0/0	1/1	0/0	0/0	.	1/1	.	0/0	1/1	1/1	1/1	1/1	1/1	1/1	1/1	0/0	1/1	1/1	0/0	0/0	1/1	1/1	1/1	0/0	1/1	0/0	0/0	0/0	1/1	1/1	1/1	1/1	1/1	0/0	0/0	1/1	1/1	1/1	1/1	0/0	0/0	0/0	0/0	1/1	0/0	0/0	0/0	.	0/0	0/0	0/0	1/1	1/1	1/1	1/1	1/1	0/0	0/0	.	1/1	1/1	0/0	0/0	0/0	0/0	0/0	0/0	1/1	1/1	1/1	0/0	1/1	1/1	1/1	0/0	1/1	1/1	1/1	0/0	0/0	1/1	1/1	1/1	0/0	0/0	1/1	0/0	0/0	0/0	1/1	0/0	1/1	1/1	0/0	1/1	0/0	0/0	0/0	1/1	1/1	0/0	1/1	1/1	1/1	0/0	1/1	1/1	1/1	1/1	.	0/0	0/0	1/1	0/0	0/0	0/0	1/1	1/1	1/1	0/0	1/1	1/1	1/1	1/1	1/1	1/1	1/1	1/1	1/1	1/1	.	0/0	0/0	1/1	1/1	0/0	1/1	1/1	1/1	0/0	1/1	0/0	0/0	0/0	1/1	1/1	0/0	1/1	0/0
3L	1093297	3L_1093297_SNP	T	A	.	.	T5EFF=-0.001529;T5VAR=7.3419495572811e-06;T5VARACC=0.00429116309017258;T5PVAL=0.9719;T5MIXED=0.876311503189609;T6EFF=-0.3028;T6VAR=0.0825233616490134;T6VARACC=0.0187967399913241;T6PVAL=0.000133;T6MIXED=7.98566581934195e-05;AVGEFF=-0.1522;AVGVAR=0.0421344761944164;AVGVARACC=0.0142433501863933;AVGPVAL=0.006908;AVGMIXED=0.00308116643772458;DIFFEFF=0.3012;DIFFVAR=0.137387218415534;DIFFVARACC=0.0266839906487675;DIFFPVAL=5.582e-07;DIFFMIXED=3.75037091455337e-07;ANNOT=(FBgn0004870|bab1|INTRON)	GT	0/0	0/0	0/0	0/0	1/1	0/0	0/0	0/0	0/0	1/1	0/0	0/0	0/0	0/0	0/0	1/1	1/1	1/1	0/0	0/0	0/0	1/1	.	0/0	0/0	0/0	0/0	1/1	1/1	0/0	0/0	1/1	1/1	1/1	1/1	0/0	1/1	0/0	0/0	1/1	0/0	0/0	0/0	1/1	0/0	0/0	1/1	0/0	0/0	0/0	1/1	0/0	0/0	0/0	0/0	1/1	0/0	0/0	0/0	0/0	0/0	0/0	1/1	1/1	0/0	0/0	0/0	0/0	0/0	0/0	0/0	0/0	1/1	0/0	0/0	0/0	0/0	0/0	0/0	1/1	0/0	0/0	0/0	0/0	1/1	.	0/0	0/0	0/0	0/0	0/0	0/0	0/0	0/0	1/1	0/0	0/0	0/0	0/0	1/1	0/0	0/0	0/0	0/0	0/0	0/0	0/0	1/1	0/0	0/0	0/0	0/0	0/0	0/0	0/0	0/0	0/0	1/1	1/1	0/0	1/1	0/0	0/0	0/0	.	1/1	0/0	0/0	0/0	0/0	0/0	0/0	0/0	0/0	1/1	0/0	0/0	0/0	0/0	0/0	0/0	0/0	0/0	0/0	1/1	0/0	0/0	0/0	0/0	0/0	0/0	1/1	1/1	1/1	1/1	1/1	0/0	1/1	0/0	0/0	0/0	1/1	1/1	0/0	0/0	0/0	1/1	0/0	0/0	0/0	1/1	1/1	0/0	1/1	0/0
3L	1099962	3L_1099962_SNP	G	C	.	.	T5EFF=-0.2313;T5VAR=0.113386175200556;T5VARACC=NA;T5PVAL=8.084e-06;T5MIXED=1.63008824220389e-06;T6EFF=-0.3161;T6VAR=0.0608696128431579;T6VARACC=0.0242583195773807;T6PVAL=0.001264;T6MIXED=0.00111603345058492;AVGEFF=-0.2737;AVGVAR=0.0915795123284728;AVGVARACC=0.0537852014209915;AVGPVAL=6.691e-05;AVGMIXED=3.57918251859429e-05;DIFFEFF=0.08482;DIFFVAR=0.00753301933096357;DIFFVARACC=0.00298641181051568;DIFFPVAL=0.2633;DIFFMIXED=0.265142837051551;ANNOT=(FBgn0004870|bab1|SYNONYMOUS_CODING)	GT	0/0	0/0	0/0	0/0	0/0	0/0	0/0	0/0	0/0	1/1	0/0	0/0	0/0	1/1	0/0	0/0	0/0	0/0	0/0	0/0	0/0	0/0	0/0	0/0	0/0	0/0	0/0	0/0	0/0	0/0	0/0	0/0	.	1/1	0/0	0/0	0/0	1/1	0/0	0/0	0/0	.	0/0	0/0	0/0	0/0	0/0	1/1	1/1	0/0	0/0	0/0	0/0	.	0/0	0/0	0/0	1/1	0/0	0/0	0/0	0/0	1/1	1/1	0/0	0/0	0/0	0/0	1/1	0/0	0/0	0/0	0/0	0/0	0/0	0/0	0/0	0/0	0/0	0/0	0/0	0/0	1/1	0/0	0/0	0/0	0/0	0/0	.	0/0	0/0	0/0	0/0	1/1	0/0	0/0	0/0	.	0/0	0/0	0/0	0/0	0/0	0/0	0/0	1/1	1/1	0/0	0/0	1/1	0/0	0/0	0/0	1/1	0/0	0/0	1/1	0/0	0/0	0/0	0/0	1/1	0/0	0/0	1/1	0/0	0/0	0/0	0/0	0/0	0/0	0/0	0/0	0/0	0/0	0/0	0/0	0/0	0/0	0/0	0/0	0/0	0/0	0/0	.	0/0	0/0	1/1	0/0	0/0	0/0	0/0	0/0	0/0	1/1	0/0	0/0	0/0	0/0	0/0	0/0	0/0	1/1	0/0	1/1	0/0	0/0	1/1	0/0	0/0	0/0	1/1	.	1/1	0/0
3L	11132623	3L_11132623_SNP	A	G	.	.	T5EFF=0.08903;T5VAR=0.00736276548301161;T5VARACC=0.000847536369208701;T5PVAL=0.2688;T5MIXED=0.145947199736001;T6EFF=0.5853;T6VAR=0.0888367160042044;T6VARACC=0.0152182677663584;T6PVAL=8.711e-05;T6MIXED=4.13370846021591e-05;AVGEFF=0.3372;AVGVAR=0.0601730952771127;AVGVARACC=0.0117906041067129;AVGPVAL=0.001351;AVGMIXED=0.000388213518296834;DIFFEFF=-0.4962;DIFFVAR=0.106666251257012;DIFFVARACC=0.0229607404707304;DIFFPVAL=1.556e-05;DIFFMIXED=9.14203683065007e-06;ANNOT=(FBgn0052082|CG32082|INTRON)	GT	0/0	0/0	0/0	0/0	0/0	0/0	1/1	0/0	0/0	0/0	0/0	0/0	0/0	0/0	0/0	0/0	0/0	0/0	0/0	0/0	0/0	0/0	0/0	0/0	0/0	0/0	0/0	0/0	0/0	0/0	0/0	0/0	.	0/0	0/0	0/0	0/0	0/0	0/0	0/0	0/0	1/1	0/0	0/0	0/0	0/0	0/0	0/0	0/0	0/0	.	0/0	0/0	0/0	0/0	0/0	0/0	0/0	0/0	1/1	0/0	1/1	0/0	0/0	0/0	0/0	0/0	0/0	0/0	0/0	0/0	0/0	.	1/1	0/0	0/0	.	.	0/0	0/0	0/0	0/0	0/0	1/1	0/0	0/0	0/0	0/0	0/0	0/0	0/0	0/0	0/0	0/0	0/0	0/0	0/0	0/0	0/0	0/0	0/0	0/0	0/0	1/1	1/1	0/0	0/0	0/0	0/0	0/0	0/0	0/0	0/0	0/0	0/0	0/0	0/0	0/0	0/0	0/0	0/0	0/0	0/0	0/0	0/0	0/0	0/0	0/0	0/0	0/0	0/0	0/0	0/0	0/0	0/0	0/0	0/0	0/0	0/0	0/0	.	1/1	0/0	0/0	0/0	0/0	0/0	0/0	0/0	0/0	0/0	0/0	0/0	0/0	0/0	0/0	0/0	0/0	0/0	0/0	0/0	0/0	0/0	0/0	0/0	0/0	0/0	0/0	1/1	0/0	0/0	0/0	0/0	.	0/0
3L	1120350	3L_1120350_SNP	C	T	.	.	T5EFF=-0.2862;T5VAR=0.0872785448145448;T5VARACC=0.0685487315202087;T5PVAL=7.214e-05;T5MIXED=3.56325076408156e-05;T6EFF=-0.5389;T6VAR=0.0879072408451667;T6VARACC=0.0587611282724532;T6PVAL=6.775e-05;T6MIXED=3.0888379312502e-05;AVGEFF=-0.4126;AVGVAR=0.104307039745325;AVGVARACC=0.0915261374980009;AVGPVAL=1.305e-05;AVGMIXED=3.09766131620697e-06;DIFFEFF=0.2527;DIFFVAR=0.0326171095519025;DIFFVARACC=0.00500611073980339;DIFFPVAL=0.01677;DIFFMIXED=0.0145452553873573;ANNOT=(FBgn0004870|bab1|19261 bp UPSTREAM),(FBgn0025525|bab2|20079 bp UPSTREAM)	GT	0/0	0/0	0/0	1/1	0/0	1/1	0/0	0/0	0/0	1/1	0/0	0/0	0/0	0/0	0/0	0/0	0/0	0/0	0/0	0/0	0/0	0/0	0/0	0/0	0/0	0/0	0/0	0/0	0/0	0/0	0/0	0/0	0/0	0/0	0/0	0/0	0/0	0/0	0/0	1/1	0/0	0/0	0/0	0/0	0/0	0/0	0/0	0/0	0/0	0/0	0/0	0/0	0/0	0/0	0/0	0/0	0/0	0/0	0/0	0/0	0/0	0/0	0/0	0/0	0/0	0/0	0/0	0/0	0/0	0/0	0/0	0/0	0/0	0/0	0/0	0/0	0/0	0/0	0/0	0/0	0/0	0/0	0/0	0/0	0/0	1/1	0/0	0/0	0/0	0/0	0/0	0/0	0/0	0/0	0/0	0/0	0/0	0/0	0/0	0/0	0/0	0/0	0/0	0/0	0/0	0/0	0/0	0/0	0/0	0/0	0/0	0/0	0/0	1/1	0/0	0/0	0/0	0/0	0/0	0/0	1/1	0/0	0/0	0/0	0/0	0/0	0/0	0/0	0/0	0/0	0/0	0/0	1/1	0/0	0/0	0/0	0/0	0/0	0/0	0/0	0/0	0/0	0/0	0/0	0/0	0/0	0/0	0/0	0/0	0/0	0/0	0/0	0/0	1/1	0/0	1/1	0/0	0/0	0/0	0/0	0/0	0/0	0/0	0/0	0/0	1/1	1/1	0/0	0/0	0/0	0/0	0/0	0/0	0/0	0/0
3L	1120423	3L_1120423_SNP	T	A	.	.	T5EFF=-0.2395;T5VAR=0.0840597549215386;T5VARACC=0.0846708027624203;T5PVAL=0.0001092;T5MIXED=4.50447335957216e-05;T6EFF=-0.4388;T6VAR=0.0805840318683791;T6VARACC=0.0695269055890246;T6PVAL=0.0001538;T6MIXED=7.71882964493899e-05;AVGEFF=-0.3391;AVGVAR=0.0971757382030835;AVGVARACC=0.128922009181099;AVGPVAL=2.98e-05;AVGMIXED=7.87884486958854e-06;DIFFEFF=0.1992;DIFFVAR=0.028183791598803;DIFFVARACC=0.0165055608349637;DIFFPVAL=0.02726;DIFFMIXED=0.0242451868417765;ANNOT=(FBgn0004870|bab1|19334 bp UPSTREAM),(FBgn0025525|bab2|20006 bp UPSTREAM)	GT	0/0	0/0	0/0	1/1	0/0	1/1	0/0	0/0	0/0	0/0	0/0	0/0	0/0	0/0	0/0	0/0	0/0	0/0	0/0	0/0	0/0	0/0	0/0	0/0	0/0	0/0	0/0	0/0	0/0	0/0	0/0	0/0	0/0	0/0	0/0	0/0	0/0	0/0	0/0	1/1	.	0/0	0/0	0/0	0/0	0/0	0/0	0/0	0/0	0/0	0/0	0/0	1/1	1/1	0/0	0/0	0/0	0/0	0/0	0/0	0/0	0/0	0/0	0/0	0/0	0/0	0/0	0/0	0/0	.	0/0	0/0	0/0	0/0	0/0	0/0	1/1	0/0	0/0	0/0	0/0	0/0	0/0	0/0	0/0	1/1	0/0	0/0	1/1	0/0	0/0	0/0	0/0	0/0	0/0	0/0	0/0	0/0	0/0	0/0	0/0	0/0	0/0	0/0	0/0	0/0	0/0	0/0	0/0	0/0	0/0	0/0	0/0	1/1	0/0	0/0	0/0	0/0	0/0	0/0	1/1	0/0	0/0	0/0	0/0	0/0	0/0	0/0	0/0	0/0	0/0	0/0	1/1	0/0	0/0	0/0	0/0	0/0	0/0	0/0	0/0	0/0	0/0	0/0	0/0	0/0	0/0	1/1	0/0	0/0	0/0	0/0	0/0	1/1	0/0	1/1	0/0	0/0	0/0	0/0	0/0	0/0	0/0	0/0	1/1	0/0	1/1	0/0	0/0	0/0	0/0	0/0	0/0	0/0	1/1
3L	11453823	3L_11453823_SNP	C	T	.	.	T5EFF=-0.2813;T5VAR=0.135956688135144;T5VARACC=0.0860458970883278;T5PVAL=8.108e-07;T5MIXED=7.58091679737517e-07;T6EFF=-0.3106;T6VAR=0.0477071175085857;T6VARACC=0.0451223162566251;T6PVAL=0.004332;T6MIXED=0.00607209043287906;AVGEFF=-0.296;AVGVAR=0.0874289912396005;AVGVARACC=0.0616332210058288;AVGPVAL=9.501e-05;AVGMIXED=0.00028079890785293;DIFFEFF=0.02923;DIFFVAR=0.000711633286628174;DIFFVARACC=0.00128004005302856;DIFFPVAL=0.7306;DIFFMIXED=0.745331571309891;ANNOT=(FBgn0036161|CG7551|1098 bp DOWNSTREAM),(FBgn0036162|CG6140|2847 bp UPSTREAM)	GT	.	1/1	1/1	1/1	1/1	1/1	1/1	1/1	1/1	0/0	1/1	1/1	1/1	1/1	1/1	0/0	1/1	0/0	1/1	0/0	1/1	1/1	1/1	1/1	1/1	1/1	1/1	1/1	1/1	.	1/1	1/1	.	1/1	1/1	1/1	0/0	1/1	1/1	1/1	1/1	1/1	1/1	1/1	1/1	1/1	1/1	1/1	1/1	1/1	.	1/1	1/1	1/1	0/0	1/1	1/1	1/1	1/1	1/1	1/1	.	1/1	1/1	1/1	1/1	1/1	1/1	1/1	1/1	1/1	1/1	1/1	1/1	1/1	1/1	1/1	1/1	1/1	1/1	0/0	1/1	1/1	0/0	1/1	1/1	1/1	1/1	1/1	1/1	1/1	1/1	1/1	1/1	1/1	1/1	1/1	1/1	1/1	1/1	1/1	0/0	1/1	1/1	1/1	1/1	0/0	1/1	1/1	1/1	1/1	1/1	1/1	0/0	1/1	1/1	1/1	1/1	1/1	1/1	1/1	1/1	1/1	1/1	0/0	0/0	1/1	1/1	1/1	1/1	1/1	.	1/1	1/1	1/1	1/1	1/1	1/1	0/0	1/1	1/1	1/1	1/1	1/1	1/1	0/0	1/1	0/0	1/1	1/1	1/1	1/1	1/1	1/1	1/1	1/1	0/0	1/1	1/1	0/0	1/1	1/1	1/1	1/1	0/0	0/0	1/1	1/1	1/1	1/1	1/1	1/1	1/1	1/1	1/1
3L	1230773	3L_1230773_SNP	C	A	.	.	T5EFF=0.1909;T5VAR=0.0388152202392387;T5VARACC=0.0195816415536108;T5PVAL=0.009375;T5MIXED=0.0106951276401776;T6EFF=0.6363;T6VAR=0.122686793990286;T6VARACC=0.118626124997823;T6PVAL=2.311e-06;T6MIXED=1.47878203819475e-06;AVGEFF=0.4136;AVGVAR=0.104894730401293;AVGVARACC=0.0705254588518948;AVGPVAL=1.382e-05;AVGMIXED=1.08039653940695e-05;DIFFEFF=-0.4454;DIFFVAR=0.10151986804461;DIFFVARACC=0.0873530702648247;DIFFPVAL=1.935e-05;DIFFMIXED=1.33683369279598e-05;ANNOT=(FBgn0264272|mwh|SYNONYMOUS_CODING)	GT	0/0	0/0	1/1	0/0	1/1	0/0	1/1	0/0	0/0	0/0	0/0	0/0	0/0	0/0	0/0	0/0	0/0	0/0	0/0	0/0	0/0	0/0	.	0/0	1/1	0/0	0/0	0/0	0/0	0/0	0/0	0/0	0/0	0/0	0/0	0/0	0/0	0/0	0/0	0/0	0/0	0/0	0/0	0/0	0/0	0/0	0/0	0/0	0/0	1/1	0/0	0/0	0/0	0/0	0/0	0/0	0/0	0/0	0/0	0/0	0/0	0/0	0/0	1/1	0/0	0/0	0/0	0/0	0/0	0/0	0/0	0/0	0/0	0/0	0/0	0/0	0/0	0/0	0/0	0/0	0/0	1/1	0/0	0/0	0/0	0/0	0/0	0/0	0/0	0/0	0/0	0/0	0/0	0/0	0/0	0/0	0/0	0/0	0/0	0/0	0/0	0/0	0/0	0/0	0/0	0/0	0/0	0/0	0/0	0/0	0/0	0/0	0/0	0/0	0/0	0/0	0/0	0/0	0/0	1/1	0/0	0/0	0/0	0/0	0/0	0/0	0/0	0/0	0/0	0/0	0/0	0/0	0/0	0/0	0/0	0/0	0/0	0/0	0/0	0/0	0/0	0/0	0/0	0/0	0/0	0/0	.	0/0	0/0	0/0	1/1	0/0	0/0	0/0	0/0	0/0	0/0	0/0	0/0	0/0	1/1	0/0	0/0	0/0	0/0	0/0	0/0	0/0	0/0	1/1	0/0	0/0	1/1	0/0	0/0
3L	1242674	3L_1242674_SNP	T	A	.	.	T5EFF=0.1012;T5VAR=0.0362610473608044;T5VARACC=0.0270806066675401;T5PVAL=0.01209;T5MIXED=0.01431238598178;T6EFF=0.3363;T6VAR=0.113863374793188;T6VARACC=0.0889207556484553;T6PVAL=5.626e-06;T6MIXED=4.29254747790704e-06;AVGEFF=0.2187;AVGVAR=0.0975151631733768;AVGVARACC=0.0885079720254006;AVGPVAL=2.882e-05;AVGMIXED=3.13336421068548e-05;DIFFEFF=-0.2351;DIFFVAR=0.0939188521705035;DIFFVARACC=0.0632039235582325;DIFFPVAL=4.118e-05;DIFFMIXED=3.08070730069549e-05;ANNOT=(FBgn0004378|Klp61F|SYNONYMOUS_CODING),(FBgn0035192|CG9194|649 bp DOWNSTREAM)	GT	1/1	0/0	1/1	0/0	1/1	0/0	1/1	0/0	0/0	0/0	0/0	1/1	0/0	0/0	0/0	0/0	0/0	1/1	0/0	1/1	0/0	0/0	1/1	0/0	1/1	0/0	0/0	0/0	0/0	1/1	0/0	0/0	.	0/0	0/0	1/1	0/0	0/0	1/1	0/0	1/1	1/1	0/0	0/0	0/0	0/0	1/1	0/0	0/0	1/1	1/1	1/1	1/1	.	0/0	0/0	0/0	1/1	0/0	0/0	0/0	0/0	0/0	1/1	0/0	0/0	1/1	0/0	0/0	0/0	0/0	0/0	0/0	0/0	0/0	0/0	1/1	0/0	0/0	0/0	1/1	1/1	0/0	1/1	1/1	0/0	0/0	1/1	0/0	0/0	0/0	0/0	1/1	1/1	0/0	0/0	0/0	0/0	0/0	1/1	1/1	0/0	0/0	1/1	1/1	0/0	0/0	0/0	0/0	1/1	0/0	0/0	1/1	0/0	0/0	1/1	0/0	0/0	0/0	1/1	0/0	0/0	1/1	0/0	0/0	1/1	0/0	1/1	0/0	0/0	1/1	0/0	0/0	0/0	0/0	1/1	1/1	0/0	0/0	0/0	0/0	0/0	0/0	0/0	0/0	0/0	1/1	0/0	0/0	0/0	1/1	1/1	0/0	0/0	0/0	0/0	0/0	0/0	1/1	0/0	1/1	0/0	1/1	0/0	0/0	1/1	0/0	0/0	1/1	1/1	0/0	0/0	1/1	0/0	0/0
3L	1244830	3L_1244830_SNP	G	A	.	.	T5EFF=0.08715;T5VAR=0.0305486917924358;T5VARACC=0.031307321561331;T5PVAL=0.02345;T5MIXED=0.0176871653908286;T6EFF=0.3136;T6VAR=0.110882257380132;T6VARACC=0.0880592810454119;T6PVAL=1.032e-05;T6MIXED=5.31056811706961e-06;AVGEFF=0.2004;AVGVAR=0.0917314594981577;AVGVARACC=0.0770749785573212;AVGPVAL=6.593e-05;AVGMIXED=2.68189614531993e-05;DIFFEFF=-0.2265;DIFFVAR=0.0985689452540289;DIFFVARACC=0.0582842559286964;DIFFPVAL=3.409e-05;DIFFMIXED=2.392821679904e-05;ANNOT=(FBgn0004378|Klp61F|SYNONYMOUS_CODING)	GT	1/1	1/1	1/1	0/0	1/1	1/1	1/1	0/0	0/0	0/0	0/0	0/0	0/0	0/0	0/0	1/1	1/1	1/1	0/0	1/1	0/0	0/0	1/1	0/0	1/1	0/0	0/0	0/0	0/0	1/1	0/0	0/0	.	0/0	0/0	1/1	0/0	1/1	1/1	0/0	1/1	1/1	0/0	0/0	0/0	0/0	0/0	1/1	1/1	1/1	.	1/1	1/1	1/1	0/0	0/0	0/0	1/1	1/1	1/1	0/0	0/0	0/0	1/1	0/0	0/0	0/0	1/1	0/0	.	0/0	0/0	1/1	1/1	0/0	0/0	1/1	1/1	0/0	0/0	1/1	1/1	0/0	1/1	1/1	0/0	0/0	1/1	0/0	0/0	.	1/1	1/1	1/1	0/0	0/0	0/0	0/0	0/0	1/1	1/1	1/1	0/0	1/1	1/1	0/0	0/0	0/0	0/0	1/1	0/0	0/0	.	1/1	1/1	1/1	0/0	1/1	0/0	1/1	0/0	0/0	1/1	0/0	0/0	1/1	0/0	1/1	0/0	0/0	1/1	0/0	0/0	0/0	0/0	1/1	1/1	1/1	0/0	0/0	0/0	0/0	1/1	0/0	0/0	1/1	1/1	0/0	.	0/0	1/1	1/1	0/0	0/0	0/0	0/0	0/0	0/0	1/1	0/0	1/1	0/0	1/1	0/0	0/0	1/1	0/0	0/0	1/1	1/1	0/0	0/0	1/1	.	0/0
3L	1244833	3L_1244833_SNP	G	A	.	.	T5EFF=0.09058;T5VAR=0.0329874888725112;T5VARACC=0.0325991244769642;T5PVAL=0.01811;T5MIXED=0.0108361378308122;T6EFF=0.313;T6VAR=0.111265429963517;T6VARACC=0.0862750692869337;T6PVAL=9.347e-06;T6MIXED=5.83026333800626e-06;AVGEFF=0.2018;AVGVAR=0.0935245234985533;AVGVARACC=0.0780797829899953;AVGPVAL=5.267e-05;AVGMIXED=2.84322644998397e-05;DIFFEFF=-0.2224;DIFFVAR=0.0956992519192198;DIFFVARACC=0.0560072965096758;DIFFPVAL=4.266e-05;DIFFMIXED=3.28853933928184e-05;ANNOT=(FBgn0004378|Klp61F|SYNONYMOUS_CODING)	GT	1/1	1/1	1/1	0/0	1/1	1/1	1/1	0/0	0/0	0/0	0/0	0/0	0/0	0/0	0/0	1/1	1/1	1/1	0/0	1/1	0/0	0/0	1/1	0/0	1/1	0/0	0/0	0/0	0/0	1/1	0/0	0/0	.	0/0	0/0	1/1	0/0	1/1	1/1	0/0	1/1	1/1	0/0	0/0	0/0	0/0	0/0	1/1	1/1	1/1	1/1	1/1	1/1	1/1	0/0	0/0	0/0	1/1	1/1	1/1	0/0	0/0	0/0	1/1	0/0	0/0	0/0	1/1	0/0	.	0/0	0/0	1/1	1/1	0/0	0/0	1/1	1/1	0/0	0/0	1/1	1/1	0/0	1/1	1/1	0/0	0/0	1/1	0/0	0/0	.	1/1	1/1	1/1	0/0	0/0	0/0	0/0	0/0	1/1	1/1	1/1	0/0	1/1	1/1	0/0	0/0	0/0	0/0	1/1	0/0	0/0	.	1/1	1/1	1/1	0/0	1/1	0/0	1/1	0/0	0/0	1/1	0/0	0/0	1/1	0/0	1/1	0/0	0/0	1/1	0/0	0/0	0/0	0/0	1/1	1/1	1/1	0/0	0/0	0/0	0/0	1/1	0/0	0/0	1/1	1/1	0/0	.	0/0	1/1	1/1	0/0	0/0	0/0	0/0	0/0	0/0	1/1	0/0	1/1	0/0	1/1	0/0	0/0	1/1	0/0	0/0	1/1	1/1	0/0	0/0	1/1	.	0/0
3L	1267191	3L_1267191_SNP	G	A	.	.	T5EFF=0.1732;T5VAR=0.0440479807129212;T5VARACC=0.0100310238137502;T5PVAL=0.005581;T5MIXED=0.00292504422152298;T6EFF=0.5236;T6VAR=0.114419304731572;T6VARACC=0.0262297404844098;T6PVAL=5.32e-06;T6MIXED=2.82800268264726e-06;AVGEFF=0.3484;AVGVAR=0.102601041306411;AVGVARACC=0.0323145986789015;AVGPVAL=1.737e-05;AVGMIXED=6.93978439655549e-06;DIFFEFF=-0.3503;DIFFVAR=0.0863069233471777;DIFFVARACC=0.0153598511383799;DIFFPVAL=8.746e-05;DIFFMIXED=6.68663978957838e-05;ANNOT=(FBgn0035199|CG9134|INTRON)	GT	0/0	0/0	1/1	0/0	0/0	0/0	0/0	0/0	0/0	0/0	0/0	0/0	0/0	0/0	0/0	0/0	0/0	0/0	0/0	0/0	1/1	0/0	.	0/0	0/0	0/0	0/0	0/0	0/0	0/0	0/0	0/0	0/0	0/0	0/0	0/0	0/0	0/0	1/1	0/0	0/0	0/0	0/0	0/0	0/0	1/1	0/0	0/0	0/0	1/1	0/0	0/0	1/1	0/0	0/0	0/0	0/0	0/0	0/0	0/0	0/0	1/1	0/0	0/0	0/0	0/0	0/0	0/0	0/0	0/0	0/0	0/0	0/0	0/0	0/0	0/0	0/0	0/0	0/0	0/0	0/0	0/0	0/0	0/0	0/0	0/0	0/0	0/0	0/0	0/0	0/0	0/0	1/1	1/1	0/0	1/1	0/0	0/0	0/0	0/0	1/1	0/0	0/0	0/0	0/0	0/0	0/0	0/0	1/1	0/0	0/0	0/0	0/0	0/0	0/0	0/0	0/0	0/0	0/0	1/1	0/0	0/0	0/0	0/0	0/0	0/0	1/1	0/0	0/0	1/1	0/0	.	0/0	1/1	0/0	0/0	0/0	0/0	0/0	1/1	0/0	0/0	0/0	0/0	0/0	0/0	0/0	0/0	0/0	0/0	0/0	0/0	0/0	0/0	0/0	0/0	0/0	0/0	0/0	0/0	0/0	0/0	0/0	0/0	0/0	0/0	0/0	0/0	0/0	0/0	0/0	0/0	0/0	0/0	0/0
3L	1271857	3L_1271857_SNP	G	T	.	.	T5EFF=0.1648;T5VAR=0.0459317294155775;T5VARACC=0.0110693614525415;T5PVAL=0.005416;T5MIXED=0.00442545563469575;T6EFF=0.5098;T6VAR=0.120622667082113;T6VARACC=0.0287507543601781;T6PVAL=4.256e-06;T6MIXED=1.86799123235662e-06;AVGEFF=0.3373;AVGVAR=0.108275311189545;AVGVARACC=0.0343316161868457;AVGPVAL=1.413e-05;AVGMIXED=6.13036861513805e-06;DIFFEFF=-0.345;DIFFVAR=0.0923336855568779;DIFFVARACC=0.0213785657911331;DIFFPVAL=6.549e-05;DIFFMIXED=3.72859378735292e-05;ANNOT=(FBgn0035199|CG9134|INTRON)	GT	0/0	0/0	1/1	0/0	0/0	.	0/0	0/0	0/0	0/0	0/0	0/0	0/0	0/0	0/0	0/0	0/0	0/0	0/0	0/0	0/0	0/0	1/1	0/0	0/0	0/0	0/0	0/0	0/0	0/0	0/0	0/0	.	0/0	0/0	0/0	0/0	0/0	1/1	0/0	0/0	0/0	0/0	0/0	0/0	1/1	0/0	.	.	1/1	0/0	0/0	1/1	0/0	0/0	0/0	0/0	0/0	0/0	0/0	0/0	1/1	0/0	0/0	0/0	0/0	0/0	0/0	0/0	0/0	0/0	0/0	0/0	0/0	.	.	0/0	0/0	0/0	0/0	0/0	0/0	0/0	0/0	0/0	0/0	0/0	0/0	0/0	0/0	0/0	0/0	1/1	1/1	0/0	1/1	0/0	0/0	0/0	0/0	1/1	0/0	0/0	0/0	0/0	0/0	.	0/0	1/1	0/0	0/0	0/0	0/0	0/0	0/0	0/0	0/0	0/0	0/0	1/1	0/0	0/0	0/0	0/0	0/0	0/0	0/0	0/0	0/0	1/1	0/0	1/1	0/0	.	0/0	0/0	0/0	0/0	0/0	1/1	0/0	0/0	0/0	0/0	0/0	0/0	0/0	0/0	0/0	0/0	0/0	1/1	0/0	0/0	0/0	0/0	0/0	0/0	1/1	0/0	0/0	0/0	0/0	0/0	0/0	0/0	0/0	0/0	1/1	0/0	0/0	0/0	0/0	0/0	0/0
3L	1271873	3L_1271873_SNP	T	C	.	.	T5EFF=0.1707;T5VAR=0.0883886629708345;T5VARACC=0.0307803437942025;T5PVAL=7.12e-05;T5MIXED=0.000406404195284088;T6EFF=0.3529;T6VAR=0.107740668727779;T6VARACC=0.0467845871717914;T6PVAL=1.04e-05;T6MIXED=6.03381861594285e-06;AVGEFF=0.2618;AVGVAR=0.119898741073333;AVGVARACC=0.0598972693990037;AVGPVAL=3.063e-06;AVGMIXED=2.16693871859794e-06;DIFFEFF=-0.1822;DIFFVAR=0.0484910171438595;DIFFVARACC=0.0172185101428581;DIFFPVAL=0.003601;DIFFMIXED=0.00296840524186762;ANNOT=(FBgn0035199|CG9134|INTRON)	GT	0/0	0/0	1/1	0/0	1/1	.	0/0	0/0	0/0	0/0	1/1	0/0	0/0	0/0	0/0	0/0	0/0	1/1	0/0	0/0	0/0	0/0	1/1	0/0	0/0	0/0	0/0	0/0	0/0	0/0	0/0	0/0	.	0/0	0/0	1/1	1/1	0/0	0/0	0/0	1/1	0/0	0/0	0/0	0/0	1/1	0/0	0/0	0/0	1/1	0/0	0/0	1/1	0/0	1/1	0/0	0/0	0/0	0/0	0/0	0/0	1/1	0/0	1/1	0/0	0/0	0/0	0/0	0/0	0/0	0/0	0/0	0/0	0/0	0/0	0/0	0/0	0/0	0/0	0/0	0/0	1/1	0/0	0/0	0/0	0/0	1/1	1/1	0/0	1/1	0/0	0/0	1/1	1/1	0/0	1/1	0/0	0/0	0/0	0/0	1/1	0/0	0/0	1/1	0/0	0/0	0/0	0/0	1/1	0/0	1/1	0/0	0/0	0/0	0/0	0/0	0/0	1/1	0/0	1/1	0/0	0/0	0/0	1/1	0/0	0/0	0/0	0/0	0/0	1/1	1/1	1/1	1/1	1/1	0/0	0/0	0/0	0/0	0/0	1/1	1/1	1/1	0/0	0/0	0/0	0/0	1/1	0/0	0/0	0/0	0/0	1/1	0/0	0/0	0/0	0/0	0/0	0/0	1/1	0/0	0/0	1/1	0/0	0/0	0/0	0/0	0/0	0/0	1/1	0/0	1/1	0/0	0/0	0/0	0/0
3L	1381936	3L_1381936_SNP	G	C	.	.	T5EFF=0.1637;T5VAR=0.10916019911047;T5VARACC=0.0577799812276557;T5PVAL=1.081e-05;T5MIXED=4.41729195755377e-06;T6EFF=0.2309;T6VAR=0.0628400231685839;T6VARACC=0.0329029814407265;T6PVAL=0.0009767;T6MIXED=0.000984927793172988;AVGEFF=0.1973;AVGVAR=0.0921170858800789;AVGVARACC=0.0491581546254848;AVGPVAL=5.737e-05;AVGMIXED=4.84453228184148e-05;DIFFEFF=-0.06726;DIFFVAR=0.00908842156457508;DIFFVARACC=0.000480610918226432;DIFFPVAL=0.2162;DIFFMIXED=0.215431602832191;ANNOT=(FBgn0003138|Ptp61F|INTRON),(FBgn0003295|ru|INTRON)	GT	0/0	1/1	0/0	1/1	1/1	0/0	1/1	1/1	0/0	1/1	1/1	1/1	1/1	1/1	0/0	1/1	0/0	1/1	0/0	1/1	0/0	0/0	1/1	1/1	1/1	1/1	1/1	1/1	1/1	0/0	1/1	1/1	.	1/1	0/0	0/0	0/0	0/0	1/1	0/0	0/0	1/1	1/1	0/0	1/1	1/1	1/1	1/1	1/1	0/0	1/1	0/0	1/1	.	1/1	1/1	1/1	1/1	1/1	1/1	1/1	0/0	1/1	0/0	1/1	0/0	1/1	0/0	0/0	1/1	1/1	0/0	0/0	.	0/0	0/0	1/1	0/0	0/0	1/1	1/1	1/1	1/1	1/1	0/0	0/0	0/0	0/0	0/0	0/0	0/0	0/0	0/0	0/0	1/1	0/0	1/1	0/0	0/0	1/1	0/0	1/1	1/1	0/0	1/1	1/1	1/1	0/0	1/1	0/0	.	1/1	0/0	1/1	1/1	1/1	0/0	1/1	1/1	0/0	1/1	1/1	0/0	0/0	1/1	1/1	0/0	1/1	0/0	0/0	0/0	0/0	1/1	0/0	0/0	0/0	0/0	1/1	1/1	0/0	1/1	1/1	1/1	1/1	1/1	1/1	1/1	1/1	0/0	0/0	0/0	0/0	0/0	0/0	1/1	0/0	1/1	0/0	0/0	0/0	0/0	.	0/0	0/0	1/1	1/1	1/1	1/1	0/0	0/0	0/0	0/0	0/0	1/1	1/1
3L	1543958	3L_1543958_SNP	C	T	.	.	T5EFF=0.09782;T5VAR=0.012664838048247;T5VARACC=0.00017392871702937;T5PVAL=0.1404;T5MIXED=0.132072626795739;T6EFF=0.5474;T6VAR=0.111572815168801;T6VARACC=0.0637133480879541;T6PVAL=7.081e-06;T6MIXED=5.37886448596099e-06;AVGEFF=0.3226;AVGVAR=0.0787654038945506;AVGVARACC=0.0236869817604677;AVGPVAL=0.000184;AVGMIXED=0.000153090188904461;DIFFEFF=-0.4496;DIFFVAR=0.126561261790668;DIFFVARACC=0.0659284492259826;DIFFPVAL=1.56e-06;DIFFMIXED=1.07662299222376e-06;ANNOT=(FBgn0035229|CG7852|SYNONYMOUS_CODING),(FBgn0052313|CG32313|870 bp DOWNSTREAM)	GT	0/0	0/0	1/1	0/0	.	0/0	1/1	0/0	0/0	1/1	0/0	0/0	0/0	0/0	0/0	0/0	0/0	0/0	1/1	0/0	0/0	0/0	0/0	0/0	0/0	0/0	0/0	0/0	0/0	0/0	0/0	0/0	0/0	0/0	0/0	0/0	0/0	0/0	0/0	0/0	0/0	1/1	0/0	0/0	0/0	0/0	0/0	0/0	0/0	1/1	0/0	0/0	0/0	0/0	0/0	0/0	0/0	0/0	1/1	0/0	0/0	0/0	0/0	0/0	0/0	0/0	0/0	1/1	0/0	0/0	0/0	0/0	0/0	0/0	0/0	0/0	0/0	0/0	0/0	0/0	0/0	1/1	0/0	0/0	0/0	0/0	0/0	0/0	0/0	0/0	0/0	0/0	0/0	0/0	0/0	0/0	0/0	0/0	.	0/0	0/0	0/0	0/0	0/0	0/0	0/0	0/0	0/0	0/0	0/0	0/0	1/1	0/0	0/0	0/0	1/1	0/0	0/0	0/0	1/1	0/0	0/0	0/0	0/0	0/0	0/0	0/0	0/0	0/0	0/0	0/0	0/0	1/1	0/0	0/0	0/0	0/0	0/0	0/0	0/0	0/0	0/0	0/0	0/0	0/0	0/0	0/0	0/0	0/0	0/0	0/0	0/0	0/0	0/0	0/0	0/0	0/0	0/0	0/0	0/0	0/0	0/0	0/0	0/0	0/0	0/0	0/0	0/0	0/0	1/1	0/0	0/0	1/1	0/0	0/0
3L	1544057	3L_1544057_SNP	C	T	.	.	T5EFF=0.07127;T5VAR=0.00705336441001349;T5VARACC=0.000176740078665916;T5PVAL=0.2706;T5MIXED=0.243149033028829;T6EFF=0.4941;T6VAR=0.0960994971112889;T6VARACC=0.0488871064178802;T6PVAL=3.145e-05;T6MIXED=2.50587009115913e-05;AVGEFF=0.2827;AVGVAR=0.063732149318272;AVGVARACC=0.0162867197051048;AVGPVAL=0.0007775;AVGMIXED=0.000627430916686312;DIFFEFF=-0.4228;DIFFVAR=0.118747657548491;DIFFVARACC=0.0563479987808863;DIFFPVAL=3.221e-06;DIFFMIXED=2.44883193085133e-06;ANNOT=(FBgn0035229|CG7852|SYNONYMOUS_CODING),(FBgn0052313|CG32313|771 bp DOWNSTREAM)	GT	0/0	0/0	1/1	0/0	.	0/0	1/1	0/0	0/0	1/1	0/0	0/0	0/0	0/0	0/0	0/0	0/0	0/0	1/1	0/0	0/0	0/0	0/0	0/0	0/0	0/0	0/0	0/0	0/0	0/0	0/0	0/0	0/0	0/0	0/0	0/0	0/0	0/0	0/0	0/0	0/0	1/1	0/0	0/0	0/0	0/0	0/0	0/0	0/0	1/1	0/0	0/0	0/0	0/0	0/0	0/0	0/0	0/0	1/1	0/0	0/0	0/0	0/0	0/0	0/0	0/0	0/0	1/1	0/0	0/0	0/0	0/0	0/0	0/0	0/0	0/0	0/0	0/0	0/0	0/0	0/0	1/1	0/0	0/0	0/0	0/0	0/0	0/0	0/0	0/0	0/0	0/0	0/0	0/0	0/0	0/0	0/0	0/0	1/1	0/0	0/0	0/0	0/0	0/0	0/0	0/0	0/0	0/0	0/0	0/0	0/0	1/1	0/0	0/0	0/0	1/1	0/0	0/0	0/0	1/1	0/0	0/0	0/0	0/0	0/0	0/0	0/0	0/0	0/0	0/0	0/0	0/0	1/1	0/0	0/0	0/0	0/0	0/0	0/0	0/0	0/0	0/0	0/0	0/0	0/0	0/0	0/0	0/0	0/0	0/0	0/0	0/0	0/0	0/0	0/0	0/0	0/0	0/0	0/0	0/0	0/0	0/0	0/0	0/0	0/0	0/0	0/0	0/0	0/0	1/1	0/0	0/0	1/1	0/0	0/0
3L	17094322	3L_17094322_SNP	C	T	.	.	T5EFF=0.07323;T5VAR=0.00869837887848706;T5VARACC=0.00743018494883904;T5PVAL=0.2349;T5MIXED=0.412304160887449;T6EFF=-0.3128;T6VAR=0.0465152251685772;T6VARACC=0.023421543394909;T6PVAL=0.005545;T6MIXED=0.00414457317009983;AVGEFF=-0.1198;AVGVAR=0.0137929054506111;AVGVARACC=0.0112226263403429;AVGPVAL=0.1342;AVGMIXED=0.0867597321808282;DIFFEFF=0.386;DIFFVAR=0.116562988638626;DIFFVARACC=0.062467592798415;DIFFPVAL=7.679e-06;DIFFMIXED=6.80381635948133e-06;ANNOT=(FBgn0260943|Rbp6|INTRON)	GT	0/0	0/0	0/0	0/0	0/0	0/0	0/0	0/0	1/1	0/0	0/0	0/0	0/0	0/0	0/0	0/0	0/0	0/0	0/0	0/0	0/0	0/0	0/0	0/0	0/0	.	0/0	1/1	0/0	0/0	0/0	0/0	0/0	0/0	0/0	1/1	0/0	0/0	0/0	1/1	0/0	0/0	1/1	0/0	0/0	0/0	0/0	0/0	0/0	0/0	0/0	0/0	0/0	0/0	0/0	0/0	0/0	.	0/0	0/0	0/0	0/0	0/0	1/1	0/0	0/0	0/0	.	1/1	0/0	0/0	0/0	0/0	0/0	0/0	0/0	0/0	0/0	0/0	.	0/0	0/0	0/0	0/0	0/0	0/0	0/0	0/0	1/1	0/0	0/0	1/1	0/0	0/0	0/0	0/0	0/0	0/0	0/0	0/0	0/0	0/0	1/1	0/0	0/0	0/0	0/0	0/0	0/0	0/0	0/0	0/0	0/0	0/0	.	0/0	0/0	0/0	1/1	0/0	1/1	0/0	1/1	0/0	1/1	0/0	0/0	0/0	0/0	0/0	0/0	0/0	0/0	0/0	1/1	.	.	0/0	1/1	0/0	0/0	0/0	.	0/0	0/0	0/0	0/0	0/0	0/0	0/0	1/1	0/0	0/0	0/0	0/0	0/0	0/0	0/0	0/0	0/0	.	0/0	0/0	0/0	0/0	0/0	1/1	0/0	0/0	.	0/0	0/0	0/0	.	0/0
3L	17193468	3L_17193468_SNP	G	A	.	.	T5EFF=-0.3227;T5VAR=0.122167989845247;T5VARACC=0.0943116107537681;T5PVAL=3.42e-06;T5MIXED=3.07591931686841e-06;T6EFF=-0.2762;T6VAR=0.0251145784930292;T6VARACC=0.0103516866413312;T6PVAL=0.0402;T6MIXED=0.0441033986186026;AVGEFF=-0.2994;AVGVAR=0.0598665753832526;AVGVARACC=0.0271594946831136;AVGPVAL=0.001391;AVGMIXED=0.00235561221207991;DIFFEFF=-0.04657;DIFFVAR=0.00121317662375588;DIFFVARACC=0.00763786206886646;DIFFPVAL=0.654;DIFFMIXED=0.632278381742173;ANNOT=(FBgn0260943|Rbp6|INTRON)	GT	0/0	0/0	0/0	1/1	0/0	0/0	0/0	0/0	0/0	1/1	.	0/0	0/0	0/0	0/0	0/0	0/0	0/0	0/0	0/0	0/0	0/0	0/0	1/1	0/0	0/0	0/0	0/0	0/0	0/0	0/0	0/0	0/0	0/0	0/0	0/0	0/0	0/0	1/1	0/0	0/0	0/0	0/0	0/0	0/0	0/0	0/0	0/0	0/0	0/0	.	0/0	0/0	0/0	1/1	0/0	0/0	0/0	1/1	0/0	0/0	0/0	0/0	0/0	0/0	.	0/0	0/0	0/0	0/0	0/0	0/0	0/0	0/0	.	0/0	.	0/0	0/0	0/0	0/0	0/0	0/0	0/0	0/0	0/0	0/0	0/0	0/0	0/0	0/0	0/0	0/0	0/0	0/0	0/0	0/0	0/0	0/0	0/0	.	0/0	0/0	0/0	0/0	0/0	1/1	0/0	0/0	0/0	0/0	0/0	0/0	1/1	.	0/0	0/0	0/0	0/0	0/0	0/0	0/0	0/0	0/0	1/1	0/0	0/0	0/0	0/0	0/0	0/0	0/0	0/0	0/0	0/0	0/0	0/0	1/1	0/0	0/0	0/0	0/0	0/0	0/0	0/0	0/0	0/0	0/0	0/0	1/1	0/0	0/0	0/0	0/0	0/0	0/0	0/0	0/0	0/0	0/0	0/0	0/0	0/0	0/0	0/0	0/0	0/0	0/0	0/0	0/0	0/0	0/0	1/1	0/0	1/1
3L	1904606	3L_1904606_SNP	A	T	.	.	T5EFF=0.1525;T5VAR=0.079306847236445;T5VARACC=0.0191101642659755;T5PVAL=0.000199;T5MIXED=0.000246635817928404;T6EFF=0.3556;T6VAR=0.123418754362529;T6VARACC=0.0313431989129474;T6PVAL=2.636e-06;T6MIXED=3.66151779468061e-06;AVGEFF=0.2541;AVGVAR=0.12816100387663;AVGVARACC=0.033026825933511;AVGPVAL=1.642e-06;AVGMIXED=2.76068605423428e-06;DIFFEFF=-0.2031;DIFFVAR=0.0663920433111333;DIFFVARACC=0.00513830647850727;DIFFPVAL=0.0006932;DIFFMIXED=0.000644775322914202;ANNOT=(FBgn0035290|CG1887|INTRON)	GT	0/0	0/0	0/0	0/0	0/0	0/0	0/0	0/0	0/0	0/0	0/0	1/1	0/0	0/0	1/1	0/0	0/0	1/1	1/1	1/1	0/0	1/1	0/0	.	1/1	0/0	1/1	1/1	0/0	0/0	0/0	0/0	1/1	1/1	1/1	0/0	0/0	0/0	0/0	0/0	0/0	0/0	.	0/0	0/0	1/1	0/0	0/0	0/0	1/1	0/0	1/1	0/0	1/1	0/0	0/0	0/0	0/0	0/0	1/1	0/0	1/1	0/0	1/1	0/0	0/0	0/0	.	0/0	0/0	0/0	0/0	.	1/1	0/0	0/0	0/0	1/1	0/0	1/1	0/0	0/0	0/0	0/0	0/0	0/0	0/0	0/0	0/0	1/1	0/0	1/1	0/0	0/0	0/0	1/1	1/1	0/0	0/0	1/1	0/0	0/0	1/1	1/1	0/0	0/0	0/0	0/0	0/0	0/0	0/0	0/0	1/1	0/0	1/1	0/0	0/0	1/1	0/0	0/0	0/0	0/0	1/1	0/0	0/0	0/0	0/0	1/1	1/1	0/0	0/0	1/1	0/0	0/0	0/0	1/1	1/1	0/0	1/1	1/1	0/0	0/0	1/1	1/1	1/1	0/0	0/0	0/0	0/0	1/1	0/0	0/0	0/0	0/0	0/0	0/0	0/0	0/0	1/1	1/1	0/0	0/0	0/0	0/0	0/0	0/0	0/0	0/0	1/1	0/0	1/1	0/0	0/0	.	0/0
3L	20271995	3L_20271995_SNP	C	T	.	.	T5EFF=-0.2205;T5VAR=0.100413815647813;T5VARACC=0.0374237705610169;T5PVAL=2.698e-05;T5MIXED=4.47223282804677e-06;T6EFF=-0.3304;T6VAR=0.0642002170818384;T6VARACC=0.0301828297560319;T6PVAL=0.0008878;T6MIXED=0.00086694419531872;AVGEFF=-0.2754;AVGVAR=0.0903862706258143;AVGVARACC=0.0459441686177131;AVGPVAL=7.138e-05;AVGMIXED=5.09450663107646e-05;DIFFEFF=0.1098;DIFFVAR=0.0119307681070413;DIFFVARACC=3.60050418078406e-05;DIFFPVAL=0.1575;DIFFMIXED=0.160742281155359;ANNOT=(FBgn0052227|gogo|INTRON)	GT	0/0	0/0	0/0	1/1	.	0/0	0/0	0/0	0/0	1/1	1/1	1/1	0/0	1/1	0/0	1/1	0/0	0/0	0/0	0/0	.	0/0	1/1	0/0	0/0	0/0	0/0	0/0	0/0	0/0	1/1	0/0	1/1	0/0	1/1	0/0	0/0	0/0	0/0	0/0	0/0	0/0	1/1	0/0	0/0	0/0	0/0	0/0	0/0	0/0	0/0	0/0	1/1	0/0	0/0	.	0/0	0/0	0/0	0/0	0/0	0/0	1/1	0/0	0/0	0/0	0/0	0/0	0/0	0/0	0/0	0/0	0/0	0/0	0/0	0/0	0/0	0/0	0/0	0/0	0/0	0/0	0/0	0/0	0/0	0/0	0/0	0/0	0/0	0/0	0/0	0/0	0/0	0/0	0/0	0/0	1/1	0/0	0/0	0/0	0/0	0/0	0/0	0/0	0/0	0/0	0/0	1/1	0/0	0/0	1/1	0/0	0/0	1/1	0/0	0/0	1/1	0/0	0/0	0/0	0/0	.	0/0	0/0	0/0	1/1	0/0	.	0/0	0/0	0/0	0/0	0/0	0/0	0/0	0/0	0/0	1/1	0/0	0/0	0/0	0/0	0/0	0/0	0/0	0/0	0/0	1/1	0/0	0/0	0/0	0/0	0/0	0/0	0/0	0/0	0/0	0/0	0/0	0/0	0/0	0/0	1/1	1/1	0/0	1/1	0/0	0/0	0/0	0/0	0/0	1/1	0/0	.	0/0
3L	21331129	3L_21331129_SNP	T	A	.	.	T5EFF=0.1754;T5VAR=0.0745837897225794;T5VARACC=0.060513853222163;T5PVAL=0.00042;T5MIXED=0.000132747555237745;T6EFF=0.3843;T6VAR=0.105047504876395;T6VARACC=0.0690067450842393;T6PVAL=2.436e-05;T6MIXED=2.31535197928902e-05;AVGEFF=0.2799;AVGVAR=0.111038637296392;AVGVARACC=0.0879009193259054;AVGPVAL=1.383e-05;AVGMIXED=8.68255269712619e-06;DIFFEFF=-0.2089;DIFFVAR=0.0535057481882246;DIFFVARACC=0.0326601033537383;DIFFPVAL=0.002969;DIFFMIXED=0.003069819148909;ANNOT=(FBgn0037073|CG7338|UTR_3_PRIME),(FBgn0037074|CG7324|121 bp DOWNSTREAM)	GT	0/0	0/0	1/1	0/0	.	0/0	0/0	0/0	0/0	0/0	0/0	0/0	0/0	0/0	0/0	0/0	0/0	0/0	0/0	1/1	1/1	0/0	0/0	0/0	0/0	0/0	0/0	0/0	0/0	1/1	0/0	0/0	0/0	0/0	0/0	0/0	.	.	1/1	0/0	0/0	0/0	0/0	0/0	1/1	0/0	0/0	0/0	0/0	0/0	0/0	1/1	0/0	0/0	0/0	0/0	0/0	0/0	1/1	1/1	1/1	0/0	0/0	1/1	0/0	0/0	0/0	0/0	0/0	0/0	0/0	0/0	0/0	0/0	0/0	1/1	0/0	0/0	0/0	.	1/1	0/0	1/1	0/0	0/0	0/0	0/0	0/0	0/0	0/0	0/0	0/0	0/0	0/0	0/0	0/0	.	1/1	0/0	0/0	1/1	0/0	.	1/1	0/0	0/0	0/0	1/1	0/0	1/1	0/0	0/0	1/1	0/0	0/0	1/1	0/0	0/0	0/0	.	.	0/0	1/1	0/0	0/0	0/0	0/0	0/0	0/0	0/0	0/0	1/1	0/0	1/1	1/1	1/1	.	0/0	0/0	1/1	0/0	0/0	0/0	0/0	0/0	1/1	0/0	0/0	0/0	0/0	0/0	0/0	0/0	0/0	0/0	0/0	0/0	0/0	0/0	0/0	0/0	.	0/0	0/0	0/0	0/0	0/0	0/0	1/1	.	0/0	0/0	1/1	.	0/0
3L	21372701	3L_21372701_SNP	G	C	.	.	T5EFF=0.1008;T5VAR=0.0408786808537561;T5VARACC=0.0175944347611736;T5PVAL=0.01192;T5MIXED=0.0116452248648254;T6EFF=0.3292;T6VAR=0.127521946737774;T6VARACC=0.0726547767259301;T6PVAL=5.462e-06;T6MIXED=6.98598590913331e-06;AVGEFF=0.215;AVGVAR=0.109592836063592;AVGVARACC=0.0633311663421914;AVGPVAL=2.746e-05;AVGMIXED=4.52110212408236e-05;DIFFEFF=-0.2284;DIFFVAR=0.102338971992767;DIFFVARACC=0.0625649512330069;DIFFPVAL=5.246e-05;DIFFMIXED=4.33682074734225e-05;ANNOT=(FBgn0052440|CG32440|18372 bp DOWNSTREAM),(FBgn0261258|rgn|2900 bp DOWNSTREAM)	GT	0/0	0/0	1/1	0/0	.	0/0	1/1	0/0	0/0	1/1	0/0	0/0	1/1	1/1	1/1	0/0	0/0	0/0	0/0	0/0	1/1	1/1	1/1	1/1	1/1	0/0	0/0	0/0	0/0	1/1	1/1	0/0	1/1	1/1	1/1	1/1	.	0/0	1/1	1/1	0/0	1/1	0/0	0/0	1/1	0/0	0/0	1/1	1/1	1/1	0/0	1/1	0/0	0/0	0/0	.	1/1	0/0	1/1	1/1	1/1	1/1	0/0	1/1	0/0	1/1	0/0	.	.	0/0	1/1	1/1	0/0	0/0	0/0	1/1	0/0	0/0	1/1	.	1/1	0/0	1/1	1/1	0/0	1/1	1/1	0/0	1/1	1/1	0/0	0/0	1/1	0/0	0/0	0/0	1/1	.	.	0/0	1/1	0/0	1/1	0/0	0/0	0/0	0/0	1/1	1/1	.	0/0	0/0	1/1	0/0	1/1	1/1	0/0	0/0	0/0	.	0/0	.	1/1	1/1	0/0	0/0	1/1	.	0/0	1/1	1/1	1/1	0/0	1/1	1/1	1/1	.	0/0	0/0	1/1	0/0	0/0	.	0/0	0/0	1/1	0/0	1/1	0/0	0/0	1/1	0/0	0/0	1/1	0/0	.	0/0	.	1/1	0/0	1/1	.	0/0	0/0	.	1/1	0/0	0/0	.	.	0/0	0/0	1/1	.	0/0
3L	3649407	3L_3649407_SNP	T	G	.	.	T5EFF=-0.3539;T5VAR=0.11740327001451;T5VARACC=0.124042783961007;T5PVAL=4.205e-06;T5MIXED=0.000277218584152994;T6EFF=-0.2132;T6VAR=0.0118359103676721;T6VARACC=0.0224816144108155;T6PVAL=0.1554;T6MIXED=0.19174144129639;AVGEFF=-0.2835;AVGVAR=0.0428430974044145;AVGVARACC=0.0699197466385647;AVGPVAL=0.006443;AVGMIXED=0.0263046108117095;DIFFEFF=-0.1407;DIFFVAR=0.00858123715329875;DIFFVARACC=0.00549918650797548;DIFFPVAL=0.2268;DIFFMIXED=0.221556819531855;ANNOT=(FBgn0263239|dar1|INTRON)	GT	0/0	0/0	0/0	0/0	0/0	0/0	0/0	0/0	0/0	1/1	0/0	0/0	0/0	0/0	0/0	0/0	0/0	0/0	0/0	0/0	0/0	0/0	0/0	0/0	0/0	0/0	1/1	0/0	0/0	0/0	0/0	0/0	0/0	0/0	0/0	0/0	0/0	0/0	0/0	0/0	1/1	0/0	0/0	0/0	0/0	0/0	0/0	0/0	0/0	0/0	0/0	0/0	0/0	0/0	0/0	0/0	0/0	0/0	0/0	1/1	0/0	0/0	0/0	0/0	1/1	0/0	0/0	0/0	0/0	0/0	0/0	1/1	0/0	0/0	0/0	0/0	0/0	0/0	0/0	0/0	0/0	0/0	1/1	0/0	0/0	0/0	0/0	0/0	0/0	0/0	0/0	0/0	0/0	0/0	0/0	0/0	0/0	.	0/0	0/0	0/0	0/0	0/0	0/0	0/0	0/0	0/0	0/0	0/0	0/0	0/0	0/0	0/0	.	0/0	0/0	0/0	0/0	0/0	0/0	0/0	0/0	0/0	0/0	0/0	0/0	0/0	0/0	0/0	0/0	0/0	0/0	0/0	0/0	0/0	0/0	0/0	0/0	0/0	0/0	0/0	0/0	0/0	0/0	0/0	0/0	0/0	1/1	0/0	0/0	0/0	0/0	0/0	0/0	0/0	0/0	0/0	0/0	0/0	0/0	0/0	0/0	0/0	0/0	1/1	1/1	0/0	0/0	0/0	0/0	0/0	0/0	0/0	.	0/0
3L	3649448	3L_3649448_SNP	C	T	.	.	T5EFF=-0.4154;T5VAR=0.145970138231642;T5VARACC=0.139296492859824;T5PVAL=2.32e-07;T5MIXED=2.01722727245498e-06;T6EFF=-0.316;T6VAR=0.0237430609182183;T6VARACC=0.0317823503228175;T6PVAL=0.04357;T6MIXED=0.0483288876166839;AVGEFF=-0.3657;AVGVAR=0.0648244128338986;AVGVARACC=0.0888284946352822;AVGPVAL=0.0007503;AVGMIXED=0.00157174606388312;DIFFEFF=-0.09942;DIFFVAR=0.00391770007932067;DIFFVARACC=0.00277124787904154;DIFFPVAL=0.4147;DIFFMIXED=0.417213216423102;ANNOT=(FBgn0263239|dar1|INTRON)	GT	0/0	0/0	0/0	0/0	0/0	0/0	0/0	0/0	0/0	1/1	0/0	0/0	0/0	0/0	0/0	0/0	0/0	0/0	0/0	0/0	0/0	0/0	0/0	0/0	0/0	0/0	1/1	0/0	0/0	0/0	0/0	0/0	0/0	0/0	0/0	0/0	0/0	0/0	0/0	0/0	1/1	0/0	0/0	0/0	0/0	0/0	0/0	0/0	0/0	0/0	0/0	0/0	0/0	0/0	0/0	0/0	0/0	0/0	0/0	.	0/0	0/0	0/0	0/0	1/1	0/0	0/0	0/0	0/0	0/0	0/0	1/1	0/0	0/0	0/0	0/0	0/0	0/0	0/0	0/0	0/0	0/0	1/1	0/0	0/0	0/0	0/0	0/0	0/0	0/0	0/0	0/0	0/0	0/0	0/0	0/0	0/0	0/0	0/0	0/0	0/0	0/0	0/0	0/0	0/0	0/0	0/0	0/0	0/0	0/0	0/0	0/0	0/0	0/0	0/0	0/0	0/0	0/0	0/0	0/0	0/0	0/0	0/0	0/0	0/0	0/0	0/0	0/0	0/0	0/0	0/0	0/0	0/0	0/0	0/0	0/0	0/0	0/0	0/0	0/0	0/0	0/0	0/0	0/0	0/0	0/0	0/0	1/1	0/0	0/0	0/0	0/0	0/0	0/0	0/0	0/0	0/0	0/0	0/0	0/0	0/0	0/0	0/0	0/0	1/1	1/1	0/0	0/0	0/0	0/0	0/0	0/0	0/0	.	.
3L	3698913	3L_3698913_SNP	T	C	.	.	T5EFF=-0.2522;T5VAR=0.108359983558259;T5VARACC=0.0396507516065566;T5PVAL=9.771e-06;T5MIXED=0.000141704312069975;T6EFF=-0.3154;T6VAR=0.0481119406574556;T6VARACC=0.00384817958739669;T6PVAL=0.003738;T6MIXED=0.00428767232850375;AVGEFF=-0.2838;AVGVAR=0.0790030846103434;AVGVARACC=0.0188606234017653;AVGPVAL=0.0001797;AVGMIXED=0.000513687237446158;DIFFEFF=0.06329;DIFFVAR=0.00325100036460232;DIFFVARACC=0.00363050378706209;DIFFPVAL=0.4562;DIFFMIXED=0.438916122189036;ANNOT=(FBgn0263496|CR43485|4686 bp UPSTREAM),(FBgn0262535|CG43089|15114 bp DOWNSTREAM)	GT	0/0	0/0	1/1	1/1	0/0	0/0	0/0	1/1	0/0	1/1	0/0	0/0	0/0	0/0	0/0	1/1	1/1	0/0	0/0	0/0	0/0	0/0	0/0	0/0	0/0	0/0	0/0	0/0	0/0	0/0	0/0	0/0	0/0	0/0	0/0	1/1	1/1	0/0	0/0	0/0	1/1	0/0	0/0	0/0	0/0	0/0	0/0	0/0	0/0	0/0	0/0	0/0	0/0	0/0	0/0	0/0	0/0	0/0	0/0	0/0	1/1	0/0	0/0	0/0	0/0	0/0	0/0	0/0	0/0	0/0	0/0	0/0	0/0	0/0	0/0	0/0	0/0	1/1	0/0	0/0	0/0	0/0	0/0	1/1	.	0/0	1/1	0/0	0/0	0/0	0/0	0/0	0/0	0/0	0/0	0/0	0/0	0/0	0/0	0/0	1/1	1/1	0/0	0/0	0/0	1/1	0/0	0/0	0/0	0/0	0/0	0/0	0/0	1/1	0/0	0/0	0/0	0/0	0/0	0/0	0/0	0/0	0/0	0/0	0/0	0/0	0/0	0/0	0/0	0/0	0/0	0/0	0/0	0/0	0/0	0/0	0/0	0/0	0/0	0/0	0/0	0/0	0/0	0/0	0/0	0/0	0/0	1/1	0/0	0/0	0/0	0/0	0/0	0/0	0/0	0/0	0/0	0/0	0/0	1/1	0/0	0/0	0/0	0/0	0/0	0/0	0/0	0/0	0/0	0/0	0/0	1/1	0/0	.	0/0
3L	496532	3L_496532_SNP	T	C	.	.	T5EFF=0.1424;T5VAR=0.0467772524349483;T5VARACC=0.0265702586283625;T5PVAL=0.004615;T5MIXED=0.00672283180650432;T6EFF=0.4219;T6VAR=0.117303992801698;T6VARACC=0.0814134897401045;T6PVAL=4.838e-06;T6MIXED=6.21601228552744e-06;AVGEFF=0.2821;AVGVAR=0.105882918325054;AVGVARACC=0.0762634279659838;AVGPVAL=1.493e-05;AVGMIXED=2.94034803083376e-05;DIFFEFF=-0.2795;DIFFVAR=0.0873521118282471;DIFFVARACC=0.0397596997123779;DIFFPVAL=9.119e-05;DIFFMIXED=8.19376751719881e-05;ANNOT=(FBgn0001316|klar|INTRON)	GT	1/1	1/1	0/0	1/1	1/1	1/1	1/1	1/1	1/1	1/1	.	0/0	1/1	1/1	1/1	1/1	1/1	1/1	1/1	1/1	1/1	1/1	1/1	0/0	0/0	1/1	1/1	0/0	1/1	0/0	1/1	1/1	.	0/0	1/1	1/1	1/1	1/1	1/1	1/1	0/0	.	1/1	1/1	1/1	1/1	1/1	1/1	1/1	0/0	1/1	0/0	1/1	1/1	1/1	1/1	1/1	1/1	1/1	1/1	1/1	0/0	1/1	1/1	1/1	1/1	1/1	0/0	1/1	0/0	0/0	1/1	1/1	0/0	1/1	1/1	1/1	0/0	1/1	1/1	1/1	1/1	1/1	1/1	1/1	1/1	1/1	1/1	1/1	1/1	1/1	1/1	1/1	1/1	1/1	0/0	0/0	1/1	1/1	1/1	1/1	1/1	0/0	0/0	1/1	1/1	1/1	1/1	1/1	1/1	1/1	.	0/0	1/1	1/1	1/1	1/1	1/1	1/1	1/1	1/1	1/1	0/0	1/1	1/1	1/1	0/0	.	1/1	1/1	0/0	0/0	1/1	1/1	1/1	0/0	0/0	1/1	1/1	1/1	1/1	1/1	1/1	0/0	1/1	1/1	1/1	1/1	1/1	1/1	1/1	1/1	1/1	1/1	1/1	1/1	1/1	0/0	1/1	1/1	1/1	1/1	1/1	1/1	1/1	1/1	1/1	1/1	1/1	1/1	1/1	1/1	1/1	1/1	1/1
3L	5524263	3L_5524263_SNP	G	A	.	.	T5EFF=0.03714;T5VAR=0.00543419217874446;T5VARACC=0.00205035767079803;T5PVAL=0.3423;T5MIXED=0.200799621350838;T6EFF=0.2875;T6VAR=0.0902836192385581;T6VARACC=0.0528698867861518;T6PVAL=7.579e-05;T6MIXED=9.11015673082881e-05;AVGEFF=0.1623;AVGVAR=0.0588151300304173;AVGVARACC=0.0267638556144239;AVGPVAL=0.001538;AVGMIXED=0.0014741044356691;DIFFEFF=-0.2504;DIFFVAR=0.114617615192359;DIFFVARACC=0.059822716368596;DIFFPVAL=7.168e-06;DIFFMIXED=8.16306883455106e-06;ANNOT=(FBgn0035610|Lkr|1822 bp UPSTREAM),(FBgn0035611|CG13285|2805 bp UPSTREAM)	GT	1/1	1/1	0/0	.	0/0	1/1	0/0	1/1	.	1/1	0/0	1/1	1/1	0/0	0/0	1/1	1/1	0/0	0/0	1/1	0/0	1/1	1/1	1/1	1/1	1/1	1/1	1/1	0/0	1/1	0/0	0/0	.	0/0	1/1	1/1	0/0	1/1	1/1	1/1	0/0	1/1	0/0	1/1	1/1	0/0	1/1	1/1	1/1	0/0	1/1	0/0	1/1	1/1	1/1	0/0	1/1	1/1	0/0	0/0	1/1	0/0	0/0	1/1	1/1	0/0	1/1	0/0	0/0	1/1	1/1	1/1	1/1	0/0	0/0	1/1	1/1	0/0	1/1	1/1	1/1	0/0	1/1	0/0	1/1	1/1	1/1	1/1	1/1	1/1	0/0	1/1	1/1	0/0	0/0	0/0	1/1	1/1	1/1	0/0	1/1	0/0	1/1	0/0	1/1	1/1	0/0	1/1	1/1	0/0	0/0	1/1	0/0	0/0	1/1	1/1	0/0	1/1	1/1	0/0	1/1	0/0	1/1	1/1	1/1	1/1	1/1	1/1	1/1	1/1	0/0	0/0	1/1	1/1	1/1	1/1	1/1	1/1	1/1	0/0	0/0	1/1	0/0	0/0	0/0	1/1	1/1	1/1	1/1	1/1	1/1	0/0	1/1	1/1	1/1	1/1	1/1	0/0	1/1	.	.	1/1	1/1	1/1	1/1	1/1	1/1	0/0	1/1	1/1	1/1	0/0	.	.	0/0
3L	5540120	3L_5540120_SNP	C	G	.	.	T5EFF=-0.3348;T5VAR=0.110114545447681;T5VARACC=0.0686772711922062;T5PVAL=7.254e-06;T5MIXED=3.09388802105353e-05;T6EFF=-0.3898;T6VAR=0.0424212230439093;T6VARACC=0.0089244694740922;T6PVAL=0.006247;T6MIXED=0.00638788125252201;AVGEFF=-0.3623;AVGVAR=0.0741841778200908;AVGVARACC=0.0403783671178204;AVGPVAL=0.0002659;AVGMIXED=0.000404964770423503;DIFFEFF=0.05504;DIFFVAR=0.00142759694010599;DIFFVARACC=0.00656028814964305;DIFFPVAL=0.6196;DIFFMIXED=0.608761904819126;ANNOT=(FBgn0035612|CG10625|INTRON)	GT	0/0	0/0	0/0	1/1	0/0	0/0	0/0	0/0	0/0	0/0	0/0	0/0	0/0	0/0	0/0	0/0	0/0	0/0	0/0	0/0	0/0	0/0	0/0	0/0	0/0	0/0	0/0	0/0	0/0	0/0	0/0	1/1	0/0	0/0	0/0	0/0	1/1	0/0	0/0	0/0	0/0	0/0	0/0	0/0	0/0	0/0	0/0	0/0	0/0	0/0	0/0	0/0	0/0	0/0	0/0	1/1	0/0	0/0	0/0	0/0	0/0	0/0	0/0	0/0	0/0	0/0	0/0	0/0	0/0	0/0	0/0	0/0	0/0	0/0	0/0	0/0	0/0	0/0	0/0	0/0	0/0	1/1	0/0	0/0	0/0	0/0	0/0	0/0	0/0	0/0	0/0	0/0	0/0	0/0	0/0	0/0	0/0	0/0	0/0	0/0	0/0	0/0	0/0	0/0	0/0	0/0	0/0	0/0	0/0	0/0	0/0	0/0	0/0	1/1	0/0	0/0	0/0	0/0	0/0	0/0	0/0	1/1	0/0	0/0	0/0	0/0	0/0	0/0	0/0	0/0	0/0	0/0	0/0	0/0	0/0	0/0	0/0	0/0	0/0	0/0	0/0	0/0	0/0	0/0	0/0	0/0	0/0	1/1	0/0	1/1	0/0	0/0	0/0	0/0	0/0	0/0	0/0	0/0	0/0	0/0	0/0	0/0	0/0	0/0	0/0	1/1	0/0	1/1	0/0	0/0	0/0	0/0	0/0	0/0	0/0
3L	5552263	3L_5552263_SNP	G	A	.	.	T5EFF=-0.3075;T5VAR=0.115933990622782;T5VARACC=0.0710155166863913;T5PVAL=4.872e-06;T5MIXED=3.1187797697057e-06;T6EFF=-0.4216;T6VAR=0.0629785556210321;T6VARACC=0.031900978827235;T6PVAL=0.0008984;T6MIXED=0.000780985736758176;AVGEFF=-0.3646;AVGVAR=0.0945640228140801;AVGVARACC=0.0636897723322726;AVGPVAL=4.07e-05;AVGMIXED=2.69197215132582e-05;DIFFEFF=0.1141;DIFFVAR=0.00786704392558781;DIFFVARACC=0.00447025984862293;DIFFPVAL=0.2473;DIFFMIXED=0.244580928419921;ANNOT=(FBgn0010894|sinu|307 bp DOWNSTREAM)	GT	0/0	0/0	0/0	0/0	0/0	0/0	0/0	1/1	.	0/0	0/0	0/0	0/0	0/0	0/0	0/0	0/0	0/0	0/0	0/0	0/0	1/1	0/0	0/0	0/0	1/1	0/0	0/0	0/0	0/0	0/0	1/1	.	0/0	0/0	0/0	0/0	0/0	0/0	0/0	0/0	.	1/1	0/0	0/0	0/0	0/0	0/0	0/0	0/0	0/0	0/0	0/0	0/0	0/0	0/0	1/1	0/0	0/0	0/0	0/0	0/0	0/0	0/0	0/0	0/0	1/1	0/0	0/0	0/0	0/0	0/0	0/0	0/0	0/0	0/0	0/0	0/0	0/0	0/0	0/0	0/0	0/0	0/0	0/0	0/0	0/0	0/0	0/0	0/0	0/0	0/0	0/0	0/0	0/0	0/0	0/0	0/0	0/0	0/0	0/0	0/0	0/0	0/0	0/0	0/0	0/0	0/0	0/0	1/1	0/0	0/0	0/0	1/1	0/0	0/0	0/0	0/0	0/0	0/0	0/0	0/0	0/0	1/1	0/0	0/0	0/0	0/0	0/0	0/0	0/0	0/0	0/0	0/0	0/0	0/0	0/0	0/0	0/0	0/0	0/0	1/1	0/0	0/0	0/0	0/0	0/0	1/1	0/0	1/1	0/0	0/0	0/0	0/0	0/0	0/0	0/0	0/0	0/0	0/0	0/0	0/0	0/0	0/0	0/0	0/0	0/0	1/1	0/0	0/0	0/0	0/0	0/0	0/0	0/0
3L	5556250	3L_5556250_SNP	G	A	.	.	T5EFF=-0.2641;T5VAR=0.112918050873875;T5VARACC=0.0502094747824312;T5PVAL=7.012e-06;T5MIXED=6.1059237487853e-06;T6EFF=-0.2943;T6VAR=0.0399944815007326;T6VARACC=0.00708575540014728;T6PVAL=0.008728;T6MIXED=0.00818499287063186;AVGEFF=-0.2792;AVGVAR=0.0726856097049749;AVGVARACC=0.0256487021712781;AVGPVAL=0.0003626;AVGMIXED=0.000333783625952399;DIFFEFF=0.03017;DIFFVAR=0.000713023373048207;DIFFVARACC=0.00141538114993356;DIFFPVAL=0.7288;DIFFMIXED=0.729222820568932;ANNOT=(FBgn0029118|Sucb|SYNONYMOUS_CODING),(FBgn0029121|Sras|624 bp UPSTREAM)	GT	0/0	0/0	0/0	1/1	.	0/0	0/0	0/0	0/0	0/0	1/1	0/0	0/0	0/0	0/0	0/0	0/0	0/0	0/0	0/0	0/0	0/0	0/0	0/0	0/0	1/1	0/0	0/0	0/0	0/0	0/0	1/1	0/0	1/1	0/0	0/0	1/1	0/0	0/0	0/0	0/0	0/0	0/0	0/0	0/0	0/0	0/0	0/0	0/0	0/0	0/0	0/0	0/0	0/0	0/0	0/0	0/0	1/1	0/0	0/0	0/0	0/0	0/0	0/0	0/0	0/0	0/0	0/0	0/0	0/0	0/0	0/0	0/0	0/0	0/0	0/0	0/0	0/0	0/0	0/0	0/0	.	0/0	0/0	0/0	1/1	0/0	0/0	0/0	0/0	1/1	0/0	0/0	0/0	1/1	0/0	0/0	0/0	0/0	0/0	0/0	1/1	0/0	0/0	0/0	0/0	0/0	0/0	0/0	1/1	0/0	0/0	0/0	1/1	.	1/1	0/0	0/0	0/0	0/0	0/0	0/0	0/0	0/0	0/0	1/1	0/0	.	0/0	0/0	0/0	0/0	0/0	0/0	0/0	0/0	0/0	0/0	0/0	0/0	0/0	0/0	0/0	0/0	0/0	0/0	0/0	1/1	0/0	1/1	0/0	0/0	1/1	0/0	0/0	0/0	0/0	0/0	0/0	0/0	0/0	0/0	0/0	0/0	0/0	0/0	0/0	1/1	0/0	0/0	0/0	0/0	0/0	0/0	0/0
3L	8166801	3L_8166801_SNP	C	G	.	.	T5EFF=0.05357;T5VAR=0.00593665294474579;T5VARACC=0.00393791623898965;T5PVAL=0.3151;T5MIXED=0.287762830162447;T6EFF=0.3847;T6VAR=0.0872695580576867;T6VARACC=0.0525815029051118;T6PVAL=8.346e-05;T6MIXED=6.16780543430775e-05;AVGEFF=0.2191;AVGVAR=0.0574047657971681;AVGVARACC=0.0477639938134937;AVGPVAL=0.001548;AVGMIXED=0.00126335838308411;DIFFEFF=-0.3311;DIFFVAR=0.108291205585364;DIFFVARACC=0.0411518949351529;DIFFPVAL=1.045e-05;DIFFMIXED=7.90032118101474e-06;ANNOT=(FBgn0040290|RecQ4|SYNONYMOUS_CODING)	GT	1/1	0/0	0/0	0/0	.	0/0	1/1	0/0	0/0	0/0	1/1	0/0	0/0	0/0	0/0	0/0	0/0	0/0	1/1	0/0	1/1	0/0	0/0	0/0	.	0/0	0/0	0/0	0/0	1/1	0/0	0/0	0/0	0/0	0/0	0/0	0/0	0/0	1/1	0/0	0/0	1/1	0/0	0/0	0/0	0/0	0/0	0/0	0/0	0/0	0/0	0/0	0/0	0/0	0/0	0/0	1/1	0/0	0/0	0/0	0/0	1/1	0/0	1/1	0/0	0/0	0/0	1/1	0/0	0/0	0/0	0/0	0/0	0/0	0/0	0/0	0/0	0/0	0/0	1/1	0/0	0/0	0/0	0/0	0/0	0/0	1/1	0/0	0/0	0/0	0/0	0/0	0/0	0/0	1/1	1/1	0/0	0/0	0/0	0/0	1/1	1/1	0/0	0/0	0/0	0/0	0/0	0/0	1/1	1/1	0/0	0/0	0/0	0/0	0/0	1/1	0/0	0/0	0/0	0/0	0/0	0/0	0/0	0/0	0/0	0/0	0/0	0/0	0/0	0/0	1/1	1/1	0/0	0/0	0/0	0/0	0/0	0/0	0/0	0/0	0/0	1/1	0/0	0/0	0/0	0/0	0/0	0/0	0/0	0/0	0/0	0/0	0/0	0/0	0/0	0/0	0/0	0/0	0/0	0/0	0/0	0/0	0/0	0/0	0/0	0/0	0/0	0/0	0/0	0/0	0/0	0/0	1/1	.	0/0
3L	8878124	3L_8878124_SNP	C	T	.	.	T5EFF=0.1956;T5VAR=0.116453249020542;T5VARACC=0.0519778025629378;T5PVAL=6.817e-06;T5MIXED=7.92196540455383e-05;T6EFF=0.3099;T6VAR=0.0832967499245073;T6VARACC=0.0556007238523514;T6PVAL=0.0001626;T6MIXED=0.000223876460316411;AVGEFF=0.2527;AVGVAR=0.11232851733822;AVGVARACC=0.061774244828837;AVGPVAL=1.015e-05;AVGMIXED=4.0343554086676e-05;DIFFEFF=-0.1142;DIFFVAR=0.0189742852365237;DIFFVARACC=0.00165039841648679;DIFFPVAL=0.07676;DIFFMIXED=0.0723796398891793;ANNOT=(FBgn0263930|dally|INTRON)	GT	0/0	1/1	1/1	0/0	0/0	0/0	0/0	0/0	1/1	0/0	0/0	0/0	1/1	0/0	0/0	0/0	.	0/0	0/0	0/0	0/0	0/0	0/0	0/0	0/0	0/0	0/0	0/0	0/0	0/0	0/0	0/0	0/0	0/0	0/0	0/0	0/0	0/0	0/0	0/0	1/1	1/1	.	0/0	0/0	0/0	0/0	0/0	0/0	0/0	0/0	0/0	0/0	.	0/0	0/0	0/0	0/0	0/0	0/0	0/0	1/1	0/0	1/1	0/0	0/0	1/1	1/1	0/0	0/0	0/0	1/1	0/0	0/0	0/0	0/0	0/0	0/0	0/0	0/0	0/0	1/1	0/0	1/1	0/0	0/0	0/0	0/0	1/1	1/1	0/0	0/0	0/0	0/0	1/1	1/1	0/0	0/0	0/0	.	0/0	0/0	0/0	1/1	0/0	0/0	0/0	0/0	0/0	0/0	0/0	.	0/0	0/0	1/1	0/0	0/0	1/1	0/0	1/1	.	0/0	1/1	1/1	0/0	0/0	0/0	0/0	0/0	.	1/1	1/1	0/0	1/1	1/1	.	1/1	0/0	1/1	1/1	0/0	0/0	1/1	1/1	0/0	1/1	0/0	0/0	0/0	0/0	1/1	0/0	0/0	0/0	0/0	0/0	0/0	0/0	0/0	0/0	1/1	1/1	0/0	0/0	0/0	0/0	0/0	0/0	1/1	1/1	1/1	0/0	1/1	.	0/0
3L	8888688	3L_8888688_SNP	C	A	.	.	T5EFF=0.1511;T5VAR=0.0917178101199024;T5VARACC=0.0205518381757459;T5PVAL=6.946e-05;T5MIXED=0.000375627624075872;T6EFF=0.3138;T6VAR=0.111481009405461;T6VARACC=0.0173462850349869;T6PVAL=1.036e-05;T6MIXED=1.37053821054702e-05;AVGEFF=0.2324;AVGVAR=0.123811844212836;AVGVARACC=0.0381796278156361;AVGPVAL=3.116e-06;AVGMIXED=1.02279934085992e-05;DIFFEFF=-0.1627;DIFFVAR=0.0510296601123019;DIFFVARACC=0.00828373727248238;DIFFPVAL=0.003332;DIFFMIXED=0.00331336444809823;ANNOT=(FBgn0035934|TrpA1|INTRON)	GT	0/0	0/0	0/0	0/0	0/0	1/1	0/0	0/0	1/1	1/1	0/0	0/0	0/0	1/1	0/0	0/0	0/0	1/1	1/1	0/0	1/1	0/0	0/0	0/0	1/1	0/0	1/1	0/0	1/1	1/1	0/0	0/0	0/0	0/0	0/0	0/0	0/0	0/0	.	0/0	1/1	1/1	1/1	0/0	0/0	0/0	0/0	0/0	0/0	1/1	.	0/0	0/0	1/1	1/1	1/1	1/1	0/0	1/1	0/0	1/1	1/1	0/0	1/1	0/0	0/0	0/0	1/1	0/0	0/0	0/0	1/1	1/1	1/1	1/1	0/0	0/0	1/1	0/0	0/0	0/0	1/1	.	1/1	1/1	1/1	0/0	0/0	1/1	1/1	1/1	0/0	1/1	0/0	0/0	1/1	0/0	1/1	0/0	.	0/0	0/0	0/0	0/0	0/0	0/0	0/0	0/0	1/1	0/0	0/0	1/1	0/0	0/0	1/1	0/0	0/0	1/1	0/0	0/0	.	0/0	1/1	0/0	0/0	0/0	1/1	0/0	0/0	1/1	1/1	1/1	0/0	1/1	1/1	1/1	0/0	0/0	1/1	1/1	1/1	1/1	0/0	1/1	1/1	1/1	0/0	0/0	0/0	1/1	0/0	0/0	1/1	1/1	1/1	.	0/0	0/0	1/1	.	0/0	1/1	0/0	0/0	0/0	0/0	1/1	0/0	1/1	1/1	1/1	0/0	1/1	.	0/0
3L	9020823	3L_9020823_SNP	C	A	.	.	T5EFF=0.2128;T5VAR=0.0417841143613415;T5VARACC=0.00764626694450277;T5PVAL=0.007499;T5MIXED=0.00174337735959365;T6EFF=0.6469;T6VAR=0.10933772159705;T6VARACC=0.0260839587914372;T6PVAL=1.063e-05;T6MIXED=7.62663685716664e-06;AVGEFF=0.4299;AVGVAR=0.0981206750507281;AVGVARACC=0.03011370733213;AVGPVAL=3.194e-05;AVGMIXED=1.22779069005135e-05;DIFFEFF=-0.4341;DIFFVAR=0.0822746593520165;DIFFVARACC=0.0148611502798489;DIFFPVAL=0.0001493;DIFFMIXED=0.000121035982035839;ANNOT=(FBgn0035956|Doc2|8477 bp UPSTREAM),(FBgn0028789|Doc1|13652 bp DOWNSTREAM)	GT	0/0	0/0	0/0	0/0	0/0	0/0	0/0	0/0	0/0	0/0	0/0	0/0	0/0	0/0	0/0	0/0	0/0	.	1/1	0/0	.	0/0	0/0	0/0	0/0	0/0	0/0	0/0	1/1	0/0	0/0	0/0	0/0	0/0	0/0	0/0	0/0	0/0	0/0	0/0	0/0	0/0	0/0	0/0	1/1	0/0	0/0	0/0	0/0	1/1	0/0	0/0	0/0	0/0	0/0	0/0	0/0	0/0	0/0	1/1	0/0	1/1	0/0	0/0	0/0	0/0	0/0	0/0	0/0	0/0	0/0	0/0	0/0	0/0	0/0	0/0	0/0	0/0	0/0	0/0	0/0	0/0	0/0	0/0	0/0	0/0	0/0	0/0	0/0	0/0	0/0	0/0	1/1	0/0	0/0	0/0	0/0	0/0	0/0	0/0	0/0	0/0	0/0	0/0	0/0	0/0	0/0	0/0	0/0	0/0	0/0	0/0	0/0	0/0	0/0	0/0	0/0	0/0	0/0	0/0	0/0	0/0	0/0	0/0	0/0	0/0	1/1	0/0	.	0/0	1/1	1/1	0/0	0/0	0/0	0/0	0/0	0/0	0/0	0/0	0/0	0/0	0/0	0/0	0/0	0/0	0/0	0/0	0/0	0/0	0/0	0/0	0/0	0/0	0/0	0/0	0/0	0/0	0/0	.	0/0	0/0	0/0	0/0	0/0	0/0	0/0	0/0	0/0	0/0	0/0	0/0	0/0	.	0/0
3L	9295771	3L_9295771_MNP	CTG	ACA	.	.	T5EFF=0.1752;T5VAR=0.122121315837977;T5VARACC=0.070662199173824;T5PVAL=4.212e-06;T5MIXED=1.16670685914185e-05;T6EFF=0.2607;T6VAR=0.0722762176294684;T6VARACC=0.0305976945730425;T6PVAL=0.0004801;T6MIXED=0.000478094324708457;AVGEFF=0.218;AVGVAR=0.10382676926546;AVGVARACC=0.0333860160669672;AVGPVAL=2.436e-05;AVGMIXED=3.77364102221903e-05;DIFFEFF=-0.08558;DIFFVAR=0.0132280081611876;DIFFVARACC=0.000670247692163173;DIFFPVAL=0.1413;DIFFMIXED=0.13533444411571;ANNOT=(FBgn0264000|GluRIB|14149 bp DOWNSTREAM),(FBgn0035975|PGRP-LA|31661 bp DOWNSTREAM)	GT	0/0	0/0	1/1	.	.	0/0	0/0	0/0	0/0	0/0	0/0	1/1	1/1	0/0	0/0	1/1	0/0	0/0	1/1	0/0	0/0	0/0	0/0	0/0	1/1	0/0	0/0	0/0	1/1	0/0	1/1	0/0	1/1	1/1	1/1	1/1	1/1	1/1	0/0	0/0	0/0	0/0	0/0	0/0	1/1	0/0	0/0	0/0	0/0	1/1	0/0	1/1	0/0	1/1	0/0	1/1	0/0	0/0	0/0	1/1	1/1	1/1	0/0	0/0	0/0	0/0	0/0	0/0	0/0	0/0	1/1	1/1	1/1	0/0	.	1/1	.	0/0	.	0/0	1/1	1/1	0/0	1/1	0/0	0/0	0/0	0/0	1/1	0/0	.	0/0	1/1	1/1	0/0	0/0	1/1	1/1	0/0	0/0	0/0	0/0	1/1	0/0	0/0	0/0	0/0	0/0	0/0	.	0/0	0/0	.	0/0	1/1	1/1	1/1	1/1	0/0	1/1	.	0/0	1/1	0/0	0/0	0/0	1/1	0/0	0/0	0/0	1/1	1/1	0/0	0/0	1/1	1/1	0/0	0/0	0/0	1/1	0/0	0/0	0/0	1/1	0/0	1/1	0/0	0/0	0/0	0/0	0/0	0/0	0/0	1/1	0/0	1/1	0/0	1/1	1/1	0/0	0/0	1/1	0/0	0/0	0/0	0/0	1/1	0/0	1/1	1/1	1/1	1/1	1/1	.	0/0
3L	970261	3L_970261_SNP	G	A	.	.	T5EFF=0.108;T5VAR=0.028115399677295;T5VARACC=0.010177172236432;T5PVAL=0.02933;T5MIXED=0.00911235806152243;T6EFF=0.3773;T6VAR=0.0979077026502811;T6VARACC=0.0530677063554597;T6PVAL=3.442e-05;T6MIXED=7.9367204084609e-06;AVGEFF=0.2426;AVGVAR=0.081658372811852;AVGVARACC=0.0638669186047441;AVGPVAL=0.0001658;AVGMIXED=1.2115767931691e-05;DIFFEFF=-0.2693;DIFFVAR=0.084990920662741;DIFFVARACC=0.0552514673804249;DIFFPVAL=0.0001202;DIFFMIXED=8.05982452267744e-05;ANNOT=(FBgn0264574|Glut1|INTRON)	GT	0/0	0/0	0/0	0/0	.	0/0	1/1	0/0	.	0/0	0/0	0/0	0/0	1/1	0/0	0/0	1/1	1/1	0/0	0/0	0/0	0/0	0/0	0/0	0/0	.	0/0	0/0	0/0	0/0	1/1	0/0	0/0	0/0	0/0	0/0	0/0	0/0	0/0	0/0	0/0	0/0	0/0	0/0	0/0	0/0	0/0	0/0	0/0	0/0	1/1	0/0	0/0	0/0	1/1	0/0	0/0	0/0	0/0	1/1	0/0	0/0	0/0	0/0	0/0	0/0	0/0	0/0	0/0	.	0/0	0/0	1/1	0/0	0/0	0/0	0/0	0/0	0/0	0/0	0/0	1/1	0/0	1/1	1/1	0/0	0/0	0/0	0/0	0/0	0/0	0/0	0/0	0/0	0/0	1/1	0/0	0/0	0/0	0/0	1/1	0/0	0/0	1/1	1/1	0/0	0/0	0/0	0/0	0/0	.	0/0	0/0	0/0	.	1/1	0/0	0/0	1/1	0/0	0/0	0/0	0/0	0/0	0/0	0/0	0/0	0/0	0/0	0/0	1/1	0/0	0/0	0/0	0/0	0/0	0/0	0/0	1/1	0/0	1/1	1/1	0/0	1/1	0/0	0/0	0/0	0/0	0/0	1/1	0/0	0/0	0/0	0/0	0/0	0/0	0/0	0/0	1/1	0/0	0/0	0/0	1/1	0/0	0/0	0/0	0/0	0/0	1/1	1/1	0/0	0/0	1/1	0/0	1/1
3L	9800362	3L_9800362_SNP	G	A	.	.	T5EFF=-0.2865;T5VAR=0.12183709380415;T5VARACC=0.104660770545266;T5PVAL=3.301e-06;T5MIXED=2.04638298265548e-06;T6EFF=-0.3344;T6VAR=0.0473930720710444;T6VARACC=0.0240004765680412;T6PVAL=0.004465;T6MIXED=0.0038058209097368;AVGEFF=-0.3104;AVGVAR=0.0825645136199859;AVGVARACC=0.05673069197122;AVGPVAL=0.0001519;AVGMIXED=0.000113821689490088;DIFFEFF=0.04787;DIFFVAR=0.00164037745917953;DIFFVARACC=1.44689955183797e-07;DIFFPVAL=0.6011;DIFFMIXED=0.587057317071869;ANNOT=(FBgn0044328|CG32052|INTRON)	GT	0/0	0/0	0/0	1/1	0/0	0/0	0/0	0/0	0/0	0/0	0/0	0/0	0/0	0/0	0/0	0/0	0/0	1/1	0/0	0/0	0/0	0/0	0/0	0/0	0/0	1/1	0/0	1/1	0/0	0/0	.	0/0	0/0	0/0	0/0	0/0	0/0	0/0	0/0	1/1	0/0	0/0	1/1	1/1	0/0	0/0	0/0	0/0	0/0	0/0	0/0	.	0/0	0/0	0/0	0/0	0/0	0/0	0/0	0/0	0/0	0/0	0/0	0/0	0/0	0/0	0/0	0/0	0/0	0/0	0/0	0/0	1/1	0/0	0/0	0/0	0/0	0/0	.	0/0	0/0	1/1	0/0	0/0	0/0	0/0	0/0	0/0	1/1	0/0	0/0	0/0	0/0	0/0	0/0	0/0	0/0	0/0	0/0	0/0	0/0	0/0	0/0	0/0	0/0	0/0	1/1	0/0	0/0	0/0	0/0	0/0	1/1	0/0	0/0	0/0	0/0	.	0/0	0/0	0/0	0/0	0/0	0/0	0/0	0/0	0/0	0/0	0/0	0/0	0/0	0/0	0/0	0/0	0/0	0/0	0/0	0/0	0/0	0/0	0/0	0/0	0/0	0/0	0/0	0/0	0/0	1/1	0/0	0/0	0/0	0/0	0/0	0/0	0/0	.	1/1	0/0	0/0	1/1	.	0/0	0/0	0/0	0/0	1/1	0/0	0/0	0/0	0/0	0/0	1/1	0/0	0/0	0/0
3L	9963496	3L_9963496_SNP	C	G	.	.	T5EFF=-0.208;T5VAR=0.106064321920854;T5VARACC=0.0646755298835846;T5PVAL=1.159e-05;T5MIXED=7.05807607500251e-06;T6EFF=-0.1911;T6VAR=0.0252331343015105;T6VARACC=0.0135441448252839;T6PVAL=0.0363;T6MIXED=0.0289559218245142;AVGEFF=-0.1996;AVGVAR=0.055842013081954;AVGVARACC=0.0444140589423343;AVGPVAL=0.001694;AVGMIXED=0.00106689358319366;DIFFEFF=-0.01689;DIFFVAR=0.000332319531759357;DIFFVARACC=0.000199203717308916;DIFFPVAL=0.8113;DIFFMIXED=0.824816379556202;ANNOT=(FBgn0026404|Nc|NON_SYNONYMOUS_CODING),(FBgn0036063|CG6674|755 bp DOWNSTREAM)	GT	0/0	0/0	0/0	1/1	0/0	1/1	0/0	1/1	0/0	1/1	0/0	0/0	1/1	0/0	0/0	0/0	1/1	0/0	0/0	0/0	0/0	0/0	0/0	0/0	0/0	1/1	0/0	0/0	0/0	0/0	0/0	0/0	1/1	1/1	1/1	0/0	0/0	0/0	0/0	0/0	0/0	0/0	0/0	0/0	0/0	0/0	0/0	0/0	1/1	0/0	0/0	1/1	0/0	0/0	0/0	0/0	0/0	0/0	0/0	0/0	0/0	1/1	0/0	0/0	0/0	0/0	0/0	0/0	0/0	0/0	0/0	0/0	0/0	0/0	1/1	0/0	.	0/0	0/0	0/0	0/0	0/0	0/0	0/0	0/0	1/1	1/1	0/0	0/0	0/0	0/0	0/0	1/1	0/0	0/0	0/0	1/1	0/0	0/0	0/0	0/0	0/0	0/0	0/0	0/0	1/1	1/1	0/0	0/0	1/1	0/0	0/0	1/1	1/1	0/0	0/0	0/0	0/0	0/0	0/0	0/0	0/0	0/0	0/0	1/1	0/0	0/0	1/1	1/1	0/0	0/0	0/0	0/0	0/0	0/0	1/1	0/0	0/0	0/0	0/0	0/0	0/0	0/0	0/0	0/0	0/0	0/0	1/1	0/0	0/0	0/0	0/0	0/0	0/0	0/0	0/0	1/1	0/0	0/0	1/1	0/0	0/0	0/0	0/0	0/0	0/0	0/0	0/0	0/0	0/0	0/0	1/1	0/0	0/0	0/0
3L	9984425	3L_9984425_SNP	G	A	.	.	T5EFF=-0.1754;T5VAR=0.116509874136256;T5VARACC=0.103432884405326;T5PVAL=7.234e-06;T5MIXED=1.28580682406078e-05;T6EFF=-0.2355;T6VAR=0.0603483803982816;T6VARACC=0.0466274996915591;T6PVAL=0.001471;T6MIXED=0.00130659927930168;AVGEFF=-0.2055;AVGVAR=0.0930330350804371;AVGVARACC=0.0818036071758257;AVGPVAL=6.79e-05;AVGMIXED=7.37410442356459e-05;DIFFEFF=0.0601;DIFFVAR=0.00654987716034929;DIFFVARACC=0.00127706344964085;DIFFPVAL=0.3014;DIFFMIXED=0.28991174202521;ANNOT=(FBgn0040823|dpr6|INTRON)	GT	1/1	1/1	1/1	0/0	1/1	1/1	1/1	0/0	0/0	0/0	0/0	1/1	1/1	1/1	1/1	0/0	1/1	0/0	1/1	1/1	1/1	0/0	1/1	1/1	0/0	.	0/0	1/1	1/1	1/1	0/0	1/1	1/1	1/1	1/1	1/1	0/0	1/1	1/1	1/1	0/0	0/0	0/0	1/1	.	1/1	0/0	1/1	0/0	0/0	1/1	1/1	0/0	0/0	1/1	0/0	0/0	1/1	1/1	0/0	1/1	1/1	0/0	1/1	1/1	1/1	0/0	1/1	1/1	0/0	1/1	1/1	0/0	.	0/0	1/1	.	1/1	1/1	1/1	0/0	1/1	0/0	1/1	1/1	0/0	0/0	1/1	0/0	1/1	1/1	1/1	0/0	1/1	1/1	1/1	0/0	0/0	0/0	1/1	1/1	.	1/1	1/1	1/1	0/0	0/0	1/1	1/1	0/0	.	1/1	1/1	0/0	1/1	1/1	1/1	0/0	1/1	1/1	1/1	1/1	1/1	1/1	0/0	1/1	1/1	.	0/0	1/1	1/1	1/1	1/1	1/1	1/1	0/0	.	0/0	1/1	1/1	1/1	1/1	1/1	1/1	0/0	1/1	1/1	0/0	1/1	.	1/1	0/0	0/0	1/1	0/0	1/1	0/0	0/0	1/1	1/1	1/1	0/0	1/1	1/1	1/1	0/0	0/0	0/0	1/1	1/1	1/1	0/0	0/0	.	1/1
3R	10605876	3R_10605876_INS	A	AA	.	.	T5EFF=-0.04519;T5VAR=0.0065815904222469;T5VARACC=0.000762474853710782;T5PVAL=0.3407;T5MIXED=0.377683802112619;T6EFF=-0.335;T6VAR=0.100153245985331;T6VARACC=0.052939070058761;T6PVAL=0.0001393;T6MIXED=9.28569801635227e-05;AVGEFF=-0.1901;AVGVAR=0.0663523205716006;AVGVARACC=0.0571496396882726;AVGPVAL=0.002122;AVGMIXED=0.00218094251095744;DIFFEFF=0.2898;DIFFVAR=0.122916712980759;DIFFVARACC=0.0623688691205307;DIFFPVAL=2.169e-05;DIFFMIXED=8.26932966839312e-06;ANNOT=(FBgn0263929|jvl|INTRON)	GT	1/1	.	0/0	1/1	.	0/0	1/1	1/1	0/0	1/1	1/1	.	1/1	1/1	1/1	.	1/1	1/1	1/1	1/1	1/1	1/1	1/1	1/1	1/1	.	1/1	.	1/1	.	1/1	0/0	1/1	1/1	1/1	1/1	.	0/0	1/1	.	.	1/1	0/0	1/1	.	0/0	1/1	.	0/0	1/1	1/1	1/1	1/1	1/1	1/1	1/1	1/1	.	1/1	1/1	1/1	1/1	0/0	.	.	1/1	0/0	.	1/1	1/1	1/1	.	1/1	1/1	1/1	0/0	1/1	1/1	.	1/1	1/1	.	1/1	1/1	1/1	1/1	0/0	0/0	.	1/1	1/1	0/0	.	.	0/0	1/1	1/1	0/0	.	1/1	1/1	.	1/1	1/1	1/1	1/1	1/1	0/0	0/0	1/1	.	1/1	1/1	1/1	0/0	1/1	1/1	.	0/0	.	1/1	.	1/1	1/1	0/0	0/0	.	.	0/0	.	1/1	1/1	0/0	.	1/1	1/1	1/1	0/0	0/0	0/0	.	1/1	1/1	0/0	0/0	1/1	1/1	0/0	0/0	1/1	1/1	0/0	1/1	0/0	0/0	0/0	1/1	1/1	1/1	1/1	.	1/1	0/0	0/0	0/0	1/1	1/1	0/0	1/1	1/1	1/1	1/1	1/1	1/1	.
3R	11478545	3R_11478545_SNP	T	A	.	.	T5EFF=-0.2992;T5VAR=0.110314669086826;T5VARACC=0.0528947794662709;T5PVAL=8.54e-06;T5MIXED=3.52733821074527e-05;T6EFF=-0.2417;T6VAR=0.0203731691479803;T6VARACC=0.00527434851340697;T6PVAL=0.06178;T6MIXED=0.0743752305250603;AVGEFF=-0.2704;AVGVAR=0.0516015139939379;AVGVARACC=0.0241503386020057;AVGPVAL=0.002729;AVGMIXED=0.00601353831984675;DIFFEFF=-0.05748;DIFFVAR=0.00195718066139227;DIFFVARACC=0.00578133672021153;DIFFPVAL=0.5644;DIFFMIXED=0.549066923662777;ANNOT=(FBgn0263041|CG43336|14642 bp DOWNSTREAM),(FBgn0053207|pxb|13360 bp DOWNSTREAM)	GT	0/0	0/0	1/1	0/0	0/0	0/0	0/0	0/0	0/0	1/1	.	0/0	0/0	0/0	0/0	1/1	0/0	0/0	0/0	0/0	0/0	0/0	0/0	0/0	0/0	0/0	0/0	0/0	0/0	0/0	0/0	0/0	0/0	0/0	0/0	0/0	0/0	0/0	0/0	0/0	0/0	0/0	0/0	0/0	1/1	0/0	0/0	0/0	0/0	0/0	0/0	0/0	1/1	0/0	0/0	0/0	0/0	0/0	0/0	0/0	0/0	0/0	0/0	0/0	1/1	0/0	1/1	0/0	0/0	0/0	0/0	0/0	0/0	0/0	0/0	0/0	0/0	0/0	0/0	0/0	0/0	0/0	0/0	0/0	.	1/1	0/0	0/0	0/0	0/0	0/0	0/0	0/0	0/0	0/0	0/0	0/0	0/0	0/0	0/0	0/0	0/0	0/0	1/1	0/0	0/0	1/1	0/0	.	0/0	0/0	0/0	0/0	1/1	0/0	0/0	0/0	0/0	0/0	0/0	0/0	0/0	0/0	0/0	0/0	0/0	0/0	0/0	0/0	0/0	0/0	0/0	0/0	0/0	0/0	0/0	0/0	0/0	0/0	0/0	0/0	1/1	0/0	0/0	0/0	0/0	0/0	0/0	0/0	0/0	0/0	0/0	0/0	0/0	0/0	0/0	0/0	0/0	0/0	0/0	0/0	0/0	0/0	0/0	0/0	1/1	0/0	0/0	0/0	0/0	0/0	1/1	0/0	0/0	0/0
3R	1193279	3R_1193279_SNP	A	C	.	.	T5EFF=0.161;T5VAR=0.118505386714829;T5VARACC=0.0886318403250363;T5PVAL=8.867e-06;T5MIXED=9.13864828590215e-05;T6EFF=0.1599;T6VAR=0.0320863649615371;T6VARACC=0.00811997142458498;T6PVAL=0.02387;T6MIXED=0.0261196357649699;AVGEFF=0.1605;AVGVAR=0.0674057220335001;AVGVARACC=0.0333951211127935;AVGPVAL=0.0009505;AVGMIXED=0.00196813691161604;DIFFEFF=0.001138;DIFFVAR=2.57522178001626e-06;DIFFVARACC=0.00358430709894808;DIFFPVAL=0.984;DIFFMIXED=0.988057290038891;ANNOT=(FBgn0037315|Cerk|INTRON),(FBgn0264912|CR44103|924 bp UPSTREAM)	GT	0/0	0/0	1/1	1/1	.	0/0	1/1	.	1/1	1/1	1/1	0/0	0/0	0/0	1/1	1/1	1/1	1/1	0/0	1/1	0/0	1/1	0/0	0/0	1/1	1/1	0/0	0/0	0/0	1/1	0/0	0/0	0/0	0/0	0/0	0/0	1/1	0/0	1/1	1/1	.	0/0	.	1/1	0/0	1/1	1/1	1/1	1/1	0/0	1/1	1/1	0/0	1/1	1/1	.	1/1	1/1	1/1	0/0	0/0	1/1	1/1	.	1/1	1/1	1/1	0/0	1/1	1/1	1/1	1/1	1/1	.	0/0	0/0	0/0	0/0	0/0	0/0	0/0	0/0	1/1	1/1	.	1/1	0/0	0/0	0/0	.	0/0	0/0	0/0	0/0	1/1	0/0	0/0	0/0	1/1	1/1	0/0	0/0	0/0	0/0	1/1	1/1	1/1	1/1	0/0	0/0	0/0	1/1	1/1	.	1/1	1/1	1/1	1/1	0/0	0/0	0/0	0/0	0/0	1/1	0/0	1/1	.	0/0	1/1	1/1	1/1	0/0	1/1	0/0	0/0	0/0	.	1/1	0/0	1/1	1/1	1/1	.	0/0	1/1	0/0	.	.	0/0	1/1	1/1	1/1	1/1	1/1	0/0	1/1	1/1	0/0	0/0	1/1	1/1	1/1	0/0	0/0	1/1	1/1	1/1	1/1	1/1	.	0/0	1/1	0/0	0/0	1/1
3R	1206780	3R_1206780_DEL	AA	A	.	.	T5EFF=-0.2131;T5VAR=0.145925230062841;T5VARACC=0.116209023880396;T5PVAL=8.622e-07;T5MIXED=1.29579103423222e-06;T6EFF=-0.2729;T6VAR=0.0685673457321771;T6VARACC=0.0108579066192284;T6PVAL=0.0009598;T6MIXED=0.000918796801211004;AVGEFF=-0.243;AVGVAR=0.111038501044037;AVGVARACC=0.0394215222665666;AVGPVAL=2.132e-05;AVGMIXED=3.02764153613387e-05;DIFFEFF=0.05977;DIFFVAR=0.00535329679598063;DIFFVARACC=0.00674674142520837;DIFFPVAL=0.364;DIFFMIXED=0.346667993944589;ANNOT=(FBgn0001311|kkv|INTRON)	GT	0/0	.	0/0	1/1	0/0	0/0	0/0	.	1/1	0/0	1/1	0/0	0/0	0/0	0/0	0/0	1/1	1/1	0/0	.	0/0	1/1	0/0	0/0	1/1	1/1	0/0	0/0	0/0	1/1	0/0	0/0	0/0	0/0	0/0	0/0	1/1	0/0	1/1	0/0	1/1	0/0	.	1/1	0/0	0/0	1/1	0/0	0/0	0/0	.	0/0	0/0	0/0	1/1	0/0	.	0/0	0/0	0/0	.	0/0	.	0/0	1/1	1/1	0/0	0/0	.	1/1	0/0	.	1/1	0/0	1/1	0/0	.	0/0	0/0	0/0	0/0	0/0	0/0	0/0	0/0	1/1	0/0	0/0	0/0	0/0	0/0	0/0	0/0	0/0	1/1	0/0	0/0	0/0	1/1	0/0	0/0	0/0	0/0	0/0	1/1	1/1	1/1	0/0	0/0	0/0	0/0	0/0	0/0	0/0	1/1	1/1	0/0	1/1	0/0	0/0	0/0	.	0/0	1/1	0/0	0/0	0/0	0/0	0/0	0/0	0/0	0/0	1/1	0/0	0/0	0/0	0/0	0/0	0/0	.	0/0	0/0	.	0/0	0/0	0/0	.	1/1	0/0	1/1	.	0/0	1/1	.	0/0	.	0/0	.	0/0	0/0	1/1	0/0	0/0	0/0	1/1	1/1	1/1	1/1	0/0	0/0	0/0	1/1	0/0	0/0	1/1
3R	1211046	3R_1211046_SNP	T	G	.	.	T5EFF=-0.1807;T5VAR=0.125892066266024;T5VARACC=0.0891276695771961;T5PVAL=7.287e-06;T5MIXED=1.75272003304014e-05;T6EFF=-0.1767;T6VAR=0.0362059732380477;T6VARACC=0.00926525405289702;T6PVAL=0.01887;T6MIXED=0.0201859511761565;AVGEFF=-0.1787;AVGVAR=0.0737607138379941;AVGVARACC=0.0349845136310349;AVGPVAL=0.0007129;AVGMIXED=0.00112145313650717;DIFFEFF=-0.003935;DIFFVAR=3.02382437836856e-05;DIFFVARACC=0.0133117819738401;DIFFPVAL=0.9464;DIFFMIXED=0.957219258538087;ANNOT=(FBgn0001311|kkv|INTRON)	GT	1/1	1/1	0/0	0/0	1/1	1/1	0/0	.	.	0/0	0/0	1/1	1/1	1/1	1/1	.	0/0	0/0	1/1	0/0	1/1	0/0	1/1	1/1	0/0	0/0	1/1	1/1	.	0/0	1/1	1/1	1/1	1/1	1/1	1/1	0/0	.	0/0	1/1	0/0	.	1/1	0/0	1/1	0/0	0/0	1/1	1/1	1/1	.	1/1	.	0/0	0/0	.	0/0	.	0/0	.	.	1/1	0/0	1/1	0/0	0/0	0/0	1/1	.	0/0	1/1	0/0	0/0	1/1	1/1	1/1	1/1	1/1	1/1	1/1	1/1	1/1	1/1	1/1	.	0/0	1/1	1/1	1/1	1/1	.	1/1	1/1	1/1	0/0	0/0	1/1	1/1	0/0	1/1	0/0	1/1	1/1	1/1	0/0	0/0	0/0	.	1/1	1/1	1/1	0/0	1/1	1/1	0/0	0/0	0/0	0/0	1/1	1/1	1/1	.	1/1	0/0	1/1	1/1	1/1	1/1	1/1	.	1/1	1/1	0/0	1/1	1/1	1/1	1/1	0/0	1/1	0/0	1/1	1/1	.	1/1	0/0	1/1	.	0/0	1/1	0/0	0/0	0/0	0/0	0/0	1/1	0/0	0/0	1/1	1/1	0/0	0/0	.	1/1	1/1	0/0	0/0	0/0	0/0	.	1/1	1/1	0/0	.	1/1	0/0
3R	1211607	3R_1211607_SNP	A	G	.	.	T5EFF=-0.1712;T5VAR=0.112836775893288;T5VARACC=0.0793207479451909;T5PVAL=1.167e-05;T5MIXED=7.224058828441e-06;T6EFF=-0.2229;T6VAR=0.0555186054670482;T6VARACC=0.0187958487528126;T6PVAL=0.002463;T6MIXED=0.00235125072467932;AVGEFF=-0.1971;AVGVAR=0.0870799845424021;AVGVARACC=0.0503300395586138;AVGPVAL=0.0001313;AVGMIXED=0.000116548759553034;DIFFEFF=0.05175;DIFFVAR=0.00509154545464436;DIFFVARACC=0.00287274586321024;DIFFPVAL=0.3654;DIFFMIXED=0.364919287175795;ANNOT=(FBgn0001311|kkv|INTRON),(FBgn0037320|CG14668|760 bp DOWNSTREAM)	GT	0/0	0/0	1/1	1/1	0/0	0/0	1/1	.	1/1	1/1	1/1	0/0	0/0	0/0	0/0	0/0	1/1	1/1	0/0	1/1	0/0	1/1	0/0	0/0	1/1	1/1	0/0	0/0	0/0	1/1	0/0	0/0	0/0	0/0	0/0	0/0	1/1	0/0	1/1	0/0	1/1	0/0	1/1	1/1	1/1	1/1	.	0/0	0/0	0/0	.	0/0	0/0	.	1/1	.	1/1	.	1/1	0/0	0/0	0/0	1/1	0/0	1/1	1/1	1/1	0/0	.	1/1	0/0	1/1	1/1	0/0	0/0	0/0	0/0	0/0	0/0	0/0	0/0	0/0	0/0	0/0	.	1/1	0/0	0/0	0/0	0/0	0/0	0/0	0/0	0/0	1/1	0/0	0/0	0/0	1/1	0/0	0/0	0/0	0/0	0/0	1/1	1/1	1/1	0/0	0/0	0/0	0/0	1/1	0/0	0/0	1/1	1/1	1/1	1/1	0/0	0/0	0/0	0/0	0/0	1/1	1/1	0/0	0/0	0/0	0/0	0/0	0/0	0/0	1/1	0/0	0/0	0/0	0/0	1/1	0/0	1/1	0/0	0/0	1/1	0/0	.	0/0	.	1/1	0/0	1/1	1/1	1/1	1/1	1/1	0/0	1/1	0/0	0/0	0/0	0/0	1/1	.	1/1	1/1	1/1	1/1	1/1	1/1	1/1	0/0	0/0	1/1	.	0/0	1/1
3R	13127277	3R_13127277_SNP	T	A	.	.	T5EFF=0.1986;T5VAR=0.0411716484031566;T5VARACC=0.00557638435296842;T5PVAL=0.007248;T5MIXED=0.0222589798910362;T6EFF=0.6277;T6VAR=0.111652717829829;T6VARACC=0.0420988942738029;T6PVAL=6.603e-06;T6MIXED=7.33464758283842e-06;AVGEFF=0.4131;AVGVAR=0.0996685489860104;AVGVARACC=0.0376542852320801;AVGPVAL=2.201e-05;AVGMIXED=5.29164972958415e-05;DIFFEFF=-0.4291;DIFFVAR=0.0867061804412378;DIFFVARACC=0.0273924274617212;DIFFPVAL=8.014e-05;DIFFMIXED=6.23757023584471e-05;ANNOT=(FBgn0263501|CR43490|12737 bp DOWNSTREAM),(FBgn0038498|beat-IIa|18449 bp UPSTREAM)	GT	0/0	0/0	0/0	0/0	0/0	0/0	1/1	0/0	0/0	0/0	0/0	0/0	0/0	0/0	0/0	0/0	0/0	0/0	0/0	0/0	0/0	0/0	0/0	0/0	1/1	0/0	0/0	0/0	0/0	0/0	0/0	0/0	0/0	0/0	0/0	0/0	0/0	0/0	0/0	0/0	0/0	0/0	0/0	0/0	0/0	0/0	0/0	0/0	0/0	0/0	0/0	0/0	0/0	0/0	0/0	0/0	0/0	0/0	0/0	0/0	0/0	1/1	0/0	0/0	0/0	0/0	0/0	0/0	0/0	0/0	0/0	0/0	0/0	0/0	0/0	0/0	0/0	0/0	0/0	0/0	1/1	1/1	0/0	0/0	0/0	0/0	0/0	0/0	1/1	0/0	0/0	0/0	0/0	0/0	0/0	0/0	0/0	0/0	0/0	0/0	0/0	0/0	0/0	1/1	0/0	0/0	0/0	0/0	0/0	0/0	1/1	0/0	0/0	0/0	0/0	0/0	0/0	0/0	0/0	0/0	0/0	0/0	0/0	0/0	0/0	0/0	0/0	0/0	0/0	1/1	0/0	0/0	0/0	0/0	0/0	0/0	0/0	0/0	0/0	1/1	0/0	0/0	0/0	1/1	0/0	0/0	0/0	0/0	0/0	0/0	0/0	0/0	0/0	0/0	0/0	0/0	0/0	0/0	0/0	0/0	0/0	0/0	0/0	0/0	0/0	.	0/0	0/0	0/0	0/0	0/0	0/0	0/0	0/0	0/0
3R	13127297	3R_13127297_SNP	A	T	.	.	T5EFF=0.1986;T5VAR=0.0411716484031566;T5VARACC=0.00557638435296842;T5PVAL=0.007248;T5MIXED=0.0222589798910362;T6EFF=0.6277;T6VAR=0.111652717829829;T6VARACC=0.0420988942738029;T6PVAL=6.603e-06;T6MIXED=7.33464758283842e-06;AVGEFF=0.4131;AVGVAR=0.0996685489860104;AVGVARACC=0.0376542852320801;AVGPVAL=2.201e-05;AVGMIXED=5.29164972958415e-05;DIFFEFF=-0.4291;DIFFVAR=0.0867061804412378;DIFFVARACC=0.0273924274617212;DIFFPVAL=8.014e-05;DIFFMIXED=6.23757023584471e-05;ANNOT=(FBgn0263501|CR43490|12757 bp DOWNSTREAM),(FBgn0038498|beat-IIa|18429 bp UPSTREAM)	GT	0/0	0/0	0/0	0/0	0/0	0/0	1/1	0/0	0/0	0/0	0/0	0/0	0/0	0/0	0/0	0/0	0/0	0/0	0/0	0/0	0/0	0/0	0/0	0/0	1/1	0/0	0/0	0/0	0/0	0/0	0/0	0/0	0/0	0/0	0/0	0/0	0/0	0/0	0/0	0/0	0/0	0/0	0/0	0/0	0/0	0/0	0/0	0/0	0/0	0/0	0/0	0/0	0/0	0/0	0/0	0/0	0/0	0/0	0/0	0/0	0/0	1/1	0/0	0/0	0/0	0/0	0/0	0/0	0/0	0/0	0/0	0/0	0/0	0/0	0/0	0/0	0/0	0/0	0/0	0/0	1/1	1/1	0/0	0/0	0/0	0/0	0/0	0/0	1/1	0/0	0/0	0/0	0/0	0/0	0/0	0/0	0/0	0/0	0/0	0/0	0/0	0/0	0/0	1/1	0/0	0/0	0/0	0/0	0/0	0/0	1/1	0/0	0/0	0/0	0/0	0/0	0/0	0/0	0/0	0/0	0/0	0/0	0/0	0/0	0/0	0/0	0/0	0/0	0/0	1/1	0/0	0/0	0/0	0/0	0/0	0/0	0/0	0/0	0/0	1/1	0/0	0/0	0/0	1/1	0/0	0/0	0/0	0/0	0/0	0/0	0/0	0/0	0/0	0/0	0/0	0/0	0/0	0/0	0/0	0/0	0/0	0/0	0/0	0/0	0/0	.	0/0	0/0	0/0	0/0	0/0	0/0	0/0	0/0	0/0
3R	13515105	3R_13515105_SNP	G	A	.	.	T5EFF=-0.3896;T5VAR=0.126195022814205;T5VARACC=0.0792931645023088;T5PVAL=1.863e-06;T5MIXED=2.76365561447021e-06;T6EFF=-0.4129;T6VAR=0.040627796640739;T6VARACC=0.0204010132856126;T6PVAL=0.008202;T6MIXED=0.00746226768437771;AVGEFF=-0.4012;AVGVAR=0.0777829367076857;AVGVARACC=0.0726822332906689;AVGPVAL=0.0002209;AVGMIXED=0.000221354945443098;DIFFEFF=0.02332;DIFFVAR=0.000215998984100888;DIFFVARACC=0.0016929718823131;DIFFPVAL=0.8487;DIFFMIXED=0.829701367709715;ANNOT=(FBgn0261885|osa|UTR_3_PRIME)	GT	0/0	0/0	0/0	1/1	0/0	0/0	0/0	0/0	0/0	0/0	0/0	0/0	0/0	0/0	0/0	0/0	0/0	0/0	0/0	0/0	0/0	0/0	0/0	0/0	0/0	0/0	1/1	0/0	0/0	0/0	0/0	0/0	0/0	0/0	0/0	0/0	0/0	0/0	0/0	0/0	.	0/0	1/1	0/0	0/0	0/0	0/0	0/0	0/0	0/0	0/0	0/0	0/0	0/0	0/0	0/0	0/0	0/0	0/0	0/0	0/0	0/0	1/1	.	1/1	0/0	0/0	0/0	0/0	0/0	0/0	0/0	0/0	0/0	0/0	0/0	0/0	0/0	0/0	0/0	0/0	0/0	0/0	0/0	0/0	0/0	0/0	0/0	0/0	0/0	0/0	0/0	0/0	0/0	0/0	0/0	0/0	0/0	0/0	1/1	0/0	0/0	0/0	0/0	0/0	0/0	0/0	0/0	0/0	0/0	0/0	0/0	0/0	0/0	0/0	0/0	0/0	0/0	0/0	0/0	0/0	.	0/0	0/0	0/0	0/0	0/0	0/0	0/0	0/0	0/0	0/0	0/0	0/0	0/0	0/0	0/0	0/0	0/0	0/0	1/1	0/0	0/0	0/0	0/0	0/0	0/0	1/1	0/0	0/0	0/0	0/0	0/0	0/0	0/0	0/0	0/0	0/0	0/0	0/0	0/0	0/0	0/0	0/0	0/0	0/0	0/0	0/0	0/0	0/0	0/0	1/1	0/0	.	0/0
3R	14181488	3R_14181488_SNP	G	A	.	.	T5EFF=-0.4077;T5VAR=0.136705652972508;T5VARACC=0.0961910841517959;T5PVAL=6.97e-07;T5MIXED=1.06277139271228e-06;T6EFF=-0.4612;T6VAR=0.050244583515162;T6VARACC=0.0236455325861069;T6PVAL=0.003298;T6MIXED=0.0027782252403936;AVGEFF=-0.4344;AVGVAR=0.0900087493251078;AVGVARACC=0.0443312005394325;AVGPVAL=7.044e-05;AVGMIXED=6.6726634999904e-05;DIFFEFF=0.05351;DIFFVAR=0.00114031566833193;DIFFVARACC=0.000443129871022352;DIFFPVAL=0.662;DIFFMIXED=0.641058992521659;ANNOT=(FBgn0038606|CG15803|UTR_5_PRIME)	GT	0/0	0/0	0/0	0/0	0/0	0/0	0/0	1/1	0/0	0/0	0/0	0/0	0/0	0/0	0/0	1/1	0/0	0/0	0/0	0/0	0/0	0/0	0/0	0/0	0/0	1/1	1/1	0/0	0/0	0/0	0/0	0/0	0/0	0/0	0/0	0/0	0/0	0/0	0/0	0/0	0/0	0/0	0/0	0/0	0/0	0/0	0/0	0/0	0/0	0/0	0/0	0/0	0/0	0/0	0/0	0/0	0/0	.	0/0	0/0	0/0	0/0	0/0	0/0	1/1	0/0	0/0	0/0	.	0/0	0/0	0/0	0/0	0/0	0/0	0/0	0/0	0/0	0/0	0/0	0/0	0/0	0/0	0/0	.	0/0	0/0	0/0	0/0	0/0	0/0	0/0	0/0	0/0	0/0	0/0	0/0	0/0	1/1	.	0/0	0/0	0/0	0/0	0/0	0/0	0/0	0/0	0/0	0/0	0/0	0/0	0/0	1/1	0/0	0/0	0/0	0/0	0/0	0/0	0/0	.	0/0	0/0	0/0	0/0	0/0	0/0	0/0	0/0	0/0	0/0	0/0	0/0	0/0	0/0	0/0	0/0	0/0	0/0	1/1	0/0	0/0	0/0	0/0	0/0	0/0	0/0	0/0	0/0	0/0	0/0	0/0	0/0	0/0	0/0	0/0	0/0	0/0	0/0	0/0	0/0	0/0	0/0	0/0	1/1	0/0	0/0	0/0	0/0	0/0	0/0	0/0	0/0	0/0
3R	17063120	3R_17063120_SNP	T	C	.	.	T5EFF=0.213;T5VAR=0.063973899795967;T5VARACC=NA;T5PVAL=0.0008448;T5MIXED=0.00131393223644011;T6EFF=0.5192;T6VAR=0.108254776955567;T6VARACC=NA;T6PVAL=1.113e-05;T6MIXED=9.07973816404031e-06;AVGEFF=0.3661;AVGVAR=0.109180894399326;AVGVARACC=NA;AVGPVAL=1.016e-05;AVGMIXED=9.21915221580289e-06;DIFFEFF=-0.3062;DIFFVAR=0.0629980644432695;DIFFVARACC=NA;DIFFPVAL=0.0009287;DIFFMIXED=0.000787958270108446;ANNOT=(FBgn0000527|e|UPSTREAM)	GT	0/0	0/0	0/0	0/0	0/0	0/0	1/1	0/0	0/0	0/0	0/0	0/0	0/0	0/0	0/0	0/0	0/0	0/0	1/1	0/0	0/0	0/0	0/0	0/0	0/0	0/0	0/0	0/0	0/0	0/0	0/0	0/0	0/0	0/0	0/0	0/0	0/0	0/0	0/0	0/0	0/0	0/0	0/0	0/0	0/0	0/0	0/0	0/0	0/0	1/1	0/0	0/0	0/0	0/0	0/0	0/0	0/0	.	0/0	0/0	0/0	1/1	0/0	1/1	0/0	0/0	0/0	0/0	.	0/0	0/0	1/1	0/0	1/1	0/0	0/0	0/0	0/0	0/0	0/0	.	0/0	0/0	0/0	0/0	0/0	0/0	0/0	0/0	0/0	0/0	1/1	1/1	0/0	0/0	0/0	.	0/0	0/0	0/0	0/0	0/0	0/0	0/0	0/0	0/0	0/0	0/0	0/0	0/0	0/0	0/0	0/0	0/0	1/1	0/0	0/0	1/1	0/0	0/0	0/0	0/0	0/0	0/0	0/0	0/0	0/0	0/0	0/0	0/0	0/0	0/0	0/0	1/1	0/0	0/0	0/0	0/0	0/0	0/0	0/0	0/0	1/1	0/0	0/0	0/0	0/0	0/0	0/0	0/0	0/0	0/0	0/0	0/0	0/0	0/0	0/0	0/0	1/1	0/0	0/0	1/1	0/0	0/0	0/0	0/0	0/0	0/0	1/1	0/0	0/0	0/0	0/0	0/0	0/0
3R	18413010	3R_18413010_SNP	A	G	.	.	T5EFF=0.1394;T5VAR=0.0700614645671041;T5VARACC=0.0148530931123564;T5PVAL=0.0009088;T5MIXED=0.00012471476702292;T6EFF=0.3078;T6VAR=0.101610566595057;T6VARACC=0.0351467950095085;T6PVAL=5.598e-05;T6MIXED=2.44194876832606e-05;AVGEFF=0.2236;AVGVAR=0.107170213987202;AVGVARACC=0.0384015021622411;AVGPVAL=3.41e-05;AVGMIXED=5.41370297637533e-06;DIFFEFF=-0.1684;DIFFVAR=0.0512270893188427;DIFFVARACC=0.0119232572830182;DIFFPVAL=0.004764;DIFFMIXED=0.00419839108349212;ANNOT=(FBgn0051158|Efa6|467 bp UPSTREAM),(FBgn0014949|btn|663 bp UPSTREAM)	GT	0/0	1/1	0/0	0/0	0/0	0/0	0/0	0/0	0/0	0/0	1/1	0/0	0/0	0/0	0/0	0/0	1/1	0/0	0/0	1/1	0/0	.	0/0	0/0	1/1	0/0	.	0/0	0/0	1/1	0/0	0/0	.	0/0	1/1	1/1	.	0/0	0/0	.	0/0	0/0	0/0	1/1	0/0	1/1	0/0	0/0	0/0	1/1	1/1	1/1	0/0	0/0	1/1	0/0	0/0	.	.	1/1	0/0	1/1	0/0	1/1	1/1	1/1	0/0	1/1	0/0	1/1	0/0	0/0	1/1	0/0	0/0	1/1	0/0	0/0	1/1	0/0	1/1	0/0	0/0	1/1	0/0	0/0	0/0	1/1	1/1	1/1	.	0/0	0/0	0/0	1/1	0/0	0/0	0/0	0/0	1/1	.	1/1	1/1	1/1	.	0/0	0/0	0/0	.	0/0	1/1	1/1	1/1	1/1	0/0	1/1	0/0	.	0/0	.	1/1	.	0/0	0/0	0/0	0/0	0/0	0/0	0/0	0/0	.	0/0	0/0	1/1	0/0	1/1	.	0/0	0/0	0/0	1/1	1/1	1/1	0/0	1/1	0/0	0/0	0/0	0/0	0/0	1/1	1/1	0/0	0/0	1/1	0/0	.	0/0	1/1	.	1/1	0/0	0/0	0/0	0/0	0/0	0/0	0/0	1/1	.	1/1	0/0	.	.	0/0
3R	18413146	3R_18413146_SNP	T	A	.	.	T5EFF=0.1242;T5VAR=0.0579804577704638;T5VARACC=0.00888194024981492;T5PVAL=0.002092;T5MIXED=0.000431720575482994;T6EFF=0.3121;T6VAR=0.105705237338805;T6VARACC=0.0350043953731466;T6PVAL=2.575e-05;T6MIXED=9.56443346825833e-06;AVGEFF=0.2181;AVGVAR=0.104873339329185;AVGVARACC=0.0436605000034548;AVGPVAL=2.783e-05;AVGMIXED=4.44426052141463e-06;DIFFEFF=-0.1879;DIFFVAR=0.0630499270040012;DIFFVARACC=0.0182446335096144;DIFFPVAL=0.001314;DIFFMIXED=0.000991770473080221;ANNOT=(FBgn0051158|Efa6|331 bp UPSTREAM),(FBgn0014949|btn|779 bp UPSTREAM)	GT	0/0	.	0/0	0/0	0/0	0/0	0/0	0/0	0/0	0/0	1/1	0/0	0/0	0/0	0/0	.	1/1	0/0	0/0	1/1	0/0	1/1	0/0	0/0	1/1	0/0	.	0/0	0/0	1/1	0/0	0/0	.	0/0	.	1/1	0/0	0/0	0/0	0/0	.	0/0	0/0	1/1	0/0	1/1	0/0	0/0	0/0	1/1	1/1	1/1	0/0	0/0	1/1	0/0	0/0	0/0	.	1/1	0/0	1/1	0/0	.	1/1	1/1	0/0	1/1	0/0	1/1	0/0	0/0	1/1	0/0	0/0	1/1	0/0	0/0	1/1	0/0	1/1	0/0	0/0	1/1	0/0	0/0	0/0	1/1	1/1	1/1	0/0	0/0	0/0	0/0	1/1	0/0	0/0	0/0	0/0	1/1	1/1	1/1	1/1	1/1	1/1	0/0	0/0	0/0	0/0	0/0	1/1	1/1	1/1	1/1	0/0	1/1	0/0	0/0	0/0	.	1/1	.	0/0	0/0	0/0	0/0	0/0	0/0	0/0	0/0	1/1	0/0	0/0	1/1	0/0	1/1	.	0/0	0/0	0/0	1/1	1/1	1/1	0/0	1/1	0/0	0/0	0/0	0/0	0/0	1/1	1/1	0/0	0/0	1/1	0/0	.	0/0	1/1	1/1	1/1	0/0	0/0	0/0	0/0	0/0	0/0	0/0	1/1	.	1/1	0/0	1/1	.	0/0
3R	18413268	3R_18413268_SNP	C	T	.	.	T5EFF=0.1139;T5VAR=0.0497665492828641;T5VARACC=0.00228983093141444;T5PVAL=0.005122;T5MIXED=0.0016270440433275;T6EFF=0.3153;T6VAR=0.107973035008558;T6VARACC=0.0287475558812336;T6PVAL=2.814e-05;T6MIXED=7.64737319372944e-06;AVGEFF=0.2146;AVGVAR=0.101432847143107;AVGVARACC=0.0333310056490392;AVGPVAL=5.08e-05;AVGMIXED=6.8480395643944e-06;DIFFEFF=-0.2014;DIFFVAR=0.0742177502905127;DIFFVARACC=0.014885047415767;DIFFPVAL=0.0005805;DIFFMIXED=0.000395762049204695;ANNOT=(FBgn0051158|Efa6|209 bp UPSTREAM),(FBgn0014949|btn|921 bp UPSTREAM)	GT	0/0	.	0/0	.	0/0	0/0	0/0	0/0	0/0	0/0	.	0/0	0/0	0/0	0/0	0/0	1/1	0/0	0/0	1/1	0/0	1/1	0/0	0/0	1/1	0/0	1/1	0/0	0/0	1/1	0/0	0/0	.	0/0	.	1/1	0/0	0/0	0/0	0/0	1/1	.	0/0	1/1	0/0	1/1	0/0	0/0	0/0	1/1	1/1	1/1	0/0	0/0	1/1	0/0	0/0	0/0	1/1	1/1	0/0	1/1	0/0	.	1/1	1/1	0/0	1/1	.	.	0/0	0/0	1/1	0/0	0/0	1/1	0/0	0/0	1/1	0/0	1/1	0/0	0/0	1/1	0/0	0/0	0/0	1/1	1/1	.	0/0	0/0	.	0/0	1/1	0/0	0/0	0/0	0/0	1/1	1/1	1/1	1/1	1/1	1/1	0/0	0/0	0/0	0/0	0/0	1/1	1/1	1/1	1/1	0/0	1/1	0/0	1/1	0/0	1/1	1/1	.	0/0	0/0	0/0	0/0	0/0	.	.	0/0	1/1	0/0	0/0	1/1	0/0	1/1	.	1/1	0/0	0/0	1/1	1/1	1/1	0/0	.	0/0	0/0	0/0	0/0	0/0	1/1	1/1	0/0	0/0	1/1	0/0	.	0/0	1/1	1/1	1/1	0/0	0/0	0/0	0/0	0/0	0/0	0/0	1/1	.	1/1	0/0	1/1	.	0/0
3R	18449065	3R_18449065_SNP	C	T	.	.	T5EFF=-0.3656;T5VAR=0.108778209803505;T5VARACC=0.0889432343842362;T5PVAL=8.306e-06;T5MIXED=7.22681961019517e-06;T6EFF=-0.3674;T6VAR=0.0312021895516924;T6VARACC=0.026358190498084;T6PVAL=0.01937;T6MIXED=0.0204691935273379;AVGEFF=-0.3665;AVGVAR=0.0628709087311203;AVGVARACC=0.0694548113289604;AVGPVAL=0.0008174;AVGMIXED=0.000995057910778616;DIFFEFF=0.001741;DIFFVAR=1.18338108875853e-06;DIFFVARACC=0.000339839588302748;DIFFPVAL=0.9886;DIFFMIXED=0.998726466437671;ANNOT=(FBgn0020278|loco|INTRON)	GT	0/0	0/0	0/0	1/1	0/0	0/0	0/0	0/0	0/0	0/0	0/0	0/0	0/0	0/0	0/0	0/0	0/0	0/0	0/0	0/0	0/0	0/0	0/0	0/0	0/0	0/0	0/0	0/0	0/0	0/0	0/0	0/0	0/0	0/0	0/0	0/0	0/0	0/0	0/0	0/0	0/0	0/0	1/1	0/0	0/0	0/0	0/0	0/0	0/0	0/0	0/0	0/0	0/0	0/0	0/0	0/0	0/0	0/0	1/1	0/0	0/0	0/0	0/0	0/0	0/0	0/0	0/0	0/0	0/0	0/0	0/0	0/0	0/0	0/0	0/0	0/0	0/0	0/0	0/0	0/0	0/0	0/0	0/0	0/0	0/0	1/1	0/0	0/0	0/0	0/0	0/0	1/1	0/0	0/0	0/0	0/0	0/0	0/0	0/0	0/0	0/0	0/0	0/0	0/0	0/0	0/0	1/1	0/0	0/0	1/1	0/0	0/0	0/0	0/0	0/0	0/0	0/0	0/0	0/0	0/0	0/0	0/0	0/0	0/0	0/0	0/0	0/0	0/0	0/0	0/0	0/0	0/0	0/0	0/0	0/0	0/0	0/0	1/1	0/0	0/0	0/0	0/0	0/0	0/0	0/0	0/0	0/0	1/1	0/0	0/0	0/0	0/0	0/0	0/0	0/0	0/0	0/0	0/0	0/0	0/0	0/0	0/0	0/0	0/0	0/0	0/0	0/0	0/0	0/0	0/0	0/0	0/0	0/0	0/0	0/0
3R	20283209	3R_20283209_SNP	T	G	.	.	T5EFF=-0.1716;T5VAR=0.0743916779662928;T5VARACC=0.0376616281228247;T5PVAL=0.0003624;T5MIXED=0.000300792710403114;T6EFF=-0.3703;T6VAR=0.101426620684796;T6VARACC=0.0251418207713417;T6PVAL=2.735e-05;T6MIXED=2.0340479281088e-05;AVGEFF=-0.271;AVGVAR=0.109097871097097;AVGVARACC=0.0567775217208752;AVGPVAL=1.305e-05;AVGMIXED=8.55269506477984e-06;DIFFEFF=0.1986;DIFFVAR=0.0490900933947166;DIFFVARACC=0.0150213713325449;DIFFPVAL=0.004008;DIFFMIXED=0.00362748960293676;ANNOT=(FBgn0000036|nAcRalpha-96Aa|UPSTREAM)	GT	1/1	1/1	1/1	0/0	1/1	1/1	1/1	0/0	1/1	0/0	1/1	1/1	0/0	1/1	1/1	0/0	1/1	1/1	1/1	0/0	1/1	1/1	1/1	0/0	1/1	1/1	1/1	1/1	1/1	1/1	1/1	0/0	1/1	1/1	1/1	1/1	1/1	0/0	1/1	0/0	1/1	.	1/1	1/1	1/1	1/1	1/1	1/1	1/1	1/1	1/1	1/1	1/1	1/1	1/1	0/0	1/1	.	1/1	1/1	1/1	1/1	1/1	.	1/1	1/1	1/1	1/1	.	0/0	0/0	1/1	1/1	1/1	0/0	0/0	0/0	1/1	1/1	.	0/0	1/1	0/0	1/1	.	1/1	1/1	1/1	1/1	1/1	1/1	1/1	1/1	1/1	0/0	1/1	1/1	1/1	1/1	1/1	1/1	1/1	1/1	1/1	1/1	1/1	1/1	1/1	1/1	1/1	1/1	1/1	1/1	1/1	0/0	1/1	1/1	1/1	1/1	1/1	1/1	1/1	1/1	0/0	1/1	1/1	1/1	.	1/1	1/1	1/1	1/1	0/0	1/1	1/1	1/1	1/1	1/1	1/1	1/1	1/1	1/1	1/1	1/1	1/1	1/1	0/0	0/0	0/0	0/0	1/1	0/0	1/1	0/0	0/0	0/0	1/1	0/0	1/1	1/1	1/1	1/1	1/1	1/1	0/0	1/1	1/1	1/1	1/1	1/1	1/1	1/1	.	1/1	1/1
3R	21006399	3R_21006399_SNP	A	C	.	.	T5EFF=-0.2776;T5VAR=0.118143563962139;T5VARACC=0.120028530459002;T5PVAL=6.181e-06;T5MIXED=1.0272117338469e-05;T6EFF=-0.1737;T6VAR=0.0131770485881519;T6VARACC=0.0174304844392658;T6PVAL=0.1421;T6MIXED=0.140956183734427;AVGEFF=-0.2256;AVGVAR=0.0452736798404737;AVGVARACC=0.0659527429934468;AVGPVAL=0.006072;AVGMIXED=0.00672196129055068;DIFFEFF=-0.1039;DIFFVAR=0.00779755492744334;DIFFVARACC=0.00386480422428359;DIFFPVAL=0.2594;DIFFMIXED=0.253269630573038;ANNOT=(FBgn0083946|lobo|INTRON)	GT	1/1	1/1	1/1	0/0	.	1/1	1/1	1/1	1/1	1/1	1/1	1/1	1/1	1/1	1/1	1/1	1/1	1/1	1/1	0/0	1/1	1/1	1/1	1/1	1/1	1/1	0/0	1/1	1/1	1/1	1/1	1/1	.	1/1	.	1/1	1/1	1/1	1/1	1/1	.	1/1	1/1	1/1	1/1	0/0	1/1	1/1	1/1	1/1	1/1	1/1	1/1	1/1	1/1	1/1	0/0	1/1	1/1	1/1	1/1	1/1	0/0	.	0/0	1/1	1/1	0/0	1/1	1/1	1/1	1/1	1/1	1/1	1/1	1/1	1/1	1/1	1/1	1/1	1/1	1/1	1/1	1/1	1/1	1/1	1/1	1/1	0/0	1/1	1/1	1/1	1/1	1/1	0/0	1/1	1/1	1/1	1/1	1/1	1/1	1/1	1/1	1/1	1/1	1/1	0/0	1/1	1/1	1/1	1/1	0/0	1/1	0/0	1/1	1/1	1/1	1/1	0/0	1/1	1/1	1/1	1/1	1/1	1/1	1/1	1/1	1/1	1/1	1/1	1/1	1/1	1/1	1/1	1/1	.	.	.	1/1	1/1	1/1	1/1	1/1	1/1	1/1	1/1	1/1	0/0	1/1	1/1	1/1	1/1	1/1	1/1	1/1	.	.	1/1	1/1	1/1	1/1	1/1	1/1	1/1	1/1	0/0	1/1	1/1	1/1	1/1	1/1	1/1	0/0	1/1	1/1
3R	22549473	3R_22549473_SNP	A	T	.	.	T5EFF=0.1684;T5VAR=0.106989722320159;T5VARACC=0.0367833458037787;T5PVAL=2.153e-05;T5MIXED=2.6654580767978e-05;T6EFF=0.286;T6VAR=0.0903088611424683;T6VARACC=0.0541799849651893;T6PVAL=0.0001021;T6MIXED=0.0001252329108338;AVGEFF=0.2272;AVGVAR=0.115149448638969;AVGVARACC=0.0706285624206719;AVGPVAL=9.99e-06;AVGMIXED=1.6242085032246e-05;DIFFEFF=-0.1176;DIFFVAR=0.0252074338721923;DIFFVARACC=0.0107038081003527;DIFFPVAL=0.0436;DIFFMIXED=0.0412729147935039;ANNOT=(FBgn0039451|CG6420|INTRON)	GT	0/0	0/0	1/1	0/0	0/0	0/0	0/0	0/0	0/0	0/0	0/0	1/1	1/1	1/1	1/1	0/0	0/0	1/1	0/0	0/0	1/1	0/0	0/0	0/0	0/0	0/0	0/0	0/0	1/1	0/0	0/0	0/0	.	0/0	0/0	1/1	.	0/0	.	1/1	.	.	0/0	0/0	1/1	1/1	1/1	0/0	0/0	1/1	0/0	0/0	1/1	0/0	0/0	0/0	0/0	.	1/1	1/1	0/0	1/1	0/0	.	0/0	0/0	0/0	1/1	0/0	1/1	0/0	1/1	1/1	0/0	1/1	1/1	1/1	0/0	1/1	1/1	1/1	1/1	0/0	0/0	0/0	0/0	1/1	0/0	1/1	0/0	0/0	1/1	1/1	0/0	1/1	0/0	0/0	1/1	1/1	0/0	0/0	0/0	1/1	1/1	0/0	0/0	0/0	1/1	0/0	0/0	0/0	1/1	0/0	0/0	0/0	1/1	0/0	0/0	0/0	.	0/0	1/1	1/1	1/1	0/0	0/0	0/0	1/1	0/0	1/1	0/0	1/1	0/0	0/0	0/0	0/0	0/0	.	0/0	1/1	0/0	0/0	0/0	1/1	1/1	0/0	.	0/0	1/1	0/0	1/1	0/0	1/1	0/0	0/0	0/0	.	1/1	1/1	0/0	1/1	0/0	0/0	0/0	0/0	0/0	0/0	0/0	1/1	.	1/1	0/0	.	0/0	0/0
3R	22549474	3R_22549474_SNP	C	G	.	.	T5EFF=0.1688;T5VAR=0.107602099245907;T5VARACC=0.0357412898920657;T5PVAL=1.915e-05;T5MIXED=3.75884738479013e-05;T6EFF=0.3051;T6VAR=0.101927527849858;T6VARACC=0.0540206764004445;T6PVAL=3.268e-05;T6MIXED=3.70152916120402e-05;AVGEFF=0.2369;AVGVAR=0.124571721590459;AVGVARACC=0.0691622054738606;AVGPVAL=3.818e-06;AVGMIXED=6.36109213288074e-06;DIFFEFF=-0.1363;DIFFVAR=0.0335690092975226;DIFFVARACC=0.015027463557875;DIFFPVAL=0.01923;DIFFMIXED=0.0172401602412449;ANNOT=(FBgn0039451|CG6420|INTRON)	GT	0/0	0/0	1/1	0/0	0/0	0/0	0/0	0/0	0/0	0/0	0/0	1/1	1/1	1/1	1/1	0/0	0/0	1/1	0/0	0/0	1/1	0/0	0/0	0/0	0/0	0/0	0/0	0/0	1/1	0/0	0/0	0/0	.	0/0	0/0	1/1	.	0/0	0/0	1/1	.	.	0/0	0/0	1/1	1/1	.	0/0	0/0	1/1	0/0	0/0	1/1	0/0	0/0	0/0	0/0	.	1/1	1/1	0/0	1/1	0/0	.	0/0	0/0	0/0	1/1	0/0	1/1	0/0	1/1	1/1	0/0	1/1	1/1	1/1	0/0	1/1	.	1/1	1/1	0/0	0/0	0/0	0/0	1/1	0/0	1/1	0/0	0/0	1/1	1/1	0/0	1/1	0/0	0/0	1/1	1/1	0/0	0/0	0/0	1/1	1/1	0/0	0/0	0/0	1/1	0/0	0/0	0/0	1/1	0/0	0/0	0/0	1/1	0/0	0/0	0/0	.	0/0	1/1	1/1	1/1	0/0	0/0	0/0	1/1	0/0	1/1	0/0	1/1	0/0	0/0	0/0	0/0	0/0	.	0/0	1/1	0/0	0/0	0/0	1/1	1/1	0/0	1/1	0/0	1/1	0/0	1/1	0/0	1/1	0/0	0/0	0/0	.	1/1	1/1	0/0	1/1	0/0	0/0	0/0	0/0	0/0	0/0	0/0	1/1	.	1/1	0/0	1/1	0/0	0/0
3R	22822290	3R_22822290_SNP	T	C	.	.	T5EFF=-0.2348;T5VAR=0.117119174414868;T5VARACC=0.0856975139173369;T5PVAL=5.612e-06;T5MIXED=5.74181476604701e-06;T6EFF=-0.2341;T6VAR=0.0334582874212893;T6VARACC=0.0416335911920517;T6PVAL=0.01763;T6MIXED=0.0114028041765377;AVGEFF=-0.2345;AVGVAR=0.0677976506662443;AVGVARACC=0.0701531224470104;AVGPVAL=0.0006527;AVGMIXED=0.000356094850643835;DIFFEFF=-0.000759;DIFFVAR=5.91470717704643e-07;DIFFVARACC=0.000441970842365302;DIFFPVAL=0.9921;DIFFMIXED=0.971593192553419;ANNOT=(FBgn0039469|TwdlC|126 bp DOWNSTREAM),(FBgn0004842|NepYr|412 bp UPSTREAM)	GT	0/0	0/0	0/0	0/0	0/0	0/0	0/0	0/0	0/0	0/0	0/0	0/0	0/0	0/0	0/0	0/0	0/0	0/0	0/0	0/0	0/0	0/0	0/0	0/0	0/0	0/0	1/1	1/1	0/0	1/1	0/0	0/0	.	1/1	.	0/0	0/0	0/0	0/0	0/0	.	0/0	1/1	1/1	0/0	0/0	0/0	0/0	0/0	0/0	1/1	1/1	0/0	0/0	1/1	0/0	0/0	0/0	0/0	0/0	0/0	0/0	1/1	0/0	1/1	0/0	0/0	0/0	0/0	0/0	0/0	0/0	0/0	0/0	0/0	0/0	0/0	0/0	.	0/0	0/0	0/0	0/0	1/1	0/0	0/0	0/0	0/0	0/0	0/0	.	0/0	1/1	0/0	0/0	0/0	0/0	0/0	0/0	0/0	1/1	0/0	0/0	0/0	1/1	1/1	0/0	0/0	0/0	0/0	0/0	0/0	1/1	1/1	1/1	0/0	0/0	0/0	0/0	0/0	0/0	.	0/0	1/1	0/0	0/0	0/0	0/0	0/0	0/0	0/0	0/0	1/1	0/0	0/0	0/0	0/0	0/0	0/0	0/0	0/0	0/0	.	0/0	0/0	0/0	0/0	1/1	0/0	0/0	0/0	0/0	0/0	0/0	0/0	0/0	0/0	0/0	0/0	1/1	0/0	0/0	0/0	0/0	1/1	1/1	0/0	0/0	0/0	0/0	0/0	1/1	0/0	0/0	0/0
3R	23828912	3R_23828912_SNP	G	C	.	.	T5EFF=-0.0395;T5VAR=0.00353554002797657;T5VARACC=0.00456255999879668;T5PVAL=0.4467;T5MIXED=0.314047078594188;T6EFF=-0.3627;T6VAR=0.0852583579624324;T6VARACC=0.0657958171221488;T6PVAL=0.000135;T6MIXED=8.73997846367032e-05;AVGEFF=-0.2011;AVGVAR=0.0530171909772466;AVGVARACC=0.0567926460416914;AVGPVAL=0.002841;AVGMIXED=0.00156454000755668;DIFFEFF=0.3232;DIFFVAR=0.113842431385541;DIFFVARACC=0.0731090388197477;DIFFPVAL=8.774e-06;DIFFMIXED=5.86772942533609e-06;ANNOT=(FBgn0039568|CG4815|12439 bp UPSTREAM),(FBgn0085391|CG34362|23425 bp DOWNSTREAM)	GT	0/0	0/0	0/0	0/0	.	1/1	1/1	0/0	0/0	1/1	0/0	1/1	1/1	0/0	1/1	0/0	1/1	0/0	0/0	1/1	0/0	0/0	0/0	0/0	0/0	0/0	0/0	0/0	0/0	0/0	0/0	1/1	.	0/0	0/0	1/1	0/0	0/0	0/0	0/0	0/0	0/0	0/0	0/0	0/0	0/0	1/1	0/0	0/0	0/0	0/0	0/0	0/0	0/0	0/0	1/1	0/0	0/0	0/0	0/0	.	0/0	0/0	0/0	0/0	0/0	0/0	0/0	0/0	0/0	0/0	0/0	0/0	0/0	0/0	0/0	0/0	0/0	0/0	0/0	0/0	0/0	0/0	0/0	.	0/0	0/0	0/0	1/1	0/0	0/0	1/1	0/0	0/0	0/0	0/0	0/0	0/0	0/0	0/0	0/0	.	0/0	0/0	0/0	0/0	1/1	0/0	.	0/0	0/0	0/0	0/0	0/0	1/1	0/0	0/0	1/1	1/1	.	0/0	.	0/0	0/0	0/0	0/0	0/0	0/0	1/1	0/0	0/0	0/0	1/1	0/0	0/0	0/0	0/0	0/0	0/0	0/0	0/0	0/0	0/0	0/0	0/0	0/0	1/1	0/0	1/1	0/0	0/0	1/1	0/0	1/1	0/0	1/1	0/0	0/0	0/0	0/0	0/0	0/0	1/1	1/1	1/1	0/0	0/0	0/0	0/0	0/0	0/0	0/0	0/0	.	0/0
3R	25139132	3R_25139132_SNP	C	A	.	.	T5EFF=-0.1673;T5VAR=0.110121991208737;T5VARACC=0.113490350189373;T5PVAL=3.347e-05;T5MIXED=0.000118423636919351;T6EFF=-0.3091;T6VAR=0.095553100437352;T6VARACC=0.0833906157757474;T6PVAL=0.0001186;T6MIXED=4.87132556113352e-05;AVGEFF=-0.2382;AVGVAR=0.11988879203938;AVGVARACC=0.12806309207267;AVGPVAL=1.423e-05;AVGMIXED=6.8585154093238e-06;DIFFEFF=0.1418;DIFFVAR=0.0326948913614934;DIFFVARACC=0.0234005124325027;DIFFPVAL=0.02681;DIFFMIXED=0.0179058083320205;ANNOT=(FBgn0039659|CG14506|16230 bp UPSTREAM),(FBgn0015622|Cnx99A|1844 bp UPSTREAM)	GT	.	.	0/0	1/1	0/0	0/0	0/0	0/0	.	.	.	0/0	1/1	0/0	1/1	1/1	0/0	1/1	0/0	1/1	0/0	.	0/0	.	0/0	0/0	.	.	.	.	0/0	1/1	0/0	0/0	0/0	0/0	.	.	0/0	0/0	0/0	0/0	1/1	1/1	1/1	.	.	0/0	0/0	0/0	1/1	1/1	0/0	1/1	1/1	0/0	1/1	.	0/0	0/0	0/0	0/0	1/1	0/0	1/1	1/1	1/1	1/1	0/0	0/0	0/0	0/0	0/0	0/0	1/1	0/0	1/1	0/0	0/0	0/0	0/0	0/0	0/0	1/1	0/0	0/0	0/0	.	1/1	0/0	.	0/0	0/0	0/0	1/1	.	1/1	0/0	1/1	1/1	0/0	0/0	0/0	0/0	.	.	1/1	0/0	0/0	0/0	0/0	0/0	1/1	1/1	1/1	0/0	0/0	0/0	1/1	0/0	1/1	0/0	0/0	0/0	0/0	0/0	0/0	0/0	0/0	.	0/0	0/0	1/1	1/1	0/0	0/0	0/0	0/0	0/0	0/0	0/0	0/0	0/0	0/0	0/0	0/0	0/0	0/0	0/0	0/0	0/0	1/1	0/0	1/1	1/1	1/1	.	0/0	0/0	1/1	0/0	1/1	0/0	0/0	1/1	1/1	1/1	1/1	1/1	0/0	0/0	.	0/0	0/0	.
3R	25139342	3R_25139342_SNP	T	C	.	.	T5EFF=-0.1713;T5VAR=0.119101458506438;T5VARACC=0.1308281783156;T5PVAL=2.777e-05;T5MIXED=8.4683520812696e-05;T6EFF=-0.3352;T6VAR=0.110039805565019;T6VARACC=0.0890571622774798;T6PVAL=5.862e-05;T6MIXED=3.8891884497398e-05;AVGEFF=-0.2532;AVGVAR=0.136024495217274;AVGVARACC=0.131381908947325;AVGPVAL=6.791e-06;AVGMIXED=8.85995893024228e-06;DIFFEFF=0.1639;DIFFVAR=0.041396967225234;DIFFVARACC=0.0275033333273654;DIFFPVAL=0.01553;DIFFMIXED=0.0101163172883267;ANNOT=(FBgn0039659|CG14506|16440 bp UPSTREAM),(FBgn0015622|Cnx99A|1634 bp UPSTREAM)	GT	0/0	0/0	0/0	1/1	0/0	0/0	0/0	.	0/0	0/0	0/0	0/0	1/1	0/0	1/1	.	0/0	1/1	.	1/1	0/0	.	0/0	.	0/0	0/0	.	1/1	.	1/1	0/0	1/1	0/0	0/0	0/0	0/0	.	.	0/0	0/0	0/0	.	1/1	1/1	1/1	.	0/0	0/0	0/0	0/0	.	.	0/0	1/1	1/1	0/0	.	.	0/0	0/0	0/0	0/0	1/1	0/0	1/1	1/1	1/1	.	0/0	0/0	0/0	0/0	0/0	0/0	.	0/0	1/1	0/0	0/0	.	0/0	0/0	0/0	1/1	0/0	0/0	0/0	.	1/1	0/0	.	0/0	0/0	0/0	1/1	.	1/1	0/0	1/1	1/1	0/0	0/0	0/0	0/0	.	.	1/1	0/0	0/0	0/0	0/0	0/0	1/1	.	.	0/0	0/0	.	1/1	0/0	1/1	0/0	0/0	0/0	0/0	0/0	0/0	0/0	.	.	0/0	0/0	1/1	1/1	0/0	0/0	0/0	0/0	0/0	0/0	0/0	.	0/0	0/0	0/0	0/0	0/0	.	0/0	0/0	0/0	1/1	0/0	1/1	1/1	1/1	.	0/0	0/0	1/1	.	1/1	0/0	0/0	1/1	1/1	1/1	1/1	1/1	0/0	0/0	.	0/0	0/0	.
3R	25607750	3R_25607750_SNP	C	A	.	.	T5EFF=-0.2773;T5VAR=0.0968534791163442;T5VARACC=0.0537016068252654;T5PVAL=4.727e-05;T5MIXED=9.5091142134028e-06;T6EFF=-0.2701;T6VAR=0.0267258260490386;T6VARACC=0.00185363579511322;T6PVAL=0.03589;T6MIXED=0.0262823987494066;AVGEFF=-0.2737;AVGVAR=0.0553104188919815;AVGVARACC=0.0148449760711148;AVGPVAL=0.00236;AVGMIXED=0.00103395804672806;DIFFEFF=-0.007191;DIFFVAR=3.17331757359373e-05;DIFFVARACC=0.00157125529434726;DIFFPVAL=0.9428;DIFFMIXED=0.950802718157887;ANNOT=(FBgn0001297|kay|INTRON)	GT	0/0	0/0	0/0	0/0	.	0/0	0/0	1/1	0/0	1/1	0/0	0/0	0/0	0/0	0/0	0/0	0/0	0/0	0/0	0/0	0/0	0/0	0/0	0/0	0/0	1/1	0/0	0/0	0/0	0/0	0/0	0/0	0/0	0/0	0/0	0/0	0/0	1/1	.	0/0	0/0	0/0	0/0	0/0	.	0/0	0/0	0/0	0/0	0/0	0/0	0/0	0/0	0/0	0/0	0/0	0/0	.	0/0	0/0	.	0/0	0/0	0/0	0/0	0/0	0/0	0/0	.	0/0	0/0	0/0	0/0	0/0	0/0	0/0	1/1	0/0	0/0	0/0	0/0	0/0	0/0	0/0	.	0/0	0/0	0/0	0/0	0/0	0/0	0/0	0/0	1/1	0/0	0/0	0/0	0/0	1/1	0/0	0/0	.	0/0	0/0	0/0	0/0	0/0	0/0	0/0	0/0	0/0	0/0	0/0	0/0	0/0	0/0	0/0	0/0	0/0	0/0	0/0	0/0	0/0	1/1	0/0	0/0	0/0	0/0	0/0	1/1	0/0	0/0	0/0	0/0	0/0	0/0	0/0	1/1	0/0	0/0	0/0	0/0	0/0	0/0	0/0	0/0	0/0	1/1	0/0	0/0	1/1	0/0	0/0	0/0	0/0	0/0	0/0	0/0	.	0/0	1/1	0/0	0/0	0/0	0/0	0/0	0/0	.	0/0	0/0	0/0	0/0	0/0	0/0	1/1
3R	25877754	3R_25877754_SNP	T	C	.	.	T5EFF=3.761e-05;T5VAR=5.5249884345933e-09;T5VARACC=4.89355673812808e-05;T5PVAL=0.9993;T5MIXED=0.961154085139352;T6EFF=-0.2466;T6VAR=0.0710657955919956;T6VARACC=0.0253682349300531;T6PVAL=0.0006557;T6MIXED=0.000609318818401991;AVGEFF=-0.1233;AVGVAR=0.0355686655081082;AVGVARACC=0.0213485634259868;AVGPVAL=0.01692;AVGMIXED=0.0144467311556725;DIFFEFF=0.2466;DIFFVAR=0.118301591867206;DIFFVARACC=0.0663520127127634;DIFFPVAL=8.461e-06;DIFFMIXED=7.60601034042712e-06;ANNOT=(FBgn0264838|CR44046|EXON),(FBgn0039742|CG15528|UTR_5_PRIME),(FBgn0039743|CG7946|482 bp UPSTREAM)	GT	0/0	1/1	1/1	0/0	.	1/1	0/0	1/1	1/1	0/0	0/0	1/1	1/1	0/0	0/0	0/0	0/0	0/0	1/1	1/1	1/1	1/1	1/1	0/0	0/0	0/0	0/0	0/0	1/1	0/0	1/1	0/0	1/1	1/1	1/1	1/1	0/0	0/0	1/1	1/1	.	0/0	1/1	0/0	0/0	0/0	1/1	0/0	0/0	0/0	0/0	0/0	1/1	0/0	0/0	1/1	0/0	1/1	.	0/0	0/0	0/0	1/1	.	0/0	0/0	.	1/1	.	1/1	0/0	1/1	1/1	0/0	0/0	0/0	0/0	0/0	0/0	1/1	.	1/1	0/0	0/0	1/1	0/0	1/1	1/1	.	1/1	0/0	1/1	0/0	0/0	0/0	1/1	0/0	0/0	0/0	0/0	0/0	0/0	0/0	0/0	0/0	1/1	0/0	1/1	0/0	0/0	0/0	0/0	0/0	0/0	1/1	0/0	0/0	1/1	1/1	.	1/1	.	0/0	1/1	1/1	1/1	0/0	0/0	0/0	1/1	0/0	.	1/1	1/1	0/0	0/0	0/0	1/1	0/0	0/0	0/0	1/1	1/1	0/0	0/0	0/0	0/0	1/1	0/0	0/0	0/0	1/1	1/1	0/0	0/0	0/0	.	1/1	1/1	0/0	0/0	1/1	1/1	1/1	1/1	1/1	1/1	0/0	0/0	.	0/0	1/1	.	.	1/1
3R	26056155	3R_26056155_SNP	A	T	.	.	T5EFF=-0.2123;T5VAR=0.121319069737962;T5VARACC=0.0962174832527024;T5PVAL=4.254e-06;T5MIXED=1.28328977997117e-05;T6EFF=-0.2409;T6VAR=0.0434512786704138;T6VARACC=0.0204576545882034;T6PVAL=0.007038;T6MIXED=0.00829704294978281;AVGEFF=-0.2266;AVGVAR=0.0789802268112596;AVGVARACC=0.0513762266065531;AVGPVAL=0.0002447;AVGMIXED=0.000453101909801971;DIFFEFF=0.02859;DIFFVAR=0.00100415150465246;DIFFVARACC=0.0025222084058254;DIFFPVAL=0.6853;DIFFMIXED=0.689715035992157;ANNOT=(FBgn0000279|CecC|13495 bp DOWNSTREAM),(FBgn0263402|CG43448|6661 bp UPSTREAM)	GT	1/1	1/1	1/1	0/0	.	1/1	1/1	0/0	.	0/0	0/0	1/1	0/0	1/1	0/0	.	1/1	1/1	1/1	1/1	1/1	1/1	1/1	1/1	0/0	0/0	0/0	1/1	1/1	1/1	1/1	1/1	1/1	1/1	1/1	1/1	1/1	1/1	1/1	0/0	1/1	1/1	0/0	1/1	0/0	1/1	0/0	1/1	1/1	0/0	1/1	1/1	1/1	1/1	1/1	1/1	1/1	.	1/1	1/1	1/1	1/1	1/1	.	1/1	0/0	1/1	1/1	.	1/1	1/1	1/1	1/1	1/1	1/1	1/1	1/1	1/1	.	1/1	1/1	0/0	1/1	1/1	.	1/1	1/1	1/1	0/0	1/1	1/1	1/1	1/1	1/1	0/0	0/0	1/1	1/1	0/0	1/1	1/1	1/1	1/1	1/1	1/1	1/1	.	1/1	1/1	1/1	1/1	1/1	1/1	0/0	0/0	1/1	1/1	1/1	1/1	1/1	1/1	1/1	1/1	1/1	1/1	1/1	1/1	1/1	1/1	1/1	1/1	1/1	0/0	0/0	1/1	1/1	1/1	0/0	0/0	0/0	1/1	1/1	1/1	1/1	1/1	1/1	1/1	0/0	1/1	0/0	1/1	1/1	1/1	1/1	1/1	1/1	1/1	1/1	1/1	1/1	1/1	1/1	1/1	1/1	0/0	1/1	1/1	0/0	1/1	1/1	1/1	0/0	1/1	1/1	1/1
3R	26062475	3R_26062475_SNP	A	C	.	.	T5EFF=-0.1852;T5VAR=0.111410208105355;T5VARACC=0.0584050615771599;T5PVAL=1.109e-05;T5MIXED=6.74883069440281e-06;T6EFF=-0.1826;T6VAR=0.0313424749487552;T6VARACC=0.0307413368544667;T6PVAL=0.0225;T6MIXED=0.0265265245421089;AVGEFF=-0.1839;AVGVAR=0.0642387562466675;AVGVARACC=0.0495052033957769;AVGPVAL=0.0009849;AVGMIXED=0.00144457782981603;DIFFEFF=-0.002609;DIFFVAR=1.0683170884463e-05;DIFFVARACC=0.00109966374946385;DIFFPVAL=0.9667;DIFFMIXED=0.938299125607176;ANNOT=(FBgn0263402|CG43448|341 bp DOWNSTREAM)	GT	1/1	1/1	1/1	1/1	.	1/1	0/0	1/1	1/1	0/0	0/0	1/1	1/1	0/0	0/0	0/0	1/1	1/1	1/1	1/1	0/0	1/1	1/1	1/1	0/0	0/0	0/0	0/0	1/1	1/1	1/1	1/1	1/1	1/1	1/1	1/1	.	1/1	1/1	0/0	1/1	1/1	1/1	1/1	1/1	1/1	0/0	1/1	1/1	1/1	1/1	1/1	1/1	.	.	1/1	1/1	.	1/1	0/0	1/1	1/1	0/0	0/0	1/1	0/0	1/1	0/0	0/0	1/1	1/1	0/0	1/1	1/1	1/1	0/0	1/1	1/1	.	1/1	1/1	1/1	1/1	1/1	.	0/0	1/1	1/1	1/1	1/1	0/0	1/1	1/1	1/1	1/1	0/0	1/1	1/1	0/0	1/1	1/1	0/0	1/1	1/1	1/1	0/0	0/0	0/0	0/0	1/1	1/1	1/1	1/1	0/0	0/0	1/1	1/1	1/1	1/1	0/0	1/1	.	1/1	1/1	0/0	1/1	1/1	1/1	0/0	1/1	1/1	1/1	0/0	1/1	1/1	1/1	1/1	0/0	1/1	1/1	1/1	0/0	1/1	1/1	1/1	1/1	0/0	0/0	0/0	0/0	1/1	1/1	0/0	1/1	1/1	1/1	1/1	1/1	1/1	1/1	1/1	1/1	1/1	1/1	0/0	1/1	1/1	1/1	1/1	.	1/1	0/0	1/1	0/0	1/1
3R	26553128	3R_26553128_SNP	C	G	.	.	T5EFF=-0.3331;T5VAR=0.121911833671174;T5VARACC=0.0760873722749409;T5PVAL=3.507e-06;T5MIXED=2.64171102905236e-07;T6EFF=-0.4278;T6VAR=0.0583733570188994;T6VARACC=0.0339805723263799;T6PVAL=0.001605;T6MIXED=0.00120551359311428;AVGEFF=-0.3804;AVGVAR=0.0933979669863466;AVGVARACC=0.076973150133074;AVGPVAL=5.616e-05;AVGMIXED=1.48984482850766e-05;DIFFEFF=0.0947;DIFFVAR=0.00474107542135579;DIFFVARACC=0.00327074329881877;DIFFPVAL=0.3752;DIFFMIXED=0.368902984246981;ANNOT=(FBgn0039797|CG1340|NON_SYNONYMOUS_CODING)	GT	0/0	0/0	0/0	0/0	0/0	1/1	0/0	0/0	0/0	1/1	0/0	0/0	0/0	0/0	0/0	0/0	0/0	0/0	0/0	1/1	0/0	0/0	0/0	0/0	0/0	1/1	0/0	0/0	0/0	0/0	0/0	0/0	0/0	0/0	0/0	0/0	0/0	0/0	0/0	0/0	0/0	0/0	0/0	0/0	0/0	0/0	0/0	0/0	0/0	0/0	0/0	0/0	0/0	0/0	0/0	0/0	0/0	0/0	.	0/0	0/0	0/0	0/0	.	0/0	0/0	0/0	0/0	.	0/0	0/0	0/0	0/0	0/0	0/0	0/0	0/0	0/0	0/0	0/0	0/0	0/0	0/0	0/0	0/0	0/0	0/0	0/0	0/0	.	.	0/0	0/0	0/0	0/0	0/0	0/0	0/0	0/0	0/0	0/0	0/0	0/0	0/0	0/0	0/0	0/0	1/1	0/0	0/0	0/0	0/0	0/0	0/0	1/1	0/0	0/0	0/0	0/0	.	0/0	0/0	0/0	0/0	.	0/0	0/0	0/0	0/0	0/0	0/0	0/0	1/1	0/0	0/0	0/0	0/0	0/0	0/0	0/0	0/0	1/1	0/0	0/0	0/0	0/0	0/0	1/1	0/0	0/0	0/0	0/0	0/0	0/0	0/0	0/0	0/0	0/0	0/0	0/0	0/0	0/0	0/0	0/0	1/1	0/0	0/0	0/0	0/0	0/0	0/0	0/0	0/0	1/1	1/1
3R	26781069	3R_26781069_SNP	G	T	.	.	T5EFF=-0.2714;T5VAR=0.117351247320729;T5VARACC=0.0665597319203377;T5PVAL=4.815e-06;T5MIXED=3.42224967524849e-05;T6EFF=-0.322;T6VAR=0.0466752527892592;T6VARACC=0.0264201662446025;T6PVAL=0.004661;T6MIXED=0.00697638227478758;AVGEFF=-0.2967;AVGVAR=0.0807872632014972;AVGVARACC=0.0473747046197592;AVGPVAL=0.0001724;AVGMIXED=0.000720000659819972;DIFFEFF=0.05058;DIFFVAR=0.00190986134289997;DIFFVARACC=0.000159084516128116;DIFFPVAL=0.5715;DIFFMIXED=0.583772274187891;ANNOT=(FBgn0039811|CG15550|18245 bp UPSTREAM),(FBgn0039812|CG15548|4353 bp DOWNSTREAM)	GT	0/0	1/1	0/0	0/0	0/0	0/0	0/0	0/0	0/0	1/1	0/0	0/0	0/0	0/0	1/1	0/0	1/1	0/0	1/1	0/0	0/0	0/0	0/0	0/0	0/0	.	0/0	0/0	0/0	0/0	0/0	0/0	0/0	0/0	0/0	0/0	0/0	1/1	0/0	1/1	0/0	0/0	0/0	0/0	0/0	0/0	0/0	0/0	0/0	0/0	0/0	0/0	0/0	0/0	0/0	0/0	0/0	0/0	0/0	0/0	0/0	0/0	1/1	.	0/0	0/0	0/0	0/0	0/0	0/0	0/0	0/0	0/0	0/0	0/0	0/0	0/0	0/0	0/0	0/0	0/0	0/0	0/0	0/0	.	0/0	0/0	1/1	0/0	0/0	0/0	0/0	0/0	0/0	0/0	0/0	0/0	0/0	1/1	0/0	1/1	1/1	0/0	0/0	0/0	0/0	1/1	0/0	0/0	0/0	0/0	0/0	0/0	0/0	0/0	0/0	0/0	0/0	0/0	0/0	0/0	.	0/0	1/1	0/0	1/1	0/0	0/0	0/0	0/0	0/0	0/0	0/0	0/0	0/0	0/0	0/0	1/1	0/0	0/0	0/0	0/0	0/0	0/0	0/0	0/0	0/0	1/1	0/0	0/0	0/0	0/0	0/0	0/0	0/0	0/0	0/0	0/0	0/0	0/0	0/0	0/0	0/0	0/0	0/0	1/1	0/0	0/0	0/0	0/0	0/0	0/0	0/0	.	0/0
3R	26781081	3R_26781081_SNP	C	T	.	.	T5EFF=-0.2891;T5VAR=0.122848391049579;T5VARACC=0.0921943974091922;T5PVAL=2.79e-06;T5MIXED=6.74107149307255e-06;T6EFF=-0.354;T6VAR=0.0510813851464675;T6VARACC=0.0418626988188155;T6PVAL=0.003041;T6MIXED=0.0036045427336268;AVGEFF=-0.3215;AVGVAR=0.0865351086091459;AVGVARACC=0.0609724564665398;AVGPVAL=9.873e-05;AVGMIXED=0.000188727807830861;DIFFEFF=0.06491;DIFFVAR=0.00283154699729347;DIFFVARACC=0.00286749994388596;DIFFPVAL=0.4907;DIFFMIXED=0.492474811290379;ANNOT=(FBgn0039811|CG15550|18257 bp UPSTREAM),(FBgn0039812|CG15548|4341 bp DOWNSTREAM)	GT	0/0	1/1	0/0	0/0	0/0	0/0	0/0	0/0	0/0	1/1	0/0	0/0	0/0	0/0	1/1	0/0	1/1	0/0	1/1	0/0	0/0	0/0	0/0	0/0	0/0	1/1	0/0	0/0	0/0	0/0	0/0	0/0	0/0	0/0	0/0	0/0	0/0	0/0	0/0	1/1	0/0	0/0	0/0	0/0	0/0	0/0	0/0	0/0	0/0	0/0	0/0	0/0	0/0	0/0	0/0	0/0	0/0	0/0	0/0	0/0	0/0	0/0	1/1	.	0/0	0/0	0/0	0/0	0/0	0/0	0/0	0/0	0/0	0/0	0/0	0/0	0/0	0/0	0/0	0/0	0/0	0/0	0/0	0/0	.	0/0	0/0	1/1	0/0	0/0	0/0	0/0	0/0	0/0	0/0	0/0	0/0	0/0	1/1	0/0	0/0	1/1	0/0	0/0	0/0	0/0	.	0/0	0/0	0/0	0/0	0/0	0/0	0/0	0/0	0/0	0/0	0/0	0/0	0/0	0/0	.	0/0	1/1	0/0	1/1	0/0	0/0	0/0	0/0	0/0	0/0	0/0	0/0	0/0	0/0	0/0	1/1	0/0	0/0	0/0	0/0	0/0	0/0	0/0	0/0	0/0	1/1	0/0	0/0	0/0	0/0	0/0	0/0	0/0	0/0	0/0	0/0	0/0	0/0	0/0	0/0	0/0	0/0	0/0	1/1	0/0	0/0	0/0	0/0	0/0	0/0	0/0	.	0/0
3R	26781114	3R_26781114_SNP	T	G	.	.	T5EFF=-0.3112;T5VAR=0.144460976641253;T5VARACC=0.0996600577812436;T5PVAL=2.936e-07;T5MIXED=5.33473231837886e-07;T6EFF=-0.3738;T6VAR=0.059437530918468;T6VARACC=0.0438856533236414;T6PVAL=0.001312;T6MIXED=0.00154794421917065;AVGEFF=-0.3425;AVGVAR=0.101270623107726;AVGVARACC=0.0692916198404372;AVGPVAL=2.219e-05;AVGMIXED=4.10163359213747e-05;DIFFEFF=0.06255;DIFFVAR=0.00277808241455703;DIFFVARACC=0.00248053859905756;DIFFPVAL=0.4936;DIFFMIXED=0.497873398121406;ANNOT=(FBgn0039811|CG15550|18290 bp UPSTREAM),(FBgn0039812|CG15548|4308 bp DOWNSTREAM)	GT	0/0	1/1	0/0	0/0	0/0	0/0	0/0	0/0	0/0	1/1	0/0	0/0	0/0	0/0	1/1	0/0	1/1	0/0	1/1	0/0	0/0	0/0	0/0	0/0	0/0	1/1	0/0	0/0	0/0	0/0	0/0	0/0	0/0	0/0	0/0	0/0	0/0	0/0	0/0	1/1	0/0	0/0	0/0	0/0	0/0	0/0	0/0	0/0	0/0	0/0	0/0	0/0	0/0	0/0	0/0	0/0	0/0	0/0	0/0	0/0	0/0	0/0	1/1	.	0/0	0/0	0/0	0/0	0/0	0/0	0/0	0/0	0/0	0/0	0/0	0/0	0/0	0/0	0/0	0/0	0/0	0/0	0/0	0/0	.	0/0	0/0	1/1	0/0	0/0	0/0	0/0	0/0	0/0	0/0	0/0	0/0	0/0	1/1	0/0	0/0	1/1	0/0	0/0	0/0	0/0	1/1	0/0	0/0	0/0	0/0	0/0	0/0	0/0	0/0	0/0	0/0	0/0	0/0	0/0	0/0	.	0/0	1/1	0/0	1/1	0/0	0/0	0/0	0/0	0/0	0/0	0/0	0/0	0/0	0/0	0/0	1/1	0/0	0/0	0/0	0/0	0/0	0/0	0/0	0/0	0/0	1/1	0/0	0/0	0/0	0/0	0/0	0/0	0/0	0/0	0/0	0/0	0/0	0/0	0/0	0/0	0/0	0/0	0/0	1/1	0/0	0/0	0/0	0/0	0/0	0/0	0/0	.	0/0
3R	26781263	3R_26781263_SNP	A	G	.	.	T5EFF=-0.286;T5VAR=0.1277036062031;T5VARACC=0.0887072546700013;T5PVAL=1.719e-06;T5MIXED=4.29855585678555e-06;T6EFF=-0.3623;T6VAR=0.0584440872876514;T6VARACC=0.0506420171093867;T6PVAL=0.001493;T6MIXED=0.00164217623978805;AVGEFF=-0.3242;AVGVAR=0.0948390686077233;AVGVARACC=0.0709448390437988;AVGPVAL=4.4e-05;AVGMIXED=8.01999869782086e-05;DIFFEFF=0.07633;DIFFVAR=0.00434285041491123;DIFFVARACC=0.00608936645482155;DIFFPVAL=0.3932;DIFFMIXED=0.39172503489134;ANNOT=(FBgn0039811|CG15550|18439 bp UPSTREAM),(FBgn0039812|CG15548|4159 bp DOWNSTREAM)	GT	0/0	1/1	0/0	0/0	0/0	0/0	0/0	0/0	0/0	1/1	0/0	0/0	0/0	0/0	1/1	0/0	.	0/0	1/1	0/0	0/0	0/0	1/1	0/0	0/0	1/1	0/0	0/0	0/0	0/0	0/0	0/0	0/0	0/0	0/0	0/0	.	0/0	0/0	1/1	0/0	0/0	0/0	0/0	0/0	0/0	0/0	0/0	0/0	0/0	0/0	0/0	0/0	1/1	0/0	0/0	0/0	0/0	0/0	0/0	0/0	0/0	1/1	.	0/0	0/0	0/0	0/0	0/0	0/0	0/0	0/0	0/0	0/0	0/0	0/0	0/0	0/0	0/0	0/0	0/0	0/0	0/0	0/0	.	0/0	0/0	1/1	0/0	0/0	0/0	0/0	0/0	0/0	0/0	0/0	0/0	0/0	1/1	0/0	0/0	1/1	0/0	0/0	0/0	0/0	1/1	0/0	0/0	0/0	0/0	0/0	0/0	0/0	0/0	0/0	0/0	0/0	0/0	0/0	0/0	.	0/0	1/1	0/0	1/1	0/0	0/0	0/0	0/0	0/0	0/0	0/0	0/0	0/0	0/0	0/0	1/1	0/0	0/0	0/0	0/0	0/0	0/0	0/0	0/0	0/0	1/1	0/0	0/0	0/0	0/0	0/0	0/0	0/0	0/0	0/0	0/0	0/0	0/0	0/0	0/0	0/0	0/0	0/0	1/1	0/0	0/0	0/0	0/0	0/0	0/0	0/0	0/0	0/0
3R	26782268	3R_26782268_SNP	A	T	.	.	T5EFF=-0.275;T5VAR=0.133061536379261;T5VARACC=0.0909709405765628;T5PVAL=1.256e-06;T5MIXED=5.72198007467448e-06;T6EFF=-0.3451;T6VAR=0.0567437795636875;T6VARACC=0.0305193273982894;T6PVAL=0.001935;T6MIXED=0.00237745695722757;AVGEFF=-0.31;AVGVAR=0.0946425784573709;AVGVARACC=0.0508031932768225;AVGPVAL=5.249e-05;AVGMIXED=0.000138003390782229;DIFFEFF=0.07012;DIFFVAR=0.00386774091292121;DIFFVARACC=0.00692956412761314;DIFFPVAL=0.4246;DIFFMIXED=0.428222554938785;ANNOT=(FBgn0039811|CG15550|19444 bp UPSTREAM),(FBgn0039812|CG15548|3154 bp DOWNSTREAM)	GT	0/0	1/1	0/0	0/0	.	0/0	0/0	0/0	0/0	.	0/0	0/0	0/0	0/0	1/1	1/1	1/1	0/0	1/1	0/0	0/0	0/0	0/0	0/0	0/0	1/1	0/0	0/0	0/0	0/0	0/0	0/0	0/0	0/0	0/0	0/0	0/0	0/0	0/0	1/1	.	0/0	0/0	0/0	0/0	0/0	0/0	0/0	0/0	0/0	0/0	0/0	0/0	0/0	0/0	0/0	0/0	0/0	0/0	0/0	0/0	0/0	1/1	.	0/0	0/0	0/0	0/0	0/0	0/0	0/0	0/0	0/0	0/0	0/0	0/0	0/0	0/0	0/0	0/0	0/0	.	0/0	0/0	.	0/0	0/0	1/1	0/0	0/0	0/0	0/0	0/0	0/0	1/1	0/0	0/0	0/0	1/1	0/0	0/0	.	0/0	0/0	0/0	0/0	1/1	0/0	1/1	0/0	0/0	0/0	0/0	0/0	0/0	0/0	0/0	0/0	0/0	0/0	0/0	.	0/0	1/1	0/0	1/1	0/0	0/0	1/1	0/0	0/0	0/0	0/0	0/0	0/0	0/0	0/0	1/1	0/0	0/0	0/0	0/0	0/0	0/0	0/0	0/0	0/0	1/1	0/0	0/0	0/0	0/0	0/0	0/0	0/0	0/0	0/0	0/0	0/0	0/0	0/0	0/0	0/0	0/0	0/0	1/1	0/0	0/0	0/0	0/0	0/0	0/0	0/0	0/0	0/0
3R	26783503	3R_26783503_SNP	C	T	.	.	T5EFF=-0.292;T5VAR=0.112980981161079;T5VARACC=0.0773114114536462;T5PVAL=6.545e-06;T5MIXED=1.70233150631868e-05;T6EFF=-0.3503;T6VAR=0.0463382807346388;T6VARACC=0.035479645696188;T6PVAL=0.00457;T6MIXED=0.00575791193564423;AVGEFF=-0.3211;AVGVAR=0.0789630378005949;AVGVARACC=0.046979403509923;AVGPVAL=0.0001885;AVGMIXED=0.000412333636568019;DIFFEFF=0.05832;DIFFVAR=0.0021513404071558;DIFFVARACC=0.00650231431501791;DIFFPVAL=0.5457;DIFFMIXED=0.553741644901475;ANNOT=(FBgn0039811|CG15550|20679 bp UPSTREAM),(FBgn0039812|CG15548|1919 bp DOWNSTREAM)	GT	0/0	0/0	0/0	0/0	0/0	0/0	0/0	0/0	0/0	1/1	0/0	0/0	0/0	0/0	1/1	0/0	.	0/0	1/1	0/0	0/0	0/0	0/0	0/0	0/0	1/1	0/0	0/0	0/0	0/0	0/0	0/0	0/0	0/0	0/0	0/0	0/0	0/0	0/0	1/1	0/0	0/0	0/0	0/0	0/0	0/0	0/0	0/0	0/0	0/0	0/0	0/0	0/0	0/0	0/0	0/0	0/0	0/0	0/0	0/0	0/0	0/0	1/1	.	0/0	0/0	0/0	0/0	0/0	0/0	0/0	0/0	0/0	0/0	0/0	0/0	0/0	0/0	0/0	0/0	0/0	0/0	0/0	0/0	0/0	0/0	0/0	1/1	0/0	0/0	0/0	0/0	0/0	0/0	1/1	0/0	0/0	0/0	1/1	0/0	0/0	1/1	0/0	0/0	0/0	0/0	1/1	0/0	0/0	0/0	0/0	0/0	0/0	0/0	0/0	0/0	0/0	0/0	0/0	0/0	0/0	.	0/0	1/1	0/0	1/1	0/0	0/0	0/0	0/0	0/0	0/0	0/0	0/0	0/0	0/0	0/0	1/1	0/0	0/0	0/0	0/0	0/0	0/0	0/0	0/0	0/0	0/0	0/0	0/0	0/0	0/0	0/0	0/0	0/0	0/0	0/0	0/0	0/0	0/0	0/0	0/0	0/0	0/0	0/0	1/1	0/0	0/0	0/0	0/0	0/0	0/0	0/0	0/0	0/0
3R	9719899	3R_9719899_SNP	A	T	.	.	T5EFF=-0.3894;T5VAR=0.125280002749636;T5VARACC=0.0767295855844131;T5PVAL=1.905e-06;T5MIXED=1.04598446287883e-05;T6EFF=-0.405;T6VAR=0.039374073148043;T6VARACC=0.0103633609504385;T6PVAL=0.00907;T6MIXED=0.0113346692935585;AVGEFF=-0.3972;AVGVAR=0.0764075374795915;AVGVARACC=0.0379252740398215;AVGPVAL=0.000242;AVGMIXED=0.000577803497395078;DIFFEFF=0.01559;DIFFVAR=9.75022315224088e-05;DIFFVARACC=0.00140833018775516;DIFFPVAL=0.8977;DIFFMIXED=0.899493240068729;ANNOT=(FBgn0008646|E5|19261 bp UPSTREAM),(FBgn0000576|ems|7682 bp DOWNSTREAM)	GT	0/0	0/0	0/0	0/0	0/0	1/1	0/0	0/0	0/0	0/0	0/0	0/0	0/0	0/0	1/1	1/1	0/0	1/1	0/0	0/0	0/0	0/0	0/0	0/0	0/0	0/0	0/0	0/0	0/0	0/0	0/0	0/0	0/0	0/0	0/0	0/0	0/0	0/0	0/0	0/0	.	0/0	0/0	0/0	0/0	0/0	0/0	0/0	0/0	0/0	0/0	0/0	0/0	0/0	0/0	0/0	0/0	0/0	0/0	0/0	0/0	0/0	0/0	0/0	0/0	0/0	0/0	0/0	.	0/0	0/0	0/0	0/0	0/0	0/0	0/0	0/0	0/0	0/0	0/0	0/0	0/0	0/0	0/0	0/0	0/0	0/0	0/0	0/0	0/0	0/0	0/0	0/0	0/0	1/1	0/0	0/0	0/0	1/1	0/0	0/0	0/0	0/0	0/0	0/0	1/1	0/0	0/0	0/0	0/0	0/0	0/0	0/0	0/0	0/0	0/0	0/0	0/0	0/0	0/0	0/0	0/0	0/0	0/0	0/0	0/0	0/0	0/0	0/0	0/0	0/0	0/0	0/0	0/0	0/0	0/0	0/0	0/0	0/0	.	0/0	0/0	0/0	0/0	0/0	0/0	0/0	1/1	0/0	0/0	0/0	0/0	0/0	0/0	0/0	0/0	0/0	0/0	0/0	0/0	0/0	0/0	0/0	0/0	0/0	1/1	0/0	0/0	0/0	0/0	0/0	0/0	0/0	0/0	0/0
X	12958087	X_12958087_SNP	C	G	.	.	T5EFF=0.04502;T5VAR=0.00856951504409029;T5VARACC=0.000132676073139715;T5PVAL=0.2355;T5MIXED=0.211448081368706;T6EFF=0.2885;T6VAR=0.0975040560785176;T6VARACC=0.0332267952435825;T6PVAL=4.21e-05;T6MIXED=4.03325366718264e-05;AVGEFF=0.1667;AVGVAR=0.0663384536930032;AVGVARACC=0.0225590161466162;AVGPVAL=0.0008079;AVGMIXED=0.000883374276947207;DIFFEFF=-0.2434;DIFFVAR=0.117713116995778;DIFFVARACC=0.0536673465813131;DIFFPVAL=6.035e-06;DIFFMIXED=5.79191640621776e-06;ANNOT=(FBgn0261388|CG42629|INTRON)	GT	1/1	.	0/0	1/1	0/0	1/1	0/0	1/1	1/1	0/0	.	.	1/1	0/0	0/0	0/0	1/1	0/0	0/0	1/1	0/0	1/1	1/1	1/1	0/0	1/1	0/0	1/1	1/1	1/1	1/1	1/1	1/1	1/1	1/1	1/1	1/1	0/0	0/0	0/0	0/0	0/0	0/0	0/0	1/1	0/0	1/1	1/1	0/0	0/0	0/0	0/0	1/1	1/1	0/0	1/1	1/1	1/1	0/0	0/0	0/0	0/0	1/1	0/0	1/1	0/0	0/0	0/0	1/1	1/1	0/0	1/1	0/0	0/0	0/0	0/0	0/0	0/0	0/0	1/1	1/1	1/1	0/0	0/0	0/0	1/1	0/0	1/1	1/1	0/0	1/1	0/0	1/1	1/1	0/0	0/0	1/1	0/0	0/0	.	1/1	1/1	.	1/1	.	.	1/1	1/1	0/0	1/1	0/0	1/1	0/0	1/1	1/1	0/0	1/1	.	1/1	1/1	1/1	0/0	1/1	0/0	0/0	1/1	0/0	0/0	1/1	1/1	1/1	1/1	0/0	0/0	1/1	0/0	0/0	1/1	1/1	0/0	0/0	1/1	0/0	0/0	0/0	0/0	1/1	1/1	1/1	1/1	1/1	1/1	1/1	1/1	1/1	0/0	0/0	1/1	0/0	0/0	1/1	1/1	1/1	1/1	1/1	0/0	0/0	1/1	0/0	0/0	0/0	.	0/0	0/0	0/0
X	3205237	X_3205237_SNP	A	G	.	.	T5EFF=-0.3295;T5VAR=0.107561825825481;T5VARACC=0.0614628518513227;T5PVAL=1.058e-05;T5MIXED=7.08651543390643e-06;T6EFF=-0.2843;T6VAR=0.0227550193166732;T6VARACC=0.0115969532676345;T6PVAL=0.04758;T6MIXED=0.0416911688254768;AVGEFF=-0.3069;AVGVAR=0.0535017133303595;AVGVARACC=0.0264831166020991;AVGPVAL=0.002199;AVGMIXED=0.00163287297589289;DIFFEFF=-0.04524;DIFFVAR=0.000983981177703051;DIFFVARACC=0.00142357316903208;DIFFPVAL=0.682;DIFFMIXED=0.69197058041132;ANNOT=(FBgn0000479|dnc|INTRON)	GT	0/0	0/0	0/0	1/1	0/0	0/0	0/0	0/0	0/0	1/1	0/0	0/0	0/0	0/0	0/0	1/1	0/0	0/0	0/0	0/0	0/0	0/0	0/0	0/0	0/0	0/0	0/0	0/0	0/0	0/0	0/0	0/0	0/0	0/0	0/0	0/0	0/0	0/0	0/0	0/0	0/0	0/0	0/0	0/0	0/0	0/0	0/0	0/0	0/0	0/0	0/0	0/0	0/0	0/0	0/0	0/0	0/0	0/0	0/0	0/0	0/0	0/0	0/0	0/0	0/0	0/0	0/0	0/0	0/0	0/0	0/0	0/0	0/0	0/0	0/0	0/0	0/0	0/0	0/0	0/0	.	0/0	0/0	0/0	0/0	0/0	0/0	0/0	0/0	0/0	0/0	0/0	0/0	0/0	0/0	0/0	0/0	0/0	0/0	0/0	0/0	0/0	0/0	0/0	0/0	0/0	0/0	0/0	0/0	0/0	0/0	0/0	1/1	0/0	0/0	0/0	1/1	0/0	0/0	0/0	0/0	0/0	0/0	0/0	0/0	0/0	0/0	0/0	0/0	0/0	0/0	0/0	0/0	0/0	.	0/0	0/0	0/0	0/0	0/0	1/1	1/1	0/0	1/1	0/0	0/0	0/0	0/0	0/0	0/0	0/0	0/0	0/0	0/0	0/0	0/0	0/0	0/0	0/0	1/1	0/0	0/0	0/0	0/0	0/0	1/1	0/0	0/0	0/0	0/0	0/0	0/0	0/0	0/0	1/1
X	3254475	X_3254475_SNP	A	T	.	.	T5EFF=-0.3095;T5VAR=0.109839149082161;T5VARACC=0.0640982386527996;T5PVAL=7.46e-06;T5MIXED=0.00011451528637982;T6EFF=-0.347;T6VAR=0.0392423071948005;T6VARACC=0.0232503000977523;T6PVAL=0.008592;T6MIXED=0.0115073379256567;AVGEFF=-0.3282;AVGVAR=0.071082565696304;AVGVARACC=0.0558677681684299;AVGPVAL=0.0003618;AVGMIXED=0.00133137050350419;DIFFEFF=0.03755;DIFFVAR=0.000775633774784149;DIFFVARACC=0.000365167190808924;DIFFPVAL=0.7145;DIFFMIXED=0.713295934878498;ANNOT=(FBgn0000479|dnc|16675 bp DOWNSTREAM),(FBgn0262656|dm|12717 bp DOWNSTREAM)	GT	1/1	1/1	0/0	1/1	1/1	1/1	1/1	1/1	1/1	0/0	1/1	1/1	1/1	1/1	1/1	1/1	1/1	1/1	1/1	1/1	1/1	0/0	1/1	1/1	1/1	1/1	1/1	1/1	1/1	1/1	1/1	1/1	1/1	1/1	1/1	1/1	1/1	1/1	1/1	1/1	1/1	1/1	1/1	1/1	1/1	1/1	1/1	1/1	1/1	1/1	1/1	1/1	1/1	0/0	1/1	1/1	1/1	1/1	1/1	1/1	1/1	1/1	1/1	1/1	1/1	1/1	1/1	1/1	1/1	1/1	1/1	1/1	1/1	1/1	1/1	1/1	1/1	1/1	0/0	1/1	1/1	1/1	1/1	1/1	0/0	1/1	1/1	1/1	1/1	1/1	0/0	1/1	1/1	1/1	1/1	1/1	1/1	1/1	1/1	1/1	1/1	0/0	1/1	1/1	0/0	0/0	1/1	1/1	1/1	1/1	1/1	1/1	1/1	0/0	1/1	1/1	1/1	1/1	1/1	1/1	1/1	1/1	1/1	1/1	1/1	1/1	1/1	1/1	1/1	1/1	1/1	1/1	1/1	1/1	1/1	1/1	1/1	1/1	1/1	1/1	1/1	1/1	1/1	1/1	1/1	1/1	1/1	0/0	1/1	1/1	1/1	1/1	1/1	1/1	1/1	1/1	1/1	1/1	1/1	1/1	1/1	1/1	1/1	1/1	1/1	0/0	1/1	1/1	1/1	1/1	1/1	1/1	1/1	1/1	1/1
X	3254549	X_3254549_SNP	A	G	.	.	T5EFF=-0.3202;T5VAR=0.12649518970112;T5VARACC=0.0718410562078916;T5PVAL=1.685e-06;T5MIXED=1.08141570615876e-05;T6EFF=-0.3656;T6VAR=0.0468549032860775;T6VARACC=0.0171987766162277;T6PVAL=0.004344;T6MIXED=0.00546836793500757;AVGEFF=-0.3429;AVGVAR=0.083408144241099;AVGVARACC=0.0542915556278372;AVGPVAL=0.0001219;AVGMIXED=0.000336392614149095;DIFFEFF=0.04536;DIFFVAR=0.00121962097439915;DIFFVARACC=0.000878614711592174;DIFFPVAL=0.6492;DIFFMIXED=0.650132843977636;ANNOT=(FBgn0000479|dnc|16749 bp DOWNSTREAM),(FBgn0262656|dm|12643 bp DOWNSTREAM)	GT	1/1	1/1	0/0	1/1	1/1	1/1	1/1	1/1	1/1	0/0	1/1	1/1	1/1	1/1	1/1	1/1	0/0	1/1	1/1	1/1	1/1	0/0	1/1	1/1	1/1	1/1	1/1	1/1	1/1	1/1	0/0	1/1	1/1	1/1	1/1	1/1	1/1	1/1	1/1	1/1	1/1	1/1	1/1	1/1	1/1	1/1	1/1	1/1	1/1	1/1	1/1	1/1	1/1	0/0	1/1	1/1	1/1	1/1	1/1	1/1	1/1	1/1	1/1	1/1	1/1	1/1	1/1	1/1	1/1	1/1	1/1	1/1	1/1	1/1	1/1	1/1	1/1	1/1	.	1/1	1/1	1/1	1/1	1/1	0/0	1/1	1/1	1/1	1/1	1/1	0/0	1/1	1/1	1/1	1/1	1/1	1/1	1/1	1/1	1/1	1/1	0/0	1/1	1/1	0/0	0/0	1/1	1/1	1/1	1/1	1/1	1/1	1/1	0/0	.	1/1	1/1	1/1	1/1	1/1	1/1	1/1	1/1	1/1	1/1	1/1	1/1	1/1	1/1	.	1/1	1/1	1/1	1/1	1/1	1/1	1/1	1/1	1/1	1/1	1/1	1/1	1/1	1/1	1/1	1/1	1/1	0/0	1/1	1/1	1/1	1/1	1/1	1/1	1/1	1/1	1/1	1/1	1/1	1/1	1/1	1/1	1/1	1/1	1/1	0/0	1/1	1/1	1/1	1/1	1/1	1/1	1/1	1/1	1/1
X	3254648	X_3254648_SNP	A	T	.	.	T5EFF=-0.302;T5VAR=0.129304338262308;T5VARACC=0.07224001902254;T5PVAL=1.692e-06;T5MIXED=1.2448527679329e-05;T6EFF=-0.3366;T6VAR=0.0450029174749279;T6VARACC=0.0161213965380915;T6PVAL=0.00577;T6MIXED=0.00687314451914126;AVGEFF=-0.3193;AVGVAR=0.0820922975343185;AVGVARACC=0.047780715778511;AVGPVAL=0.0001664;AVGMIXED=0.000431786085939907;DIFFEFF=0.03453;DIFFVAR=0.000807112809854378;DIFFVARACC=0.000568121241775243;DIFFPVAL=0.7147;DIFFMIXED=0.716630992372218;ANNOT=(FBgn0000479|dnc|16848 bp DOWNSTREAM),(FBgn0262656|dm|12544 bp DOWNSTREAM)	GT	1/1	.	0/0	1/1	1/1	1/1	1/1	1/1	1/1	0/0	1/1	1/1	1/1	1/1	1/1	1/1	0/0	1/1	1/1	0/0	.	0/0	1/1	1/1	1/1	.	1/1	1/1	1/1	1/1	0/0	1/1	1/1	1/1	1/1	1/1	1/1	1/1	1/1	1/1	1/1	1/1	1/1	1/1	1/1	1/1	1/1	1/1	1/1	1/1	1/1	1/1	1/1	0/0	1/1	1/1	1/1	1/1	1/1	1/1	1/1	1/1	1/1	1/1	1/1	1/1	1/1	1/1	1/1	1/1	1/1	1/1	1/1	1/1	0/0	1/1	1/1	1/1	.	.	1/1	1/1	1/1	1/1	0/0	1/1	1/1	1/1	1/1	1/1	0/0	1/1	1/1	1/1	1/1	1/1	1/1	1/1	1/1	1/1	1/1	0/0	1/1	1/1	0/0	0/0	1/1	1/1	1/1	.	1/1	1/1	1/1	0/0	1/1	1/1	1/1	.	1/1	1/1	1/1	1/1	1/1	1/1	1/1	1/1	1/1	1/1	1/1	1/1	1/1	1/1	1/1	1/1	1/1	1/1	1/1	1/1	1/1	1/1	1/1	1/1	1/1	1/1	1/1	1/1	1/1	0/0	1/1	1/1	1/1	1/1	1/1	1/1	1/1	1/1	1/1	1/1	1/1	1/1	1/1	1/1	1/1	1/1	1/1	0/0	1/1	1/1	1/1	1/1	1/1	1/1	1/1	1/1	1/1
X	3254664	X_3254664_SNP	T	A	.	.	T5EFF=-0.2769;T5VAR=0.11267578591026;T5VARACC=0.0644246115241134;T5PVAL=6.747e-06;T5MIXED=9.19442017758281e-05;T6EFF=-0.3126;T6VAR=0.0406879902934169;T6VARACC=0.0154364589078163;T6PVAL=0.007967;T6MIXED=0.0107034309594345;AVGEFF=-0.2947;AVGVAR=0.0730567264392273;AVGVARACC=0.0435420366785528;AVGPVAL=0.0003358;AVGMIXED=0.0012193106225486;DIFFEFF=0.0357;DIFFVAR=0.00090529645520351;DIFFVARACC=0.000652982773280464;DIFFPVAL=0.6952;DIFFMIXED=0.699614894006318;ANNOT=(FBgn0000479|dnc|16864 bp DOWNSTREAM),(FBgn0262656|dm|12528 bp DOWNSTREAM)	GT	1/1	.	0/0	1/1	1/1	1/1	1/1	1/1	1/1	0/0	1/1	1/1	1/1	1/1	1/1	1/1	0/0	1/1	1/1	0/0	1/1	0/0	1/1	1/1	1/1	1/1	1/1	1/1	1/1	1/1	0/0	1/1	1/1	1/1	1/1	1/1	1/1	1/1	1/1	1/1	1/1	1/1	1/1	1/1	1/1	1/1	1/1	.	1/1	1/1	1/1	1/1	1/1	0/0	1/1	1/1	1/1	1/1	1/1	1/1	1/1	1/1	1/1	1/1	1/1	1/1	1/1	1/1	1/1	1/1	1/1	1/1	1/1	1/1	0/0	1/1	1/1	1/1	0/0	1/1	1/1	1/1	1/1	1/1	0/0	1/1	1/1	1/1	1/1	1/1	0/0	1/1	1/1	1/1	1/1	1/1	1/1	1/1	1/1	1/1	1/1	0/0	1/1	1/1	0/0	0/0	1/1	1/1	1/1	1/1	1/1	1/1	1/1	0/0	1/1	1/1	1/1	.	1/1	1/1	1/1	1/1	1/1	1/1	1/1	1/1	1/1	1/1	1/1	1/1	1/1	1/1	1/1	1/1	1/1	1/1	1/1	1/1	1/1	1/1	1/1	1/1	1/1	1/1	1/1	1/1	1/1	0/0	1/1	1/1	1/1	1/1	1/1	1/1	1/1	1/1	1/1	1/1	1/1	1/1	1/1	1/1	1/1	1/1	1/1	0/0	1/1	1/1	1/1	1/1	1/1	1/1	1/1	1/1	1/1
X	3254671	X_3254671_SNP	C	T	.	.	T5EFF=-0.297;T5VAR=0.123205794145361;T5VARACC=0.0700267040785368;T5PVAL=2.514e-06;T5MIXED=1.84460491122795e-05;T6EFF=-0.3296;T6VAR=0.0431804457411716;T6VARACC=0.0152020406458551;T6PVAL=0.006387;T6MIXED=0.00818608286044441;AVGEFF=-0.3133;AVGVAR=0.0787504260512759;AVGVARACC=0.0463625502818727;AVGPVAL=0.000201;AVGMIXED=0.000572885122082833;DIFFEFF=0.03261;DIFFVAR=0.000719739586251765;DIFFVARACC=0.000765782715826724;DIFFPVAL=0.7276;DIFFMIXED=0.732027672486224;ANNOT=(FBgn0000479|dnc|16871 bp DOWNSTREAM),(FBgn0262656|dm|12521 bp DOWNSTREAM)	GT	1/1	.	0/0	1/1	1/1	1/1	1/1	1/1	1/1	0/0	1/1	1/1	1/1	1/1	1/1	1/1	0/0	1/1	1/1	0/0	1/1	0/0	1/1	1/1	1/1	1/1	1/1	1/1	1/1	1/1	0/0	1/1	1/1	1/1	1/1	1/1	1/1	1/1	1/1	1/1	1/1	1/1	1/1	1/1	1/1	1/1	1/1	1/1	1/1	1/1	1/1	1/1	1/1	0/0	1/1	1/1	1/1	1/1	1/1	1/1	1/1	1/1	1/1	1/1	1/1	1/1	1/1	1/1	1/1	1/1	1/1	1/1	1/1	1/1	0/0	1/1	1/1	1/1	.	1/1	1/1	1/1	1/1	1/1	0/0	1/1	1/1	1/1	1/1	.	0/0	1/1	1/1	1/1	1/1	1/1	1/1	1/1	1/1	1/1	1/1	0/0	1/1	1/1	0/0	0/0	1/1	1/1	1/1	1/1	1/1	1/1	1/1	0/0	1/1	1/1	1/1	.	1/1	1/1	1/1	1/1	1/1	1/1	1/1	1/1	1/1	1/1	1/1	1/1	1/1	1/1	1/1	1/1	1/1	1/1	1/1	1/1	1/1	1/1	1/1	1/1	1/1	1/1	1/1	1/1	1/1	0/0	1/1	1/1	1/1	1/1	1/1	1/1	1/1	1/1	1/1	1/1	1/1	1/1	1/1	1/1	1/1	1/1	1/1	0/0	1/1	1/1	1/1	1/1	1/1	1/1	1/1	1/1	1/1
X	3254674	X_3254674_SNP	T	A	.	.	T5EFF=-0.3128;T5VAR=0.129289095492922;T5VARACC=0.0746018504199716;T5PVAL=1.694e-06;T5MIXED=1.54679659346991e-05;T6EFF=-0.3361;T6VAR=0.0426951391091011;T6VARACC=0.013745185958555;T6PVAL=0.007204;T6MIXED=0.00902342564365453;AVGEFF=-0.3244;AVGVAR=0.0801386724107669;AVGVARACC=0.0473232019310081;AVGPVAL=0.0002007;AVGMIXED=0.000591929957604519;DIFFEFF=0.02338;DIFFVAR=0.000352313276154063;DIFFVARACC=0.00240635521929888;DIFFPVAL=0.8092;DIFFMIXED=0.811685349262632;ANNOT=(FBgn0000479|dnc|16874 bp DOWNSTREAM),(FBgn0262656|dm|12518 bp DOWNSTREAM)	GT	1/1	.	0/0	1/1	1/1	1/1	1/1	1/1	1/1	0/0	1/1	1/1	1/1	1/1	1/1	1/1	0/0	1/1	1/1	0/0	1/1	0/0	1/1	1/1	1/1	1/1	1/1	1/1	1/1	1/1	0/0	1/1	1/1	1/1	1/1	1/1	1/1	1/1	1/1	1/1	1/1	1/1	1/1	1/1	1/1	1/1	1/1	1/1	1/1	1/1	1/1	1/1	1/1	0/0	1/1	1/1	1/1	1/1	1/1	1/1	1/1	1/1	1/1	1/1	1/1	1/1	1/1	1/1	1/1	1/1	1/1	1/1	1/1	1/1	0/0	1/1	1/1	1/1	.	1/1	1/1	1/1	1/1	1/1	0/0	1/1	1/1	1/1	1/1	1/1	0/0	1/1	1/1	1/1	1/1	1/1	1/1	1/1	1/1	1/1	.	.	1/1	1/1	0/0	0/0	1/1	1/1	1/1	1/1	1/1	1/1	1/1	0/0	1/1	1/1	1/1	.	1/1	1/1	1/1	1/1	1/1	1/1	1/1	1/1	1/1	1/1	1/1	1/1	1/1	1/1	1/1	1/1	1/1	1/1	1/1	1/1	1/1	1/1	1/1	1/1	.	1/1	.	1/1	1/1	0/0	1/1	1/1	1/1	1/1	1/1	1/1	1/1	1/1	1/1	1/1	1/1	1/1	1/1	1/1	1/1	1/1	1/1	0/0	1/1	1/1	1/1	1/1	1/1	1/1	1/1	1/1	1/1
X	3254679	X_3254679_SNP	A	C	.	.	T5EFF=-0.2812;T5VAR=0.118013003402253;T5VARACC=0.0711323568973024;T5PVAL=4.224e-06;T5MIXED=6.04607510677912e-05;T6EFF=-0.3194;T6VAR=0.0428277688745151;T6VARACC=0.0166896571188648;T6PVAL=0.006611;T6MIXED=0.00894822071469477;AVGEFF=-0.3003;AVGVAR=0.0767705397296691;AVGVARACC=0.0468346226570535;AVGPVAL=0.0002437;AVGMIXED=0.000913236270601696;DIFFEFF=0.03816;DIFFVAR=0.00103661623756633;DIFFVARACC=0.000502938555581534;DIFFPVAL=0.6759;DIFFMIXED=0.679531039732133;ANNOT=(FBgn0000479|dnc|16879 bp DOWNSTREAM),(FBgn0262656|dm|12513 bp DOWNSTREAM)	GT	1/1	.	0/0	1/1	1/1	1/1	1/1	1/1	1/1	0/0	1/1	1/1	1/1	1/1	1/1	1/1	0/0	1/1	1/1	0/0	1/1	0/0	1/1	1/1	1/1	.	1/1	1/1	1/1	1/1	0/0	.	1/1	1/1	1/1	1/1	1/1	1/1	1/1	1/1	1/1	1/1	1/1	1/1	1/1	1/1	1/1	1/1	1/1	1/1	1/1	1/1	1/1	0/0	1/1	1/1	1/1	1/1	1/1	1/1	1/1	1/1	1/1	1/1	1/1	1/1	1/1	1/1	1/1	1/1	1/1	1/1	1/1	1/1	0/0	1/1	1/1	1/1	0/0	1/1	1/1	1/1	1/1	1/1	0/0	1/1	1/1	1/1	1/1	1/1	0/0	1/1	1/1	1/1	1/1	1/1	1/1	1/1	1/1	1/1	1/1	0/0	1/1	1/1	0/0	0/0	1/1	1/1	1/1	1/1	1/1	1/1	1/1	0/0	1/1	1/1	1/1	.	1/1	1/1	1/1	1/1	1/1	1/1	1/1	1/1	1/1	1/1	1/1	1/1	1/1	1/1	1/1	1/1	1/1	1/1	1/1	1/1	1/1	1/1	1/1	1/1	1/1	1/1	1/1	1/1	1/1	0/0	1/1	1/1	1/1	1/1	1/1	1/1	1/1	1/1	1/1	1/1	1/1	1/1	1/1	1/1	1/1	1/1	1/1	0/0	1/1	1/1	1/1	1/1	1/1	1/1	1/1	1/1	1/1
X	3254755	X_3254755_SNP	T	C	.	.	T5EFF=-0.3041;T5VAR=0.1210129131665;T5VARACC=0.0709451165600483;T5PVAL=3.131e-06;T5MIXED=1.3142699562287e-05;T6EFF=-0.3508;T6VAR=0.0459191311673113;T6VARACC=0.0201789905440933;T6PVAL=0.004887;T6MIXED=0.00556156983188604;AVGEFF=-0.3274;AVGVAR=0.0809371929252909;AVGVARACC=0.0524954325222557;AVGPVAL=0.0001625;AVGMIXED=0.000338293491790341;DIFFEFF=0.0467;DIFFVAR=0.0013723520240924;DIFFVARACC=0.000402321600656674;DIFFPVAL=0.6305;DIFFMIXED=0.628018786332718;ANNOT=(FBgn0000479|dnc|16955 bp DOWNSTREAM),(FBgn0262656|dm|12437 bp DOWNSTREAM)	GT	1/1	1/1	0/0	1/1	1/1	1/1	1/1	1/1	1/1	0/0	1/1	1/1	1/1	1/1	1/1	1/1	0/0	1/1	1/1	0/0	1/1	1/1	1/1	1/1	1/1	1/1	1/1	1/1	1/1	1/1	0/0	1/1	1/1	1/1	1/1	1/1	1/1	1/1	1/1	1/1	1/1	1/1	1/1	1/1	1/1	1/1	1/1	.	1/1	1/1	1/1	1/1	1/1	0/0	1/1	1/1	1/1	1/1	1/1	1/1	1/1	1/1	1/1	1/1	1/1	1/1	1/1	1/1	1/1	1/1	1/1	1/1	1/1	1/1	.	1/1	1/1	1/1	.	1/1	1/1	1/1	1/1	1/1	0/0	1/1	1/1	1/1	1/1	1/1	0/0	1/1	1/1	1/1	1/1	1/1	1/1	1/1	1/1	1/1	.	0/0	1/1	1/1	0/0	0/0	1/1	1/1	1/1	1/1	1/1	1/1	1/1	0/0	1/1	1/1	1/1	0/0	1/1	1/1	1/1	1/1	1/1	1/1	1/1	1/1	1/1	1/1	1/1	1/1	1/1	1/1	1/1	1/1	1/1	1/1	1/1	1/1	1/1	1/1	1/1	1/1	1/1	1/1	1/1	1/1	1/1	0/0	1/1	1/1	1/1	1/1	1/1	1/1	1/1	1/1	1/1	1/1	1/1	1/1	1/1	1/1	1/1	1/1	1/1	0/0	1/1	1/1	1/1	1/1	1/1	1/1	1/1	1/1	1/1
X	4773496	X_4773496_SNP	T	C	.	.	T5EFF=-0.2286;T5VAR=0.0926371058250522;T5VARACC=0.054754020160548;T5PVAL=6.043e-05;T5MIXED=4.90099690812012e-07;T6EFF=-0.3196;T6VAR=0.0506428447072327;T6VARACC=0.0120925240070665;T6PVAL=0.003359;T6MIXED=0.00133212503435311;AVGEFF=-0.2741;AVGVAR=0.0761351663520289;AVGVARACC=0.0392621999787526;AVGPVAL=0.0002943;AVGMIXED=1.60723995691189e-05;DIFFEFF=0.09092;DIFFVAR=0.00680742142897935;DIFFVARACC=0.000219851074676869;DIFFPVAL=0.2877;DIFFMIXED=0.268565017674858;ANNOT=(FBgn0260971|CG42594|INTRON)	GT	1/1	1/1	1/1	0/0	1/1	1/1	1/1	1/1	1/1	1/1	1/1	1/1	1/1	1/1	1/1	.	1/1	1/1	1/1	1/1	1/1	1/1	1/1	1/1	1/1	0/0	.	1/1	1/1	1/1	1/1	1/1	1/1	1/1	1/1	1/1	1/1	0/0	1/1	1/1	1/1	1/1	0/0	1/1	1/1	1/1	1/1	1/1	1/1	1/1	1/1	1/1	1/1	.	1/1	1/1	1/1	1/1	1/1	1/1	1/1	1/1	1/1	1/1	1/1	1/1	1/1	1/1	1/1	1/1	1/1	1/1	1/1	1/1	1/1	0/0	0/0	1/1	1/1	1/1	1/1	1/1	1/1	1/1	1/1	1/1	1/1	1/1	1/1	1/1	0/0	1/1	1/1	1/1	1/1	1/1	1/1	1/1	.	1/1	1/1	1/1	1/1	1/1	1/1	0/0	1/1	1/1	1/1	1/1	1/1	.	1/1	1/1	0/0	1/1	1/1	1/1	1/1	1/1	1/1	1/1	1/1	0/0	1/1	1/1	1/1	1/1	1/1	1/1	0/0	1/1	.	1/1	1/1	1/1	1/1	0/0	1/1	1/1	1/1	1/1	1/1	1/1	1/1	1/1	0/0	1/1	0/0	1/1	0/0	0/0	1/1	1/1	1/1	1/1	1/1	1/1	1/1	1/1	0/0	1/1	1/1	1/1	0/0	0/0	1/1	1/1	1/1	.	1/1	1/1	1/1	0/0	1/1
X	6359553	X_6359553_SNP	G	A	.	.	T5EFF=-0.2395;T5VAR=0.109046552470773;T5VARACC=0.0895336679240121;T5PVAL=9.122e-06;T5MIXED=3.16686282024951e-05;T6EFF=-0.2325;T6VAR=0.0296252183321991;T6VARACC=0.0198690183782368;T6PVAL=0.02355;T6MIXED=0.022798979035355;AVGEFF=-0.236;AVGVAR=0.0613260782387598;AVGVARACC=0.0514540942059192;AVGPVAL=0.00102;AVGMIXED=0.00139719740227207;DIFFEFF=-0.006944;DIFFVAR=4.50935726127113e-05;DIFFVARACC=0.0053411356000182;DIFFPVAL=0.9301;DIFFMIXED=0.934464946244443;ANNOT=(FBgn0259242|CG42340|INTRON)	GT	0/0	0/0	1/1	1/1	0/0	0/0	0/0	0/0	.	1/1	0/0	0/0	0/0	0/0	0/0	0/0	1/1	0/0	0/0	0/0	0/0	0/0	0/0	1/1	0/0	1/1	1/1	0/0	0/0	0/0	1/1	0/0	0/0	0/0	0/0	1/1	0/0	0/0	0/0	0/0	0/0	0/0	0/0	0/0	0/0	0/0	0/0	1/1	0/0	0/0	0/0	1/1	0/0	0/0	0/0	0/0	0/0	0/0	1/1	0/0	0/0	0/0	0/0	0/0	0/0	0/0	0/0	0/0	0/0	0/0	0/0	0/0	0/0	0/0	0/0	0/0	0/0	0/0	0/0	0/0	0/0	0/0	0/0	0/0	0/0	0/0	0/0	0/0	0/0	0/0	1/1	0/0	0/0	0/0	0/0	0/0	1/1	0/0	0/0	.	0/0	0/0	1/1	1/1	0/0	1/1	0/0	0/0	0/0	0/0	0/0	0/0	1/1	1/1	0/0	0/0	0/0	0/0	0/0	0/0	0/0	0/0	0/0	0/0	0/0	0/0	0/0	0/0	0/0	0/0	0/0	0/0	0/0	0/0	1/1	0/0	0/0	1/1	0/0	0/0	0/0	0/0	0/0	0/0	0/0	0/0	0/0	1/1	0/0	0/0	0/0	0/0	0/0	0/0	0/0	0/0	0/0	0/0	0/0	0/0	0/0	0/0	0/0	0/0	0/0	1/1	0/0	0/0	0/0	0/0	0/0	0/0	0/0	0/0	0/0
X	9121094	X_9121094_SNP	T	C	.	.	T5EFF=0.1701;T5VAR=0.0837679727368824;T5VARACC=0.00082151904224953;T5PVAL=0.0001291;T5MIXED=6.83638958080666e-05;T6EFF=0.3852;T6VAR=0.120869984256629;T6VARACC=0.00614764583954919;T6PVAL=3.396e-06;T6MIXED=1.70512249280844e-06;AVGEFF=0.2777;AVGVAR=0.127890242061958;AVGVARACC=0.00294287397934456;AVGPVAL=1.687e-06;AVGMIXED=6.75261122665896e-07;DIFFEFF=-0.2151;DIFFVAR=0.0629499599786603;DIFFVARACC=0.0015741509486159;DIFFPVAL=0.0009664;DIFFMIXED=0.00074648409143382;ANNOT=(FBgn0030108|Gr8a|76 bp UPSTREAM),(FBgn0030107|CG15370|493 bp DOWNSTREAM)	GT	0/0	1/1	0/0	0/0	0/0	0/0	1/1	0/0	0/0	0/0	0/0	0/0	0/0	1/1	0/0	.	0/0	1/1	1/1	0/0	0/0	0/0	0/0	0/0	0/0	0/0	1/1	0/0	0/0	0/0	0/0	1/1	0/0	0/0	0/0	0/0	0/0	0/0	0/0	0/0	.	0/0	0/0	0/0	0/0	1/1	0/0	0/0	1/1	1/1	1/1	1/1	0/0	1/1	1/1	0/0	0/0	0/0	0/0	1/1	.	1/1	0/0	0/0	0/0	1/1	0/0	0/0	0/0	0/0	0/0	1/1	0/0	0/0	0/0	1/1	0/0	0/0	.	0/0	0/0	0/0	0/0	0/0	0/0	0/0	0/0	0/0	0/0	1/1	0/0	0/0	0/0	1/1	0/0	0/0	1/1	0/0	0/0	1/1	0/0	0/0	0/0	1/1	0/0	0/0	0/0	0/0	0/0	0/0	1/1	0/0	1/1	0/0	0/0	0/0	0/0	0/0	1/1	1/1	1/1	0/0	0/0	0/0	0/0	0/0	1/1	0/0	1/1	0/0	0/0	0/0	0/0	0/0	0/0	0/0	0/0	0/0	1/1	1/1	0/0	0/0	0/0	0/0	.	0/0	0/0	0/0	0/0	0/0	0/0	0/0	0/0	0/0	0/0	0/0	0/0	1/1	1/1	0/0	0/0	0/0	0/0	0/0	1/1	0/0	1/1	0/0	0/0	0/0	1/1	0/0	0/0	0/0	0/0
X	9121129	X_9121129_SNP	C	T	.	.	T5EFF=0.184;T5VAR=0.116203030097719;T5VARACC=NA;T5PVAL=4.742e-06;T5MIXED=4.52350251464524e-06;T6EFF=0.4147;T6VAR=0.166162639348126;T6VARACC=NA;T6PVAL=2.857e-08;T6MIXED=1.39269386275417e-08;AVGEFF=0.2993;AVGVAR=0.176217741891527;AVGVARACC=NA;AVGPVAL=9.909e-09;AVGMIXED=3.99710301726669e-09;DIFFEFF=-0.2307;DIFFVAR=0.0860438155878698;DIFFVARACC=NA;DIFFPVAL=9.414e-05;DIFFMIXED=7.04973139836087e-05;ANNOT=(FBgn0030108|Gr8a|41 bp UPSTREAM),(FBgn0030107|CG15370|528 bp DOWNSTREAM)	GT	0/0	1/1	0/0	0/0	0/0	0/0	1/1	0/0	0/0	0/0	0/0	0/0	0/0	1/1	0/0	.	0/0	1/1	1/1	0/0	0/0	0/0	0/0	.	0/0	0/0	1/1	0/0	0/0	0/0	0/0	1/1	0/0	0/0	0/0	0/0	0/0	0/0	0/0	0/0	1/1	0/0	0/0	0/0	0/0	1/1	0/0	0/0	1/1	1/1	1/1	1/1	1/1	1/1	1/1	1/1	0/0	0/0	1/1	1/1	0/0	1/1	0/0	0/0	0/0	1/1	0/0	1/1	0/0	0/0	0/0	1/1	0/0	0/0	0/0	1/1	0/0	0/0	1/1	0/0	0/0	0/0	0/0	0/0	0/0	0/0	0/0	0/0	1/1	1/1	0/0	0/0	0/0	1/1	0/0	0/0	1/1	0/0	0/0	0/0	0/0	0/0	0/0	1/1	0/0	0/0	0/0	0/0	0/0	.	1/1	0/0	1/1	0/0	0/0	0/0	0/0	0/0	1/1	1/1	1/1	0/0	0/0	0/0	0/0	0/0	1/1	0/0	1/1	1/1	0/0	0/0	0/0	0/0	0/0	0/0	0/0	0/0	1/1	1/1	0/0	0/0	0/0	0/0	1/1	0/0	0/0	0/0	0/0	0/0	0/0	0/0	0/0	0/0	1/1	0/0	0/0	1/1	1/1	0/0	0/0	0/0	0/0	0/0	1/1	0/0	1/1	0/0	0/0	1/1	1/1	0/0	1/1	0/0	0/0
X	9121177	X_9121177_SNP	C	T	.	.	T5EFF=0.1692;T5VAR=0.108253962526698;T5VARACC=0.00357377719197371;T5PVAL=1.049e-05;T5MIXED=7.97770016768044e-06;T6EFF=0.3486;T6VAR=0.130872837181573;T6VARACC=0.00225990381830415;T6PVAL=1.082e-06;T6MIXED=5.5832030259339e-07;AVGEFF=0.2589;AVGVAR=0.145702772643049;AVGVARACC=0.00196250476503873;AVGPVAL=2.384e-07;AVGMIXED=1.10729040627535e-07;DIFFEFF=-0.1794;DIFFVAR=0.0589637749363243;DIFFVARACC=0.000277859836093908;DIFFPVAL=0.001329;DIFFMIXED=0.00112998138928966;ANNOT=(FBgn0030108|Gr8a|UTR_5_PRIME),(FBgn0030107|CG15370|576 bp DOWNSTREAM)	GT	0/0	1/1	0/0	0/0	0/0	0/0	1/1	1/1	0/0	0/0	0/0	0/0	0/0	1/1	0/0	0/0	0/0	1/1	1/1	0/0	0/0	0/0	0/0	1/1	0/0	0/0	1/1	0/0	0/0	0/0	0/0	1/1	1/1	1/1	1/1	0/0	0/0	0/0	0/0	0/0	1/1	0/0	0/0	0/0	1/1	1/1	0/0	0/0	1/1	1/1	1/1	1/1	1/1	0/0	.	1/1	0/0	0/0	1/1	1/1	0/0	1/1	0/0	1/1	0/0	1/1	0/0	1/1	0/0	0/0	0/0	1/1	0/0	0/0	0/0	1/1	0/0	0/0	1/1	.	0/0	0/0	0/0	0/0	0/0	0/0	0/0	.	1/1	1/1	0/0	0/0	0/0	1/1	0/0	0/0	1/1	0/0	0/0	0/0	0/0	0/0	0/0	1/1	0/0	0/0	0/0	0/0	0/0	0/0	1/1	0/0	1/1	1/1	0/0	0/0	0/0	1/1	1/1	1/1	1/1	0/0	0/0	0/0	0/0	1/1	1/1	0/0	1/1	1/1	0/0	0/0	0/0	0/0	0/0	0/0	0/0	0/0	1/1	1/1	0/0	0/0	1/1	0/0	1/1	0/0	1/1	0/0	1/1	0/0	0/0	0/0	0/0	0/0	1/1	0/0	1/1	1/1	1/1	0/0	0/0	0/0	0/0	0/0	1/1	0/0	1/1	0/0	0/0	1/1	1/1	0/0	1/1	0/0	0/0
X	9121338	X_9121338_SNP	A	G	.	.	T5EFF=0.1608;T5VAR=0.104528454322279;T5VARACC=0.0060069341312643;T5PVAL=1.518e-05;T5MIXED=9.77088869290475e-06;T6EFF=0.2641;T6VAR=0.0805477221439142;T6VARACC=0.000844277026564588;T6PVAL=0.0001614;T6MIXED=7.22194190511884e-05;AVGEFF=0.2124;AVGVAR=0.105464984602401;AVGVARACC=0.00200533620765275;AVGPVAL=1.384e-05;AVGMIXED=3.34312609822211e-06;DIFFEFF=-0.1033;DIFFVAR=0.0207186602867211;DIFFVARACC=0.00821977570804283;DIFFPVAL=0.05959;DIFFMIXED=0.0504639981915524;ANNOT=(FBgn0030108|Gr8a|NON_SYNONYMOUS_CODING),(FBgn0030107|CG15370|737 bp DOWNSTREAM)	GT	0/0	1/1	0/0	0/0	0/0	1/1	1/1	0/0	0/0	0/0	1/1	1/1	1/1	1/1	1/1	0/0	0/0	1/1	1/1	0/0	0/0	1/1	1/1	1/1	0/0	0/0	1/1	0/0	0/0	0/0	0/0	1/1	0/0	0/0	0/0	0/0	0/0	.	0/0	0/0	1/1	0/0	0/0	1/1	0/0	0/0	0/0	1/1	1/1	1/1	1/1	1/1	1/1	1/1	1/1	1/1	0/0	0/0	1/1	1/1	0/0	1/1	0/0	0/0	0/0	1/1	0/0	1/1	0/0	0/0	0/0	1/1	0/0	0/0	0/0	0/0	1/1	0/0	.	1/1	0/0	0/0	0/0	0/0	0/0	0/0	0/0	0/0	1/1	1/1	0/0	0/0	1/1	1/1	0/0	1/1	1/1	1/1	0/0	0/0	1/1	0/0	0/0	1/1	0/0	0/0	0/0	0/0	0/0	0/0	1/1	0/0	1/1	0/0	0/0	0/0	0/0	1/1	1/1	1/1	1/1	0/0	1/1	0/0	0/0	1/1	1/1	0/0	1/1	1/1	0/0	0/0	0/0	1/1	0/0	.	1/1	0/0	1/1	1/1	1/1	0/0	0/0	0/0	1/1	0/0	0/0	0/0	0/0	1/1	0/0	0/0	0/0	1/1	1/1	0/0	1/1	1/1	1/1	1/1	0/0	0/0	1/1	1/1	1/1	0/0	1/1	0/0	0/0	1/1	1/1	0/0	1/1	0/0	0/0
