# Supplementary material for: Genetic Architecture of Abdominal Pigmentation in Drosophila melanogaster
Source: PLoS Genet. 2015 May 1;11(5):e1005163. doi: 10.1371/journal.pgen.1005163 (PMC4416719; doi:10.1371/journal.pgen.1005163)
Supplement: S6 Table — (DOC) [file pgen.1005163.s012.doc]

**Table S6.** Number of GWAS variants affecting each type of identified regulatory element.

| **Regulatory Site Symbol** | **Number of Variants** |
| --- | --- |
| BDTNP1_TFBS_bcd | 1 |
| BDTNP1_TFBS_da | 10 |
| BDTNP1_TFBS_dl | 28 |
| BDTNP1_TFBS_hb | 1 |
| BDTNP1_TFBS_Med | 9 |
| BDTNP1_TFBS_prd | 4 |
| BDTNP1_TFBS_slp1 | 2 |
| BDTNP1_TFBS_sna | 2 |
| BDTNP1_TFBS_twi | 12 |
| BDTNP1_TFBS_z | 1 |
| mE1_HDAC_PRE | 1 |
| mE1_TFBS_bab1 | 3 |
| mE1_TFBS_cad | 22 |
| mE1_TFBS_chinmo | 12 |
| mE1_TFBS_cnc | 2 |
| mE1_TFBS_D | 3 |
| mE1_TFBS_h | 1 |
| mE1_TFBS_HSA | 31 |
| mE1_TFBS_inv | 3 |
| mE1_TFBS_jumu | 1 |
| mE1_TFBS_sbb | 1 |
| mE1_TFBS_sens | 7 |
| mE1_TFBS_Trl | 2 |
| mE1_TFBS_ttk | 1 |
| mE1_TFBS_Ubx | 1 |
| mE1_TFBS_zfh1 | 2 |
| REDfly_CRMs | 17 |
