# Supplementary material for: Genetic Architecture of Abdominal Pigmentation in Drosophila melanogaster
Source: PLoS Genet. 2015 May 1;11(5):e1005163. doi: 10.1371/journal.pgen.1005163 (PMC4416719; doi:10.1371/journal.pgen.1005163)
Supplement: S7 Table — (DOC) [file pgen.1005163.s013.doc]

| **Candidate Gene** | **Chr** | **GWAS** | **VDRC**  **Transformant ID** | **VDRC**  **Construct ID** | **VDRC**  **Library** | **Exelixis**  **Line** | **Exelixis Description** |
| --- | --- | --- | --- | --- | --- | --- | --- |
| *CG33298* | *2L* | T6 | 42776 | 1036 | GD | d10678 | *P{XP}CG33298^d10678a^* |
| *Fish-lips* (*Fili*) | *2R* | T6 | 44532 | 2136 | GD | f04573 | *PBac{WH}Fili^f04573^* |
| *Vesicular monoamine transporter* (*Vmat*) | *2R* | T6 | 104072 | 112993 | KK | - | - |
| *multiple wing hairs* (*mwh*) | *3L* | T6 | 41513 | 8165 | GD | d01620 | *P{XP}mwh^d01620^* |
| *Kinesin-like protein at 61F* (*Klp61F*) | *3L* | T6 | 109280 | 100504 | KK | f02870 | *PBac{WH}Klp61F^f02870^* |
| *CG9134* | *3L* | T6 | 108295 | 101456 | KK | e00088 | *PBac{RB}CG9134^e00088^* |
| *CG7852* | *3L* | T6, T5-T6 | 103582 | 101479 | KK | c04511 | *PBac{PB}CG7852^c04511^* |
| *CG1887* | *3L* | T6 | 100219 | 107147 | KK | - | - |
| *klarsicht* (*klar*) | *3L* | T6 | 32836 | 9271 | GD | d05910 | *P{XP}klar^d05910^* |
| *Glucose transporter 1* (*Glut1*) | *3L* | T6 | - | - | - | d05758 | *P{XP}Glut1^d05758^* |
| *Exchange factor for Arf 6* (*Efa6*) | *3R* | T6 | 42321 | 14945 | GD | f03476 | *PBac{WH}Efa6^f03476^* |
| *buttonless* (*btn*) | *3R* | T6 | 105140 | 102048 | KK | - | - |
| *roughoid* (*ru*) | *3L* | T5 | 101684 | 105434 | KK | - | - |
| *CG10625* | *3L* | T5 | 102369 | 111582 | KK | e01211 | *PBac{RB}CG10625^e01211^* |
| *sinuous* (*sinu*) | *3L* | T5 | 44928 | 2315 | GD | - | - |
| *Sucb* (*Sucb*) | *3L* | T5 | - | - | - | e01940 | *PBac{RB}Sucb^e01940^* |
| *division abnormally delayed* (*dally*) | *3L* | T5 | 14136 | 5918 | GD | f01097 | *PBac{WH}dally^f01097^* |
| *CG32052* | *3L* | T5 | 105824 | 100526 | KK | - | - |
| *Nedd2-like caspase* (*Nc*) | *3L* | T5 | 100424 | 104278 | KK | - | - |
| *Ceramide kinase* (*Cerk*) | *3R* | T5 | 101550 | 109026 | KK | - | - |
| *krotzkopf verkehrt* (*kkv*) | *3R* | T5 | 100327 | 105928 | KK | c06225 | *PBac{PB}kkv^c06225^* |
| *CG15803* | *3R* | T5 | 43635 | 8745 | GD | - | - |
| *locomotion defects* (*loco*) | *3R* | T5 | 9248 | 1282 | GD | d09879 | *P{XP}loco^d09879^* |
| *TweedleC* (*TwdlC*) | *3R* | T5 | 101860 | 110000 | KK | - | - |
| *kayak* (*kay*) | *3R* | T5 | 6212 | 1469 | GD | f02002 | *PBac{WH}kay^f02002^* |
| *CG1340* | *3R* | T5 | 102825 | 103972 | KK | - | - |
| *CG42594* | *X* | T5 | 7042 | 3229 | GD | - | - |
| *CG42340* | *X* | T5 | 105046 | 113126 | KK | - | - |

**Table S7.** List of candidate genes, VDRC RNAi lines, and Exelixis insertion lines
